# Continuous Flow Synthesis of Nitrosoarenes via Photochemical Rearrangement of Aryl Imines

**DOI:** 10.1021/acs.joc.3c02362

**Published:** 2023-12-22

**Authors:** Jorge García-Lacuna, Marcus Baumann

**Affiliations:** University College Dublin, School of Chemistry, Science Centre South, Belfield, Dublin 4, Ireland

## Abstract

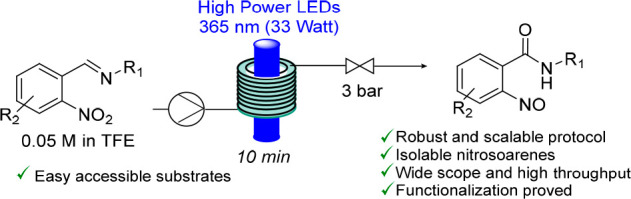

Nitrosoarenes are versatile organic building blocks; however, their intrinsic instability and limited synthetic accessibility have so far restricted their widespread use. Herein, we present a new continuous flow route toward these entities that is based on a direct photochemical rearrangement process using *o*-nitrophenylimines as starting materials. Due to the underlying redox mechanism, a new amide group accompanies the formation of the nitroso group. Crucial to the success of this approach is the use of trifluoroethanol as a solvent and high-power light-emitting diodes (365 nm) as light sources that provide uniform irradiation and high efficiency of the resulting continuous flow method. The process is fast and robust, with high functional group tolerance and high throughput. The formation of the nitroso moiety is supported by full spectroscopic analysis, including X-ray crystallography. The scalability of this flow approach allows access to gram quantities of nitroso species for which we highlight a small set of derivatization reactions underlining their synthetic utility.

## Introduction

**Scheme 1:**
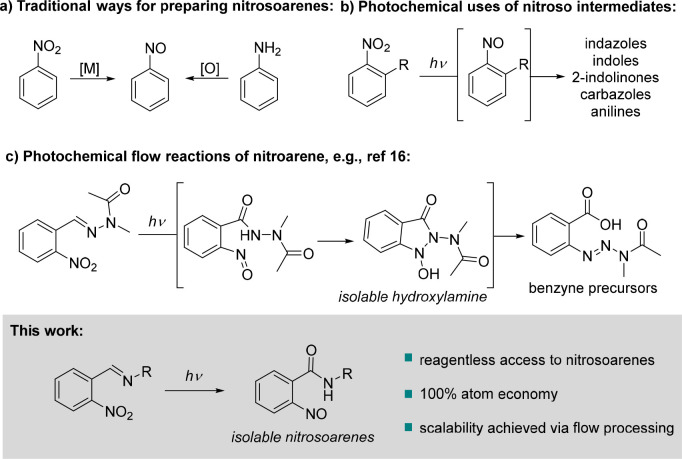
State of the Art for Nitrosoarene Chemistry Related to This Work

Nitrosoarenes are intriguing compounds that can be considered both good nucleophiles and electrophiles, resulting in many applications for the formation of new C–N bonds and heterocycles.^1^ Despite nitrosoarenes being powerful building blocks in organic synthesis, their harmful nature and instability^2^ have restricted their exploitation. Moreover, many nitrosoarenes form *cis*- and *trans*-azodioxy dimers in solution and in the solid state, which can alter their reactivity and stability.^3^ The most common ways to prepare these species involve reduction of nitro derivatives or oxidation of related aniline precursors, which often leads to overreduction or overoxidation (Scheme 1a). Other unusual alternatives are direct nitrosation reactions and the substitution of an organometallic species by a nitroso group, which typically requires bespoke conditions, toxic reagents, and/or the presence of metal catalysts.^1b,4^ An unusual option was mentioned in a report from 1970^5^ that suggests the formation of *o*-nitrosobenzanilide from *o*-nitrobenzylideneaniline upon ultraviolet (UV) irradiation in benzene; however, synthetic details are missing, and the reaction outcome was supported via only infrared and reflectance spectroscopy for the solid formed.

Due to the intrinsic value of nitrosoarenes as building blocks for organic synthesis, we developed a complementary approach exploiting *o*-nitrophenylimines that would undergo an internal redox process upon photoexcitation. Recent years have already showcased ever-growing interest in photochemistry as a green and effective synthetic methodology, whereby nitroarenes have been exploited as oxygen atom transfer reagents^6^ and toward the generation of medicinally relevant heterocycles^7^ such as indazolones,^8^ indoles, 2-indolinones,^9^ 2-*H*-indazoles,^10^ carbazoles,^11^ or pyrido[1,2-*b*]indazoles.^12^ Furthermore, 2-nitrobenzaldehyde, which we utilize as the starting material to prepare the corresponding nitrophenylimines, is a recognized actinometric compound, and its photochemical reactivity has been examined in diverse studies.^13^ Other related photochemical transformations include the reduction of nitro groups to form anilines^14^ or the synthesis of 2-aminobenzamides^15^ (Scheme 1b). The latter transformation was achieved by exciting nitrobenzaldehydes with secondary amines under UV light. However, a huge excess of the amine and acetic acid as an additive were needed.

Recently, our group developed a continuous flow procedure that involves a photochemical rearrangement of photoexcited nitroarenes to yield unusual acetyl triazene benzoic acid derivatives that are precursors to benzyne species (Scheme 1c).^16^ This study showed that the mechanism proceeds through a transient nitroso species and an isolable cyclic hydroxylamine intermediate.

In the context of photochemistry, the use of continuous flow processing is commonly favored over batch-mode operation due to its advantages such as uniform irradiation, increased photon transfer, and scalability.^17^ Another noteworthy advantage of continuous flow processing is related to miniaturization; i.e., when using or making unstable and/or toxic compounds, conditions can be quickly screened in a safer environment, and only minute amounts of hazardous reactants are handled within the flow reactor.^18^ These factors are responsible for shifting the attention of the pharmaceutical industry toward continuous manufacturing in recent years.^19^

On the basis of our previous report,^16^ we decided to investigate a similar photochemical process in flow mode involving a variety of different aldimines. We were interested in establishing whether products containing a carboxylic acid and a diazene or a cyclic hydroxylamine would form in analogy to our previous study. As initial experimentation showed that nitrosoarene **2a** was generated as the predominant species upon irradiation of **1a** at 365 nm, we commenced our investigations using that compound as a model substrate (Table 1).

**Table 1:**
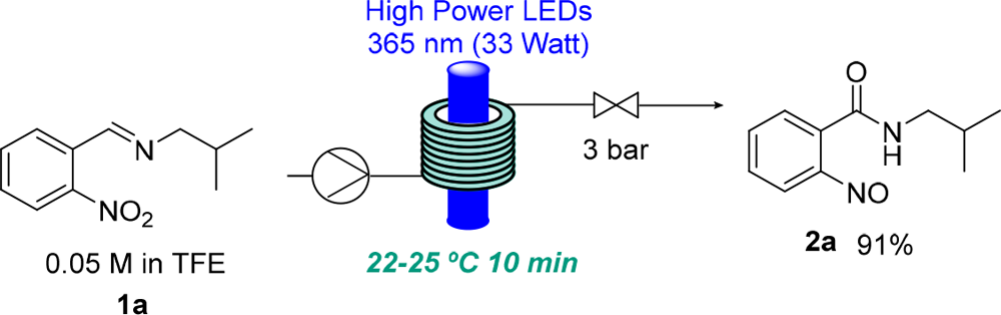
Optimization for the Preparation of **2a**

entry^a^	deviations from the optimized conditions	yield^b^ (%)
1	MeCN as the solvent	53
2	DCM as the solvent	64
3	HFIP^c^ as the solvent	80
4	20 min residence time	59
5	5 min residence time	51 (12)
6	400 nm	69 (5)
7	420 nm	30 (19)
8	365 nm (set to 66 W)	60
9	no light	0 (99)
10	0.1 M	43 (9)

a Unless otherwise noted, all reactions were performed at a 0.3 mmol scale in TFE (0.05 M) at 22–25 °C with a residence time of 10 min (flow rate of 1 mL/min) using high-power 365 nm LEDs with an input power of 33 W and a system pressure of 3 bar (optimized conditions to achieve a 91% yield).

b The qNMR yield was calculated using 1,3,5-trimethoxybenzene as the internal standard. The yields of the remaining starting materials are given in parentheses.

c 1,1,1,3,3,3-Hexafluoro-2-propanol.

The flow setup consisted of a Vaportec E-series reactor and its UV-150 photo module equipped with a coil reactor (10 mL volume, PFA) and different light sources. One peristaltic pump was used as an adjustable back-pressure regulator (BPR). Table 1 shows a summary of the optimization study with deviations from the best conditions (see the Supporting Information for more details). The main variation from our previous report is the reaction solvent. After different solvents had been tested, trifluoroethanol (TFE) and hexafluoroisopropanol (HFIP) were found to be the best options, which may be explained by their ability to form a hydrogen bonding network that may stabilize the nitroso species.^20^ As these nitroso compounds are potentially unstable and easily decompose upon handling, the reaction medium appears to be crucial. Other light sources failed to improve the outcome (entries 6 and 7). A higher light intensity (entry 8) or a longer residence time (entry 4) resulted in a lower yield due to the appearance of some decomposition. The reaction was also carried out in the dark, with no conversion observed (entry 9). Finally, an increase in concentration showed a lower yield of the product due to decomposition with starting material remaining (entry 10). Attempts to isolate the resulting nitroso compounds were met with challenges, as their instability resulted in degradation and material loss during column chromatography and recrystallization. Nevertheless, an isolated yield of 69% could be obtained for product **2a** (250 mg scale) under flash chromatography conditions.

**Scheme 2:**
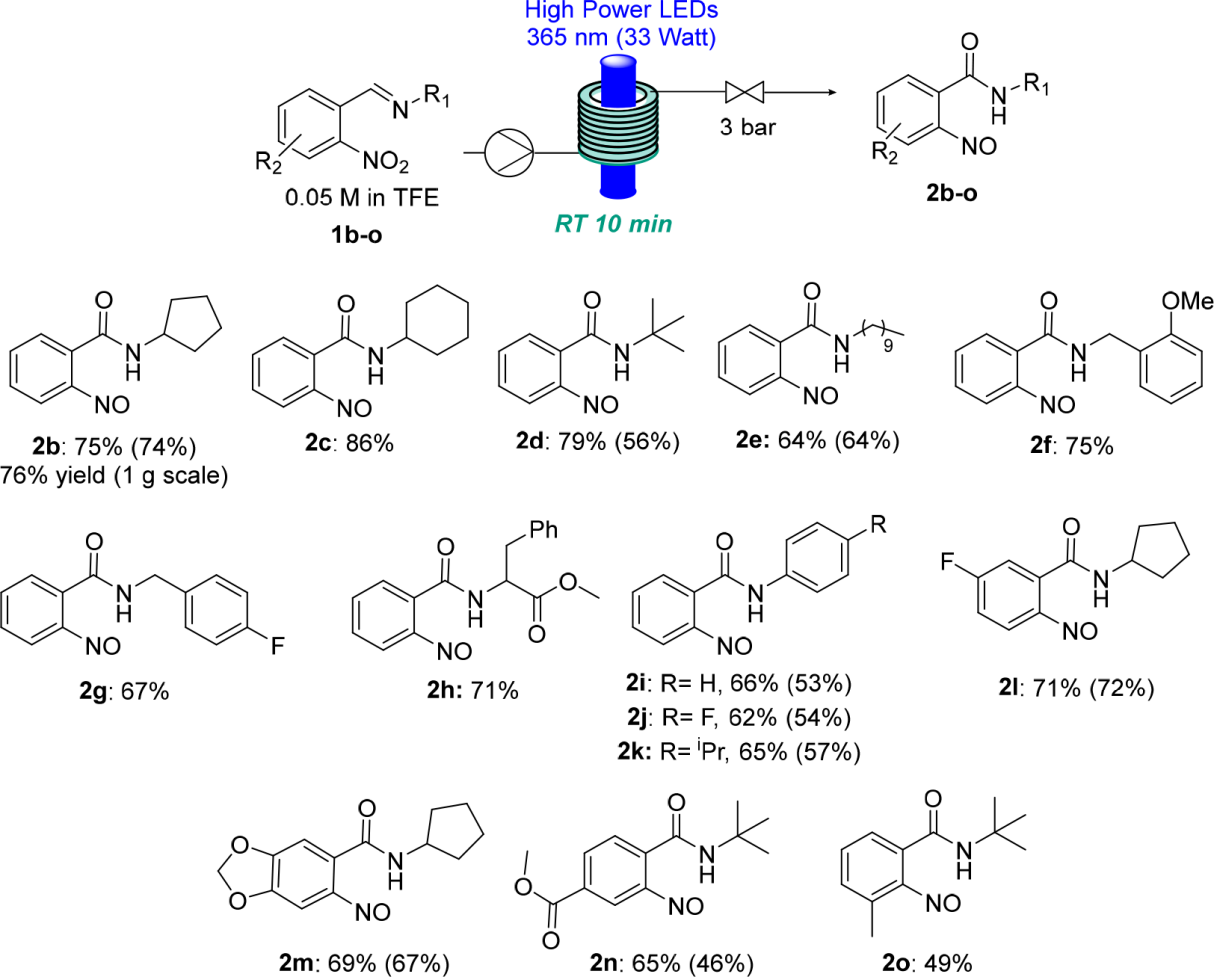
Substrate Scope for Continuous Photochemical Preparation of Nitrosoarenes All reactions were performed on a 0.3 mmol scale in TFE (0.05 M) at 22–25 °C with a residence time of 10 min (flow rate of 1 mL/min) using 365 nm LEDs with an input power of 33 W and a system pressure of 3 bar. The qNMR yield was calculated using 1,3,5-trimethoxybenzene as the internal standard; the yields in parentheses refer to those of isolable compounds.

Next, we aimed to investigate the scope of the newly developed photochemical transformation and its applicability to diverse functionalized imines. All substrates explored (**1a**–**o**) were readily accessed by condensation of the corresponding primary amine with a 2-nitrobenzaldehyde derivative. Importantly, a wide variety of nitrosobenzamides were obtained in moderate to excellent yields (Scheme 2) following the described procedure without any modification.

Although some nitrosoarenes were found to be unstable, it was possible to obtain samples of pure products for full spectroscopic characterization of these unique compounds. Imines synthesized using different aliphatic and benzylic amines (**2b**–**g**) and anilines (**2i**–**k**) were subjected to the optimized conditions, giving good to excellent yields in all cases. An imine derived from *rac*-phenylalanine (**2h**) was also tested, affording good results. Finally, different aryl substitution patterns, with both electron-donating and electron-withdrawing groups (**2l**–**o**), were tolerated, giving the desired products in moderate to good yields.

**Figure 1:**
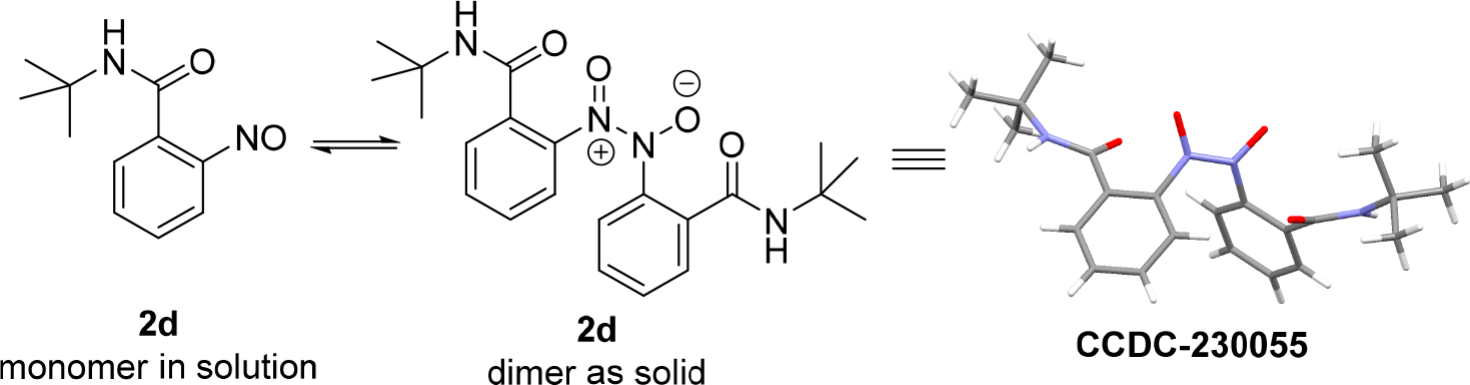
Crystal structure of compound **2d** (shown at the 50% probability level).

An interesting observation concerned the appearance of these species, which afforded a green color in solution that turns into white for the solid state, which agrees with prior reports.^1b,3a^ This appears to be due to the presence of nitroso monomers in solution versus dimers as solids. This equilibrium affects both the reactivity and the isolation by column chromatography. To unambiguously establish the connectivity of the nitroso compounds, one compound was subjected to single-crystal X-ray diffraction analysis,^21^ i.e., **2d**. As depicted in Figure 1, compound **2d** was found to be a dimer in the solid state.

**Scheme 3:**
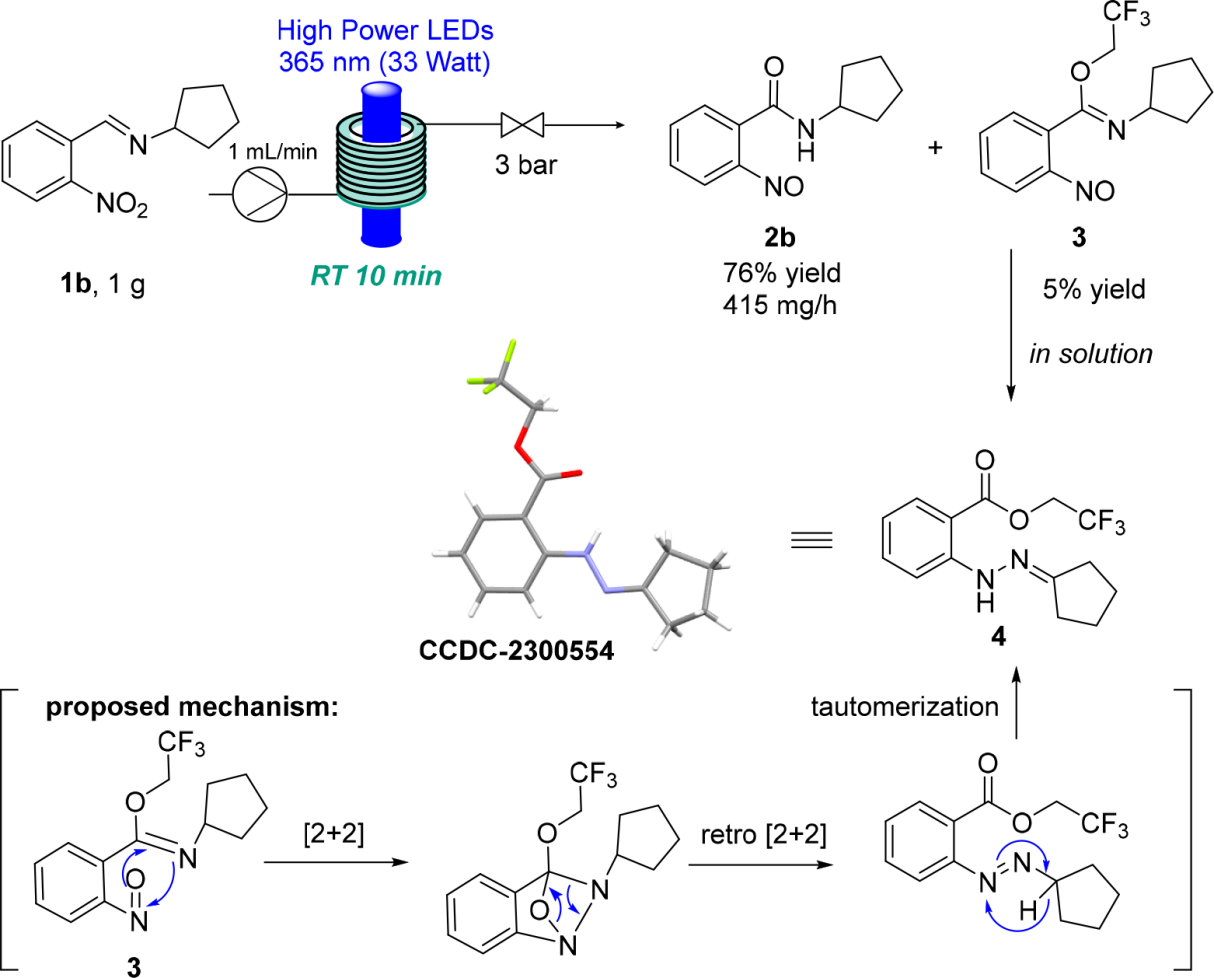
Gram-Scale Reaction of **1b** and Isolation of the Side Product and Crystal Structure of Compound **4** (shown at the 50% probability level)

A gram-scale experiment for substrate **1b** was performed showing the scalability and robustness of this transformation, which afforded the desired product in 76% isolated yield with a throughput of 415 mg h^–1^ and a space time yield of 190 mmol L^–1^ h^–1^ (Scheme 3). Importantly, this scale-up campaign enabled us to isolate an unexpected side product that we had not been able to isolate in previous small-scale reactions. The structure of this material was assigned to be that of nitroso species **3** (5% yield), which incorporated trifluoroethanol in the form of an imidate. This material rearranged in solution to yield ester **4**, whose structure was confirmed by X-ray diffraction. The proposed mechanism is shown in Scheme 3 and involves a sequence of formal [2+2] cycloaddition and cycloreversion reactions, followed by a final tautomerization to yield ester **4**.^14^ To the best of our knowledge, this is the first case in which a trifluoroethanol bearing an imidate is generated and transformed into a more stable ester. The isolation of this product because of the flow-assisted reaction scale-up also showcases the importance of being able to produce larger amounts of the desired products to discover unexpected reactivities.

**Scheme 4:**
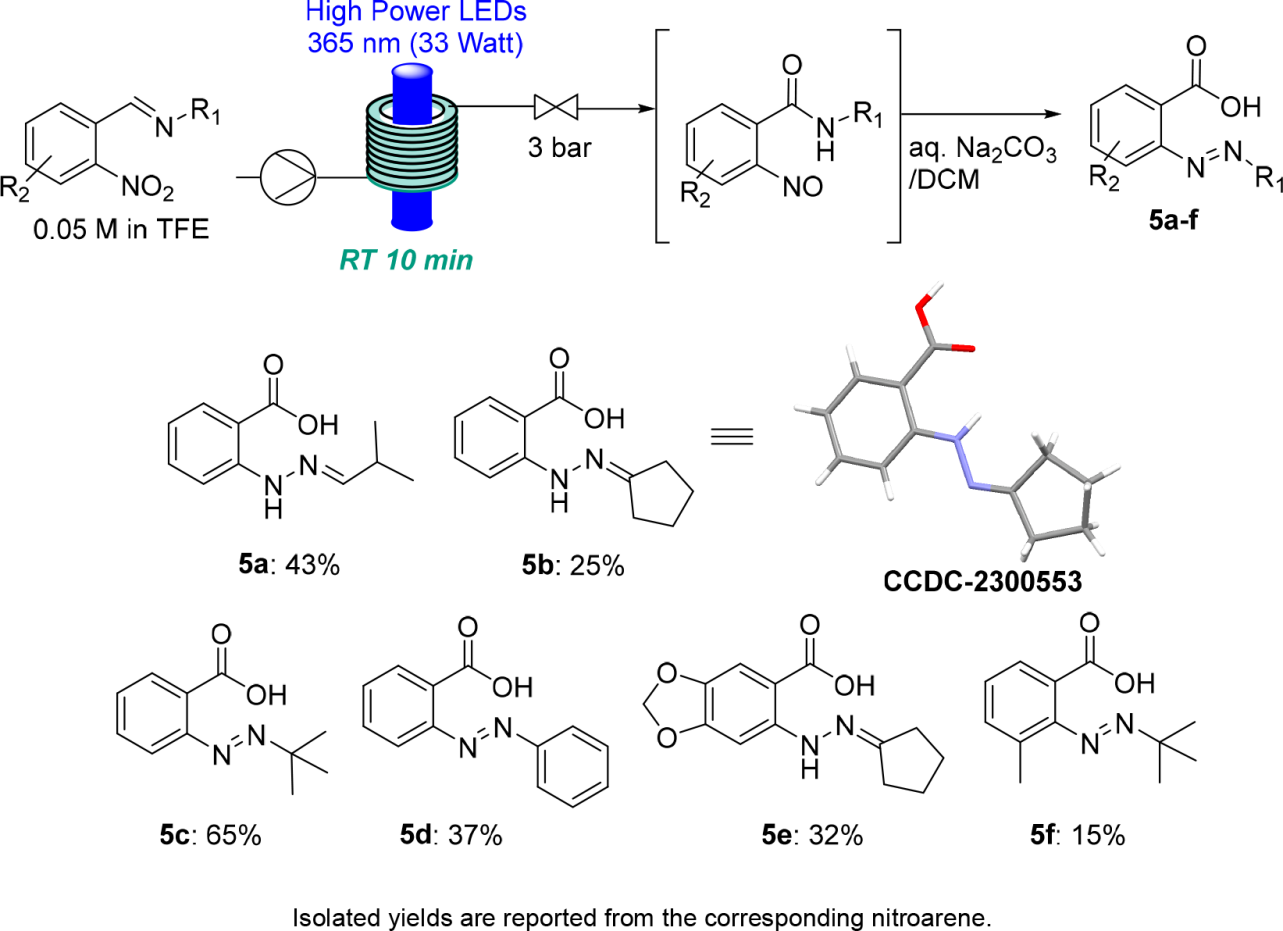
Benzoic Acids Obtained after Aqueous Treatment of Nitrosoarenes and Crystal Structure of Compound **5b** (shown at the 50% probability level)

Next, we explored the outcome when treating selected nitroso compounds with an aqueous base. This study was performed as described previously^16^ and indicated the formation of the free carboxylic acid under these conditions. Consequently, treatment of the crude reaction products with aqueous solutions of Na_2_CO_3_ rendered the anticipated benzoic acid species bearing an unusual diazine moiety in place of the nitroso group. As shown in Scheme 4, the C=N bond tends to tautomerize in some cases to increase the extent of conjugation (i.e., **5a**, **5b**, and **5e**), which was verified via X-ray crystallography.^21^ Despite the modest yields of this rearrangement sequence consisting of photochemical and base-mediated reactions, the resulting products and their rapid accessibility via the continuous flow approach are of general synthetic interest.

**Scheme 5:**
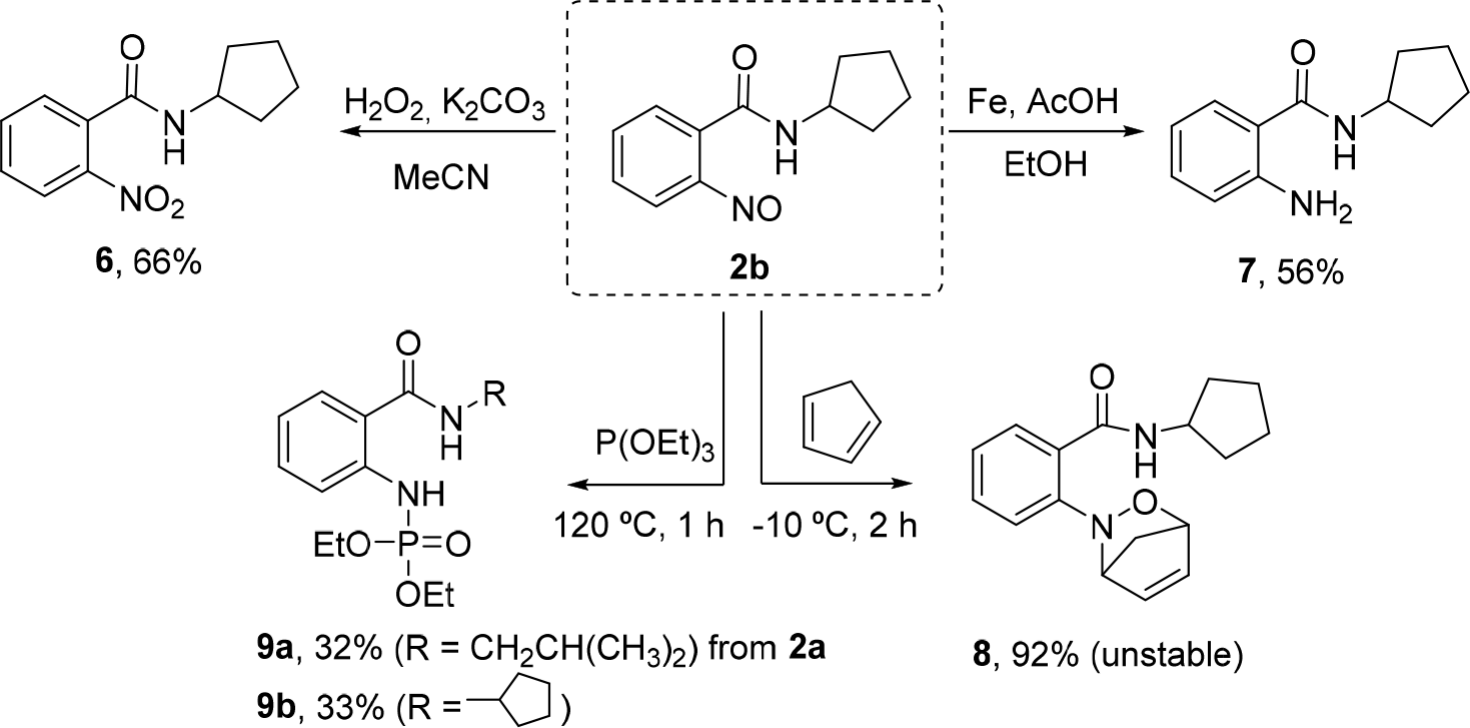
Further Functionalization Reactions of the Nitroso Moiety

Finally, to demonstrate the synthetic utility of the newly accessed nitroso species, samples of purified **2b** were subjected to different functional group interconversion reactions (Scheme 5). Good results were achieved for the oxidation (**6**, 66% yield) and reduction (**7**, 56% yield) of this group (Scheme 4). A high ^1^H NMR yield was observed in the nitroso Diels–Alder reaction with cyclopentadiene (92% yield). However, the product easily undergoes a retro-Diels–Alder process during the isolation procedure or upon gentle heating. The reaction of nitrosoarenes **2a** and **2b** with triethyl phosphite yielded phosphoramidates **9a** and **9b** in 32% and 33% isolated yields, respectively, without formation of indazole products via the expected Cadogan reaction.^22^ This type of *N*-arylphosphoramidate was also reported to form from the corresponding nitroarene, but in this case, harsher conditions would be required (i.e., microwave irradiation for 15 min at 200 °C).^23^

## Conclusions

In conclusion, we report the expedited generation of various nitrosoarenes via a photochemically triggered electron transfer process starting from *o*-nitrophenylimines. This method is facilitated by a continuous flow process exploiting a high-power LED lamp emitting photons at 365 nm. Flow photochemistry allows for a robust and effective process to access these intriguing moieties that are usually non-isolated intermediates. The solvent of choice was found to be TFE, which gave consistently superior results compared to those with other organic solvents. Gram quantities of nitroimines were processed with good functional group tolerance. X-ray crystallography was used to verify the dimeric nature of these nitroso structures in the solid state. The facile scale-up of this flow process also provided new insights into the formation of TFE-based imidates and esters under the reaction conditions. Furthermore, selected nitrosoarenes were derivatized, showing diverse synthetic applications, including the formation of benzoic acids with unusual diazine moieties. Overall, this operationally simple method for generating sets of different nitroso species and their derivatives is noteworthy for its practicality and is expected to aid in supplying chemists with these species that are otherwise difficult to access.

## Experimental Section

### General Procedure for the Synthesis of the Starting Material (*o*-nitrophenylimines) (**1a–o**)

To a solution of the corresponding amine (1 equiv) in ethanol (0.4 M) is added the corresponding aldehyde (1 equiv). The mixture is stirred for 12 h at 65 °C. Then, the solvent is evaporated in vacuo, and the corresponding product is obtained with no further purification unless otherwise specified.

### General Flow Procedure for the Synthesis of Nitrosoarenes (**2a**–**o**)

A solution of the starting material (0.3 mmol, 0.05 M) in 6 mL of degassed TFE is prepared. Once total solubility is achieved, the homogeneous solution is placed in the reaction tube inlet and the valve is switched to inject the sample. Beforehand, the flow system is stabilized by setting the light intensity, flow rate, and back pressure (3 bar) for 5 min. Upon complete injection, the vial is rinsed (1 mL of TFE), and finally, the valve is switched again to the solvent inlet with DCM. The solution is collected at the outlet of the reactor, the solvent evaporated in vacuo, and qNMR calculated using 1,3,5-trimethoxybenzene as the internal standard. Note that because of the instability of these compounds, isolation was performed directly after the reaction and total dryness of the crude mixture was avoided.

### General Procedure for the Synthesis of Benzoic Acid Derivatives (Scheme 4, **5a**–**f**)

The solvent of the corresponding crude mixture of the flow procedure mentioned above is evaporated in vacuo. The residue is redissolved in 3 mL of DCM, and 3 mL of a saturated solution of Na_2_CO_3_ is added. The mixture is stirred for 12 h. Then, phases are separated, and the organic layer is extracted twice with 5 mL of saturated Na_2_CO_3_. All of the aqueous layers are combined and acidified with HCl until the pH becomes acidic. The resulting aqueous mixture is extracted with AcOEt (thrice). Organic layers are combined and washed with brine and dried with sodium sulfate, and the solvent is evaporated in vacuo. The resulting carboxylic acid (**3a**–**f**) needs no further purification.

## Data Availability

The data underlying this study are available in the published article and its Supporting Information.

## Supporting Information Available

Optimization tables, characterization data, experimental and spectroscopic data, copies of ^1^H, ^13^C, and ^19^F NMR spectra, pictures of the flow equipment, and crystal structures of representative nitroso compounds (PDF)
