# Supplementary material for: Continuous Flow Synthesis of Nitrosoarenes via Photochemical Rearrangement of Aryl Imines
Source: J Org Chem. 2023 Dec 22;89(1):617–23. doi: 10.1021/acs.joc.3c02362 (PMC10777388; doi:10.1021/acs.joc.3c02362)

Continuous Flow Synthesis of Nitrosoarenes via Photochemical Rearrangement of Aryl Imines

Jorge García-Lacuna,* Marcus Baumann*

University College Dublin, School of Chemistry, Science Centre South, Belfield, Dublin 4, Ireland

Email: jorge.garcialacuna@ucd.ie and marcus.baumann@ucd.ie

Table of contents

General materials and methods..........S3

SI tables..........S4

Characterization data of new compounds..........S6

Characterization data of nitrosoarenes: **2a-2o**..........S6

Characterization data of compounds: **5a-5f**..........S13

Characterization data of nitrosoarenes functionalization products: **6-9**..........S15

Characterization data of starting materials (o-nitrophenylimines): **1a-1o**..........S18

X-Ray data..........S24

References..........S44

Pictures of the flow equipment and a nitroso in solution/solid state..........S45

Copies of NMR data..........S47

NMR copies of nitrosoarenes: **2a-2o**..........S47

NMR copies of side products **3** and **4**..........S65

NMR copies of compounds: **5a-f**..........S69

NMR copies of nitrosoarenes functionalization products: **6-9**..........S75

NMR copies of starting materials (*o*-nitrophenylimines): **1a-1o**..........S85

General materials and methods

Unless otherwise stated, all solvents were purchased from Fisher Scientific and used without further purification. Also, unless otherwise stated, all substrates and reagents were purchased from Fluorochem or Sigma-Aldrich and used as received. ^{1}H NMR spectra were recorded on 400, 500 and 600 MHz instruments and are reported relative to the residual solvent: $CHCl_3$ (δ 7.26 ppm) or DMSO-d6 (δ 2.50 ppm). $^{13}C\{^{1}H\}$ NMR spectra were recorded on the same instruments (100 and 125 MHz) and are reported relative to $CHCl_3$ (δ 77.0 ppm) or DMSO-d6 (δ 39.52 ppm). ^{19}F NMR were recorded at 376 MHz.

Data for 1H NMR are reported as follows: chemical shift (δ/ ppm) (integration, multiplicity, coupling constant (Hz)). Multiplicities are reported as follows: s = singlet, d = doublet, t = triplet, q = quartet, p = pentet, m = multiplet, br s = broad singlet, app = apparent. Data for $^{13}C\{^{1}H\}$ NMR are reported in terms of chemical shift (δ/ppm) and multiplicity (C, CH, CH_2, or CH_3). COSY and HSQC experiments were used in the structural assignment.

High-resolution mass spectrometry was performed using the indicated techniques on a micromass LCT orthogonal time-of-flight mass spectrometer with leucine-enkephalin (Tyr-GlyPhe-Leu) as an internal lock mass. For UV/Vis measurements, a Shimadzu UV-1800 UV spectrophotometer was used. Melting points were recorded with a Stuart SMP10 melting point apparatus and are uncorrected. IR spectra were obtained by use of a Bruker Platinum spectrometer (neat, ATR sampling) with the intensities of the characteristic signals being reported as weak (w, 71% of the tallest signal). signal), medium (m, 21–70% of the tallest signal), or strong (s, >71% of the tallest signal).

Continuous flow experiments were performed on a Vapourtec E-series system equipped with peristaltic pumps and a dynamic BPR achieved through utilization of a peristaltic pump in a reverse direction (1-9 bar, Vapourtec). For photochemical experiments the UV-150 module (Vapourtec) was used in combination with a high-power LED (365 nm, purchased from Vapourtec) regulated between 33-100 W and cooled to 25-30 °C by passing a stream of compressed air through the reactor unit. No filters were used. Reactor coils were made of PFA (Perfluoroalkoxy polymer) tubing (i.d. 1/16 inch) with a volume of 10 mL.

Batch heated reactions were performed using DrySyn heating blocks.

TLC was performed on Merck pre-coated Silica gel 60 F254 aluminium plates with realisation by UV irradiation at 254 nm, KMnO4 and vanillin stain. Flash chromatography was performed using Macherey-Nagel silica gel 60 M, with a particle range of 0.04 - 0.063 mm.

SI tables

Optimization tables

- Table S1: Solvent screening

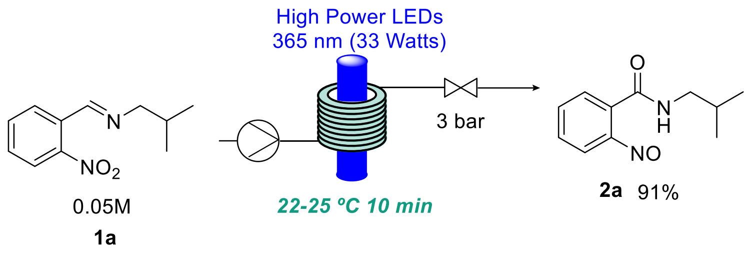

Entry[a]	Solvent	NMR yield[b]
1	MeCN	53%
2	DCM	64%
3	HFIP[c]	80%
4	Trifluoroethanol	91%[d]
5	EtOH	63%
6	Toluene	44%
7	MeCN/H_2O (2:1)	29%
8	DCM/IPA (4:1)	56%
9	DCM/TFE (4:1)	71%
10	DCM/TFE (1:1):	65%

[a] All reactions were performed at 0.3 mmol scale, in 0.05 M in the corresponding degassed solvent at 22-25 ºC with a residence time of 10 minutes (flow rate: 1 mL/min), using high power 365 nm LEDs with input power of 33 Watts, and a system pressure of 3 bar. [b] qNMR yield calculated using 1,3,5-trimethoxybenzene as internal standard, starting material remaining in brackets. [c] 1,1,1,3,3,3-Hexafluoro-2-propanol. [d] Average of 3 different runs, carried out on different days. Best conditions are highlighted.

- Table S2: Residence time screening

Entry[a]	Solvent	Res. time (min)	NMR yield[b]
1	MeCN	10	53%
2	MeCN	20	20%
3	MeCN	5	31% (18%)
4	Trifluoroethanol	10	91%
5	Trifluoroethanol	20	59%
6	Trifluoroethanol	5	51 (12%)

[a] All reactions were performed at 0.3 mmol scale, in 0.05 M in the corresponding degassed solvent at 22-25 ºC with the corresponding flow rate to achieve the desired residence time (10 mL coil reactor), using high power 365 nm LEDs with input power of 33 Watts, and a system pressure of 3 bar. [b] qNMR yield calculated using 1,3,5-trimethoxybenzene as internal standard, starting material remaining in brackets. Best conditions are highlighted.

- Table S3: Wavelength and light intensity screening

Entry[a]	Light source	NMR yield[b]
1	365 nm (set at 33 W)	91%
2	365 nm (set at 66 W)	60%
3	400 nm	69% (5%)
4	420 nm	30% (19%)
5	No light	-

[a] All reactions were performed at 0.3 mmol scale, in 0.05 M in the trifluoroethanol at 22-25 ºC ºC with a residence time of 10 minutes (flow rate: 1 mL/min), using the corresponding light source a system pressure of 3 bar [b] qNMR yield calculated using 1,3,5-trimethoxybenzene as internal standard, starting material remaining in brackets. Best conditions are highlighted.

Characterization data of new compounds

Characterization data of nitrosoarenes: 2a-2o

General flow procedure for nitrosoarenes synthesis: a solution of the starting material (0.3 mmol, 0.05 M) in 6 mL of degassed triflouroethanol is prepared. Once total solubility is achieved, the homogenous solution is placed in the reaction tube inlet and the valve is switched to inject the sample. Beforehand, the flow system is stabilized by setting the light intensity, flow rate, and back pressure (3 bar) for 5 minutes. Upon complete injection, the vial is rinsed (1 mL of trifluoroethanol) and finally, the valve is switched again to the solvent inlet with DCM. The solution is collected at the outlet of the reactor, the solvent is evaporated in vacuo and qNMR is calculated using 1,3,5-trimethoxybenzene as internal standard. Note that because of the instability of these compounds, isolation was performed straight after the reaction and total dryness of the crude mixture was avoided.

2a: *N*-isobutyl-2-nitrosobenzamide

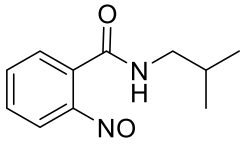

Following the general procedure and using 250 mg (1.21 mmol) of **1a** (*E*)-*N*-isobutyl-1-(2-nitrophenyl)methanimine) as starting material, 173 mg were isolated (0.84 mmol, 69% isolated yield) using column chromatography (Pentane: AcOEt, 4:1) as a white solid, which quickly starts to decompose forming a yellow oil. NMR yield using optimized conditions: 91%. **^{1}H NMR (500 MHz, $CDCl_3$)** δ 8.47 (dd, *J* = 7.8, 1.3 Hz, 1H, ArH), 8.41 (bs, 1H, NH), 7.88 – 7.78 (m, 1H, ArH), 7.46 (ddd, *J* = 8.5, 7.2, 1.2 Hz, 1H, ArH), 6.23 (dd, *J* = 8.0, 1.3 Hz, 1H, ArH), 3.46 (dd, *J* = 6.8, 5.8 Hz, 2H, CH_2), 1.99 – 1.91 (m, 1H, CH), 0.96 (d, J = 6.7 Hz, 6H, 2xCH_3). **^{13}C{^{1}H} NMR (101 MHz, $CDCl_3$)** δ 165.5 (CO), 162.3 (C), 136.7 (CH), 135.2 (CH), 132.1 (CH), 130.7 (CH), 105.7 (C), 48.0 (CH_2), 28.4 (CH), 20.2 (2xCH_3). **HRMS** (TOF-ESI+) m/z: $[M+H]^+$ Calcd for $C_{11}H_{15}N_2O_2$ 207.1133, found 207.1130.

2b: *N*-cyclopentyl-2-nitrosobenzamide

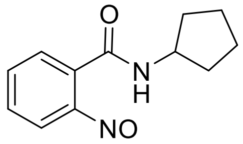

Following the general procedure using (*E*)-*N*-cyclopentyl-1-(2-nitrophenyl)methanimine (**1b**) as starting material: NMR yield: 75%, purification using column chromatography (Pentane:AcOEt; 4:1). 48 mg (0.22 mmol, 74% yield) at 0.3 mmol scale; 761 mg were obtained (3.48 mmol, isolated yield 76%, throughput of 415 mgh^{-1}, space time yield: of 190 $mmolL^{-1}h^{-1}$) at 4.58 mmol scale (1 g, 110 minutes of total time). Off white solid with a green intense color in solution (See discussion section and pictures at the end of SI). **M. p.**: (148 – 149 ºC). **^{1}H NMR (400 MHz, $CDCl_3$)** δ 8.45 (dd, *J* = 7.9, 1.4 Hz, 1H, ArH), 8.31 (bs, 1H, NH), 7.88 – 7.80

(m, 1H, ArH), 7.51 – 7.39 (m, 1H, ArH), 6.24 (dd, J = 8.1, 1.3 Hz, 1H, ArH), 4.60 (q, J = 6.7 Hz, 1H, CH), 2.15 – 2.04 (m, 2H, CH_2), 1.69 – 1.63 (m, , 4H, 2xCH_2), 1.59 – 1.51 (m, 2H, CH_2). **$^{13}C\{^1H\}$ NMR (101 MHz, $CDCl_3$)** δ 165.0 (CO), 162.4 (C), 136.8 (CH), 135.1 (C), 132.0 (CH), 130.7 (CH), 105.9 (CH), 52.4 (CH), 33.1 (2xCH_2), 23.8 (2xCH_2). **HRMS** (TOF-ESI+) m/z: $[M+H]^+$ Calcd for $C_{12}H_{15}N_2O_2$ 219.1129, found 219.1128. **IR** (neat) v/cm^{-1}: 3221 (m), 3064 (w), 2958 (m), 2839 (w), 1665 (s), 1629 (m), 1553 (m), 1448 (m), 1413 (m), 1158 (m), 878 (s).

2c*:* *N*-cyclohexyl-2-nitrosobenzamide

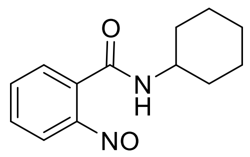

Following the general procedure using (*E*)-*N*-cyclohexyl-1-(2-nitrophenyl)methanimine (**1c**) as starting material: NMR yield: 86%, a small sample could be isolated using column chromatography (Cyclohexane:AcOEt; 4:1) for full characterization. Pale yellow solid, **M. p.**: 110 – 112 ºC. **^1H NMR (500 MHz, $CDCl_3$)** δ 8.44 (dd, J = 7.8, 1.4 Hz, 1H, ArH), 8.20 (bs, 1H, NH), 7.83 (ddd, J = 7.8, 7.2, 1.3 Hz, 1H, ArH), 7.45 (ddd, J = 8.1, 7.2, 1.4 Hz, 1H, ArH), 6.25 (dd, J = 8.1, 1.3 Hz, 1H, ArH), 4.27 – 4.14 (m, 1H, CH), 2.10 – 2.03 (m, 2H, CH_2), 1.74 – 1.20 (m, 8H, 4xCH_2). **$^{13}C\{^1H\}$ NMR (126 MHz, $CDCl_3$)** δ 164.5 (CO), 162.3 (C), 136.7 (CH), 135.3 (C), 132.0 (CH), 130.6 (CH), 106.1 (C), 49.4 (CH), 32.9 (2xCH_2), 25.6 (2xCH_2), 24.7 (CH_2). **HRMS** (TOF-ESI+) m/z: $[M+H]^+$ Calcd for $C_{13}H_{17}N_2O_2$ 233.1285, found 233.1286.

2d*:* *N*-(tert-butyl)-2-nitrosobenzamide

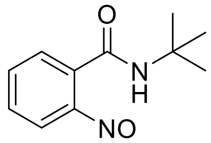

Following the general procedure using 200 mg (0.97 mmol) of (*E*)-*N*-tert-butyl-1-(2-nitrophenyl)methanimine (**1d**) as starting material, 112 mg (0.54 mmol) were obtained (56% isolated yield, NMR yield: 79%) as light brown crystals after column chromatography purification (Cyclohexane:AcOEt, from 9:1 to 3:1). Pale yellow crystals suitable for X-ray crystallography were formed by slow evaporation of a sample solution in DCM. **M. p**. 113 – 114 ºC. **^1H NMR (400 MHz, $CDCl_3$)** δ 8.38 (ddd, J = 8.0, 1.4, 0.5 Hz, 1H, Ar), 8.14 (bs, 1H, NH), 7.82 (ddd, J = 7.8, 7.2, 1.3 Hz, 1H, ArH), 7.44 (ddd, J = 8.1, 7.2, 1.4 Hz, 1H, ArH), 6.27 (ddd, J = 8.1, 1.4, 0.5 Hz, 1H, ArH), 1.52 (s, 9H, 3xCH_3). **$^{13}C\{^1H\}$ NMR (101 MHz, $CDCl_3$)** δ 164.6 (CO), 162.3 (C), 136.7 (CH), 136.0 (C), 131.6 (CH), 130.4 (CH), 106.3 (CH), 52.3 (C), 28.9 (3xCH_3). **HRMS** (TOF-ESI+) m/z: $[M+H]^+$ Calcd for $C_{11}H_{15}N_2O_2$ 207.1128, found 207.1129. **IR** (neat) v/cm^{-1}: 3224 (w), 3274 (m), 3077 (w), 3026 (w), 2972 (m), 1662 (m), 1634 (s), 1605 (w), 1550 (m), 1529 (m), 1489 (m), 1451 (m), 1402 (m), 1361 (m), 1287 (m), 1195 (m), 951 (m), 761 (s) 683 (s).

2e: *N*-decyl-2-nitrosobenzamide

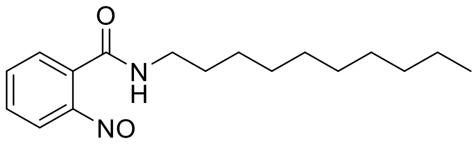

Following the general procedure using (*E*)-*N*-decyl-1-(2-nitrophenyl)methanimine (**1h**) as starting material: Isolation using column chromatography (Pentane:AcOEt; 2:1) to obtain 56 mg (0.19 mmol, 64% yield, NMR yield 64%), as a pale orange solid. **M. p.**: 105 – 106 ºC. **^{1}H NMR (400 MHz, $CDCl_3$)** δ 8.46 (dd, *J* = 7.9, 1.5 Hz, 1H, ArH), 8.35 (bs, 1H, NH), 7.88 – 7.80 (m, 1H, ArH), 7.45 (ddd, *J* = 8.4, 7.2, 1.4 Hz, 1H, ArH), 6.23 (dd, *J* = 8.1, 1.3 Hz, 1H, ArH), 3.60 (td, *J* = 7.2, 5.6 Hz, 2H, CH_2N), 1.68 – 1.60 (m, 2H, CH_2), 1.36 – 1.22 (m, 14H, 7xCH_2), 0.90 – 0.84 (m, 3H, CH_3). **^{13}C{^{1}H} NMR (101 MHz, $CDCl_3$)** δ 165.4 (C), 162.3 (C), 136.8 (CH), 135.2 (C), 132.1 (CH), 130.7 (CH), 105.8 (CH), 40.8 (CH_2), 31.9 (CH_2), 29.5 (2xCH_2), 29.4 (CH_2), 29.25 (CH_2), 29.23 (CH_2), 27.0 (CH_2), 22.6 (CH_2), 14.1 (CH_3). **HRMS** (TOF-ESI+) m/z: $[M+H]^+$ Calcd for $C_{17}H_{27}N_2O_2$ 291.2067, found 291.2071.

2f: *N*-(2-methoxybenzyl)-2-nitrosobenzamide

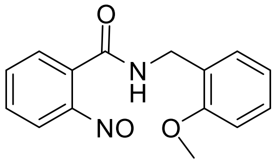

Following the general procedure using (*E*)-*N*-(2-methoxybenzyl)-1-(2-nitrophenyl)methanimine (**1f**) as starting material: NMR yield: 75%, a small sample could be isolated using column chromatography (Cyclohexane:AcOEt; 3:1) for full characterization, as white solid. **M. p.** 134 – 136 ºC. **^{1}H NMR (400 MHz, $CDCl_3$)** δ 8.95 (bs, 1H, NH), 8.50 (ddd, *J* = 7.8, 1.4, 0.5 Hz, 1H, ArH), 7.88 – 7.78 (m, 1H, ArH), 7.44 (ddd, *J* = 8.1, 7.3, 1.4 Hz, 1H, ArH), 7.38 (dd, *J* = 7.4, 1.8 Hz, 1H, ArH), 7.29 – 7.20 (m, 1H, ArH), 6.92 (td, *J* = 7.4, 1.1 Hz, 1H, ArH), 6.85 (dd, *J* = 8.2, 1.0 Hz, 1H, ArH), 6.20 (ddd, *J* = 8.2, 1.3, 0.5 Hz, 1H, ArH), 4.81 (d, *J* = 5.8 Hz, 2H, CH_2), 3.78 (s, 3H, CH_3). **^{13}C{^{1}H} NMR (101 MHz, $CDCl_3$)** δ 165.1 (CO), 162.2 (C), 157.6 (C), 136.7 (CH), 135.1 (C), 132.2 (CH), 130.8 (CH), 129.7 (CH), 128.9 (CH), 126.0 (C), 120.6 (CH), 110.3 (CH), 105.5 (CH), 55.2 (CH_2), 40.7 (CH_3). **HRMS** (TOF-ESI+) m/z: $[M+H]^+$ Calcd for $C_{15}H_{14}N_2O_3$ 271.1077, found 271.1078.

2g: *N*-(4-fluorobenzyl)-2-nitrosobenzamide

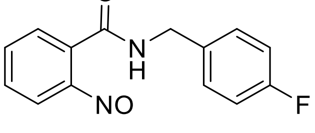

Following the general procedure using (*E*)-*N*-(4-fluorobenzyl)-1-(2-nitrophenyl)methanimine (**1e**) as starting material: NMR yield: 67%, a small sample could be isolated using column chromatography

(Cyclohexane:AcOEt; 3:1) for full characterization, as white solid. **M. p.**: 138 – 139 °C. **^{1}H NMR (400 MHz, CDCl$_3$)** δ 8.74 (bs, 1H, NH), 8.57 – 8.47 (m, 1H, ArH), 7.87 (ddd, *J* = 7.8, 7.2, 1.3 Hz, 1H, ArH), 7.49 (ddd, *J* = 8.0, 7.3, 1.4 Hz, 1H, ArH), 7.38 – 7.30 (m, 2H, ArH), 7.01 (t, *J* = 8.7 Hz, 2H, ArH), 6.25 (ddd, *J* = 8.2, 1.3, 0.5 Hz, 1H, ArH), 4.79 (d, *J* = 5.7 Hz, 2H, CH$_2$). **^{13}C{^{1}H} NMR (101 MHz, CDCl$_3$)** δ 165.5 (CO), 162.18 (d, *J* = 245.6 Hz, C), 162.17 (C), 136.9 (CH), 134.7 (C), 133.8 (d, *J* = 3.2 Hz, C), 132.2 (CH), 131.1 (CH), 129.4 (d, *J* = 8.2 Hz, 2xCH), 115.56 (d, *J* = 21.3 Hz, 2xCH), 105.9 (CH), 43.9 (CH$_2$). **^{19}F NMR (376 Hz, CDCl$_3$)** δ -112.03 – -121.09 (m). **HRMS** (TOF-ESI+) m/z: [M+H]$^+$ Calcd for $C_{14}H_{12}FN_2O_2$ 259.0877, found 259.0878.

2h methyl (2-nitrosobenzoyl)phenylalaninate

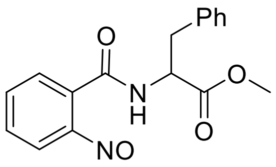

Following the general procedure using (methyl (*E*)-2-((2-nitrobenzylidene)amino)-3-phenylpropanoate (**1g**) as starting material: NMR yield: 71%, a small sample could be isolated using column chromatography (Pentane:AcOEt; 2:1) for full characterization, as a pale yellow oil. **^{1}H NMR (400 MHz, CDCl$_3$)** δ 9.16 (d, *J* = 8.1 Hz, 1H, NH), 8.42 (ddd, *J* = 7.8, 1.4, 0.5 Hz, 1H, ArH), 7.87 – 7.79 (m, 1H, ArH), 7.53 – 7.46 (m, 1H, ArH), 7.41 – 7.30 (m, 4H, ArH), 7.29 – 7.24 (m, 1H, ArH), 6.29 (ddd, *J* = 8.1, 1.4, 0.5 Hz, 1H, ArH), 5.84 (dt, *J* = 8.2, 6.2 Hz, 1H, CH), 3.59 (s, 3H, CH$_3$), 3.01 (qd, *J* = 15.6, 6.2 Hz, 2H, CH$_2$). **^{13}C{^{1}H} NMR (101 MHz, CDCl$_3$)** δ 171.2 (CO), 164.9 (CO), 162.2 (C), 140.4 (C), 136.7 (CH), 134.78 (C), 132.0 (CH), 131.0 (CH), 128.8 (2xCH), 127.7 (CH), 126.3 (2xCH), 106.1 (CH), 51.8 (CH), 51.1 (CH$_3$), 40.4 (CH$_2$). **HRMS** (TOF-ESI+) m/z: [M+H]$^+$ Calcd for $C_{17}H_{17}N_2O_4$ 313.1183, found 313.1184.

2i: 2-nitroso-*N*-phenylbenzamide

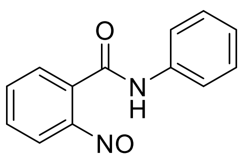

Following the general procedure using (*E*)-1-(2-nitrophenyl)-N-phenylmethanimine (**1i**) as starting material: NMR yield: 66%, isolation by crystallization using a mixture of DCM/MeOH/Pentane. (37 mg, 0.16 mmol, 53% isolated yield) as pale brown solid. **M. p.:** 160 – 162 °C. **^{1}H NMR (400 MHz, CDCl$_3$)** δ 10.56 (bs, 1H, NH), 8.62 (dd, *J* = 7.8, 1.4 Hz, 1H, ArH), 7.98 – 7.87 (m, 1H, ArH), 7.73 – 7.68 (m, 2H, ArH), 7.54 (ddd, *J* = 8.4, 7.2, 1.4 Hz, 1H, ArH), 7.39 (dd, *J* = 8.5, 7.4 Hz, 2H, ArH), 7.21 – 7.14 (m, 1H, ArH), 6.31 (dd, *J* = 8.1, 1.3 Hz, 1H, ArH). **^{13}C{^{1}H} NMR (101 MHz, CDCl$_3$)** δ 163.2 (CO), 162.1 (C), 138.2 (C), 137.2 (CH), 134.8 (C), 132.5 (CH), 131.4 (CH), 129.1 (2xCH), 124.9 (CH), 120.7 (2xCH), 106.0. **HRMS** (TOF-ESI+) m/z: [M+H]$^+$ Calcd for $C_{13}H_{11}N_2O_2$ 227.0815, found 227.0816.

2j: *N*-(4-fluorophenyl)-2-nitrosobenzamide

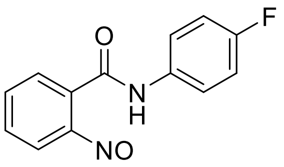

Following the general procedure using (*E)-N*-(4-fluorophenyl)-1-(2-nitrophenyl)methanimine (**1j**) as starting material: NMR yield: 62%, 39 mg (0.162 mmol, 54% yield) were isolated by crystallization using a mixture of DCM/MeOH/Pentane. Off-white solid (**M. p.** 169 – 171 ºC). **^{1}H NMR (400 MHz, DMSO-*d*$_6$)** δ 10.88 (bs, 1H, NH), 8.00 – 7.94 (m, 2H, ArH), 7.79 – 7.75 (m, 2H, ArH), 7.69 (ddd, *J* = 8.1, 6.1, 2.6 Hz, 1H, ArH), 7.22 (t, *J* = 8.9 Hz, 2H, ArH), 6.87 – 6.81 (m, 1H, ArH). **^{13}C{^{1}H} NMR (101 MHz, DMSO-*d*$_6$)** δ 165.5 (CO), 161.9 (C), 158.33 (d, *J* = 240.5 Hz, C), 138.2 (C), 136.7 (CH), 135.4 (d, *J* = 2.6 Hz, C), 130.3 (CH), 129.3 (CH), 121.30 (d, *J* = 7.9 Hz, 2xCH), 115.46 (d, *J* = 22.2 Hz, 2xCH), 109.9 (CH). **^{19}F NMR (376 MHz, DMSO-*d*$_6$)** δ -118.36 (tt, *J* = 8.7, 5.1 Hz). **HRMS** (TOF-ESI+) m/z: $[M+H]^+$ Calcd for $C_{13}H_{10}FN_2O_2$ 245.0721, found 245.0722.

2k: *N*-(4-isopropylphenyl)-2-nitrosobenzamide

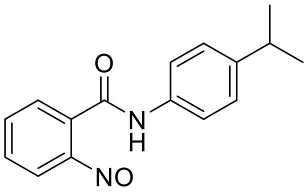

Following the general procedure using (*E*)-*N*-(4-isopropylphenyl)-1-(2-nitrophenyl)methanimine (**1k**) as starting material: NMR yield: 65%, isolation by crystallization using a mixture of DCM/MeOH/Pentane. (76 mg, 0.17 mmol, 57% isolated yield) as an off white solid. **M. p.:** 157 – 159 ºC. **^{1}H NMR (500 MHz, CDCl$_3$)** δ 10.46 (bs, 1H, NH), 8.60 (dd, *J* = 7.9, 1.4 Hz, 1H, ArH), 7.91 (ddd, *J* = 7.9, 7.2, 1.3 Hz, 1H, ArH), 7.66 – 7.58 (m, 2H, ArH), 7.52 (ddd, *J* = 8.0, 7.2, 1.4 Hz, 1H, ArH), 7.24 (d, *J* = 8.4 Hz, 2H, ArH), 6.30 (dd, *J* = 8.1, 1.3 Hz, 1H, ArH), 2.91 (p, *J* = 6.9 Hz, 1H, CH), 1.26 (s, 3H, CH_3), 1.25 (s, 3H, CH_3). **^{13}C{^{1}H} NMR (101 MHz, CDCl$_3$)** δ 163.1 (CO), 162.1 (C), 145.7 (C), 137.2 (CH), 135.8 (C), 134.9 (C), 132.5 (CH), 131.3 (CH), 127.0 (2xCH), 120.8 (2xCH), 105.9 (C), 33.7 (CH), 24.0 (2xCH_3). **HRMS** (TOF-ESI+) m/z: $[M+H]^+$ Calcd for $C_{16}H_{17}N_2O_2$ 269.1285, found 269.1287.

2l: *N*-cyclopentyl-5-fluoro-2-nitrosobenzamide

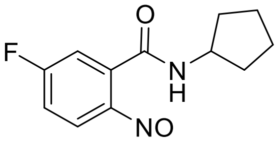

Following the general procedure using (*E*)-*N*-cyclopentyl-1-(5-fluoro-2-nitrophenyl)methanimine (**1l**) as starting material: NMR yield: 71%, isolation using column chromatography (Cyclohexane:AcOEt; 6:1) to obtain 51 mg (0.22 mmol, 72% yield), as a pale brown solid. **M. p.**:103 – 105 ºC. **^{1}H NMR (400 MHz, CDCl$_3$)** δ 8.52 (bs, 1H, NH), 8.16 (dd, *J* = 9.4, 2.8 Hz, 1H, ArH), 7.10 (ddd, *J* = 9.0, 7.1, 2.8 Hz, 1H, ArH), 6.35 (dd, *J* = 8.9, 5.2 Hz, 1H, ArH), 4.63 – 4.53 (m, 1H, CH), 2.16 – 2.05 (m, 2H, CH_2), 1.71 – 1.64 (m, 4H, 2xCH_2), 1.59 – 1.49 (m, 2H, CH_2). **^{13}C{^{1}H} NMR (101 MHz, CDCl$_3$)** δ 167.6 (d, *J* = 262.9 Hz, C), 163.4 (d, *J* = 1.6 Hz, CO),

160.3 (d, J = 3.0 Hz, C), 138.6 (d, J = 9.1 Hz, C), 118.9 (d, J = 25.2 Hz, CH), 117.9 (d, J = 23.7 Hz, CH), 109.7 (d, J = 10.1 Hz), 52.6 (CH), 33.1 (2xCH$_2$), 23.8 (2xCH$_2$). **^{19}F NMR (376 MHz, CDCl$_3$)** δ -96.28 (ddd, J = 9.4, 7.1, 5.2 Hz). **HRMS** (TOF-ESI+) m/z: [M+H]$^+$ Calcd for $C_{12}H_{14}FN_2O_2$ 237.1034, found 237.1034.

2m: *N*-cyclopentyl-6-nitrosobenzo[*d*][1,3]dioxole-5-carboxamide

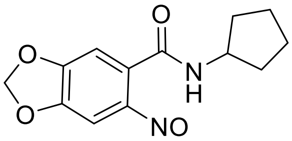

Following the general procedure using (*E*)-*N*-cyclopentyl-1-(6-nitrobenzo[*d*][1,3]dioxol-5-yl)methanimine (**1m**) as starting material: NMR yield: 69%, isolation using column chromatography (Cyclohexane:AcOEt; 4:1 to 1:1) to obtain 53 mg (0.20 mmol, 67% isolated yield) as a green solid. Light green crystals suitable for X-ray crystallography were formed by slow evaporation of a sample solution in DCM. **M. p.:** 149 – 151 ºC **^{1}H NMR (400 MHz, CDCl$_3$)** δ 8.55 (s, 1H, bs), 7.91 (s, 1H, ArH), 6.12 (s, 2H, CH$_2$), 5.88 (s, 1H, ArH), 4.59 – 4.48 (m, 1H, CH), 2.13 – 2.03 (m, 2H, CH$_2$), 1.70 – 1.61 (m, 4H, 2xCH$_2$), 1.56 – 1.48 (m, 2H, CH$_2$). **^{13}C{^{1}H} NMR (101 MHz, CDCl$_3$)** δ 163.9 (C), 161.7 (CO), 154.8 (C), 150.5 (C), 135.8 (C), 110.6 (CH), 103.2 (CH$_2$), 87.2 (CH), 52.5 (CH), 33.1 (2xCH$_2$), 23.8 (2xCH$_2$). **HRMS** (TOF-ESI+) m/z: [M+H]$^+$ Calcd for $C_{13}H_{15}N_2O_4$ 263.1026, found 263.1031. **IR** (neat) v/cm^{-1}: 3287.6 (w), 3083.9 (w), 2948.7 (w), 2867.9 (w), 1629.8 (w), 1546.7 (w), 1469.0 (m), 1415.6 (w), 1233.1 (s), 118.5 (m), 981.6 (m), 922.1 (m), 866.1 (m), 529.3 (m).

2n: methyl 4-(*tert*-butylcarbamoyl)-3-nitrosobenzoate

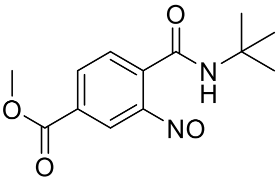

Following the general procedure using methyl (*E*)-4-((*tert*-butylimino)methyl)-3-nitrobenzoate (**1m**) as starting material: NMR yield: 65%, isolation using column chromatography (Cyclohexane:AcOEt; 6:1) to obtain 36 mg (0.14 mmol, 46% isolated yield), as a pale brown solid. **M. p**.: 170 – 172 ºC. **^{1}H NMR (400 MHz, CDCl$_3$)** δ 8.47 – 8.38 (m, 2H, ArH), 8.04 (bs, 1H, NH), 6.91 (dd, J = 1.6, 0.6 Hz, 1H, ArH), 3.94 (s, 3H, CH$_3$O), 1.52 (s, 9H, 3xCH$_3$). **^{13}C{^{1}H} NMR (101 MHz, CDCl$_3$)** δ 165.1 (C), 163.8 (C), 161.4 (C), 139.1 (C), 136.1 (CH), 132.2 (C), 132.0 (CH), 108.1 (CH), 52.8 (CH$_3$), 52.7 (C), 28.8 (3xCH$_3$). **HRMS** (TOF-ESI+) m/z: [M+H]$^+$ Calcd for $C_{13}H_{17}N_2O_4$ 265.1183, found 265.1186.

2o: *N*-(*tert*-butyl)-3-methyl-2-nitrosobenzamide

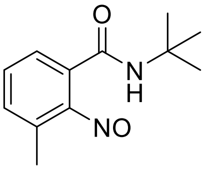

Following the general procedure using (*E*)-*N*-*tert*-butyl-1-(3-methyl-2-nitrophenyl)methanimine (**1o**) as starting material: NMR yield: 49%, full characterization was accomplished from a small crude sample, obtained by white solid precipitation of the crude in DCM/pentane. As it quickly starts to decompose in both solution, even during C-NMR sample acquisition, and solid state. **^{1}H NMR (500 MHz, $CDCl_3$)** δ 7.56 (t, *J* = 7.5 Hz, 1H, ArH), 7.47 (ddd, *J* = 7.6, 1.4, 0.7 Hz, 1H, ArH), 7.35 (ddd, *J* = 7.4, 1.4, 0.6 Hz, 1H, ArH), 5.55 (bs, 1H, NH), 2.93 (s, 3H, CH_3), 1.53 (s, 9H, 3xCH_3). **^{13}C{^{1}H} NMR (126 MHz, $CDCl_3$)** 167.3 (C), 162.1 (C), 136.1 (C), 135.2 (CH), 133.8 (CH), 126.5 (C), 126.3 (CH), 52.1 (C), 28.7 (3xCH_3), 18.6 (CH_3). **HRMS** (TOF-ESI+) m/z: $[M+H]^+$ Calcd for $C_{12}H_{17}N_2O_2$ 221.1285, found 221.1286.

3: 2,2,2-trifluoroethyl *(Z)*-*N*-cyclopentyl-2-nitrosobenzimidate

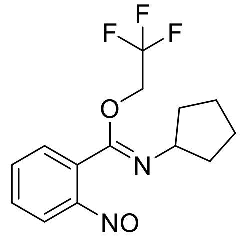

68 mg (0.23 mmol, 5% yield) of the title compound were isolated in the purification of the 1 g long-run of **1b** as a side product. Yellow oil. **^{1}H NMR (400 MHz, $CDCl_3$)** δ 7.86 (dd, *J* = 7.7, 1.5 Hz, 1H, ArH), 7.59 (tdd, *J* = 8.0, 1.5, 0.6 Hz, 1H, ArH), 7.49 – 7.41 (m, 1H, ArH), 7.23 (dd, *J* = 8.1, 1.1 Hz, 1H, ArH), 4.66 (q, *J* = 8.5 Hz, 2H, CH_2CF_3), 4.25 (tt, *J* = 7.3, 5.3 Hz, 1H, CHN), 2.10 – 1.96 (m, 4H, 2xCH_2), 1.99 – 1.87 (m, 2H, CH_2), 1.83 – 1.71 (m, 2H, CH_2). **^{13}C{^{1}H} NMR (101 MHz, $CDCl_3$)** δ 165.1 (C), 152.9 (C), 133.3 (CH), 130.2 (CH), 128.8 (CH), 124.7 (C), 123.0 (q, *J* = 277.2 Hz), 119.3 (CH), 79.1 (CH_2), 61.0 (q, *J* = 36.8 Hz, CH_2), 31.4 (2xCH_2), 25.5 (2xCH_2). **^{19}F NMR (376 MHz, $CDCl_3$)** δ -73.47 (t, *J* = 8.4 Hz). **HRMS** (TOF-ESI+) m/z: $[M+H]^+$ Calcd for $C_{14}H_{16}F_3N_2O_2$ 301.1158, found 301.1161.

4: 2,2,2-trifluoroethyl 2-(2-cyclopentylidenehydrazineyl)benzoate

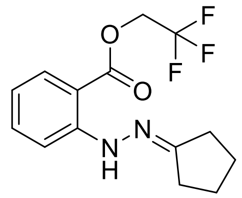

Compound X spontaneously formed the title compound in solution after 48 h. Pale brown crystals were obtained by slow evaporation of chloroform. **M. p**. (107 – 109 ºC). **^{1}H NMR (500 MHz, $CDCl_3$)** δ 10.17 (s, 1H, NH), 7.92 (dd, *J* = 8.1, 1.6 Hz, 1H, ArH), 7.64 (dd, *J* = 8.6, 1.2 Hz, 1H, ArH), 7.45 (ddd, *J* = 8.6, 7.0, 1.6 Hz, 1H, ArH), 6.73 (ddd, *J* = 8.2, 7.0, 1.2 Hz, 1H, ArH), 4.66 (q, *J* = 8.4 Hz, 2H, CH_2O), 2.52 (tt, *J* = 7.1, 1.2 Hz, 2H, CH_2), 2.38 (tt, *J* = 7.7, 1.2 Hz, 2H, CH_2), 1.97 – 1.87 (m, 2H, CH_2), 1.86 – 1.76 (m, 2H, CH_2). **^{13}C{^{1}H} NMR (126 MHz, $CDCl_3$)** δ 166.5 (C), 160.2 (C), 148.8 (C), 135.6 (CH), 131.0 (CH), 123.2 (q, *J* = 277.4 Hz, C), 116.9 (CH), 113.2 (CH), 106.7 (C, 60.3 (q, *J* = 36.7 Hz, CH_2)., 33.1 (CH_2), 27.5 (CH_2), 25.1 (CH_2), 24.9 (CH_2). **^{19}F NMR (376 MHz, $CDCl_3$)** δ -73.45 (t, *J* = 8.4 Hz). **HRMS** (TOF-ESI+) m/z: $[M+H]^+$ Calcd for $C_{14}H_{16}F_3N_2O_2$ 301.1158, found 301.1160. **IR** (neat) v/cm^{-1}: 3330 (m), 2961 (m), 2922 (w), 2876 (w), 1680 (s), 1603 (w), 1512 (m), 1501 (m), 1454 (w), 1277 (m), 1231 (m), 1155 (s), 1104 (m), 1070 (m), 964 (m), 876 (w) 747 (s), 655 (m), 526 (s).

Characterization data of compounds: 5a-5f

General procedure for benzoic acid derivatives synthesis (Scheme 3). The solvent of the corresponding crude mixture (0.3 mmol scale) of the abovementioned flow procedure is evaporated in vacuo. The residue is redissolved in 3 mL of DCM and 3 mL of a saturated solution of Na_2CO_3 are added. The mixture is stirred for 12 h. Then, phases are separated, and the organic layer is extracted twice with 5 mL of Na_2CO_3 (sat.). All the aqueous layers are combined and acidified with HCl (1M) and few drops of HCl (12 M) until acid pH. The resulting aqueous mixture is extracted with AcOEt (x3). Organic layers are combined and washed with brine, dried with sodium sulfate and solvent evaporated in vacuo. The resulting carboxylic acid (**5a-f**) is obtained with no need of further purification unless otherwise noted.

5a: (*E*)-2-(2-(2-methylpropylidene)hydrazineyl)benzoic acid

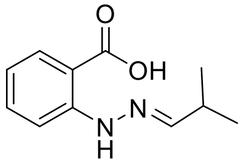

Following the general procedure B and the resulting brown solid was redissolved in AcOEt and filtered through a pad of silica. Using **1a** as starting material, 27 mg (0.13 mmol, 43% isolated yield) were obtained as a white solid. **M. p:** 158 – 161 ºC. **^{1}H NMR (400 MHz, $CDCl_3$)** δ 10.51 (bs, 1H, NH), 7.95 (dd, *J* = 8.1, 1.6 Hz, 1H, ArH), 7.67 – 7.63 (m, 1H, ArH), 7.46 (ddd, *J* = 8.8, 7.1, 1.6 Hz, 1H, ArH), 7.21 (d, *J* = 5.1 Hz, 1H, CH=), 6.78 – 6.68 (m, 1H, ArH), 2.66 – 2.55 (m, 1H, CH), 1.16 (d, *J* = 6.9 Hz, 6H, 2xCH$_3$). **^{13}C{^{1}H} NMR (101 MHz, $CDCl_3$)** δ 172.3 (CO), 150.0 (CH), 148.9 (C), 135.6 (CH), 131.7 (CH), 116.9 (CH), 113.6 (CH), 107.2 (C), 31.5 (CH), 20.1 (2xCH$_3$). **HRMS** (TOF-ESI+) m/z: $[M+H]^+$ Calcd for $C_{11}H_{15}N_2O_2$ 207.1128, found 207.1130.

5b: 2-(2-cyclopentylidenehydrazineyl)benzoic acid

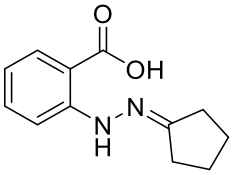

Following the general procedure B, using **1b** as starting material, 16.5 mg (0.076 mmol, 25% isolated yield) were obtained as light brown solid. **M. p**.: 175 – 177 ºC. Pale brown crystals suitable for X-ray crystallography were formed by slow evaporation of a sample solution in DCM. **^{1}H NMR (500 MHz, $CDCl_3$)** δ 10.24 (s, 1H, NH), 7.97 (dd, *J* = 8.1, 1.6 Hz, 1H, ArH), 7.64 (dd, *J* = 8.6, 1.1 Hz, 1H, ArH), 7.46 (ddd, *J* = 8.6, 7.0, 1.7 Hz, 1H, ArH), 6.73 (ddd, *J* = 8.2, 7.0, 1.2 Hz, 1H, ArH), 2.52 (tt, *J* = 7.2, 1.2 Hz, 2H, CH$_2$), 2.41 – 2.34 (m, 2H, CH$_2$), 1.96 – 1.90 (p, *J* = 7.0 Hz, 2H, CH$_2$), 1.86 – 1.76 (m, 2H, CH$_2$). **^{13}C{^{1}H} NMR (126 MHz, $CDCl_3$)** δ 173.2 (C), 160.3 (CO), 148.9 (C), 135.7 (CH), 131.8 (CH), 116.9 (CH), 113.2 (CH), 107.4 (C), 33.1

(CH_2), 27.5 (CH_2), 25.2 (CH_2), 24.9 (CH_2). **HRMS** (TOF-ESI+) m/z: $[M+H]^+$ Calcd for $C_{12}H_{15}N_2O_2$ 219.1128, found 219.1129. **IR** (neat) v/cm^{-1}: 3309 (w), 2973 (m), 2942 (w), 2915 (w), 2873 (m), 2634 (w). 2561 (w), 1657 (s), 1606 (w), 1578 (s), 1498 (s), 1403 (w), 1259 (s), 1230 (m), 746 (s).

5c: (*E*)-2-(*tert*-butyldiazenyl)benzoic acid

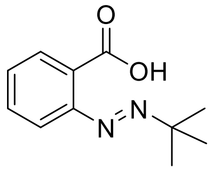

Following the general procedure B, using **1d** as starting material, 40 mg (0.195 mmol, 65% isolated yield) were obtained as pale brown solid. **M. p:** 66 – 68 ºC **^{1}H NMR (500 MHz, $CDCl_3$)** δ 8.43 – 8.37 (m, 1H, ArH), 7.81 – 7.76 (m, 1H, ArH), 7.68 – 7.62 (m, 2H, ArH), 1.43 (s, 9H, 3xCH_3). **^{13}C{^{1}H} NMR (126 MHz, $CDCl_3$)** δ 166.1 (CO), 148.4 (C), 133.7 (CH), 132.9 (CH), 132.2 (CH), 126.5 (C), 115.6 (CH), 70.2 (C), 26.8 (3xCH_3). **HRMS** (TOF-ESI+) m/z: $[M+H]^+$ Calcd for $C_{11}H_{15}N_2O_2$ 207.1128, found 207.1128.

5d: (*E*)-2-(phenyldiazenyl)benzoic acid

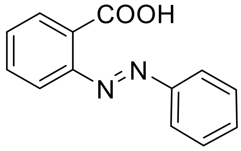

Following the general procedure B, using **1i** as starting material, 25 mg (0.111 mmol, 37% isolated yield) are obtained as pale brown solid. **M. p.:** 79 – 80 ºC. **^{1}H NMR (500 MHz, $CDCl_3$)** δ 8.48 – 8.41 (m, 1H, ArH), 8.06 – 8.01 (m, 1H, ArH), 7.91 – 7.84 (m, 2H, ArH), 7.74 – 7.66 (m, 2H, ArH), 7.65 – 7.57 (m, 3H, ArH). **^{13}C{^{1}H} NMR (126 MHz, $CDCl_3$)** δ 166.1 (CO), 151.5 (C), 149.4 (C), 133.8 (CH), 133.5 (CH), 133.1 (CH), 132.7 (CH), 129.8 (2xCH), 127.1 (C), 123.6 (2xCH), 115.8 (CH). **HRMS** (TOF-ESI+) m/z: $[M+H]^+$ Calcd for calc for $C_{13}H_{11}N_2O_2$ 227.0815, found 227.0816.

5e: 6-(2-cyclopentylidenehydrazineyl)benzo[*d*][1,3]dioxole-5-carboxylic acid

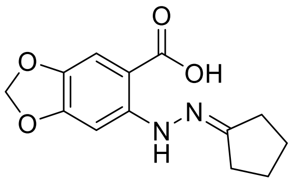

Following the general procedure B, using **1m** as starting material, 25 mg (0.096 mmol, 32% isolated yield) are obtained as pale brown solid. **M. p.** 201 – 202 ºC. **^{1}H NMR (600 MHz, DMSO-*d*$_6$)** δ 12.61 (s, 1H, COOH), 10.67 (s, 1H, NH), 7.19 (s, 1H, ArH), 6.99 (s, 1H, ArH), 5.99 (s, 2H, CH_2O), 2.41 – 2.36 (m, 2H, CH_2), 2.23 (td, J = 6.9, 1.5 Hz, 2H, CH_2), 1.83 (p, J = 7.1 Hz, 2H, CH_2), 1.71 (p, J = 6.8 Hz, 2H, CH_2). **^{13}C{^{1}H} NMR (151 MHz, DMSO-*d*$_6$)** δ 169.4 (C), 158.4 (CO), 153.0 (C), 146.7 (C), 138.6 (C), 108.3 (CH),

101.3 (CH_2), 100.3 (C), 92.8 (CH), 32.5 (CH_2), 27.3 (CH_2), 24.7 (CH_2), 24.4 (CH_2). **HRMS** (TOF-ESI+) m/z: $[M+H]^+$ Calcd for $C_{13}H_{15}N_2O_4$ 263.1031, found 263.1026.

5f: (*E*)-2-(*tert*-butyldiazenyl)-3-methylbenzoic acid

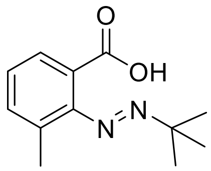

Following the general procedure B, using **1o** as starting material, 10 mg (0.045 mmol, 15% isolated yield) are obtained as pale brown solid. **M. p.:** 115 – 116 ºC. **^{1}H NMR (500 MHz, $CDCl_3$)** δ 8.03 – 7.97 (m, 1H, ArH), 7.43 (ddd, *J* = 7.6, 1.6, 0.8 Hz, 1H, ArH), 7.36 (t, *J* = 7.7 Hz, 1H, ArH), 2.33 (s, 3H, CH_3), 1.42 (s, 9H, $3xCH_3$). **$^{13}C\{^1H\}$ NMR (126 MHz, $CDCl_3$)** δ 169.2 (CO), 150.5 (C), 136.1 (CH), 130.6 (C), 130.1 (CH), 128.6 (CH), 124.1 (C), 70.2 (C), 26.8 ($3xCH_3$), 19.5 (CH_3). **HRMS** (TOF-ESI+) m/z: $[M+H]^+$ Calcd for $C_{12}H_{16}N_2O_2$ 221.1285, found 221.1285,

Characterization data of nitrosoarenes functionalization products: 6-9

6: *N*-cyclopentyl-2-nitrobenzamide

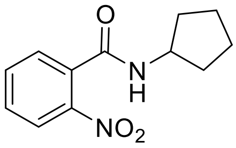

To a solution of 60 mg (0.207 mmol) of **2b** and 28 mg of K_2CO_3 (1 equiv.) in 0.8 mL of acetonitrile, H_2O_2 (1.035 mmol, 5 equiv.) are added at 0 ºC. After 1 h stirring at room temperature, the solvent is evaporated in vacuo, and residue redissolved in a mixture of H_2O and AcOEt. The aqueous layer is extracted twice with 5 mL of AcOEt. Combiend organic layers were washed with brine, dried with sodium sulfate and the solvent is evaporated in vacuo. 32 mg (0.136 mmol) were obtained as a white solid after purification using column chromatography (DCM:AcOEt 20:1). **M.p.**: 129 – 131 ºC. **^{1}H NMR (500 MHz, $CDCl_3$)** δ 8.06 (d, *J* = 1.2 Hz, 1H, ArH), 7.65 (dd, *J* = 7.5, 1.2 Hz, 1H, ArH), 7.58 – 7.53 (m, 1H, ArH), 7.51 (dd, *J* = 7.5, 1.5 Hz, 1H, ArH), 5.74 (bs, 1H, NH), 4.45 – 4.36 (m, 1H, CH), 2.13 – 2.05 (m, 2H, CH_2), 1.73 – 1.63 (m, 4H, 2x CH_2), 1.59 – 1.51 (m, 2H, CH_2). **$^{13}C\{^1H\}$ NMR (101 MHz, $CDCl_3$)** δ 166.0 (CO), 146.4 (C), 133.7 (CH), 133.3 (C), 130.3 (CH), 128.8 (CH), 124.5 (CH), 52.0 (CH), 32.8 ($2xCH_2$), 23.7 ($2xCH_2$). **HRMS** (TOF-ESI+) m/z: $[M+H]^+$ Calcd for $C_{12}H_{15}N_2O_3$ 235.1077, found 235.1079.

7: 2-amino-*N*-cyclopentylbenzamide

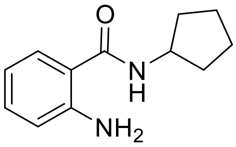

To a solution of 60 mg (0.207 mmol) of **2b** in 1 mL ethanol, 0.5 mL of H_2O, 1 mL of acetic acid, 76 mg (1.37 mmol, 5 equiv.) of Fe (powder) is added. After 1h of stirring at 30ºC in a ultrasonic bath, all starting material is consumed by TLC. The crude mixture is diluted with AcOEt (5 mL) and it is filtered through a pad of silica. The silica is washed with 10 mL of EtOAc, and the resulting organic layer is washed with $NaHCO_3$ (10 mL), brine (10 mL), dried with sodium sulfate and solvent evaporated *in vacuo*. 24 mg of **7** were obtained (56% of yield) as a white solid after purification using column chromatography (Pentane. Et_2O 4:1). **^{1}H NMR (500 MHz, $CDCl_3$)** δ 7.28 – 7.26 (m, 1H), 7.19 (ddd, *J* = 8.1, 7.2, 1.5 Hz, 1H), 6.67 (dd, *J* = 8.2, 1.1 Hz, 1H), 6.65 – 6.61 (m, 1H), 5.96 (s, 1H), 5.48 (s, 2H), 4.35 (h, *J* = 6.9 Hz, 1H), 2.11 – 2.03 (m, 2H), 1.77 – 1.62 (m, 5H), 1.53 – 1.44 (m, 2H). The reported data is in accordance with literature.[1]

8: 2-(2-oxa-3-azabicyclo[2.2.1]hept-5-en-3-yl)-*N*-cyclopentylbenzamide

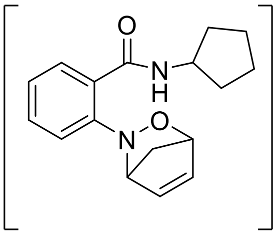

0.3 mL of freshly distilled cyclopentadiene were added to 60 mg (0.207 mmol) of **2b** under nitrogen at -10 ºC. After 2 h stirring at that temperature the excess of cyclopentadiene was evaporated under vacuum at -10 ºC, and a H-NMR and C-NMR were recorded for full characterization. A parallel reaction was performed with 11.5 mg (0.068 mmol, 0.33 equiv.) of 1,3,5-trimethoxybenzene as internal standard and a qNMR was recorded after 2 h. (NMR yield: 92%)

^{1}H NMR (400 MHz, $CDCl_3$) δ 8.21 (d, *J* = 7.6 Hz, 1H, NH), 7.92 (dd, *J* = 7.7, 1.6 Hz, 1H, ArH), 7.28 – 7.20 (m, 1H, ArH), 7.11 (td, *J* = 7.5, 1.3 Hz, 1H, ArH), 7.06 (dd, *J* = 8.0, 1.2 Hz, 1H, ArH), 6.58 – 6.51 (m, 1H, CH=), 5.81 (dt, *J* = 5.8, 2.1 Hz, 1H, CH=), 5.24 (dt, *J* = 1.7, 0.8 Hz, 1H, CHON), 4.80 – 4.74 (m, 1H, CHNO), 4.53 – 4.42 (m, 1H, CHN), 2.10 – 2.00 (m, 3H, CH_2 + C*H*H), 1.79 (dt, *J* = 8.6, 1.1 Hz, 1H, C*H*H), 1.73 – 1.65 (m, 4H, 2xCH_2), 1.56 – 1.46 (m, 2H, CH_2). **^{13}C{^{1}H} NMR (101 MHz, $CDCl_3$)** δ 166.3 (CO), 147.6 (C), 134.3 (CH), 132.2 (CH), 130.6 (CH), 130.5 (CH), 125.4 (C), 124.2 (CH), 119.7 (CH), 83.1 (CH), 70.2 (CH), 51.1 (CH), 48.1 (CH_2), 33.13 (CH_2), 33.05 (CH_2), 23.82 (CH_2), 23.78 (CH_2).

9a: diethyl (2-(isobutylcarbamoyl)phenyl)phosphoramidate

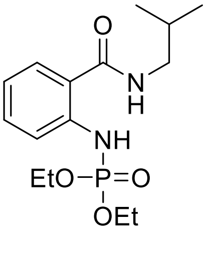

94 µL of triethylphosphite (0.545 mmol, 2.5 equiv.) are added to 45 mg (0.218 mmol) of **2b** with few drops of chloroform. The mixture is heated at 120 ºC for 1h. Then, the reaction is cooled down and diluted with DCM.

The solution is washed with brine twice. The organic layer is dried with sodium sulphate and solvent evaporated in vacuo. 23 mg (0.070 mmol, 32% yield) were obtained as a pale orange solid after purification using column chromatography (Pentane:Et_2O; 4:1). **M. p.**: 102 – 104 ºC. **^{1}H NMR (500 MHz, $CDCl_3$)** δ 9.36 (d, *J* = 10.9 Hz, 1H, NH), 7.48 (dd, *J* = 8.4, 1.2 Hz, 1H, ArH), 7.41 (dt, *J* = 7.8, 1.8 Hz, 1H, ArH), 7.36 (ddd, *J* = 8.5, 7.3, 1.5 Hz, 1H, ArH), 6.96 – 6.87 (m, 1H, ArH), 6.30 (bs, 1H, NH), 4.20 – 4.06 (m, 4H, 2xCH_2), 3.24 (dd, *J* = 6.9, 5.9 Hz, 2H, CH_2), 1.89 (dt, *J* = 13.5, 6.7 Hz, 1H, CH_2), 1.32 (td, *J* = 7.1, 0.9 Hz, 6H, 2xCH_3), 0.97 (d, *J* = 6.7 Hz, 6H, 2xCH_3). **^{13}C{^{1}H} NMR (126 MHz, $CDCl_3$)** δ 169.0 (C), 142.2 (C), 132.5 (CH), 126.6 (CH), 120.3 (CH), 119.0 (d, *J* = 9.6 Hz, C), 118.8 (d, *J* = 2.5 Hz, CH), 63.0 (d, *J* = 5.4 Hz), 47.1 (CH_2), 28.5 (CH), 20.2 (2xCH_3), 16.1 (d, *J* = 7.0 Hz, 2xCH_3). **^{31}P NMR (162 MHz, $CDCl_3$)** δ 1.25. **HRMS** (TOF-ESI+) m/z: $[M+H]^+$ Calcd for $C_{15}H_{26}N_2O_4P$ 329.1625, found 329.1628.

9b: diethyl (2-(cyclopentylcarbamoyl)phenyl)phosphoramidate

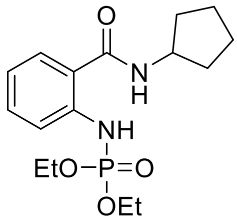

89 µL of triethylphosphite (0.515 mmol, 2.5 equiv.) are added to 45 mg (0.206 mmol) of **2b** with few drops of chloroform. The mixture is heated at 120 ºC for 1h. Then, the reaction is cooled down and diluted with DCM. The solution is washed with brine twice. The organic layer is dried with sodium sulfate and solvent evaporated in vacuo. 22 mg (0.068 mmol, 33% yield) were obtained as a yellow oil after purification using column chromatography (Pentane:Et_2O; 4:1). **^{1}H NMR (600 MHz, $CDCl_3$)** δ 9.38 (d, *J* = 10.9 Hz, 1H, NH), 7.49 – 7.44 (m, 1H, ArH), 7.38 – 7.32 (m, 2H, ArH), 6.90 (t, *J* = 7.3 Hz, 1H, ArH), 6.05 (d, *J* = 7.2 Hz, 1H, NH), 4.33 (h, *J* = 6.9 Hz, 1H, CH), 4.22 – 4.04 (m, 4H, 2xCH_2O), 2.12 – 2.03 (m, 2H, CH_2), 1.74 – 1.63 (m, 4H, 2xCH_2), 1.52 – 1.43 (m, 2H, CH_2), 1.31 (t, *J* = 7.1 Hz, 6H, 2xCH_3). **^{13}C{^{1}H} NMR (151 MHz, $CDCl_3$)** δ 168.6 (C), 142.2 (C), 132.5 (CH), 126.6 (CH), 120.2 (CH), 118.9 (d, *J* = 9.3 Hz, C), 118.8 (CH), 63.0 (d, *J* = 5.4 Hz, 2xCH_2O), 51.6 (CH), 33.2 (2xCH_2). 23.8 (2xCH_2), 16.1 (d, *J* = 6.9 Hz, 2xCH_3) .**^{31}P NMR (162 MHz, $CDCl_3$)** δ 1.96). **HRMS** (TOF-ESI+) m/z: $[M+H]^+$ Calcd for $C_{16}H_{26}N_2O_4P$ 341.1625, found 341.1629.

General procedure for *o*-nitrophenylimines synthesis: (**1a-o**) To a solution of the corresponding amine (1 equiv.) in ethanol (0.4 M), the corresponding aldehyde (500 mg, 1 equiv.) is added. The mixture is stirred for 12 h at 65 ºC. Then, the solvent is evaporated in vacuo, and the corresponding product is obtained with no further purification unless otherwise specified.

Characterization data of starting materials (o-nitrophenylimines): 1a-1o

General procedure for starting material synthesis (*o*-nitrophenylimines): (**1a-o**) To a solution of the corresponding amine (1 equiv.) in ethanol (0.4 M), the corresponding aldehyde (200 mg or 500 mg, 1 equiv.) is added. The mixture is stirred for 12 h at 65 ºC. Then, the solvent is evaporated in vacuo, and the corresponding product is obtained with no further purification unless otherwise specified.

1a: (*E*)-*N*-isobutyl-1-(2-nitrophenyl)methanimine

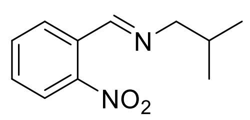

Following the general procedure, using 2-nitrobenzaldehyde (500 mg, 3.30 mmol) and isobutylamine (348 µL, 3.30 mmol), 640 mg (3.10 mmol, 94% yield) of the title compound were obtained as dark yellow oil. **^{1}H NMR (500 MHz, $CDCl_3$)** δ 8.64 (d, J = 1.5 Hz, 1H, CH=), 8.05 (dd, J = 7.8, 1.5 Hz, 1H, ArH), 8.00 (dd, J = 8.2, 1.2 Hz, 1H, ArH), 7.65 (tdd, J = 7.8, 1.3, 0.6 Hz, 1H, ArH), 7.55 (ddd, J = 8.2, 7.4, 1.5 Hz, 1H, ArH), 3.51 (dd, J = 6.6, 1.4 Hz, 2H, CH_2), 2.04 (dp, J = 13.3, 6.7 Hz, 1H, CH), 0.99 (d, J = 6.7 Hz, 6H, 2x CH_3). **^{13}C{^{1}H} NMR (126 MHz, $CDCl_3$)** δ 156.7 (CH), 148.0 (C), 133.4 (CH), 131.4 (C), 130.4 (CH), 129.7 (CH), 124.2 (CH), 69.7 (CH_2), 29.5 (CH), 20.6 (2xCH_3). **HRMS** (TOF-ESI+) m/z: $[M+H]^+$ Calcd for $C_{11}H_{15}N_2O_2$ 207.1133, found 207.1130.

1b: (*E*)-*N*-cyclopentyl-1-(2-nitrophenyl)methanimine

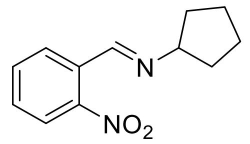

Following the general procedure, using 2-nitrobenzaldehyde (500 mg, 3.30 mmol) and cyclopentylamine (326 µL, 3.30 mmol), 677 mg (3.10 mmol, 94% yield) of the title compound were obtained as a light orange oil. **^{1}H NMR (400 MHz, $CDCl_3$)** δ 8.69 (s, 1H, CH=), 8.05 (dd, *J* = 7.8, 1.5 Hz, 1H, ArH), 7.99 (dd, *J* = 8.2, 1.3 Hz, 1H, ArH), 7.68 – 7.61 (m, 1H, ArH), 7.53 (ddd, *J* = 8.2, 7.4, 1.5 Hz, 1H, ArH), 3.94 – 3.83 (m, 1H, CHN), 1.99 – 1.79 (m, 4H, 2xCH_2), 1.77 – 1.63 (m, 4H, 2xCH_2). **^{13}C{^{1}H} NMR (101 MHz, $CDCl_3$)** δ 154.4 (CH), 148.8 (C), 133.4 (CH), 131.6 (C), 130.3 (CH), 129.9 (CH), 129.8 (CH), 124.2 (CH), 71.7 (CH), 34.4 (2xCH_3), 24.8 (2xCH_3). **HRMS** (TOF-ESI+) m/z: $[M+H]^+$ Calcd for $C_{12}H_{15}N_2O_2$ 219.1128, found 219.1128.

1c: (*E*)-*N*-cyclohexyl-1-(2-nitrophenyl)methanimine

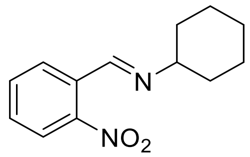

Following the general procedure, using 2-nitrobenzaldehyde (500 mg, 3.30 mmol) and cyclohexylamine (379 µL, 3.30 mmol), 702 mg (3.01 mmol, 91% yield) of the title compound were obtained as a yellow oil. **^{1}H NMR (500 MHz, $CDCl_3$)** δ 8.72 (s, 1H, CH=), 8.04 (dd, *J* = 7.8, 1.5 Hz, 1H, ArH), 8.00 (dd, *J* = 8.2, 1.2 Hz, 1H, ArH), 7.68 – 7.62 (m, 1H, ArH), 7.53 (ddd, *J* = 8.1, 7.4, 1.5 Hz, 1H, ArH), 3.35 – 3.28 (m, 1H, CH), 1.87 – 1.75 (m, 4H, 2xCH_2), 1.71 – 1.65 (m, 1H,*CH*H), 1.63 – 1.55 (m, 2H, CH_2), 1.43 – 1.33 (m, 2H, CH_2), 1.28 (tt, *J* = 12.1, 3.3 Hz, 1H, *CH*H). The reported data is in accordance with literature.[2]

1d: (*E*)-*N*-*tert*-butyl-1-(2-nitrophenyl)methanimine

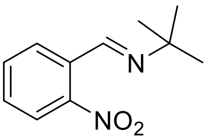

Following the general procedure, using 2-nitrobenzaldehyde (500 mg, 3.30 mmol) and tert-butylamine (346 µL, 3.30 mmol), 598 mg (2.90 mmol, 88% yield) of the title compound are obtained as a light orange oil. **^{1}H NMR (400 MHz, $CDCl_3$)** δ 8.67 (s, 1H, CH), 8.09 – 7.95 (m, 2H, ArH), 7.66 (tdd, *J* = 7.8, 1.3, 0.6 Hz, 1H, ArH), 7.53 (ddd, *J* = 8.1, 7.4, 1.5 Hz, 1H, ArH), 1.33 (s, 9H, 3xCH_3). The reported data is in accordance with literature.[2]

1e: (*E*)-*N*-decyl-1-(2-nitrophenyl)methanimine

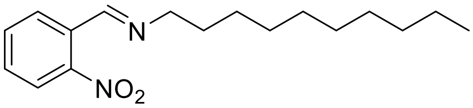

Following the general procedure, using 2-nitrobenzaldehyde (150 mg, 0.99 mmol) and decanamine (155 mg, 0.99 mmol), 285 mg (0.99 mmol, 99% yield) of the title compound were obtained as a yellow oil. **^{1}H NMR (400 MHz, $CDCl_3$)** δ 8.68 (s, 1H, CH), 8.04 (dd, *J* = 7.8, 1.5 Hz, 1H, ArH), 8.01 (dd, *J* = 8.2, 1.3 Hz, 1H, ArH), 7.70 – 7.60 (m, 1H, ArH), 7.55 (ddd, *J* = 8.9, 7.4, 1.5 Hz, 1H, ArH), 3.68 (td, *J* = 7.0, 1.4 Hz, 2H, CH_2N), 1.79 – 1.66 (m, 2H, CH_2), 1.40 – 1.22 (m, 14H, 7xCH_2), 0.91 – 0.82 (m, 3H, CH_3). **^{13}C{^{1}H} NMR** δ 156.6 (CH), 148.8 (C), 133.4 (CH), 131.4 (C), 130.4 (CH), 129.7 (CH), 124.3 (CH), 61.8 (CH_2), 31.9 (CH_2), 30.6 (CH_2), 29.6 (2xCH_2), 29.4 (CH_2), 29.3 (CH_2), 27.3 (CH_2), 22.7 (CH_2), 14.1 (CH_3). **HRMS** (TOF-ESI+) m/z: $[M+H]^+$ Calcd for $C_{17}H_{27}N_2O_2$ 291.2067, found 291.2071.

1f: (*E*)-*N*-(2-methoxybenzyl)-1-(2-nitrophenyl)methanimine

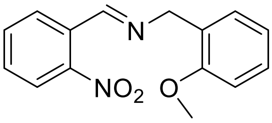

Following the general procedure, using 2-nitrobenzaldehyde (200 mg, 1.32 mmol) and (2-methoxyphenyl)methanamine (182 mg, 1.32 mmol), 341 mg (1.27 mmol, 84% yield) of the title compound were obtained as a pale yellow solid. **M. p.**: 65 – 66 ºC. **^{1}H NMR (400 MHz, $CDCl_3$)** δ 8.78 (s, 1H, CH), 8.11 (dd, *J* = 7.8, 1.5 Hz, 1H, ArH), 8.01 (ddd, *J* = 8.2, 1.3, 0.4 Hz, 1H, ArH), 7.70 – 7.60 (m, 1H, ArH), 7.55 (ddd, *J* = 8.1, 7.4, 1.6 Hz, 1H, ArH), 7.36 – 7.26 (m, 2H, ArH), 6.96 (td, *J* = 7.4, 1.1 Hz, 1H, ArH), 6.90 (dd, *J* = 8.2, 1.1 Hz, 1H, ArH), 4.89 (d, *J* = 1.5 Hz, 2H, CH_2), 3.87 (s, 3H, CH_3). **^{13}C{^{1}H} NMR (101 MHz, $CDCl_3$)** δ 157.9 (C), 157.4 (C), 148.9 (C), 133.4 (CH), 131.5 (C), 130.5 (CH), 129.9 (CH), 129.8 (CH), 128.6 (CH), 126.6 (C),

124.2 (CH), 120.6 (CH), 110.3 (CH), 59.7 (CH_2), 55.3 (CH_3). **HRMS** (TOF-ESI+) m/z: $[M+H]^+$ Calcd for $C_{15}H_{15}N_2O_3$ 271.1077, found 271.1078.

1g: (*E*)-*N*-(4-fluorobenzyl)-1-(2-nitrophenyl)methanimine

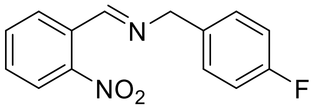

Following the general procedure, using 2-nitrobenzaldehyde (200 mg, 1.32 mmol) and (4-fluorophenyl)methanamine (165 mg, 1.32 mmol), 312 mg (1.28 mmol, 97% yield) of the title compound were obtained as pale yellow solid. **M. p.**: 56 – 58 ºC. **^{1}H NMR (400 MHz, $CDCl_3$)** δ 8.83 (s, 1H, CH), 8.09 (dd, *J* = 7.8, 1.6 Hz, 1H, ArH), 8.04 (dd, *J* = 8.2, 1.3 Hz, 1H, ArH), 7.72 – 7.63 (m, 1H, ArH), 7.58 (ddd, *J* = 8.1, 7.4, 1.5 Hz, 1H, ArH), 7.37 – 7.28 (m, 2H, ArH), 7.13 – 6.96 (m, 2H, ArH), 4.85 (s, 1H, CH_2). **^{13}C{^{1}H} NMR (101 MHz, $CDCl_3$)** δ 162.2 (d, *J* = 245.2 Hz, C), 158.0 (CH), 149.0 (C), 134.33 (d, *J* = 3.2 Hz, C), 133.6 (CH), 131.2 (C), 130.9 (CH), 129.9 (CH), 129.8 (d, *J* = 8.2 Hz, 2xCH), 124.5 (CH), 115.5 (d, *J* = 21.3 Hz, 2xCH), 64.6 (CH_2). **^{19}F NMR (376 MHz, $CDCl_3$)** δ -115.62 (tt, *J* = 8.9, 5.4 Hz). **HRMS** (TOF-ESI+) m/z: $[M+H]^+$ Calcd for $C_{14}H_{12}FN_2O_2$ 259.0877, Found at 259.0877.

1h: methyl (*E*)-2-((2-nitrobenzylidene)amino)-3-phenylpropanoate

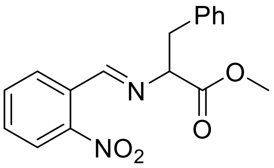

Following the general procedure, using 2-nitrobenzaldehyde (150 mg, 0.99 mmol) and methyl phenylalaninate (214 mg, 0.99 mmol), 253 mg (0.81 mmol, 82% yield) of the title compound were obtained as a yellow oil. In this case, the crude was filtered through a pad of basic alumina to purify the product. **^{1}H NMR (500 MHz, $CDCl_3$)** δ 8.81 (s, 1H, CH), 8.05 (dd, *J* = 7.8, 1.6 Hz, 1H, ArH), 8.01 (dd, *J* = 8.1, 1.2 Hz, 1H, ArH), 7.68 – 7.62 (m, 1H, ArH), 7.55 (ddd, *J* = 8.1, 7.4, 1.5 Hz, 1H, ArH), 7.48 – 7.42 (m, 2H, ArH), 7.41 – 7.33 (m, 2H, ArH), 7.32 – 7.27 (m, 1H, ArH), 4.97 (dd, *J* = 9.4, 4.6 Hz, 1H, CH), 3.67 (s, 3H, CH_3), 3.07 (dd, *J* = 15.5, 9.4 Hz, 1H, C*H*H), 2.93 (dd, *J* = 15.5, 4.5 Hz, 1H, C*H*H). **^{13}C{^{1}H} NMR (101 MHz, $CDCl_3$)** δ 171.3 (C), 157.6 (CH), 148.8 (C), 141.8 (C), 133.4 (CH), 131.1 (C), 130.8 (CH), 130.1 (CH), 128.8 (2xCH_2), 127.7 (CH), 126.9 (2xCH_2), 124.3 (CH), 71.0 (CH), 51.8 (CH_3), 42.9 (CH_2). **HRMS** (TOF-ESI+) m/z: $[M+H]^+$ Calcd for $C_{17}H_{17}N_2O_4$ 313.1183, Found at 313.1184.

1i: (*E*)-1-(2-nitrophenyl)-N-phenylmethanimine

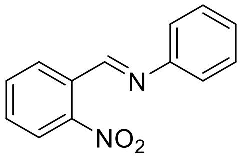

Following the general procedure, using 2-nitrobenzaldehyde (500 mg, 3.30 mmol) and aniline (307 mg, 3.30 mmol), 677 mg (3.10 mmol, 94% yield) of the title compound were obtained as a yellow solid. **^{1}H NMR (400 MHz, $CDCl_3$)** δ 8.94 (s, 1H, CH), 8.32 (dd, *J* = 7.8, 1.5 Hz, 1H, ArH), 8.08 (dd, *J* = 8.2, 1.3 Hz, 1H, ArH), 7.79

– 7.69 (m, 1H, ArH), 7.62 (ddd, *J* = 8.1, 7.3, 1.5 Hz, 1H, ArH), 7.43 (ddd, *J* = 8.4, 6.5, 0.9 Hz, 2H, ArH), 7.29 (tdd, *J* = 5.8, 1.9, 0.9 Hz, 3H). The reported data is in accordance with literature.[2]

1j: (*E)*-*N*-(4-fluorophenyl)-1-(2-nitrophenyl)methanimine

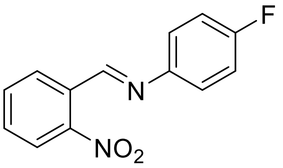

Following the general procedure, using 2-nitrobenzaldehyde (500 mg, 3.30 mmol) and 4-flouroaniline (367 mg, 3.30 mmol), 805 mg (3.30 mmol, 99% yield) of the title compound were obtained as a yellow solid. **M. p.**: 81 – 82 ºC. **^{1}H NMR (400 MHz, CDCl$_3$)** δ 8.93 (s, 1H, CH), 8.29 (dd, *J* = 7.8, 1.5 Hz, 1H, ArH), 8.08 (dd, *J* = 8.2, 1.3 Hz, 1H, ArH), 7.81 – 7.71 (m, 1H, ArH), 7.63 (ddd, *J* = 8.1, 7.4, 1.5 Hz, 1H, ArH), 7.32 – 7.25 (m, 2H, ArH), 7.18 – 7.08 (m, 2H, ArH). **^{13}C{^{1}H} NMR (101 MHz, CDCl$_3$)** δ 161.8 (d, *J* = 246.3 Hz, C), 155.50 (d, *J* = 1.8 Hz, CH), 149.3 (C), 147.03 (d, *J* = 3.0 Hz, C), 133.6 (CH), 131.2 (CH), 131.0 (C), 129.6 (CH), 124.6 (CH), 122.79 (d, *J* = 8.4 Hz, 2xCH), 116.1 (d, *J* = 22.8 Hz, 2xCH). **^{19}F NMR (376 MHz, CDCl$_3$)** δ -115.66 (tt, *J* = 8.2, 4.9 Hz). **HRMS** (TOF-ESI+) m/z: [M+H]$^+$ Calcd for $C_{13}H_{10}FN_2O_2$ 245.0721, found 245.0721.

1k: (*E*)-*N*-(4-isopropylphenyl)-1-(2-nitrophenyl)methanimine

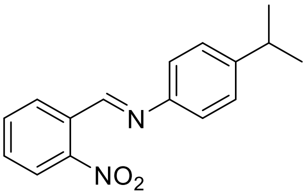

Following the general procedure, using 2-nitrobenzaldehyde (500 mg, 3.30 mmol) and 4-isopropylaniline (451 µL, 3.30 mmol), 741 mg (2.77 mmol, 84% yield) of the title compound were obtained as a pale orange solid. In this case, the crude was filtered through a pad of basic alumina to purify the product. **^{1}H NMR (400 MHz, CDCl$_3$)** δ 8.96 (s, 1H, CH), 8.32 (dd, *J* = 7.8, 1.5 Hz, 1H, ArH), 8.12 – 8.00 (m, 1H, ArH), 7.79 – 7.66 (m, 1H, ArH), 7.61 (ddd, *J* = 8.2, 7.4, 1.5 Hz, 1H, ArH), 7.33 – 7.20 (m, 4H, ArH), 2.95 (p, *J* = 6.9 Hz, 1H, CH), 1.28 (d, *J* = 6.9 Hz, 6H, 2xCH_3). The reported data is in accordance with literature.[2]

1l: (*E*)-*N*-cyclopentyl-1-(5-fluoro-2-nitrophenyl)methanimine

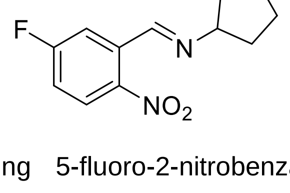

Following the general procedure, using 5-fluoro-2-nitrobenzaldehyde (250 mg, 1.48 mmol) and cyclopentylamine (146 µL, 1.48 mmol), 322 mg (1.36 mmol, 92% yield) of the title compound were obtained as a yellow solid. **M. p.**: 105 – 106 ºC. **^{1}H NMR (400 MHz, CDCl$_3$)** δ 8.71 (dd, *J* = 2.0, 0.7 Hz, 1H, CH), 8.08 (dd, *J* = 9.0, 4.9 Hz, 1H, ArH), 7.78 (dd, *J* = 9.0, 2.9 Hz, 1H, ArH), 7.19 (ddd, *J* = 9.0, 7.0, 2.9 Hz, 1H, ArH), 4.12 – 3.82 (m, 1H, CH), 2.05 – 1.77 (m, 4H, 2xCH_2), 1.77 – 1.53 (m, 4H, 2xCH_2). **^{13}C{^{1}H} NMR (126 MHz, CDCl$_3$)** δ 164.9 (d, *J* = 257.3 Hz, C), 153.4 (d, *J* = 1.9 Hz, CH), 144.7 (C), 135.0 (d, *J* = 9.0 Hz, C), 127.3 (d,

J = 9.6 Hz, CH), 117.28 (d, *J* = 23.9 Hz, CH), 116.48 (d, *J* = 24.8 Hz, CH), 71.6 (CH), 34.4 (2xCH_2), 24.8 (2xCH_2). **^{19}F NMR (376 MHz, $CDCl_3$)** δ -102.65 (dddd, *J* = 9.0, 6.9, 4.8, 2.0 Hz). **HRMS** (TOF-ESI+) m/z: $[M+H]^+$ Calcd for $C_{12}H_{14}FN_2O_2$ 237.1034, found 237.1034.

1m: (*E*)-*N*-cyclopentyl-1-(6-nitrobenzo[d][1,3]dioxol-5-yl)methanimine

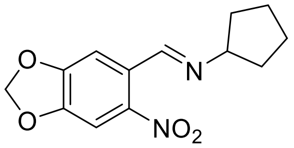

Following the general procedure, using 6-nitrobenzo[d][1,3]dioxole-5-carbaldehyde (200 mg, 1.02 mmol) and cyclopentylamine (101 µL, 1.02 mmol), 262 mg (1.00 mmol, 98% yield) of the title compound were obtained as a pale orange solid. **M. p.**: 75 – 77 ºC. **^{1}H NMR (400 MHz, $CDCl_3$)** δ 8.66 (s, 1H, CH), 7.48 (s, 1H, ArH), 7.47 (s, 1H, ArH), 6.13 (s, 2H, CH_2O), 3.90 – 3.82 (m, 1H, CH), 1.97 – 1.80 (m, 4H, 2xCH_2), 1.76 – 1.62 (m, 4H, 2xCH_2). **^{13}C{^{1}H} NMR (101 MHz, $CDCl_3$)** δ 154.4 (CH), 151.9 (C), 149.0 (C), 143.7 (C), 129.0 (C), 108.1 (CH), 104.8 (CH), 103.1 (CH_2), 71.4 (CH), 34.5 (2xCH_2), 24.7 (2xCH_2). **HRMS** (TOF-ESI+) m/z: $[M+H]^+$ Calcd for $C_{13}H_{15}N_2O_4$ 263.1026, found 263.1027.

1n: methyl (*E*)-4-((*tert*-butylimino)methyl)-3-nitrobenzoate

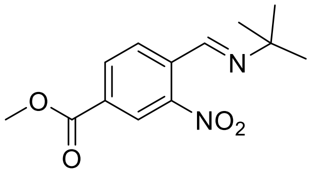

Following the general procedure, using methyl 4-formyl-3-nitrobenzoate (250 mg, 1.20 mmol) and tert-butylamine (126 µL, 1.20 mmol), 304 mg (1.15 mmol, 96% yield) of the title compound were obtained as a pale yellow solid. **M. p.**: 54 – 56 ºC. For this compound the reaction was performed in methanol as solvent. **^{1}H NMR (500 MHz, $CDCl_3$)** δ 8.67 (s, 1H, CH), 8.65 (d, *J* = 1.6 Hz, 1H, ArH), 8.28 (ddd, *J* = 8.1, 1.7, 0.7 Hz, 1H, ArH), 8.11 (d, *J* = 8.1 Hz, 1H, ArH), 3.98 (s, 3H, CH_3O), 1.33 (s, 9H, 3xCH_3). **^{13}C{^{1}H} NMR (126 MHz, $CDCl_3$)** δ 164.8 (CO), 151.2 (CH), 148.7 (C), 135.9 (C), 133.9 (CH), 132.1 (C), 130.1 (CH), 125.5 (CH), 58.8 (CH_3), 52.8 (C), 29.4 (3xCH_3). **HRMS** (TOF-ESI+) m/z: $[M+H]^+$ Calcd for $C_{13}H_{17}N_2O_4$ 265.1183, found 265.1185.

1o: (*E*)-*N*-*tert*-butyl-1-(3-methyl-2-nitrophenyl)methanimine

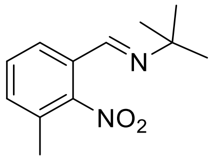

Following the general procedure, using 3-methyl-2-nitrobenzaldehyde (200 mg, 1.21 mmol) and tert-butylamine (127 µL, 1.21 mmol), 255 mg (1.16 mmol, 96% yield) of the title compound were obtained as an pale orange solid. **M. p.**: 45 – 48 ºC **^{1}H NMR (500 MHz, $CDCl_3$)** δ 8.16 (s, 1H, CH), 7.86 – 7.78 (m, 1H, ArH),

7.41 (t, J = 7.7 Hz, 1H, ArH), 7.32 (ddd, J = 7.5, 1.5, 0.7 Hz, 1H, ArH), 2.36 (s, 3H, CH_3), 1.26 (s, 9H, 3xCH_3). **$^{13}C\{^1H\}$ NMR (101 MHz, $CDCl_3$)** δ 151.0 (C), 149.4 (CH), 132.6 (CH), 130.3 (CH), 130.0 (C), 129.0 (C), 126.3 (CH), 58.3 (C), 29.3 (CH_3), 17.4 (3xCH_3). **HRMS** (TOF-ESI+) m/z: $[M+H]^+$ Calcd for $C_{12}H_{17}N_2O_2$ 221.1285, found 221.1284.

X-Ray data

The X-ray diffraction data for compounds **5b, 2d,** and **4** were collected using a SuperNova Dual four-circle diffractometer, featuring a micro-focus sealed X-ray tube, a mirror monochromator, and an Atlas detector.

The diffractometer was equipped with a low temperature device and used Cu K_{α} radiation (λ = 1.54184 Å). Data integration was performed with Crysalispro, and a multi-scan absorption correction using SCALE3 ABSPACK was applied. [3]

The structure was solved by dual methods using SHELXT and refined by full-matrix least-squares methods against F^2 by SHELXL[4,5] using OLEX2[6] as an interface. Anisotropic displacement parameters were applied to refine all non-hydrogen atoms, while hydrogen atoms were refined with isotropic displacement parameters. Some hydrogen atoms were freely refined, while others were refined at calculated positions using a riding model. The Uiso values for hydrogen atoms were constrained to 1.5 times the Ueq of their respective pivot atoms for terminal sp3 carbon atoms and 1.2 times for all other carbon atoms.

Mercury was used for making the figures depicted in the manuscript.[7] Crystallographic data for the structure reported in this paper have been deposited with the Cambridge Crystallographic Data Centre.[8]

Table S4. Crystal data and structure refinement

	5b: CDC2300553	2d: CDC2300555	4: CDC2300554
Empirical formula	$C_{12}H_{14}N_2O_2$	$C_{22}H_{28}N_4O_4$	$C_{14}H_{15}F_3N_2O_2$
Formula weight	218.25	412.48	300.28
Temperature [K]	113(12)	105.0(3)	103(5)
Crystal system	monoclinic	orthorhombic	monoclinic
Space group (number)	$C2/c$ (15)	$Pbca$ (61)	$P2_1/c$ (14)
a [Å]	20.9404(9)	18.49388(2)	13.1526(4)
b [Å]	5.0661(2)	12.0798(4)	4.9415(2)
c [Å]	20.1618(5)	20.3049(3)	20.8868(6)
α [°]	90	90	90
β [°]	91.307(3)	90	97.327(3)
γ [°]	90	90	90
Volume [$Å^3$]	2138.33(14)	4536.17(16)	1346.42(8)
Z	8	8	4
ρ_{calc} [gcm^{-3}]	1.356	1.208	1.481
μ [mm^{-1}]	0.764	0.689	1.104
F(000)	928	1760	624
Crystal size [mm^3]	0.33×0.174×0.052	0.373×0.209×0.087	0.21×0.05×0.04
Crystal colour	Clear light gold	Clear light white	Clear pale yellow
Crystal shape	Block	Block	Block
Radiation	Cu K_α (λ=1.54184 Å)	Cu K_α (λ=1.54184 Å)	Cu K_α (λ=1.54184 Å)
2Θ range [°]	8.45 to 152.55 (0.79 Å)	8.71 to 152.77 (0.79 Å)	6.78 to 152.94 (0.79 Å)
Index ranges	-26 ≤ h ≤ 26<br>-6 ≤ k ≤ 4<br>-25 ≤ l ≤ 25	-23 ≤ h ≤ 18<br>-15 ≤ k ≤ 15<br>-25 ≤ l ≤ 25	-16 ≤ h ≤ 16<br>-5 ≤ k ≤ 6<br>-25 ≤ l ≤ 26
Reflections collected	12636	53200	16439
Independent reflections	2234<br>R_{int} = 0.0626<br>R_{sigma} = 0.0434	4745<br>R_{int} = 0.0562<br>R_{sigma} = 0.0200	2806<br>R_{int} = 0.0454<br>R_{sigma} = 0.0311
Completeness	100.0 %	100.0 %	100.0 %
Data / Restraints / Parameters	2234/0/153	4745/0/285	2806/0/194
Goodness-of-fit on F^2	1.070	1.054	1.040
Final *R* indexes [$I \geq 2\sigma(I)$]	R_1 = 0.0493<br>wR_2 = 0.1144	R_1 = 0.0333<br>wR_2 = 0.0840	R_1 = 0.0472<br>wR_2 = 0.1175
Final *R* indexes [all data]	R_1 = 0.0798<br>wR_2 = 0.1384	R_1 = 0.0366<br>wR_2 = 0.0873	R_1 = 0.0661<br>wR_2 = 0.1314
Largest peak/hole [$eÅ^{-3}$]	0.28/-0.23	0.22/-0.27	0.81/-0.34

Figure S1. Asymmetric unit of **5b (CDC2300553)**

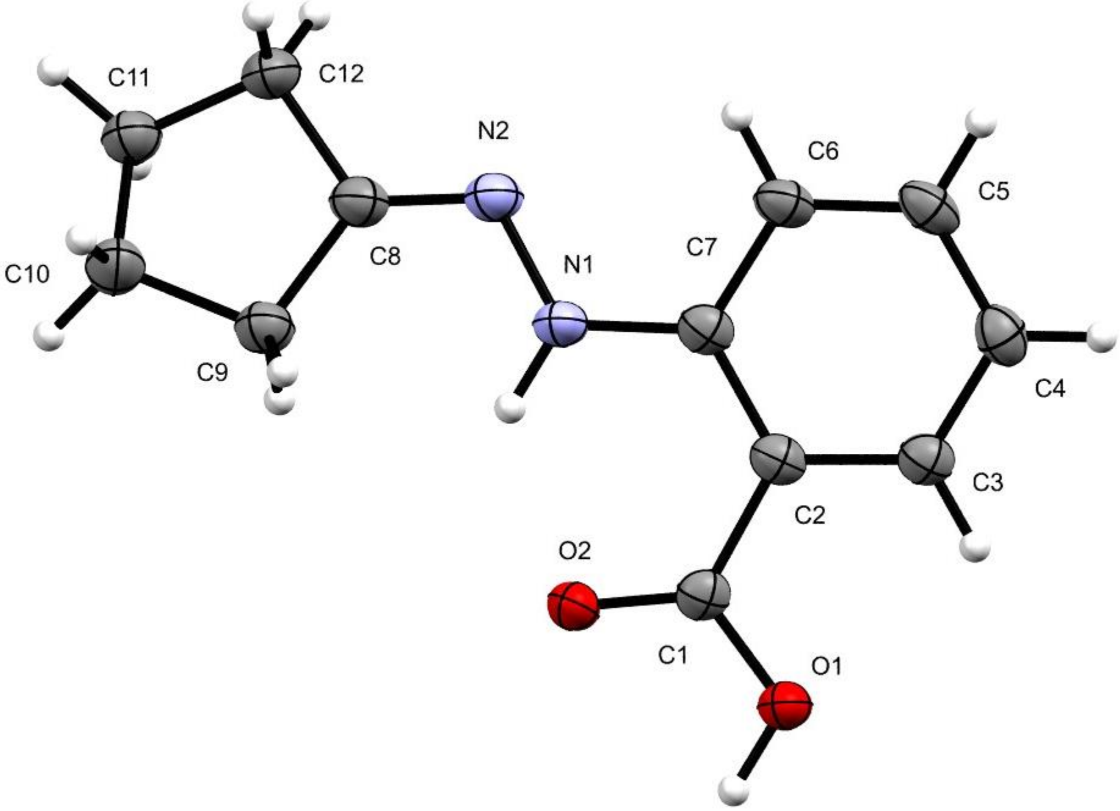

Showing the atom-labelling scheme. Displacement ellipsoids are drawn at the 50% probability level. Graphics were obtained using Mercury 3.0.[7]

Figure S2. Asymmetric unit of **2d (CDC2300555)**

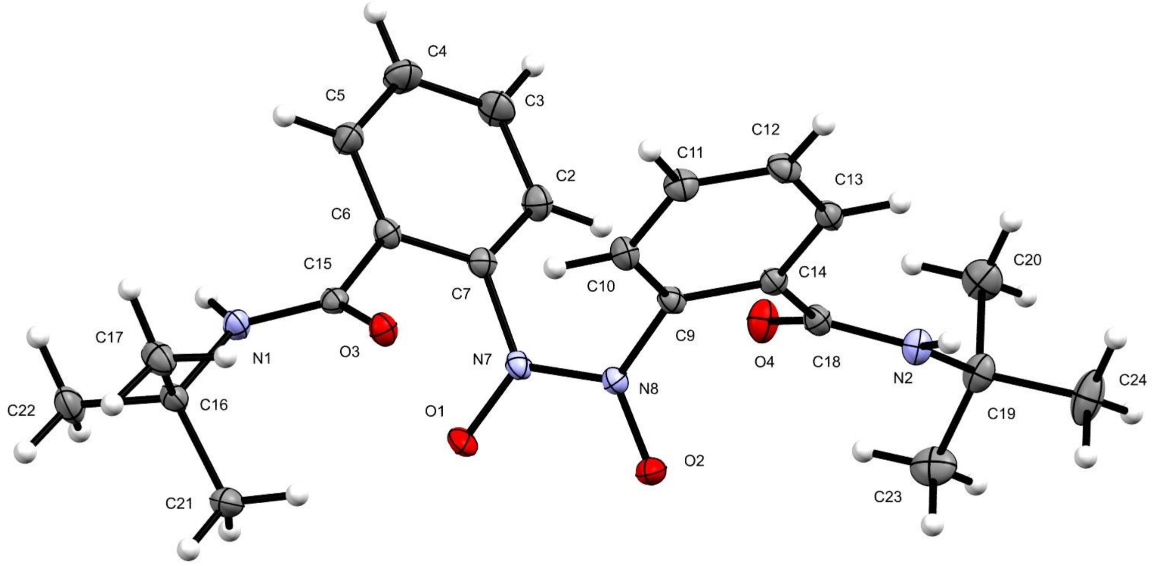

Showing the atom-labelling scheme. Displacement ellipsoids are drawn at the 50% probability level. Graphics were obtained using Mercury 3.0.[7]

Figure S3. Asymmetric unit of **4 (CDC2300554)**

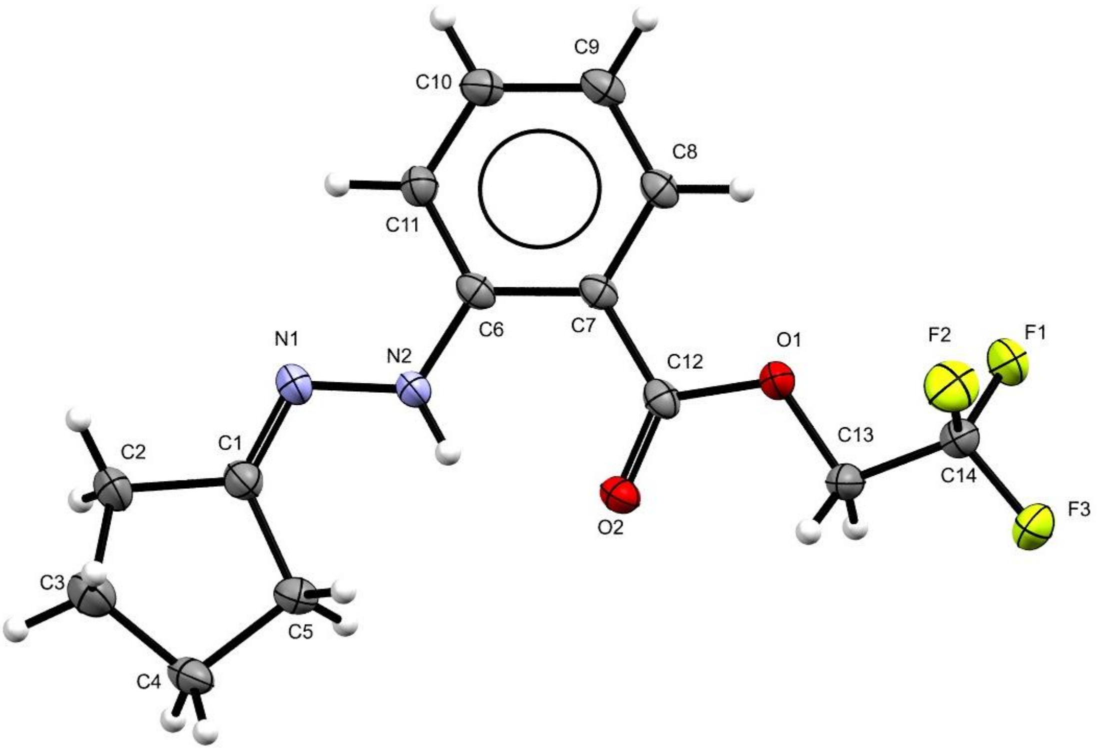

Showing the atom-labelling scheme. Displacement ellipsoids are drawn at the 50% probability level. Graphics were obtained using Mercury 3.0.[7]

Table S5. Atomic coordinates and Ueq [Å2] for compound 5b

Atom	x	y	z	Ueq
O2	0.68423(7)	0.6209(3)	0.53399(7)	0.0273(4)
O1	0.78548(7)	0.4812(3)	0.54424(7)	0.0282(4)
N1	0.59855(9)	0.4059(4)	0.61478(9)	0.0282(4)
N2	0.54384(9)	0.4157(4)	0.65230(8)	0.0300(4)
C1	0.72457(10)	0.4690(4)	0.55921(9)	0.0242(4)
C7	0.64793(10)	0.2431(4)	0.63542(9)	0.0254(5)
C2	0.70925(10)	0.2641(4)	0.60810(9)	0.0243(4)
C3	0.75754(10)	0.0888(4)	0.62939(9)	0.0268(5)
H3	0.798800	0.102391	0.611060	0.032
C6	0.63727(11)	0.0481(4)	0.68394(10)	0.0289(5)
H6	0.596465	0.032826	0.703213	0.035
C4	0.74625(11)	-0.1024(4)	0.67629(10)	0.0294(5)
H4	0.779227	-0.220649	0.689921	0.035
C5	0.68589(11)	-0.1200(4)	0.70342(10)	0.0297(5)
H5	0.678041	-0.250643	0.736045	0.036
C8	0.50190(11)	0.5844(5)	0.63342(10)	0.0298(5)
C9	0.50517(11)	0.7792(5)	0.57676(10)	0.0311(5)
H9A	0.511236	0.686986	0.534075	0.037
H9B	0.540600	0.906041	0.584067	0.037
C11	0.42050(11)	0.9018(5)	0.64996(11)	0.0335(5)
H11A	0.443307	1.034374	0.677708	0.040
H11B	0.373888	0.927282	0.654132	0.040
C12	0.44030(11)	0.6208(5)	0.66947(12)	0.0351(5)
H12A	0.447101	0.604797	0.718061	0.042
H12B	0.407819	0.490373	0.654573	0.042
C10	0.44024(11)	0.9202(5)	0.57737(11)	0.0357(6)
H10A	0.444340	1.106518	0.563300	0.043
H10B	0.408787	0.830449	0.547710	0.043
H1A	0.6070(13)	0.542(6)	0.5853(13)	0.047(8)
H1	0.7947(15)	0.624(7)	0.5159(16)	0.074(11)

Ueq is defined as 1/3 of the trace of the orthogonalized Uij tensor.

Table S6. Anisotropic displacement parameters [Å2] for compound 5b

The anisotropic displacement factor exponent takes the form:

−2π2[h2(a*)2U11 + k2(b*)2U22 + … + 2hka*b*U12]

Atom	U11	U22	U33	U23	U13	U12
O2	0.0300(8)	0.0262(8)	0.0259(7)	0.0041(6)	0.0049(6)	0.0018(6)
O1	0.0281(8)	0.0274(8)	0.0296(7)	0.0037(7)	0.0075(6)	0.0009(7)
N1	0.0289(10)	0.0290(10)	0.0270(8)	0.0049(8)	0.0087(7)	0.0007(8)
N2	0.0316(10)	0.0302(10)	0.0285(9)	0.0028(8)	0.0091(7)	−0.0002(8)
C1	0.0285(11)	0.0236(10)	0.0206(9)	−0.0045(8)	0.0033(8)	0.0005(9)
C7	0.0321(11)	0.0236(11)	0.0206(9)	−0.0022(8)	0.0028(8)	−0.0002(9)
C2	0.0314(11)	0.0209(10)	0.0206(9)	−0.0022(8)	0.0023(8)	−0.0024(8)
C3	0.0311(11)	0.0256(11)	0.0238(9)	−0.0016(9)	0.0027(8)	−0.0004(9)
C6	0.0354(12)	0.0284(11)	0.0232(9)	0.0020(9)	0.0066(8)	−0.0023(9)
C4	0.0378(12)	0.0237(11)	0.0267(10)	−0.0010(9)	−0.0014(9)	0.0037(9)
C5	0.0457(13)	0.0237(10)	0.0197(9)	0.0012(8)	0.0028(9)	−0.0010(10)
C8	0.0319(12)	0.0303(12)	0.0274(10)	0.0020(9)	0.0058(8)	−0.0021(10)
C9	0.0340(12)	0.0325(12)	0.0270(10)	0.0019(9)	0.0059(9)	0.0008(10)
C11	0.0348(13)	0.0341(13)	0.0318(11)	0.0030(10)	0.0084(9)	0.0021(10)
C12	0.0322(12)	0.0357(13)	0.0378(12)	0.0068(11)	0.0117(9)	0.0024(10)
C10	0.0356(13)	0.0428(14)	0.0290(10)	0.0047(11)	0.0057(9)	0.0063(11)

Table S7. Bond lengths and angles for compound 5b

Atom–Atom	Length [Å]
O2–C1	1.243(2)
O1–C1	1.319(2)
O1–H1	0.95(4)
N1–N2	1.388(2)
N1–C7	1.380(3)
N1–H1A	0.93(3)
N2–C8	1.277(3)
C1–C2	1.472(3)
C7–C2	1.413(3)
C7–C6	1.411(3)
C2–C3	1.406(3)
C3–H3	0.9500
C3–C4	1.378(3)
C6–H6	0.9500
C6–C5	1.378(3)
C4–H4	0.9500
C4–C5	1.391(3)
C5–H5	0.9500
C8–C9	1.512(3)
C8–C12	1.506(3)
C9–H9A	0.9900
C9–H9B	0.9900
C9–C10	1.536(3)
C11–H11A	0.9900
C11–H11B	0.9900
C11–C12	1.532(3)
C11–C10	1.533(3)
C12–H12A	0.9900
C12–H12B	0.9900
C10–H10A	0.9900
C10–H10B	0.9900

Atom–Atom–Atom	Angle [°]
C1–O1–H1	113(2)
N2–N1–H1A	119.6(17)
C7–N1–N2	118.63(18)
C7–N1–H1A	118.9(17)
C8–N2–N1	115.58(18)
O2–C1–O1	121.96(19)
O2–C1–C2	123.65(19)
O1–C1–C2	114.38(18)
N1–C7–C2	121.25(19)
N1–C7–C6	119.9(2)
C6–C7–C2	118.9(2)
C7–C2–C1	121.89(19)
C3–C2–C1	118.94(19)
C3–C2–C7	119.15(19)
C2–C3–H3	119.3
C4–C3–C2	121.4(2)
C4–C3–H3	119.3
C7–C6–H6	119.9
C5–C6–C7	120.2(2)
C5–C6–H6	119.9
C3–C4–H4	120.4
C3–C4–C5	119.1(2)
C5–C4–H4	120.4

C6–C5–C4	121.3(2)
C6–C5–H5	119.3
C4–C5–H5	119.3
N2–C8–C9	128.3(2)
N2–C8–C12	121.90(19)
C12–C8–C9	109.80(19)
C8–C9–H9A	111.0
C8–C9–H9B	111.0
C8–C9–C10	104.01(18)
H9A–C9–H9B	109.0
C10–C9–H9A	111.0
C10–C9–H9B	111.0
H11A–C11–H11B	109.1
C12–C11–H11A	111.2
C12–C11–H11B	111.2
C12–C11–C10	102.97(19)
C10–C11–H11A	111.2
C10–C11–H11B	111.2
C8–C12–C11	102.68(18)
C8–C12–H12A	111.2
C8–C12–H12B	111.2
C11–C12–H12A	111.2
C11–C12–H12B	111.2
H12A–C12–H12B	109.1
C9–C10–H10A	111.0
C9–C10–H10B	111.0
C11–C10–C9	103.74(18)
C11–C10–H10A	111.0
C11–C10–H10B	111.0
H10A–C10–H10B	109.0

Table S8. Torsion angles for compound 5b

Atom–Atom–Atom–Atom	Torsion Angle [°]
O2–C1–C2–C7	−6.8(3)
O2–C1–C2–C3	174.82(19)
O1–C1–C2–C7	172.68(18)
O1–C1–C2–C3	−5.7(3)
N1–N2–C8–C9	−1.9(3)
N1–N2–C8–C12	−179.3(2)
N1–C7–C2–C1	3.4(3)
N1–C7–C2–C3	−178.21(19)
N1–C7–C6–C5	178.18(19)
N2–N1–C7–C2	−166.62(19)
N2–N1–C7–C6	14.4(3)
N2–C8–C9–C10	178.2(2)
N2–C8–C12–C11	157.1(2)
C1–C2–C3–C4	178.35(19)
C7–N1–N2–C8	175.0(2)
C7–C2–C3–C4	−0.1(3)
C7–C6–C5–C4	0.2(3)
C2–C7–C6–C5	−0.8(3)
C2–C3–C4–C5	−0.6(3)
C3–C4–C5–C6	0.5(3)
C6–C7–C2–C1	−177.61(19)
C6–C7–C2–C3	0.8(3)
C8–C9–C10–C11	27.5(2)
C9–C8–C12–C11	−20.8(3)
C12–C8–C9–C10	−4.1(3)
C12–C11–C10–C9	−40.7(2)
C10–C11–C12–C8	37.4(2)

Table S9. Atomic coordinates and Ueq [Å2] for compound 5b

Atom	x	y	z	Ueq
O1	0.72468(4)	0.62455(6)	0.22081(4)	0.01843(15)
O2	0.62717(4)	0.47694(6)	0.23854(4)	0.01973(16)
O3	0.63564(4)	0.81022(6)	0.15538(3)	0.01607(15)
O4	0.61109(4)	0.47980(6)	0.39191(4)	0.02205(17)
N1	0.74434(5)	0.89861(7)	0.16089(4)	0.01558(17)
N2	0.51237(5)	0.36617(7)	0.38776(4)	0.01628(17)
C7	0.66926(5)	0.74845(8)	0.29412(5)	0.01472(19)
C2	0.66262(5)	0.74010(9)	0.36201(5)	0.0191(2)
H2A	0.654107	0.670637	0.382524	0.023
C3	0.66880(6)	0.83638(9)	0.39916(5)	0.0243(2)
H3	0.663907	0.833264	0.445697	0.029
C4	0.68205(6)	0.93678(9)	0.36851(5)	0.0249(2)
H4	0.686390	1.002068	0.394285	0.030
C5	0.68911(6)	0.94311(8)	0.30021(5)	0.0200(2)
H5	0.698086	1.012544	0.279817	0.024
C6	0.68306(5)	0.84816(8)	0.26184(5)	0.01487(19)
N7	0.66981(4)	0.64783(6)	0.25530(4)	0.01382(17)
N8	0.61804(4)	0.57281(7)	0.26252(4)	0.01422(17)
C9	0.54628(5)	0.60606(8)	0.28386(4)	0.01383(19)
C10	0.51389(5)	0.69399(8)	0.25154(5)	0.0168(2)
H10	0.540332	0.736567	0.220438	0.020
C11	0.44196(5)	0.71905(8)	0.26533(5)	0.0184(2)
H11	0.419378	0.780776	0.244892	0.022
C12	0.40351(5)	0.65350(8)	0.30901(5)	0.0181(2)
H12	0.353807	0.668220	0.316958	0.022
C13	0.43748(5)	0.56605(8)	0.34132(5)	0.01620(19)
H13	0.410629	0.522450	0.371618	0.019
C14	0.50998(5)	0.54121(8)	0.33014(4)	0.01403(19)
C15	0.68615(5)	0.85125(8)	0.18726(5)	0.01373(19)
C16	0.76113(5)	0.90084(8)	0.08905(5)	0.0160(2)
C17	0.70419(6)	0.96574(9)	0.05062(5)	0.0218(2)
H17A	0.657667	0.927139	0.053242	0.033
H17B	0.719053	0.971720	0.004432	0.033
H17C	0.699300	1.040004	0.069531	0.033
C18	0.54974(5)	0.45816(8)	0.37222(5)	0.01549(19)
C19	0.53696(6)	0.28166(8)	0.43575(5)	0.0194(2)
C20	0.54529(7)	0.33556(10)	0.50350(5)	0.0263(2)
H20A	0.582551	0.393139	0.501370	0.039
H20B	0.559485	0.279306	0.535782	0.039
H20C	0.499185	0.368770	0.516738	0.039
C21	0.76551(6)	0.78209(9)	0.06337(5)	0.0203(2)
H21A	0.805691	0.743574	0.084938	0.031
H21B	0.773507	0.783113	0.015662	0.031
H21C	0.720149	0.743436	0.073045	0.031
C22	0.83453(6)	0.95839(10)	0.08246(5)	0.0240(2)
H22A	0.831333	1.033370	0.100651	0.036
H22B	0.848055	0.962422	0.035857	0.036
H22C	0.871176	0.916201	0.106654	0.036
C23	0.60809(6)	0.23018(10)	0.41286(6)	0.0281(2)
H23A	0.601318	0.196380	0.369424	0.042
H23B	0.623302	0.173372	0.444414	0.042
H23C	0.645297	0.287757	0.410069	0.042
C24	0.47825(7)	0.19268(10)	0.43894(6)	0.0316(3)
H24A	0.432300	0.226602	0.452013	0.047
H24B	0.491956	0.136285	0.471314	0.047
H24C	0.472862	0.158142	0.395549	0.047
H1	0.7771(7)	0.9298(11)	0.1877(7)	0.023(3)
H2	0.4691(8)	0.3592(11)	0.3720(7)	0.024(3)

Ueq is defined as 1/3 of the trace of the orthogonalized Uij tensor

Table S10. Anisotropic displacement parameters [Å2] for compound 2d.
The anisotropic displacement factor exponent takes the form:
−2π2[h2(a*)2U11 + k2(b*)2U22 + … + 2hka*b*U12]

Atom	U11	U22	U33	U23	U13	U12
O1	0.0120(3)	0.0192(3)	0.0241(4)	0.0000(3)	0.0066(3)	0.0019(3)
O2	0.0180(3)	0.0142(3)	0.0270(4)	−0.0040(3)	0.0055(3)	−0.0003(3)
O3	0.0125(3)	0.0196(3)	0.0161(3)	0.0006(3)	−0.0010(3)	−0.0018(3)
O4	0.0156(3)	0.0240(4)	0.0265(4)	0.0057(3)	−0.0058(3)	−0.0036(3)
N1	0.0152(4)	0.0182(4)	0.0134(4)	−0.0016(3)	0.0004(3)	−0.0042(3)
N2	0.0147(4)	0.0177(4)	0.0165(4)	0.0031(3)	−0.0044(3)	−0.0016(3)
C7	0.0114(4)	0.0165(4)	0.0163(4)	−0.0012(4)	−0.0010(3)	−0.0005(3)
C2	0.0195(5)	0.0205(5)	0.0173(5)	0.0029(4)	−0.0003(4)	−0.0016(4)
C3	0.0310(6)	0.0281(5)	0.0139(5)	−0.0008(4)	0.0002(4)	−0.0025(5)
C4	0.0334(6)	0.0221(5)	0.0192(5)	−0.0052(4)	−0.0001(4)	−0.0033(4)
C5	0.0238(5)	0.0172(5)	0.0191(5)	−0.0003(4)	0.0010(4)	−0.0028(4)
C6	0.0119(4)	0.0179(5)	0.0148(4)	0.0007(3)	−0.0003(3)	−0.0005(3)
N7	0.0099(4)	0.0153(4)	0.0162(4)	0.0013(3)	0.0016(3)	0.0008(3)
N8	0.0118(4)	0.0140(4)	0.0168(4)	−0.0003(3)	0.0018(3)	−0.0004(3)
C9	0.0103(4)	0.0167(4)	0.0145(4)	−0.0016(3)	0.0006(3)	−0.0003(3)
C10	0.0160(5)	0.0185(5)	0.0159(4)	0.0023(4)	0.0008(4)	−0.0010(4)
C11	0.0165(5)	0.0192(5)	0.0196(5)	0.0010(4)	−0.0025(4)	0.0027(4)
C12	0.0125(4)	0.0221(5)	0.0198(5)	−0.0031(4)	0.0008(4)	0.0015(4)
C13	0.0144(4)	0.0189(4)	0.0152(4)	−0.0005(4)	0.0021(3)	−0.0021(4)
C14	0.0137(4)	0.0151(4)	0.0133(4)	−0.0011(3)	−0.0005(3)	−0.0010(3)
C15	0.0140(4)	0.0126(4)	0.0145(4)	0.0005(3)	0.0006(3)	0.0017(3)
C16	0.0160(4)	0.0181(4)	0.0141(4)	−0.0006(3)	0.0032(3)	−0.0009(4)
C17	0.0223(5)	0.0243(5)	0.0189(5)	0.0050(4)	0.0044(4)	0.0040(4)
C18	0.0148(4)	0.0176(4)	0.0141(4)	0.0001(3)	0.0002(3)	−0.0005(4)
C19	0.0223(5)	0.0182(5)	0.0178(5)	0.0047(4)	−0.0063(4)	−0.0015(4)
C20	0.0326(6)	0.0291(5)	0.0170(5)	0.0024(4)	−0.0053(4)	−0.0004(5)
C21	0.0202(5)	0.0202(5)	0.0206(5)	−0.0038(4)	0.0035(4)	0.0015(4)
C22	0.0194(5)	0.0309(6)	0.0216(5)	−0.0021(4)	0.0062(4)	−0.0076(4)
C23	0.0313(6)	0.0274(6)	0.0256(5)	0.0033(4)	−0.0060(5)	0.0097(5)
C24	0.0354(6)	0.0261(6)	0.0331(6)	0.0141(5)	−0.0142(5)	−0.0106(5)

Table S11. Bond lengths and angles for compound 2d

Atom–Atom	Length [Å]
O1–N7	1.2647(10)
O2–N8	1.2676(11)
O3–C15	1.2398(12)
O4–C18	1.2311(12)
N1–C15	1.3311(12)
N1–C16	1.4918(11)
N1–H1	0.898(14)
N2–C18	1.3461(13)
N2–C19	1.4829(12)
N2–H2	0.866(14)
C7–C2	1.3877(14)
C7–C6	1.3948(13)
C7–N7	1.4487(12)
C2–H2A	0.9500
C2–C3	1.3909(15)
C3–H3	0.9500
C3–C4	1.3850(15)
C4–H4	0.9500
C4–C5	1.3951(15)
C5–H5	0.9500
C5–C6	1.3910(14)
C6–C15	1.5160(12)
N7–N8	1.3264(11)
N8–C9	1.4527(12)
C9–C10	1.3848(13)
C9–C14	1.3955(13)
C10–H10	0.9500
C10–C11	1.3928(14)
C11–H11	0.9500
C11–C12	1.3854(14)
C12–H12	0.9500
C12–C13	1.3932(14)
C13–H13	0.9500
C13–C14	1.3926(13)
C14–C18	1.5089(13)
C16–C17	1.5272(14)
C16–C21	1.5285(13)
C16–C22	1.5309(13)
C17–H17A	0.9800
C17–H17B	0.9800
C17–H17C	0.9800
C19–C20	1.5297(14)
C19–C23	1.5275(15)
C19–C24	1.5292(15)
C20–H20A	0.9800
C20–H20B	0.9800
C20–H20C	0.9800
C21–H21A	0.9800
C21–H21B	0.9800
C21–H21C	0.9800
C22–H22A	0.9800
C22–H22B	0.9800
C22–H22C	0.9800
C23–H23A	0.9800
C23–H23B	0.9800
C23–H23C	0.9800
C24–H24A	0.9800
C24–H24B	0.9800

C24–H24C	0.9800

Atom–Atom–Atom	Angle [°]
C15–N1–C16	124.70(8)
C15–N1–H1	118.9(9)
C16–N1–H1	116.4(9)
C18–N2–C19	124.39(8)
C18–N2–H2	117.9(9)
C19–N2–H2	117.3(9)
C2–C7–C6	123.07(9)
C2–C7–N7	118.70(9)
C6–C7–N7	117.88(8)
C7–C2–H2A	121.0
C7–C2–C3	118.09(9)
C3–C2–H2A	121.0
C2–C3–H3	119.9
C4–C3–C2	120.20(10)
C4–C3–H3	119.9
C3–C4–H4	119.6
C3–C4–C5	120.75(10)
C5–C4–H4	119.6
C4–C5–H5	119.9
C6–C5–C4	120.26(10)
C6–C5–H5	119.9
C7–C6–C15	119.83(8)
C5–C6–C7	117.62(9)
C5–C6–C15	122.42(9)
O1–N7–C7	119.57(8)
O1–N7–N8	119.24(8)
N8–N7–C7	120.53(8)
O2–N8–N7	119.05(8)
O2–N8–C9	119.27(8)
N7–N8–C9	120.23(8)
C10–C9–N8	117.77(8)
C10–C9–C14	122.79(9)
C14–C9–N8	119.02(8)
C9–C10–H10	120.5
C9–C10–C11	118.99(9)
C11–C10–H10	120.5
C10–C11–H11	120.2
C12–C11–C10	119.65(9)
C12–C11–H11	120.2
C11–C12–H12	119.9
C11–C12–C13	120.22(9)
C13–C12–H12	119.9
C12–C13–H13	119.3
C14–C13–C12	121.39(9)
C14–C13–H13	119.3
C9–C14–C18	121.34(8)
C13–C14–C9	116.87(9)
C13–C14–C18	121.34(8)
O3–C15–N1	124.82(9)
O3–C15–C6	118.91(8)
N1–C15–C6	116.27(8)
N1–C16–C17	111.43(8)
N1–C16–C21	109.13(8)
N1–C16–C22	106.15(8)
C17–C16–C21	110.13(8)
C17–C16–C22	109.49(8)
C21–C16–C22	110.45(8)

C16–C17–H17A	109.5
C16–C17–H17B	109.5
C16–C17–H17C	109.5
H17A–C17–H17B	109.5
H17A–C17–H17C	109.5
H17B–C17–H17C	109.5
O4–C18–N2	124.91(9)
O4–C18–C14	119.46(9)
N2–C18–C14	115.56(8)
N2–C19–C20	109.20(8)
N2–C19–C23	110.15(8)
N2–C19–C24	107.09(8)
C23–C19–C20	111.11(9)
C23–C19–C24	109.77(10)
C24–C19–C20	109.43(9)
C19–C20–H20A	109.5
C19–C20–H20B	109.5
C19–C20–H20C	109.5
H20A–C20–H20B	109.5
H20A–C20–H20C	109.5
H20B–C20–H20C	109.5
C16–C21–H21A	109.5
C16–C21–H21B	109.5
C16–C21–H21C	109.5
H21A–C21–H21B	109.5
H21A–C21–H21C	109.5
H21B–C21–H21C	109.5
C16–C22–H22A	109.5
C16–C22–H22B	109.5
C16–C22–H22C	109.5
H22A–C22–H22B	109.5
H22A–C22–H22C	109.5
H22B–C22–H22C	109.5
C19–C23–H23A	109.5
C19–C23–H23B	109.5
C19–C23–H23C	109.5
H23A–C23–H23B	109.5
H23A–C23–H23C	109.5
H23B–C23–H23C	109.5
C19–C24–H24A	109.5
C19–C24–H24B	109.5
C19–C24–H24C	109.5
H24A–C24–H24B	109.5
H24A–C24–H24C	109.5
H24B–C24–H24C	109.5

Table S12. Torsion angles for compound 2d

Atom–Atom–Atom–Atom	Torsion Angle [°]
O1–N7–N8–O2	4.93(13)
O1–N7–N8–C9	−161.22(8)
O2–N8–C9–C10	−116.85(10)
O2–N8–C9–C14	55.93(12)
C7–C2–C3–C4	−0.75(16)
C7–C6–C15–O3	50.96(13)
C7–C6–C15–N1	−128.84(10)
C7–N7–N8–O2	−165.74(8)
C7–N7–N8–C9	28.11(12)
C2–C7–C6–C5	−1.08(14)
C2–C7–C6–C15	−177.20(9)
C2–C7–N7–O1	−118.84(10)
C2–C7–N7–N8	51.80(12)
C2–C3–C4–C5	0.26(18)
C3–C4–C5–C6	−0.15(17)
C4–C5–C6–C7	0.54(15)
C4–C5–C6–C15	176.54(9)
C5–C6–C15–O3	−124.96(10)
C5–C6–C15–N1	55.24(13)
C6–C7–C2–C3	1.19(15)
C6–C7–N7–O1	54.56(12)
C6–C7–N7–N8	−134.80(9)
N7–C7–C2–C3	174.23(9)
N7–C7–C6–C5	−174.18(9)
N7–C7–C6–C15	9.71(13)
N7–N8–C9–C10	49.28(12)
N7–N8–C9–C14	−137.94(9)
N8–C9–C10–C11	172.22(9)
N8–C9–C14–C13	−170.14(8)
N8–C9–C14–C18	17.48(13)
C9–C10–C11–C12	−2.39(15)
C9–C14–C18–O4	36.27(14)
C9–C14–C18–N2	−146.68(9)
C10–C9–C14–C13	2.26(14)
C10–C9–C14–C18	−170.12(9)
C10–C11–C12–C13	2.97(15)
C11–C12–C13–C14	−0.90(15)
C12–C13–C14–C9	−1.66(14)
C12–C13–C14–C18	170.72(9)
C13–C14–C18–O4	−135.77(10)
C13–C14–C18–N2	41.28(13)
C14–C9–C10–C11	−0.26(15)
C15–N1–C16–C17	63.68(12)
C15–N1–C16–C21	−58.15(12)
C15–N1–C16–C22	−177.19(9)
C16–N1–C15–O3	−5.76(15)
C16–N1–C15–C6	174.03(8)
C18–N2–C19–C20	60.98(13)
C18–N2–C19–C23	−61.30(12)
C18–N2–C19–C24	179.37(10)
C19–N2–C18–O4	5.70(16)
C19–N2–C18–C14	−171.16(9)

Table S13. Atomic coordinates and Ueq [Å2] for compound 4

Atom	x	y	z	Ueq
C1	0.16803(15)	0.2683(4)	0.53064(9)	0.0237(5)
C2	0.10534(18)	0.2507(5)	0.46500(10)	0.0304(5)
H2A	0.031394	0.273698	0.468224	0.036
H2B	0.126998	0.389855	0.435440	0.036
C3	0.12754(19)	-0.0297(5)	0.44220(11)	0.0351(6)
H3A	0.117186	-0.039878	0.394474	0.042
H3B	0.082998	-0.165220	0.459694	0.042
C4	0.24036(16)	-0.0756(5)	0.46866(10)	0.0273(5)
H4A	0.286138	0.007959	0.440107	0.033
H4B	0.255965	-0.271276	0.472757	0.033
C5	0.25308(16)	0.0627(4)	0.53558(10)	0.0235(4)
H5A	0.245806	-0.070134	0.570181	0.028
H5B	0.320874	0.151544	0.544518	0.028
C6	0.19163(15)	0.5986(4)	0.68030(9)	0.0190(4)
C7	0.25328(14)	0.5887(4)	0.74128(9)	0.0182(4)
C8	0.23172(15)	0.7656(4)	0.79065(9)	0.0205(4)
H8	0.272651	0.757995	0.831517	0.025
C9	0.15258(16)	0.9492(4)	0.78096(9)	0.0234(4)
H9	0.139072	1.067868	0.814651	0.028
C10	0.09239(16)	0.9586(4)	0.72083(10)	0.0236(4)
H10	0.037806	1.085428	0.713872	0.028
C11	0.11063(15)	0.7876(4)	0.67142(9)	0.0225(4)
H11	0.068333	0.797075	0.631062	0.027
C12	0.33894(15)	0.3968(4)	0.75290(9)	0.0201(4)
C13	0.47058(16)	0.2281(5)	0.82988(9)	0.0248(5)
H13A	0.531812	0.309651	0.814518	0.030
H13B	0.457845	0.048991	0.809147	0.030
C14	0.48577(15)	0.2008(5)	0.90180(10)	0.0240(4)
N1	0.14590(13)	0.4365(4)	0.57345(8)	0.0245(4)
N2	0.21050(13)	0.4318(4)	0.63098(8)	0.0217(4)
O1	0.38323(11)	0.4005(3)	0.81524(6)	0.0255(3)
O2	0.37064(10)	0.2491(3)	0.71313(6)	0.0235(3)
F1	0.49366(10)	0.4393(3)	0.93175(6)	0.0343(3)
F2	0.40901(10)	0.0675(3)	0.92374(6)	0.0369(4)
F3	0.57215(10)	0.0624(3)	0.92067(6)	0.0367(4)
H2	0.2584(18)	0.313(5)	0.6369(11)	0.024(6)

Ueq is defined as 1/3 of the trace of the orthogonalized Uij tensor.

Table S14. Anisotropic displacement parameters [Å2] for compound 4.
The anisotropic displacement factor exponent takes the form:
−2π2[h2(a*)2U11 + k2(b*)2U22 + … + 2hka*b*U12]

Atom	U11	U22	U33	U23	U13	U12
C1	0.0249(10)	0.0258(12)	0.0207(9)	−0.0020(8)	0.0036(8)	−0.0001(9)
C2	0.0353(12)	0.0344(14)	0.0201(10)	−0.0070(9)	−0.0017(8)	0.0048(10)
C3	0.0404(13)	0.0349(14)	0.0297(11)	−0.0077(10)	0.0033(10)	0.0031(11)
C4	0.0279(11)	0.0292(13)	0.0263(10)	−0.0080(9)	0.0096(8)	−0.0001(9)
C5	0.0245(10)	0.0241(11)	0.0230(10)	−0.0015(8)	0.0072(8)	0.0011(9)
C6	0.0233(9)	0.0176(10)	0.0173(9)	−0.0005(8)	0.0070(7)	−0.0027(8)
C7	0.0217(9)	0.0175(10)	0.0164(9)	0.0008(8)	0.0061(7)	−0.0012(8)
C8	0.0252(10)	0.0209(11)	0.0165(8)	0.0012(8)	0.0064(7)	−0.0021(8)
C9	0.0319(11)	0.0208(11)	0.0190(9)	−0.0013(8)	0.0091(8)	0.0000(9)
C10	0.0264(10)	0.0213(11)	0.0243(10)	0.0005(8)	0.0079(8)	0.0037(8)
C11	0.0251(10)	0.0238(11)	0.0184(9)	0.0008(8)	0.0023(7)	0.0019(8)
C12	0.0228(9)	0.0227(11)	0.0155(8)	0.0013(8)	0.0056(7)	−0.0028(8)
C13	0.0247(10)	0.0305(12)	0.0196(9)	0.0008(9)	0.0041(8)	0.0088(9)
C14	0.0229(10)	0.0267(12)	0.0225(9)	0.0002(9)	0.0028(8)	0.0041(9)
N1	0.0271(9)	0.0284(10)	0.0172(8)	−0.0043(7)	−0.0001(7)	0.0034(8)
N2	0.0255(9)	0.0237(10)	0.0155(8)	−0.0034(7)	0.0010(6)	0.0047(8)
O1	0.0282(7)	0.0310(9)	0.0166(7)	−0.0021(6)	0.0001(5)	0.0095(7)
O2	0.0258(7)	0.0265(8)	0.0191(7)	−0.0014(6)	0.0064(5)	0.0038(6)
F1	0.0334(7)	0.0387(8)	0.0290(6)	−0.0118(6)	−0.0025(5)	0.0031(6)
F2	0.0359(7)	0.0419(9)	0.0354(7)	0.0116(6)	0.0134(6)	−0.0007(6)
F3	0.0327(7)	0.0470(9)	0.0290(7)	0.0042(6)	−0.0010(5)	0.0162(6)

Table S15. Bond lengths and angles for compound 4

Atom–Atom	Length [Å]
C1–C2	1.510(3)
C1–C5	1.505(3)
C1–N1	1.281(3)
C2–H2A	0.9900
C2–H2B	0.9900
C2–C3	1.506(3)
C3–H3A	0.9900
C3–H3B	0.9900
C3–C4	1.533(3)
C4–H4A	0.9900
C4–H4B	0.9900
C4–C5	1.546(3)
C5–H5A	0.9900
C5–H5B	0.9900
C6–C7	1.421(3)
C6–C11	1.411(3)
C6–N2	1.366(2)
C7–C8	1.408(3)
C7–C12	1.469(3)
C8–H8	0.9500
C8–C9	1.376(3)
C9–H9	0.9500
C9–C10	1.397(3)
C10–H10	0.9500
C10–C11	1.378(3)
C11–H11	0.9500
C12–O1	1.357(2)
C12–O2	1.218(2)
C13–H13A	0.9900
C13–H13B	0.9900
C13–C14	1.496(3)
C13–O1	1.432(2)
C14–F1	1.332(3)
C14–F2	1.334(2)
C14–F3	1.341(2)
N1–N2	1.381(2)
N2–H2	0.86(2)

Atom–Atom–Atom	Angle [°]
C5–C1–C2	109.79(17)
N1–C1–C2	121.65(19)
N1–C1–C5	128.55(18)
C1–C2–H2A	111.0
C1–C2–H2B	111.0
H2A–C2–H2B	109.0
C3–C2–C1	103.57(18)
C3–C2–H2A	111.0
C3–C2–H2B	111.0
C2–C3–H3A	111.0
C2–C3–H3B	111.0
C2–C3–C4	103.89(19)
H3A–C3–H3B	109.0
C4–C3–H3A	111.0
C4–C3–H3B	111.0
C3–C4–H4A	110.9
C3–C4–H4B	110.9
C3–C4–C5	104.40(17)
H4A–C4–H4B	108.9

C5–C4–H4A	110.9
C5–C4–H4B	110.9
C1–C5–C4	104.05(17)
C1–C5–H5A	110.9
C1–C5–H5B	110.9
C4–C5–H5A	110.9
C4–C5–H5B	110.9
H5A–C5–H5B	109.0
C11–C6–C7	118.40(17)
N2–C6–C7	121.15(18)
N2–C6–C11	120.45(18)
C6–C7–C12	120.56(17)
C8–C7–C6	119.34(18)
C8–C7–C12	120.10(17)
C7–C8–H8	119.3
C9–C8–C7	121.38(18)
C9–C8–H8	119.3
C8–C9–H9	120.5
C8–C9–C10	119.04(19)
C10–C9–H9	120.5
C9–C10–H10	119.3
C11–C10–C9	121.3(2)
C11–C10–H10	119.3
C6–C11–H11	119.7
C10–C11–C6	120.52(18)
C10–C11–H11	119.7
O1–C12–C7	112.10(16)
O2–C12–C7	126.57(17)
O2–C12–O1	121.32(18)
H13A–C13–H13B	108.8
C14–C13–H13A	110.6
C14–C13–H13B	110.6
O1–C13–H13A	110.6
O1–C13–H13B	110.6
O1–C13–C14	105.60(15)
F1–C14–C13	112.59(19)
F1–C14–F2	106.99(16)
F1–C14–F3	107.51(17)
F2–C14–C13	112.29(18)
F2–C14–F3	107.29(18)
F3–C14–C13	109.91(16)
C1–N1–N2	115.29(17)
C6–N2–N1	119.68(17)
C6–N2–H2	120.3(16)
N1–N2–H2	119.7(16)
C12–O1–C13	115.56(15)

Table S16. Torsion angles for compound 4

Atom–Atom–Atom–Atom	Torsion Angle [°]
C1–C2–C3–C4	−35.7(2)
C1–N1–N2–C6	−178.23(19)
C2–C1–C5–C4	1.5(2)
C2–C1–N1–N2	−179.40(19)
C2–C3–C4–C5	37.1(2)
C3–C4–C5–C1	−23.5(2)
C5–C1–C2–C3	21.4(2)
C5–C1–N1–N2	1.8(3)
C6–C7–C8–C9	−0.5(3)
C6–C7–C12–O1	−174.70(17)
C6–C7–C12–O2	6.1(3)
C7–C6–C11–C10	0.2(3)
C7–C6–N2–N1	176.60(18)
C7–C8–C9–C10	0.3(3)
C7–C12–O1–C13	−177.17(17)
C8–C7–C12–O1	5.5(3)
C8–C7–C12–O2	−173.7(2)
C8–C9–C10–C11	0.2(3)
C9–C10–C11–C6	−0.4(3)
C11–C6–C7–C8	0.2(3)
C11–C6–C7–C12	−179.62(18)
C11–C6–N2–N1	−4.1(3)
C12–C7–C8–C9	179.37(18)
C14–C13–O1–C12	−163.29(17)
N1–C1–C2–C3	−157.6(2)
N1–C1–C5–C4	−179.6(2)
N2–C6–C7–C8	179.52(18)
N2–C6–C7–C12	−0.3(3)
N2–C6–C11–C10	−179.09(19)
O1–C13–C14–F1	−54.4(2)
O1–C13–C14–F2	66.4(2)
O1–C13–C14–F3	−174.25(17)
O2–C12–O1–C13	2.1(3)

References

(1) Zeng, D. Y., Tianbao; Tang, Niu; Deng, Wei; Xiang, Jiannan; Yin, Shuang-Feng; Kambe, Nobuaki; Qiu, Renhua. UV-Light-Induced Dehydrogenative *N*-Acylation of Amines with 2-Nitrobenzaldehydes To Give 2-Aminobenzamides. *Synthesis* **2022**, *54*, 2361–2372. (ref. 15 of main manuscript)

(2) Nykaza, T. V.; Harrison, T. S.; Ghosh, A.; Putnik, R. A.; Radosevich, A. T. A Biphilic Phosphetane Catalyzes N–N Bond-Forming Cadogan Heterocyclization via PIII/PV═O Redox Cycling. *J. Am. Chem. Soc.* **2017**, *139*, 6839–6842.

(3) CrysAlisPRO.Oxford Diffraction /Agilent Technologies, **2022**, 1.171.42.75 (Rigaku OD).

(4) Sheldrick, G. M. SHELXT - Integrated space-group and crystal-structure determination *Acta Cryst.* **2015**, *A71*, 3–8.

(5) Hübschle, C.B.; Sheldrick, G.M.; Dittrich, B. ShelXle. A Qt Graphical User Interface for SHELXL, ShelXle: A Qt Graphical User Interface for SHELXL. *J. Appl. Crystallogr.* **2011**, *44*, 1281-1284.

(6) Dolomanov, O. V.; Bourhis, L. J.; Gildea, R. J.; Howard, J. A. K.; Puschmann, H. *J. Appl. Cryst,* **2009**, *42*, 339–341.

(7) MacRae, C. F.; Sovago, I; Cottrell, S. J.; Galek, P. T. A.; McCabe, P.; Pidcock, E.; Platings, M.; Shields, G. P.; Stevens, J. S.; Towler, M.; Wood, P. A. *J. Appl. Crystallogr.*, **2020**, *53*, 226–235.

(8) Groom, C. R.; Bruno, I. J.; Lightfoot, M. P.; Ward, S. C. *Acta Cryst*. **2016**, *B72*, 171–179.

Pictures of the flow equipment and a nitroso in solution/solid state

- Representative example of the flow set-up during **1b** long run.

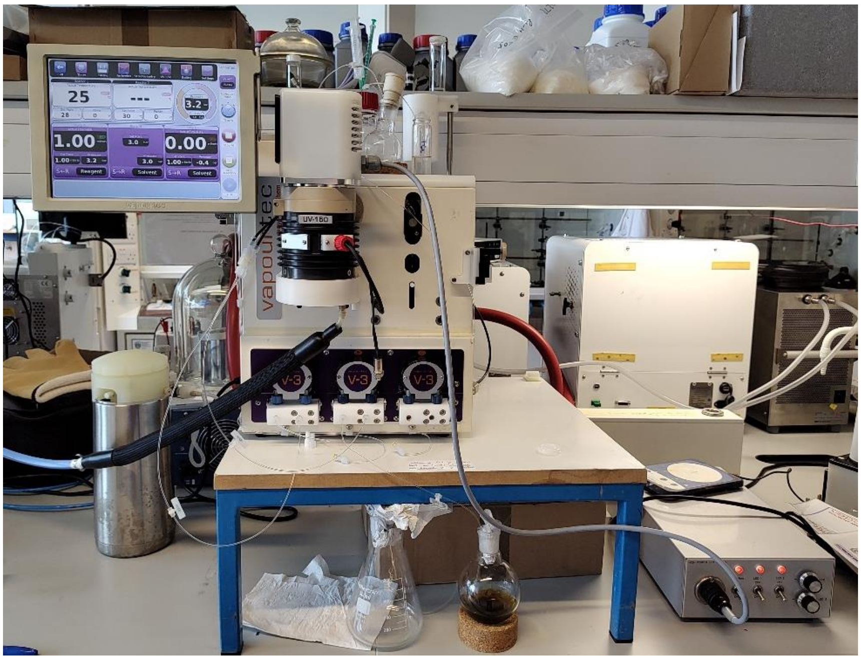

- 10 mL PFA reactor coil and 365 nm High power LEDs

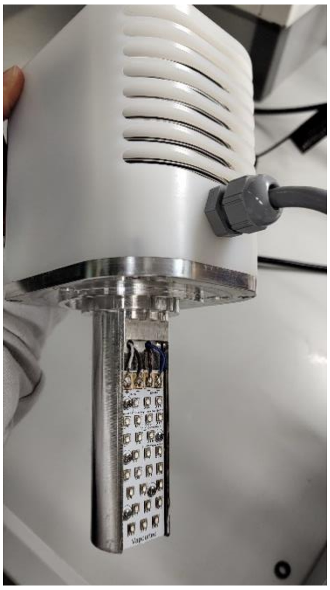

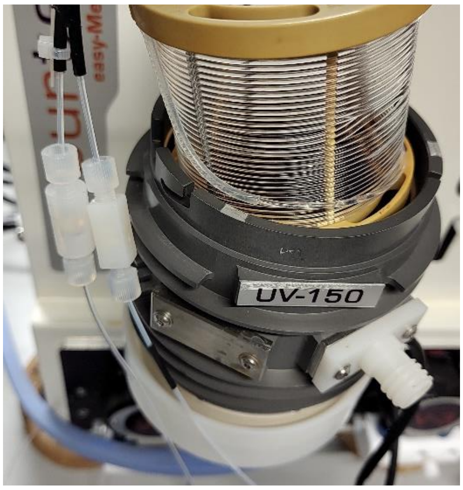

Pure **2b** sample in solution

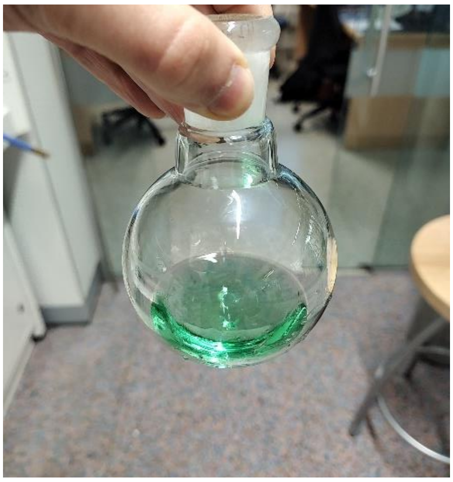

Pure **2b** sample in solid state

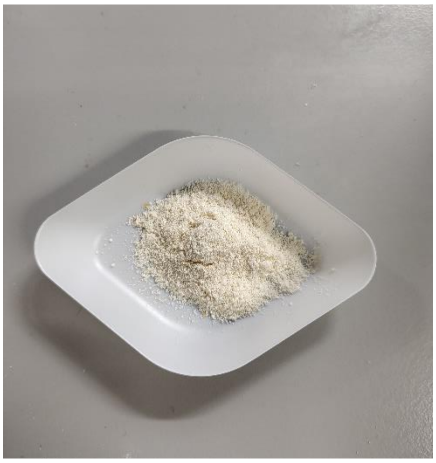

Copies of NMR data

NMR copies of nitrosoarenes: **2a-2o**

2a: *N*-isobutyl-2-nitrosobenzamide

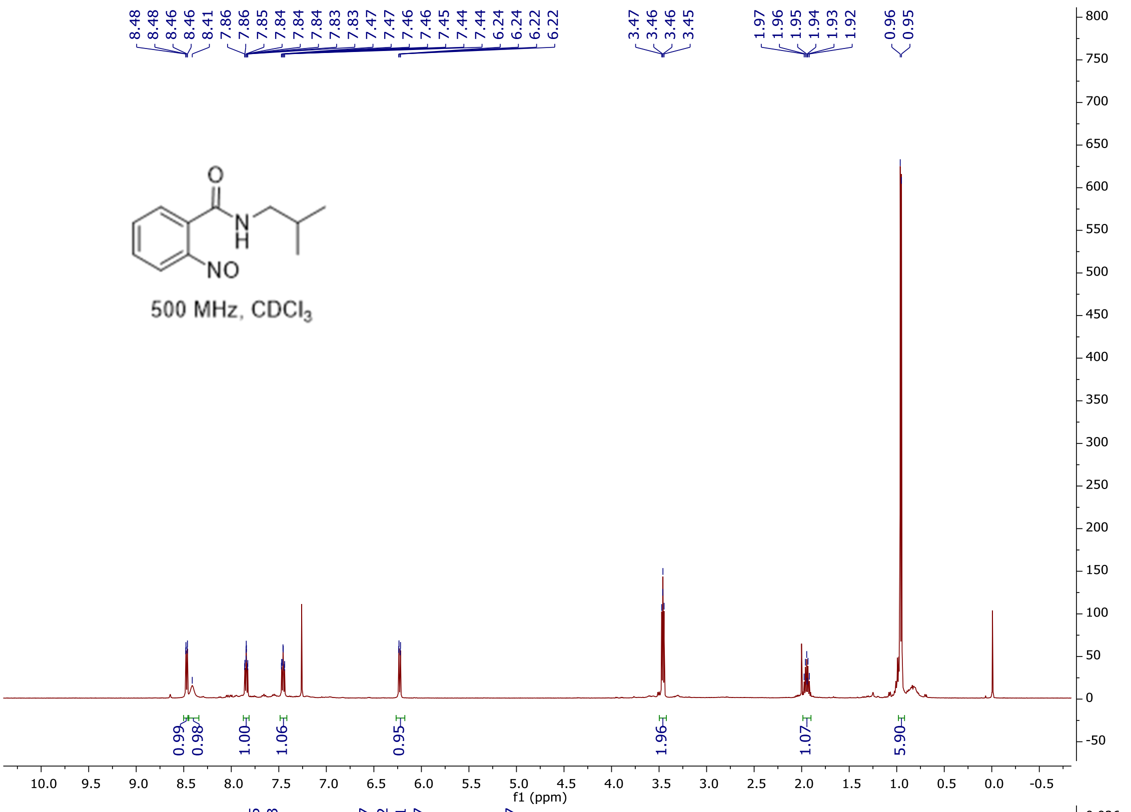

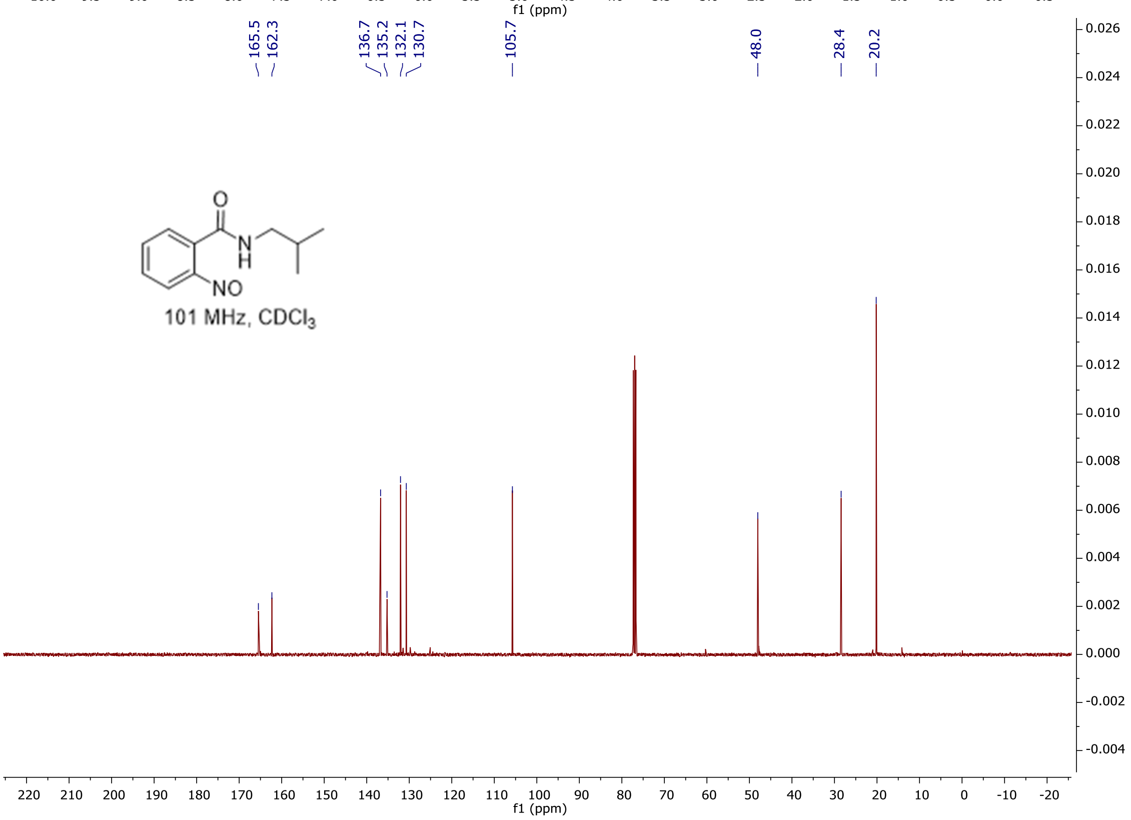

2b: *N*-cyclopentyl-2-nitrosobenzamide

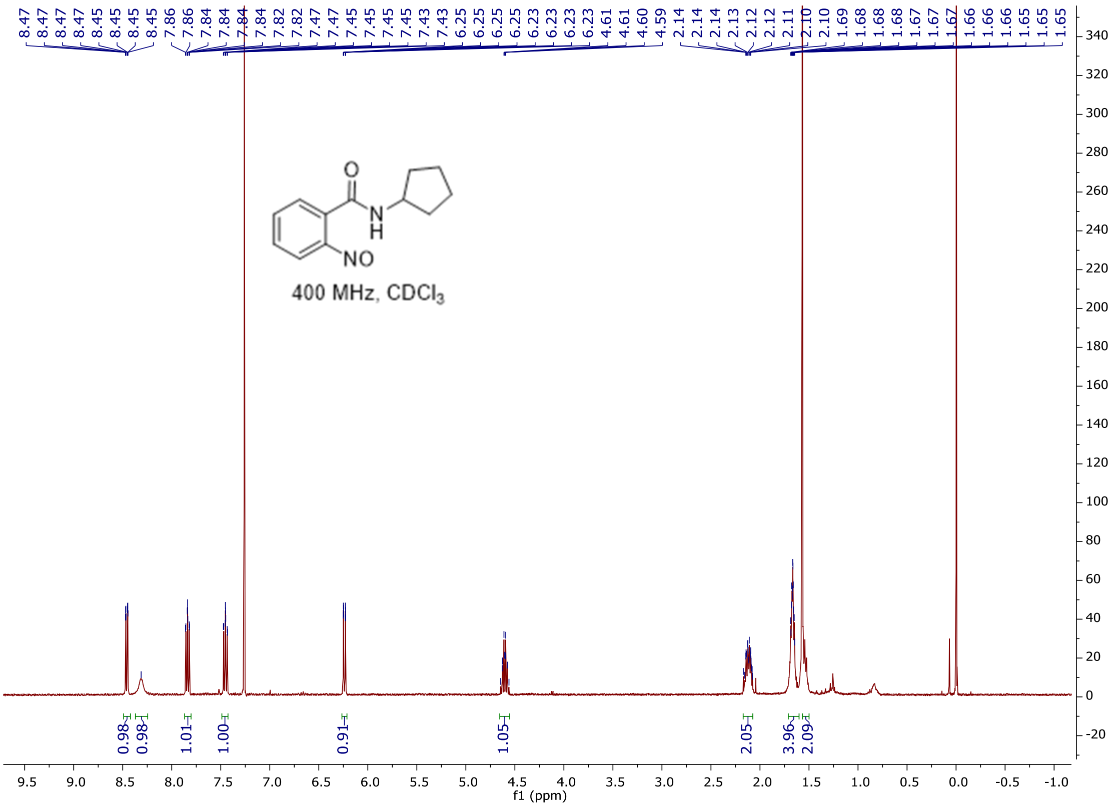

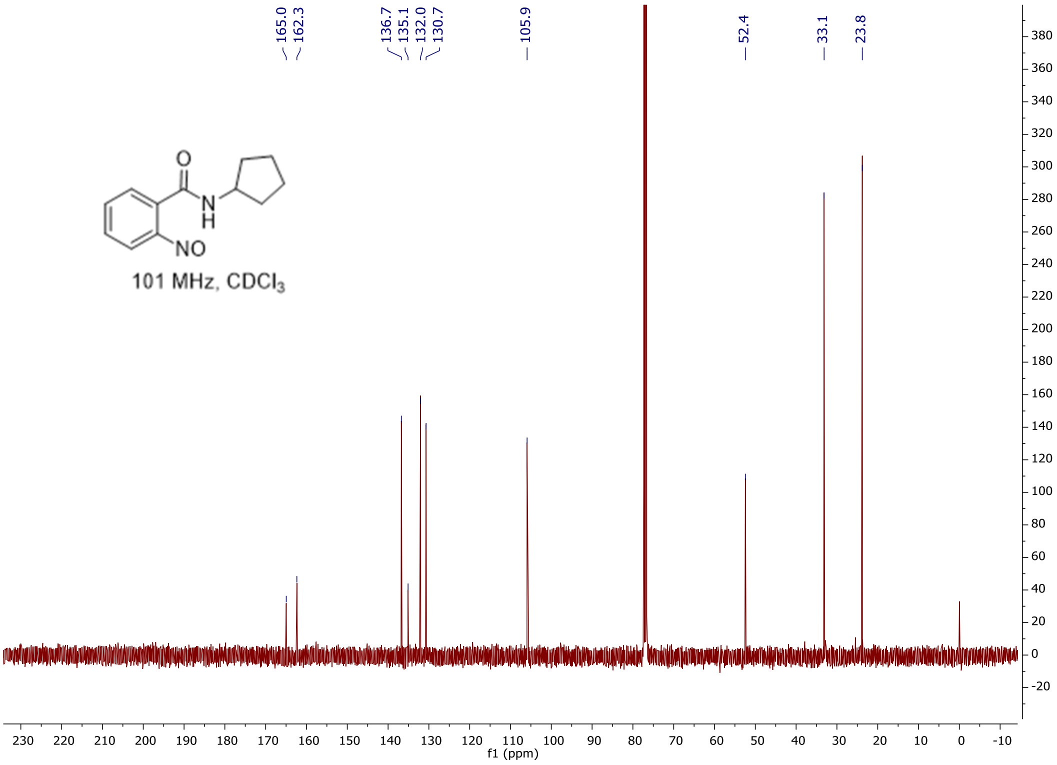

2c: *N*-cyclohexyl-2-nitrosobenzamide

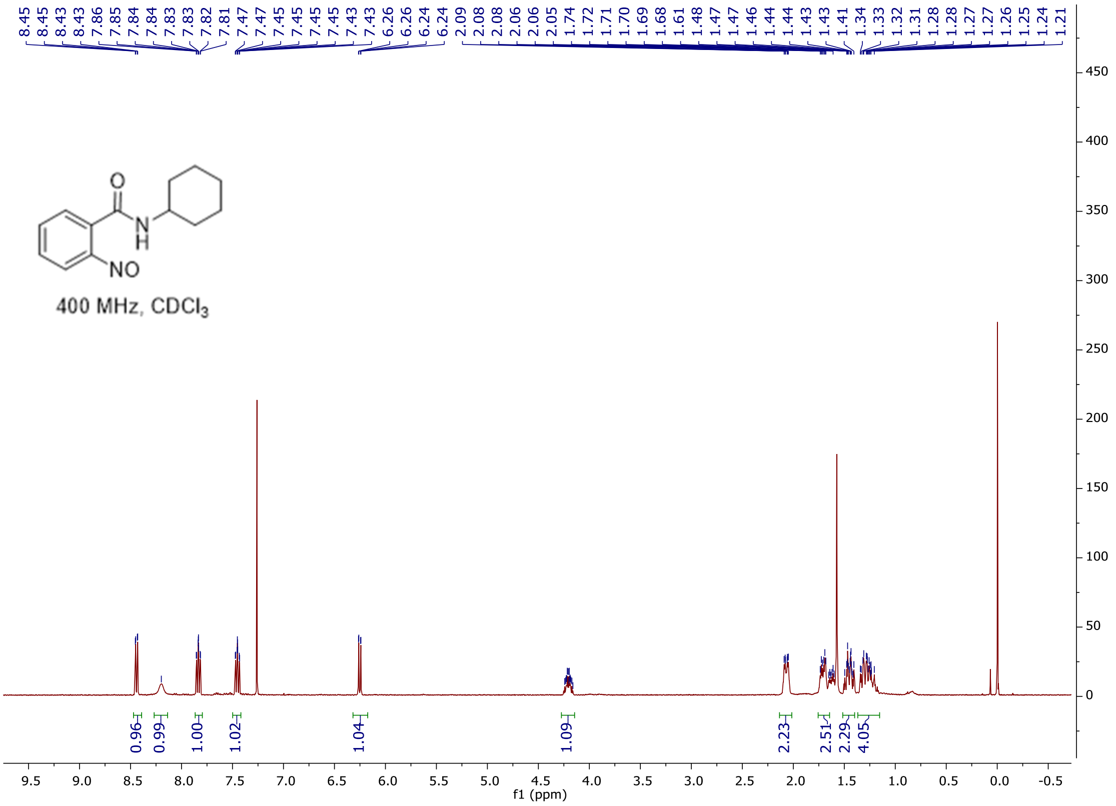

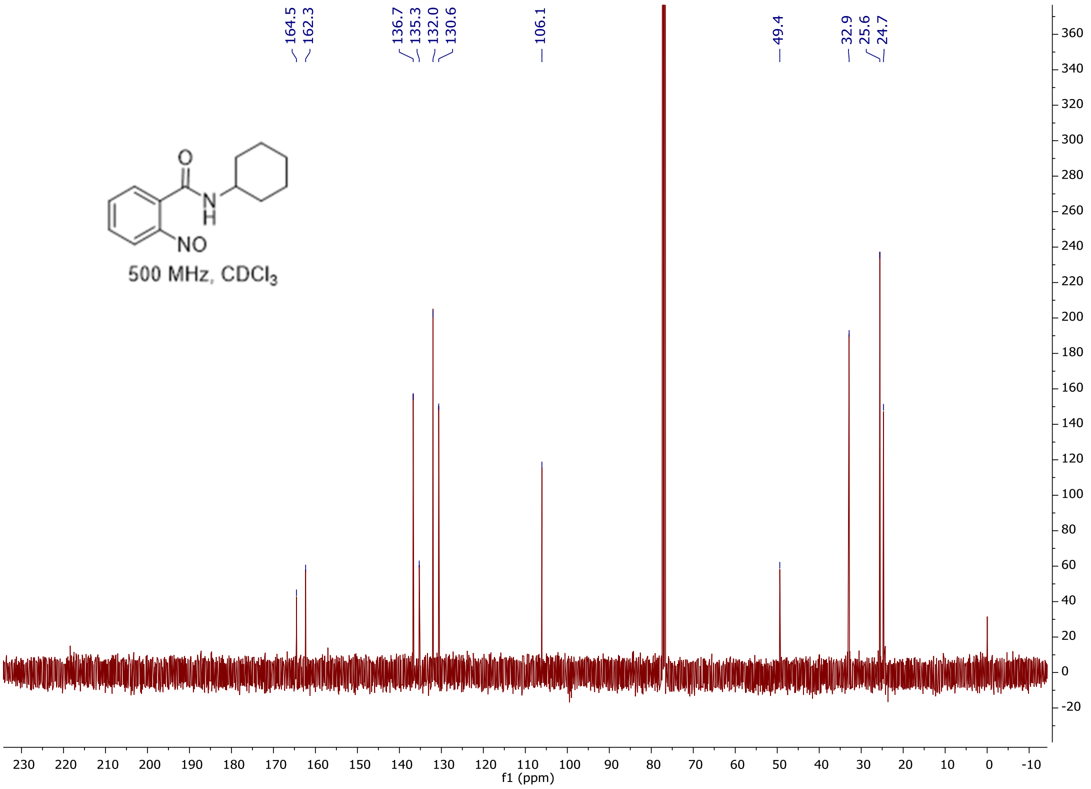

2d: *N*-(tert-butyl)-2-nitrosobenzamide

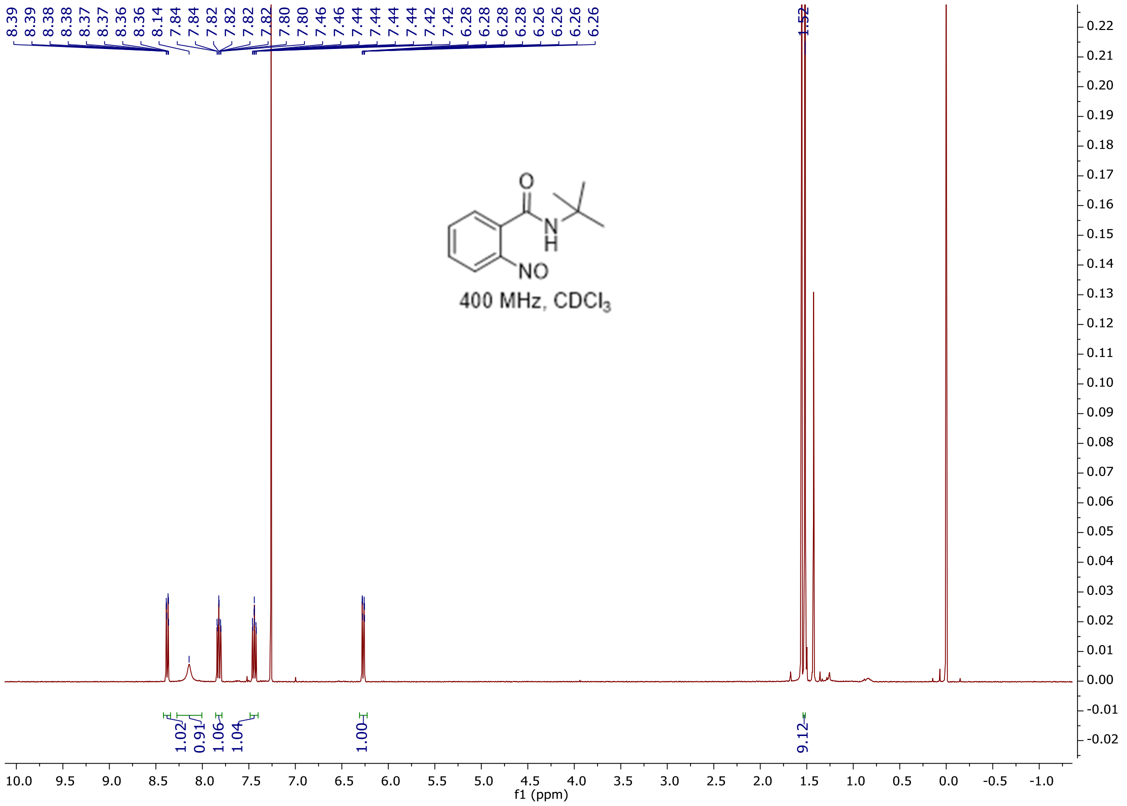

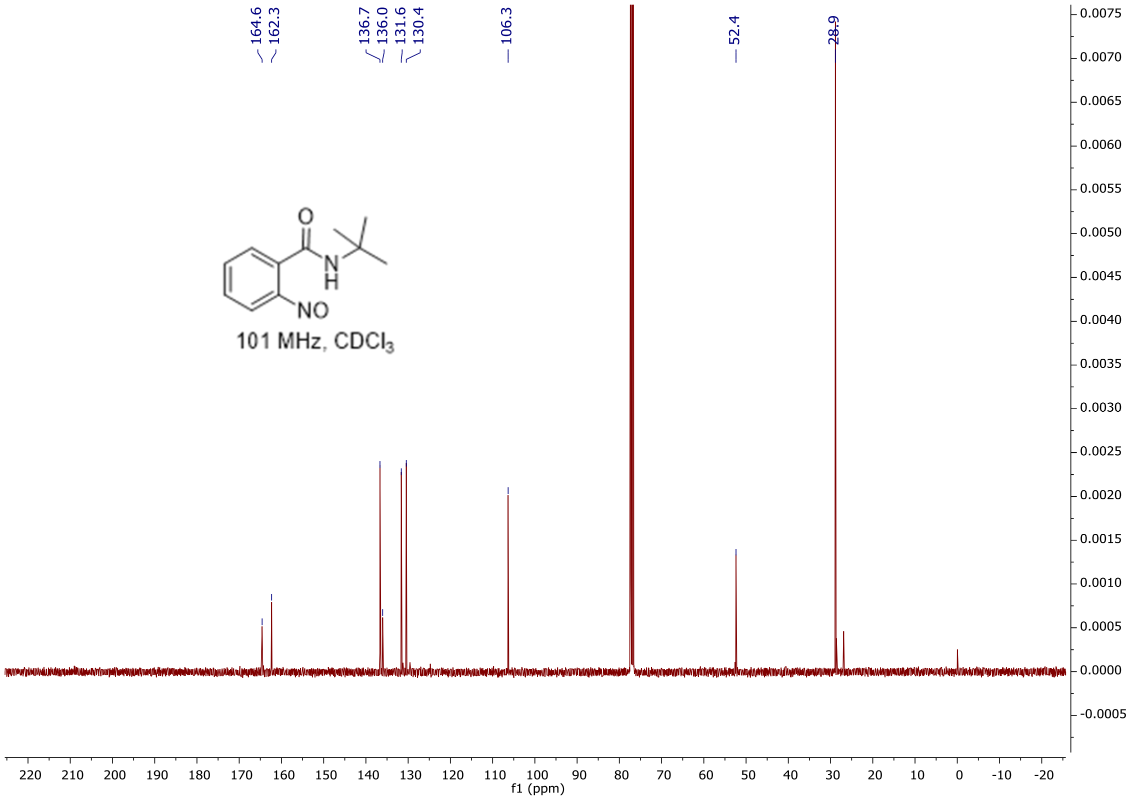

2e: *N*-decyl-2-nitrosobenzamide

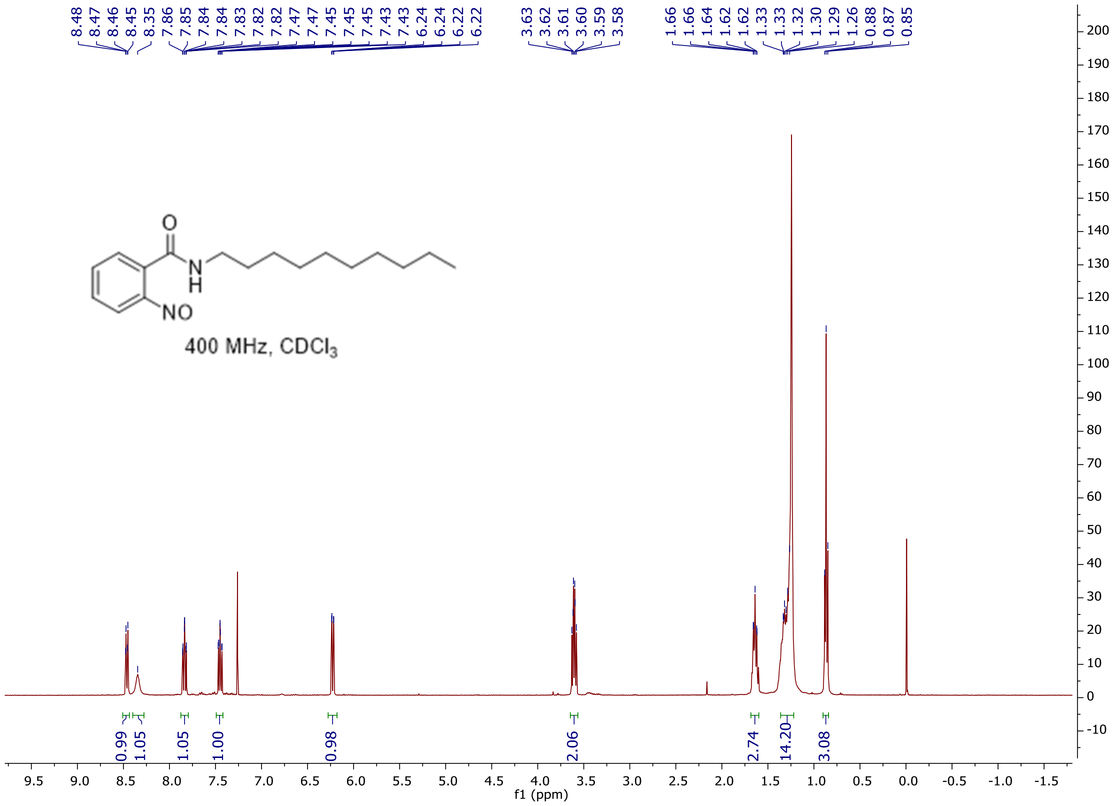

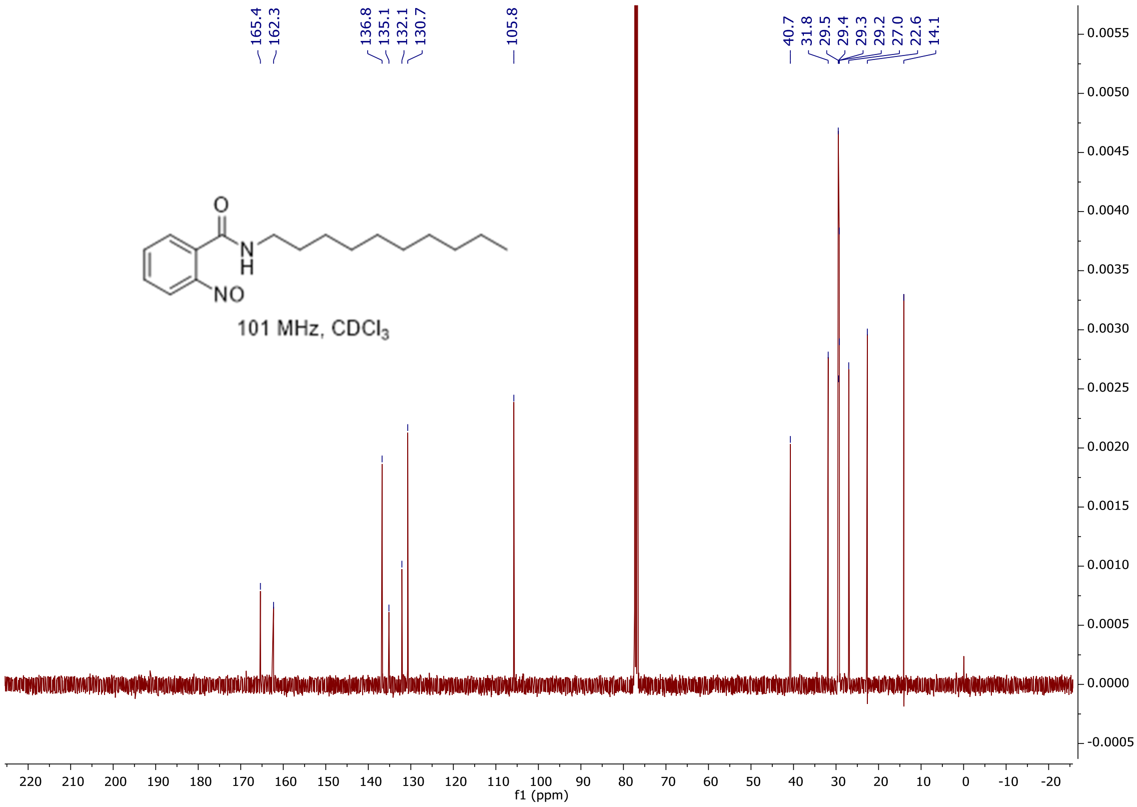

2f: *N*-(2-methoxybenzyl)-2-nitrosobenzamide

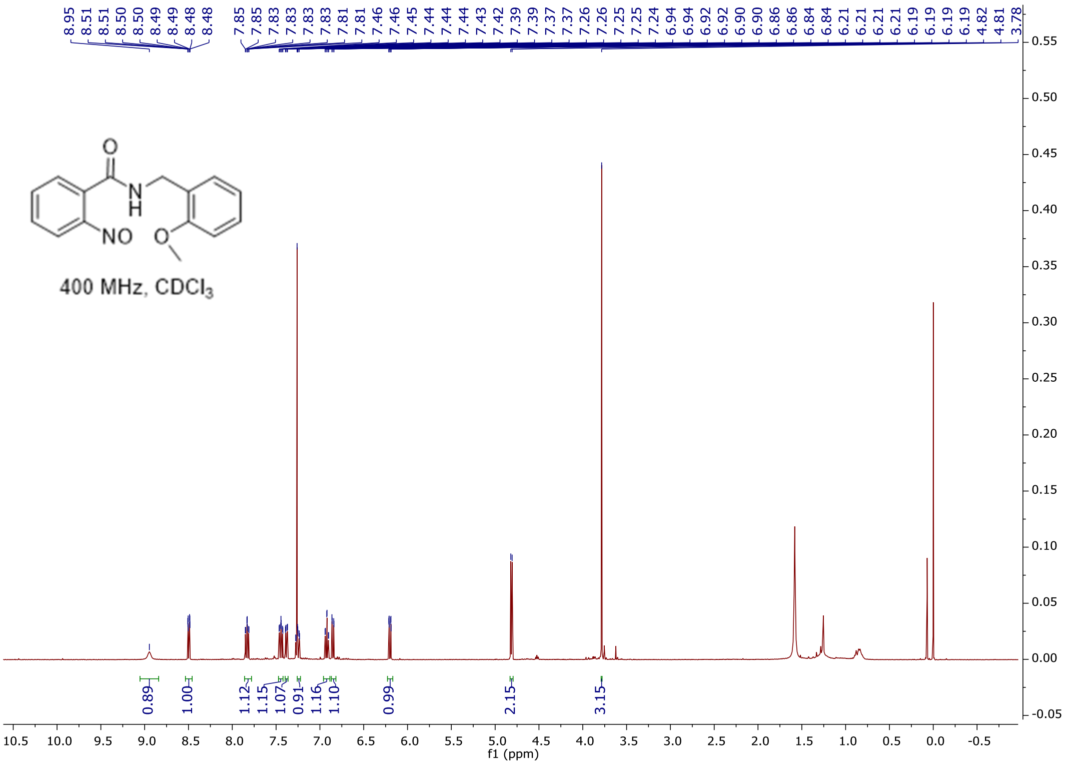

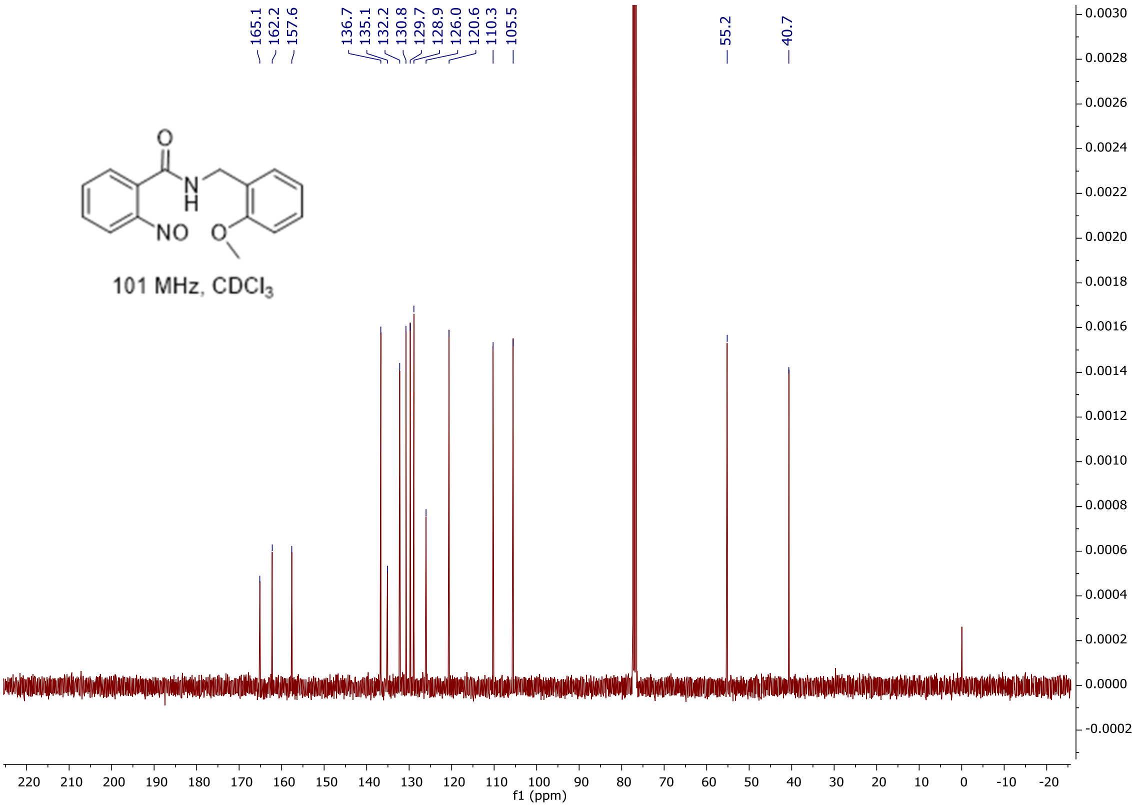

2g: *N*-(4-fluorobenzyl)-2-nitrosobenzamide

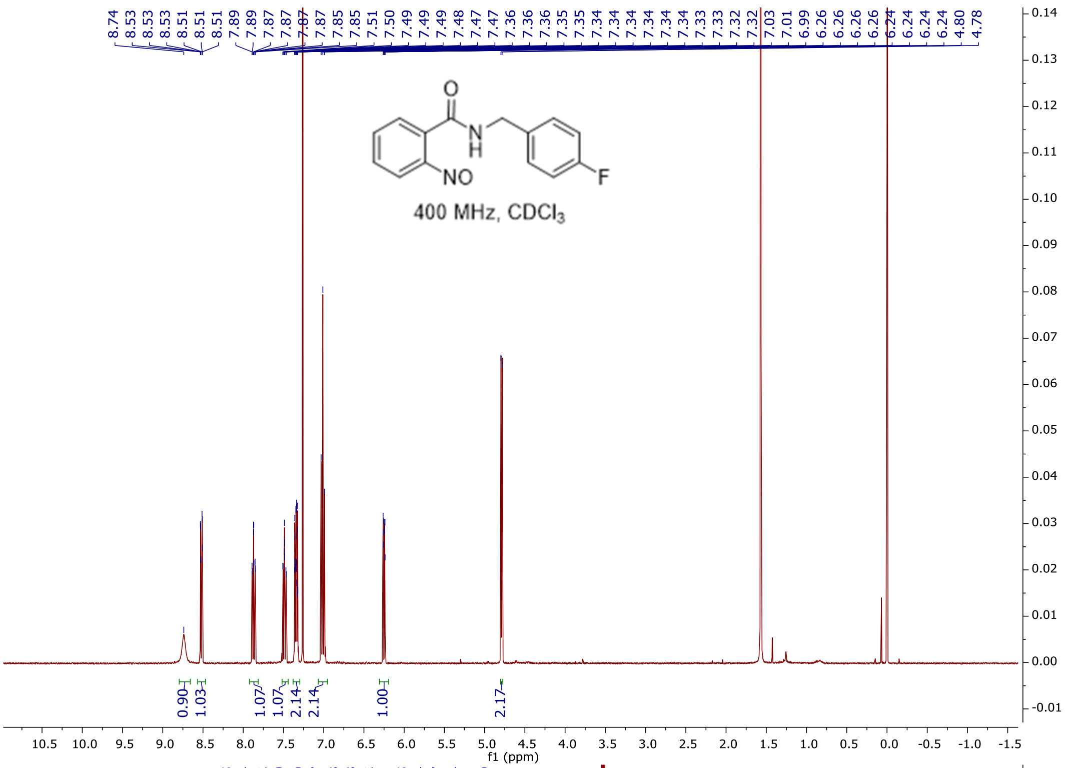

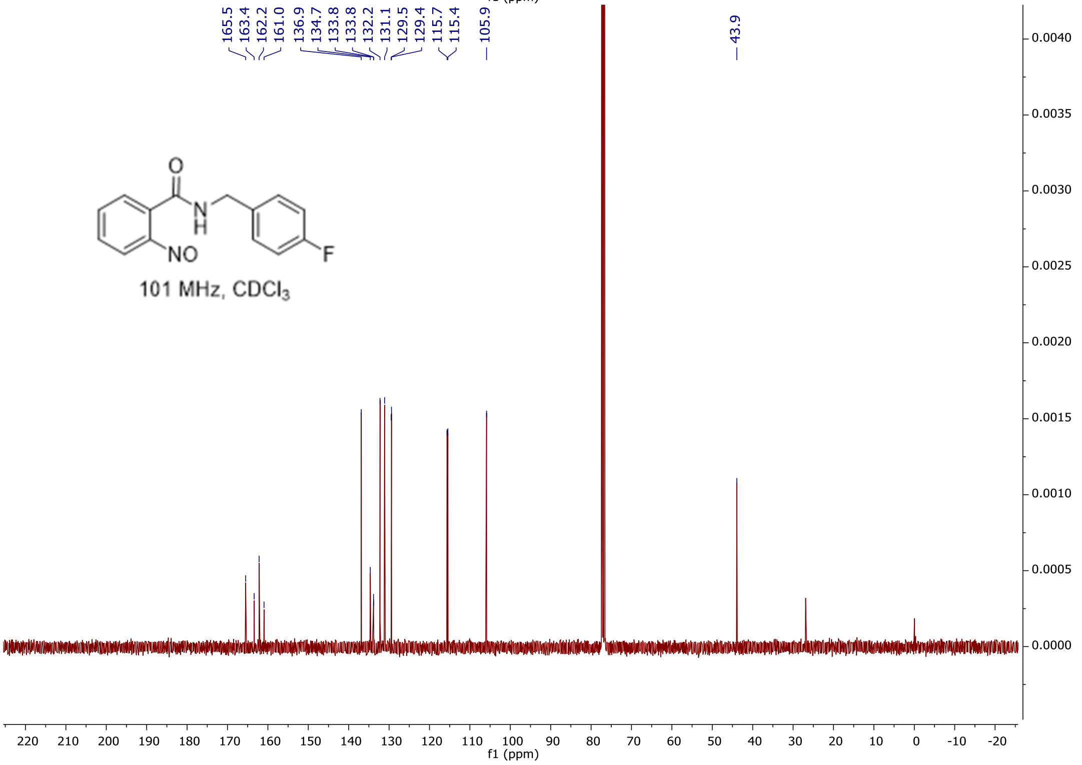

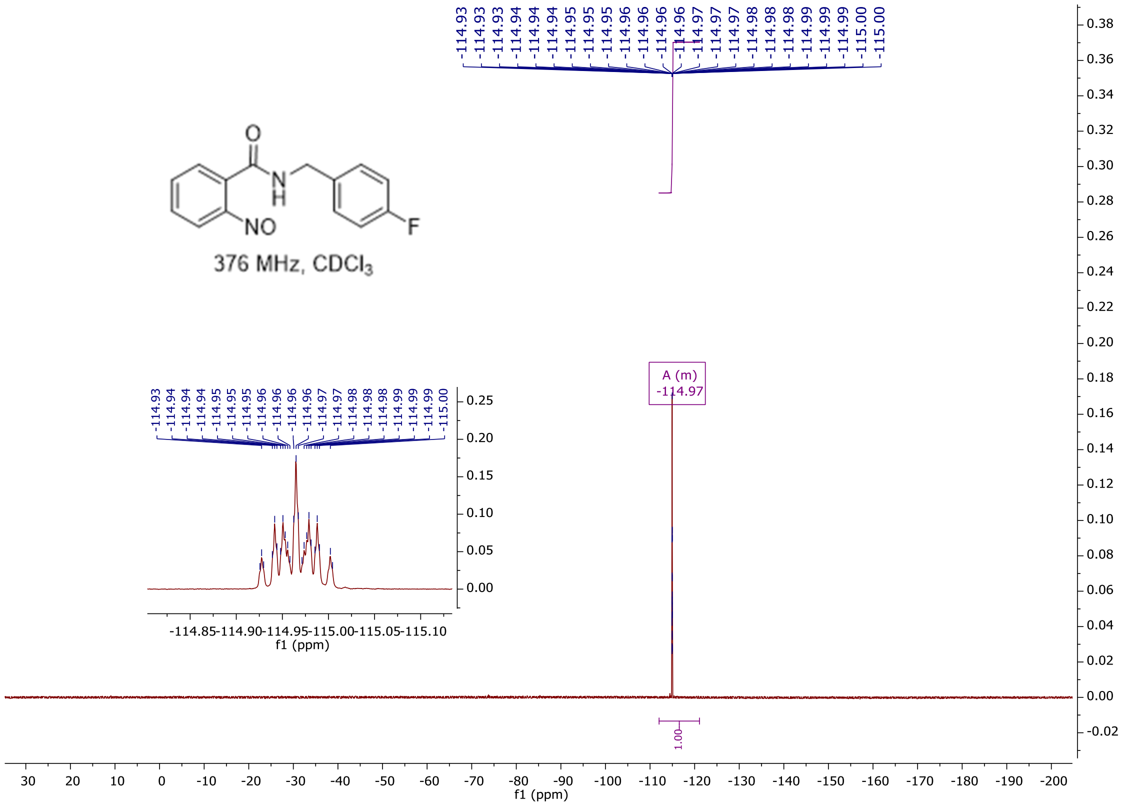
376 MHz, CDCl3
A (m)
-114.97
1.00
f1 (ppm)

2h: methyl (2-nitrosobenzoyl)phenylalaninate

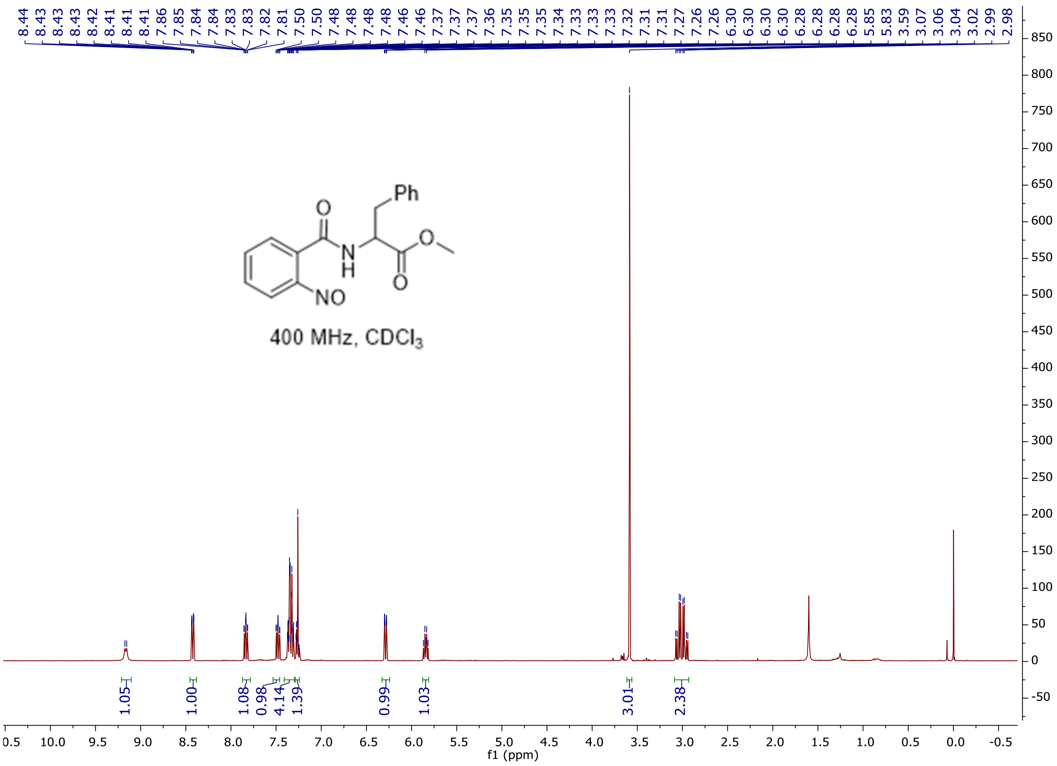

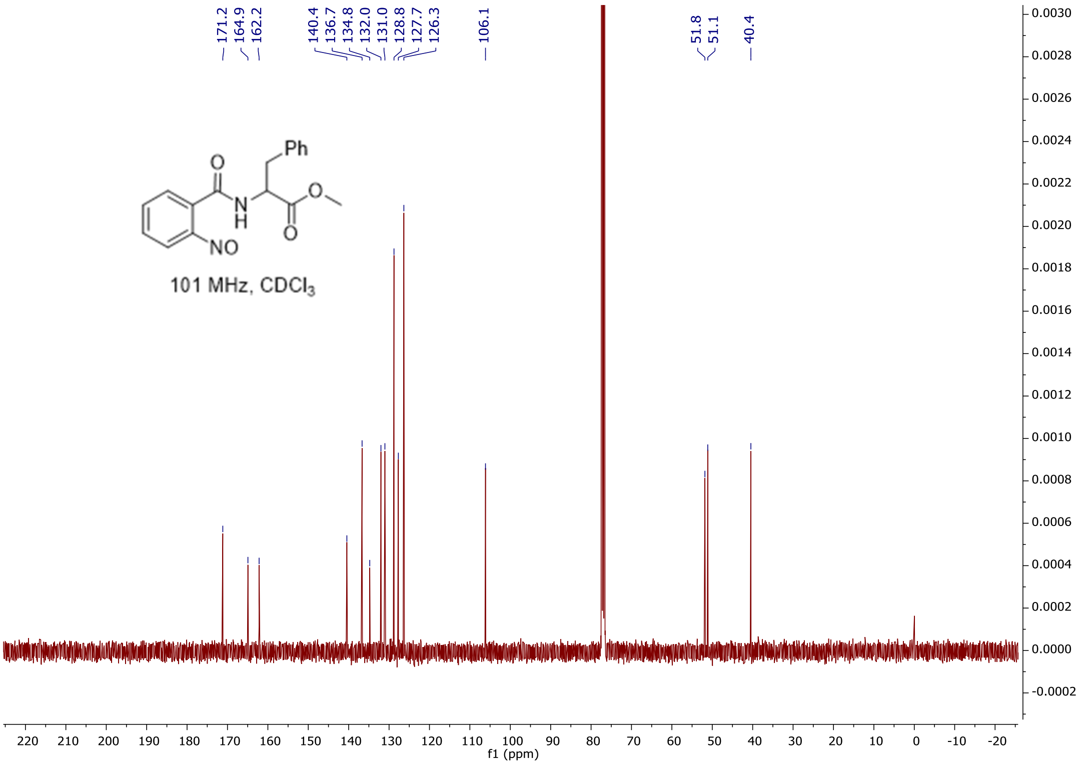

2i: 2-nitroso-*N*-phenylbenzamide

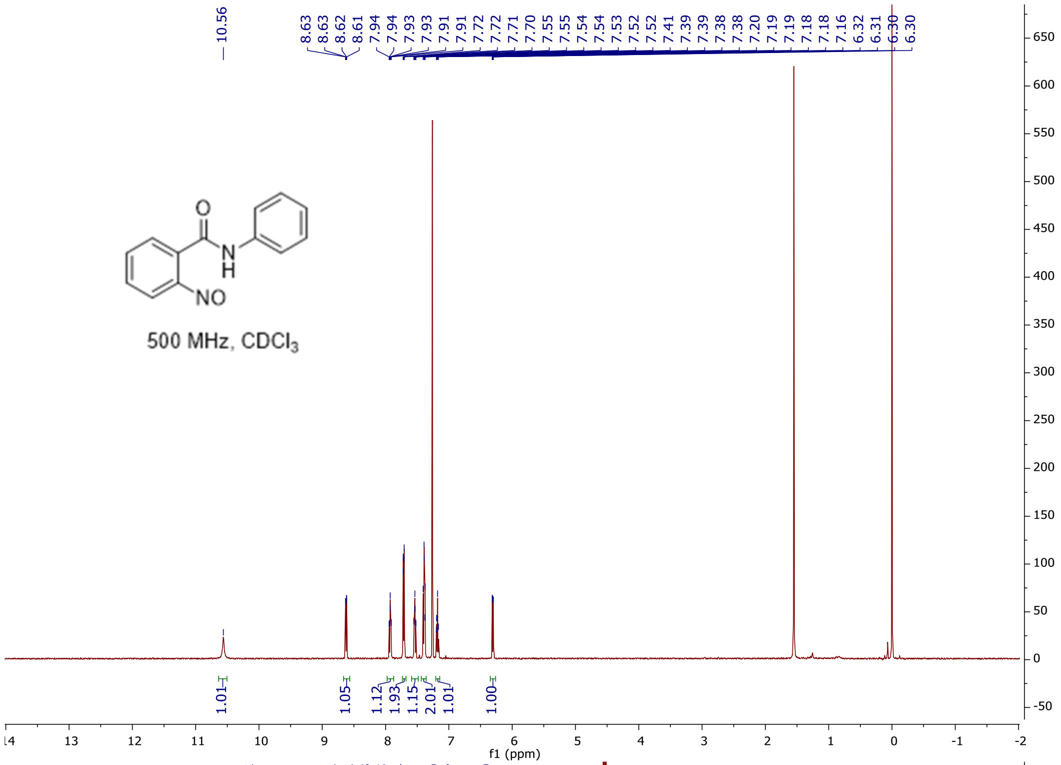

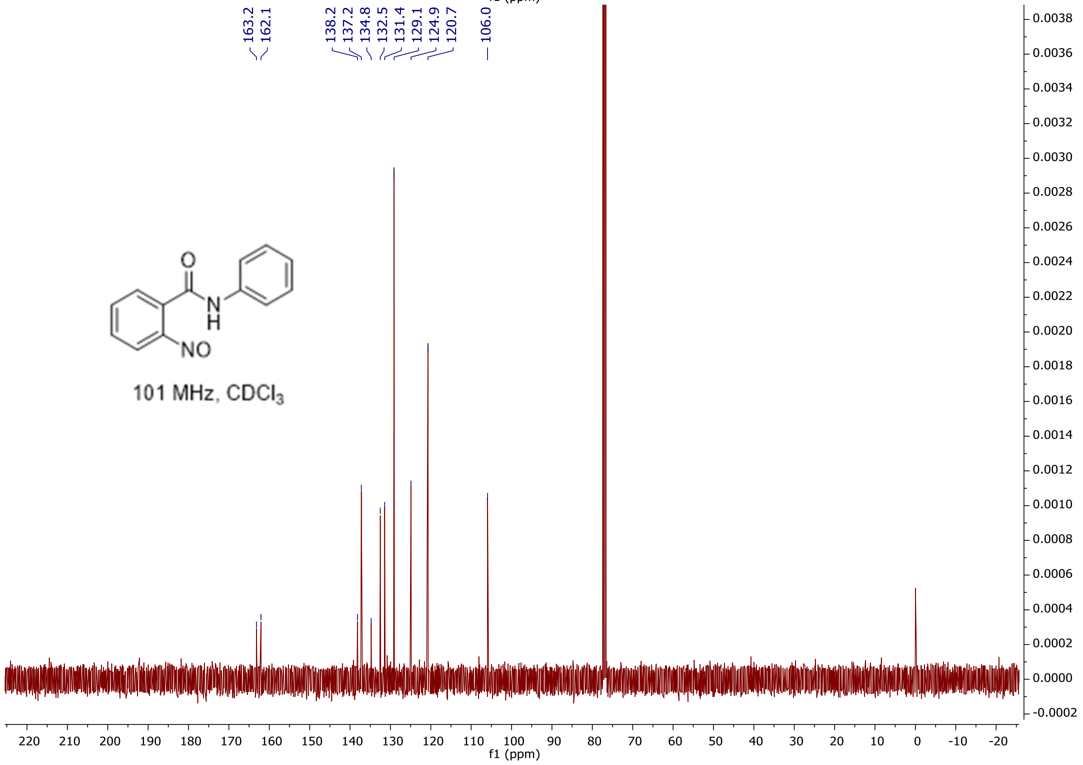

2j: *N*-(4-fluorophenyl)-2-nitrosobenzamide

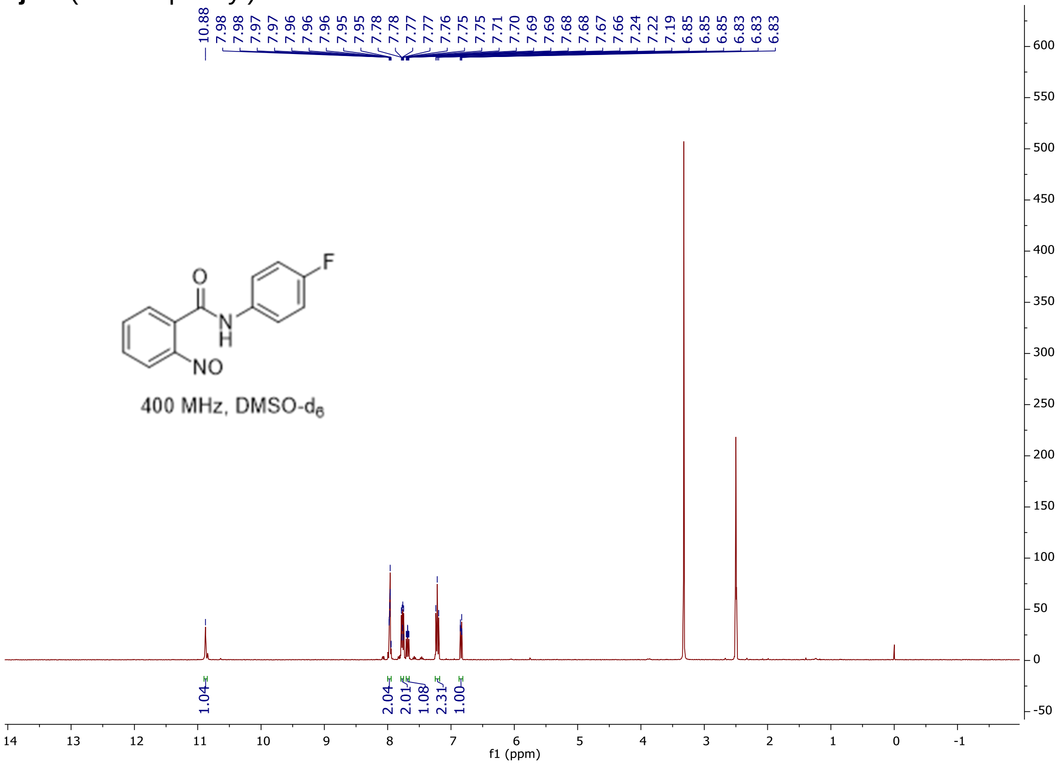

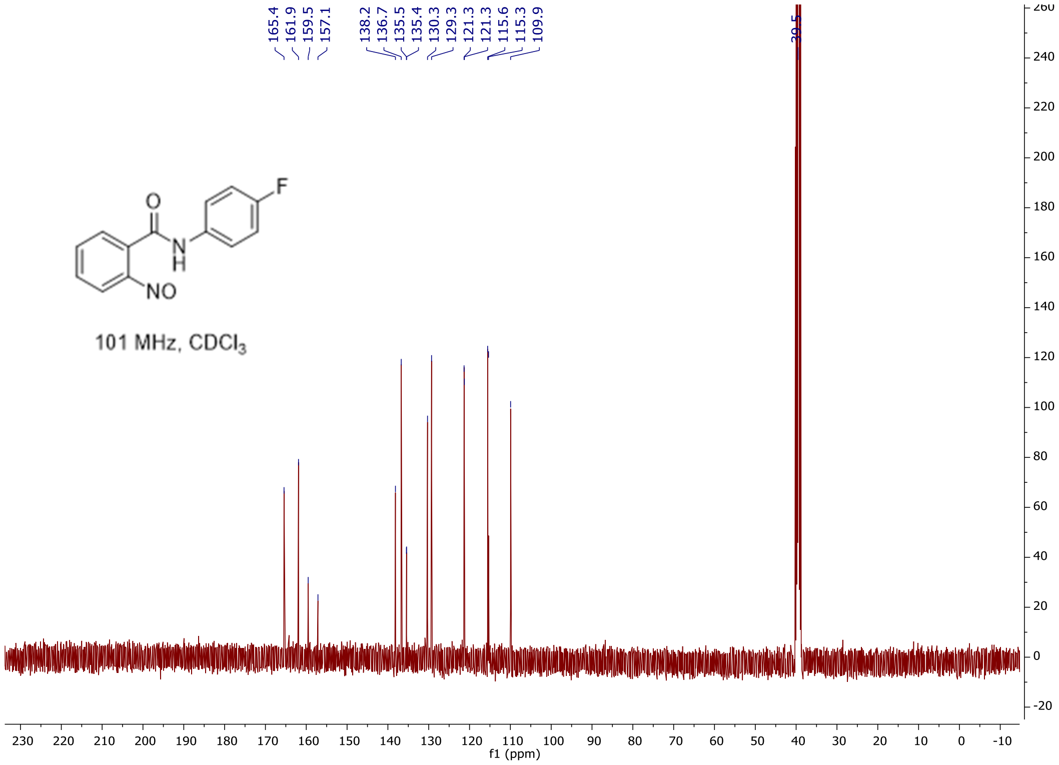

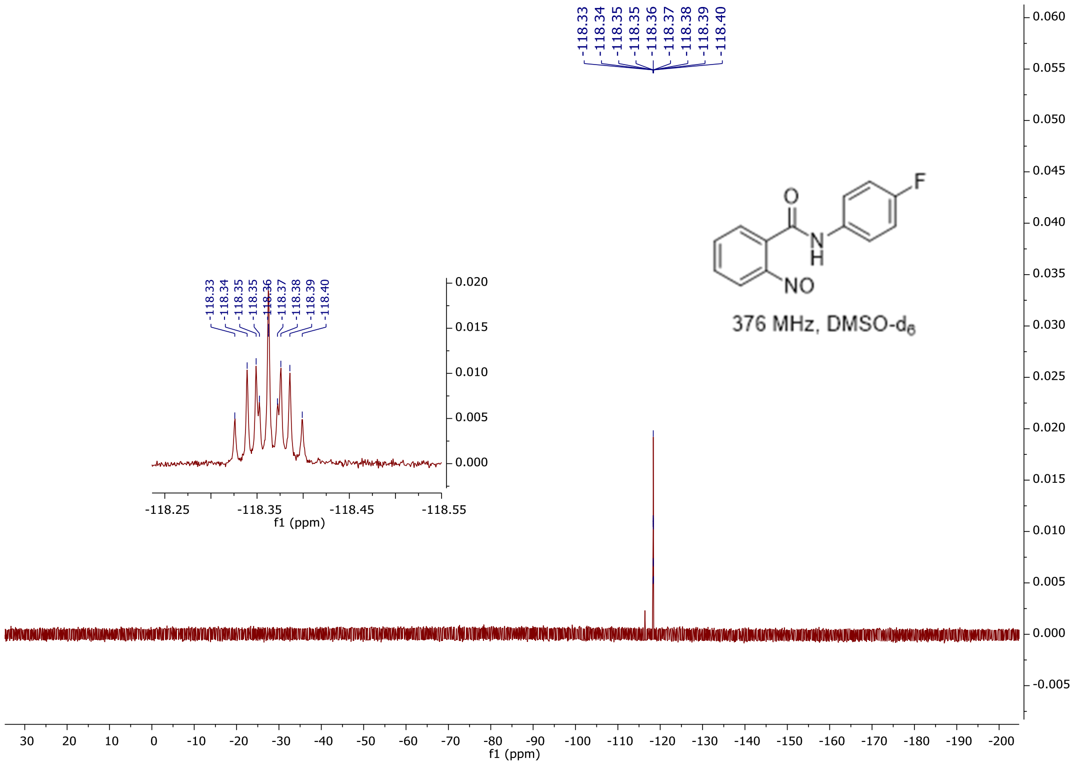
-118.33
-118.34
-118.35
-118.35
-118.36
-118.37
-118.38
-118.39
-118.40
376 MHz, DMSO-d6
f1 (ppm)

2k: *N*-(4-isopropylphenyl)-2-nitrosobenzamide

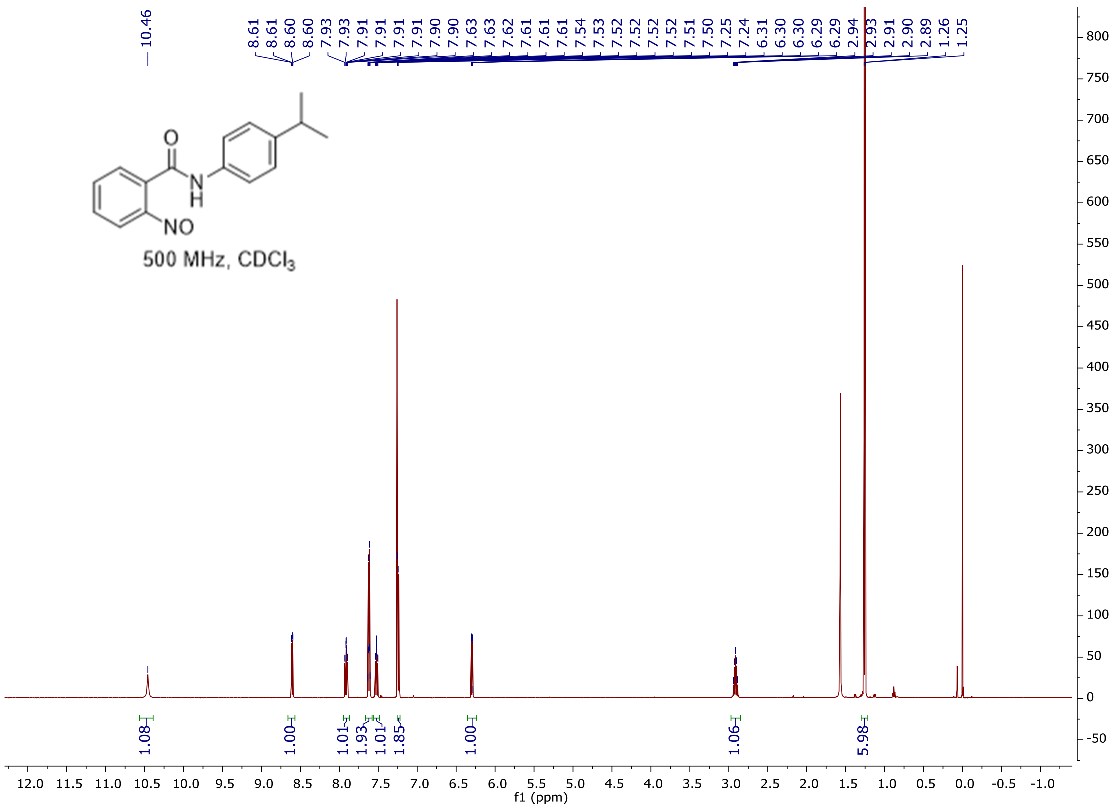

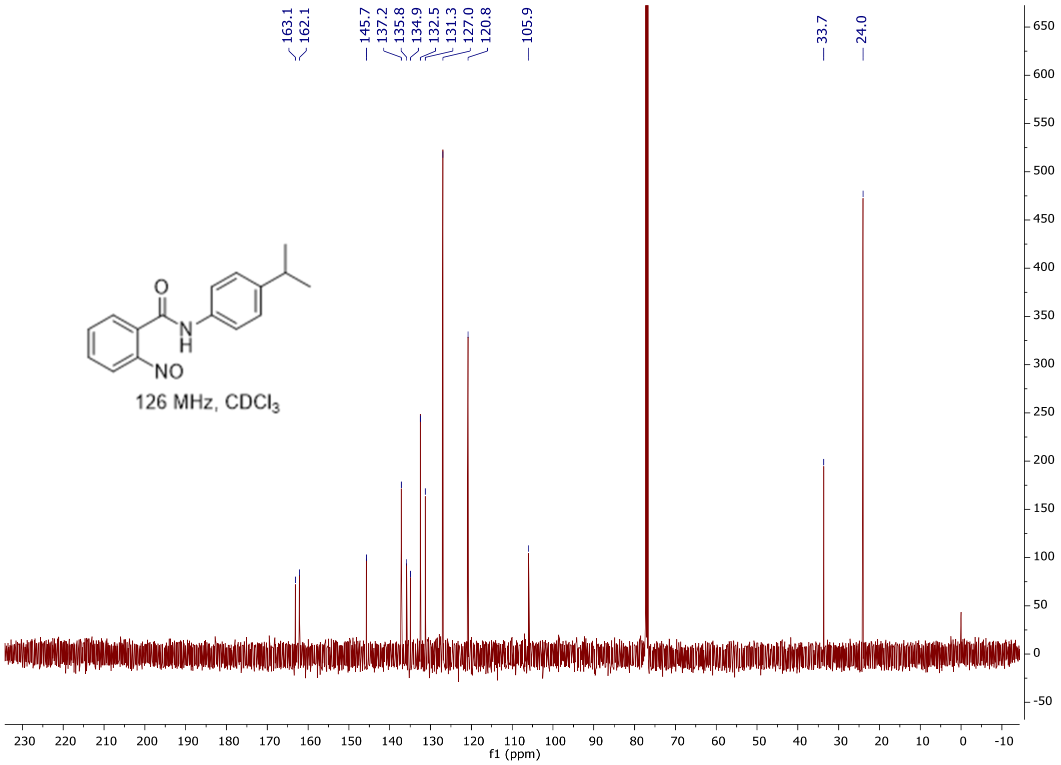

2l: *N*-cyclopentyl-5-fluoro-2-nitrosobenzamide

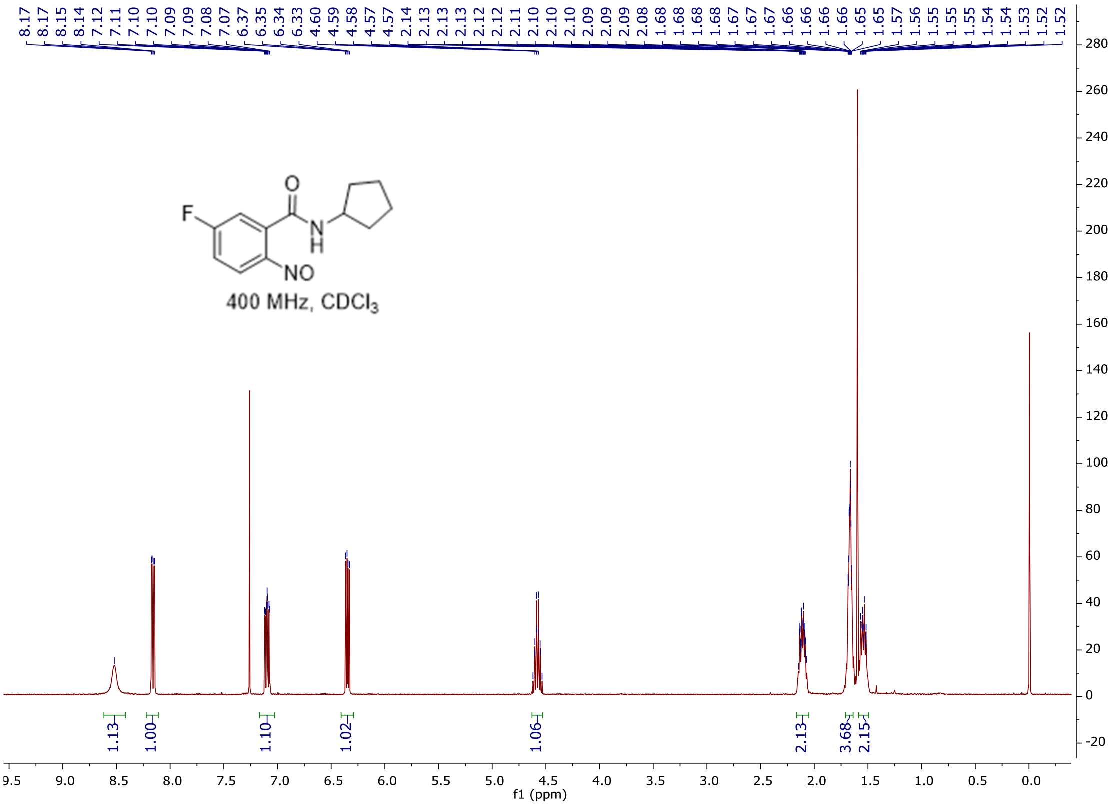

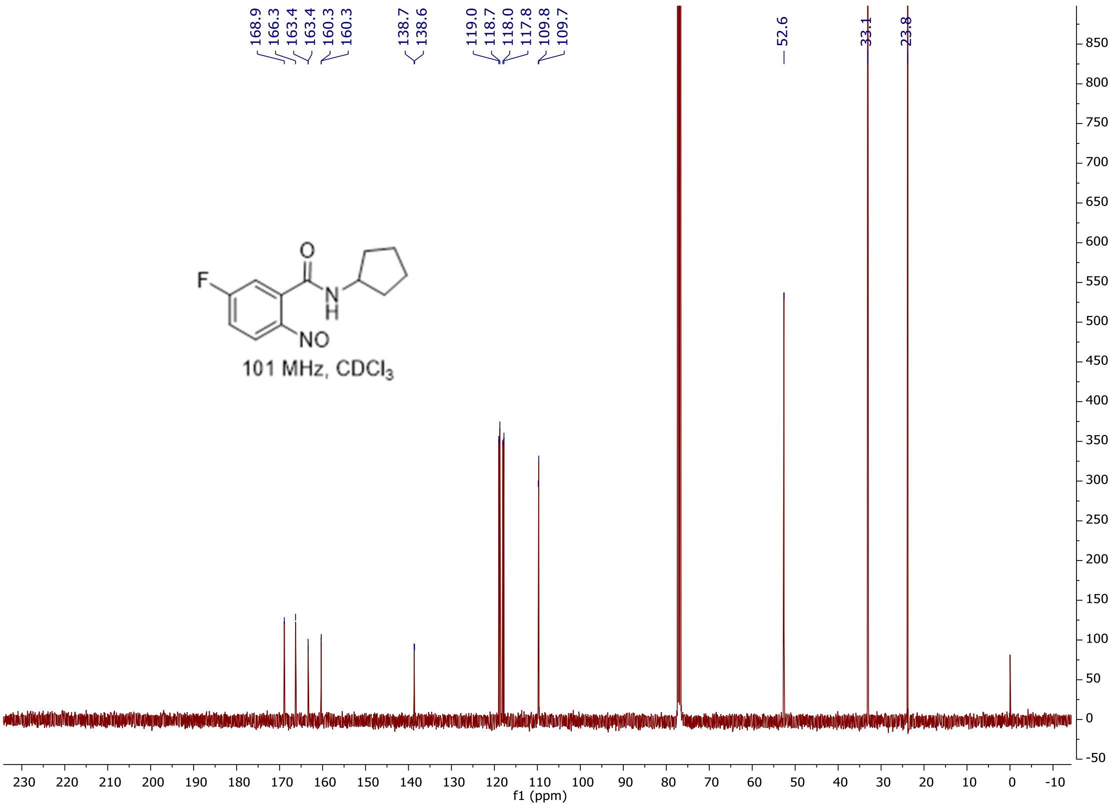

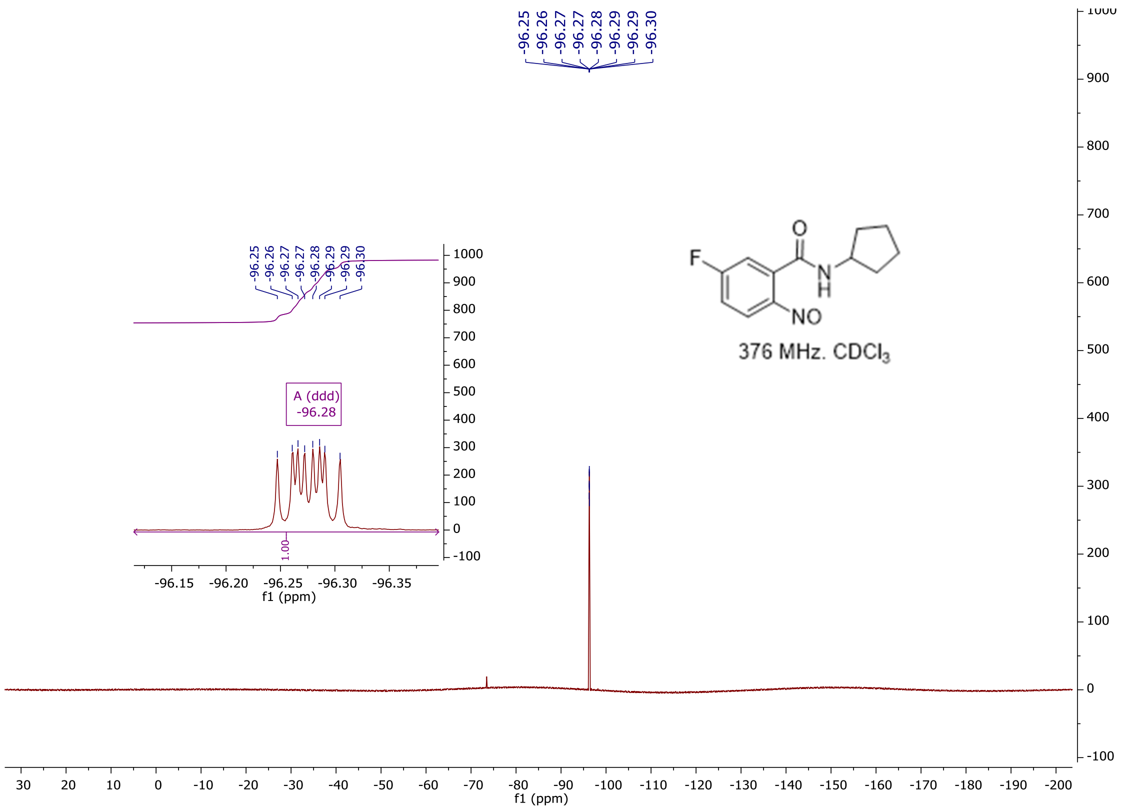

-96.25
-96.26
-96.27
-96.27
-96.28
-96.29
-96.29
-96.30
A (ddd)
-96.28
1.00
f1 (ppm)
376 MHz. CDCl3
F
O
N
H
NO

2m: *N*-cyclopentyl-6-nitrosobenzo[d][1,3]dioxole-5-carboxamide

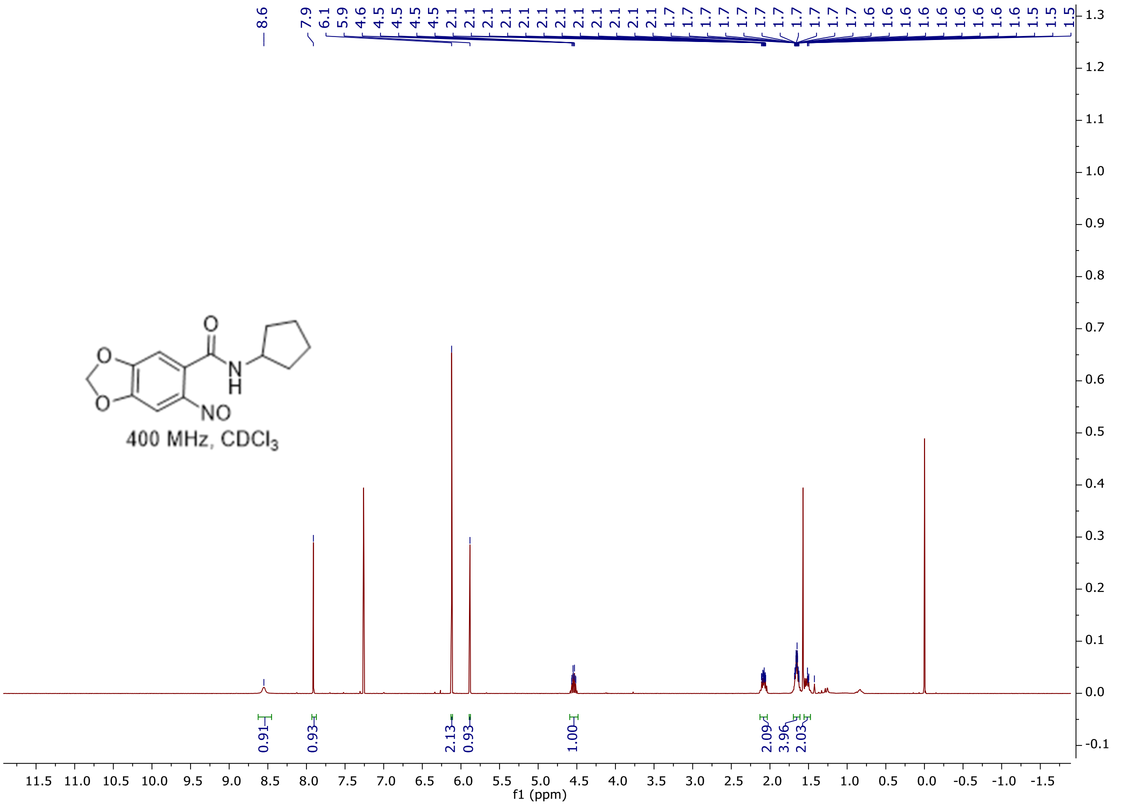

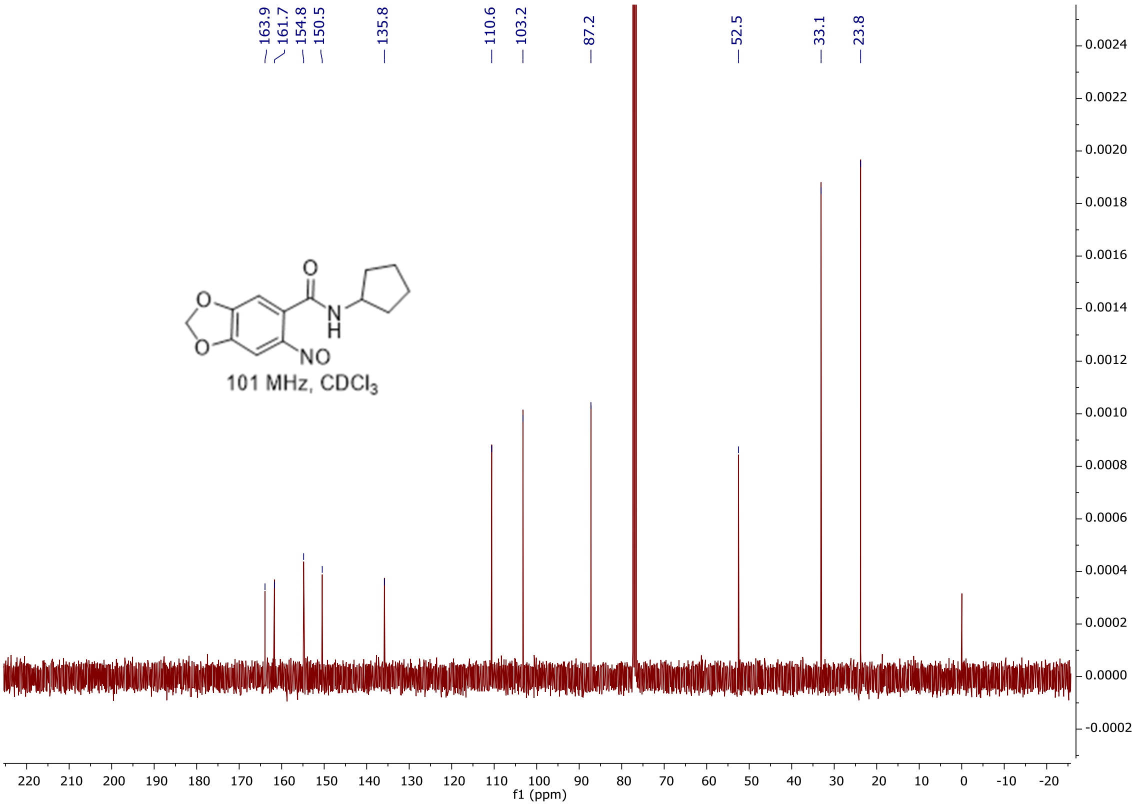

2n: methyl 4-(*tert*-butylcarbamoyl)-3-nitrosobenzoate

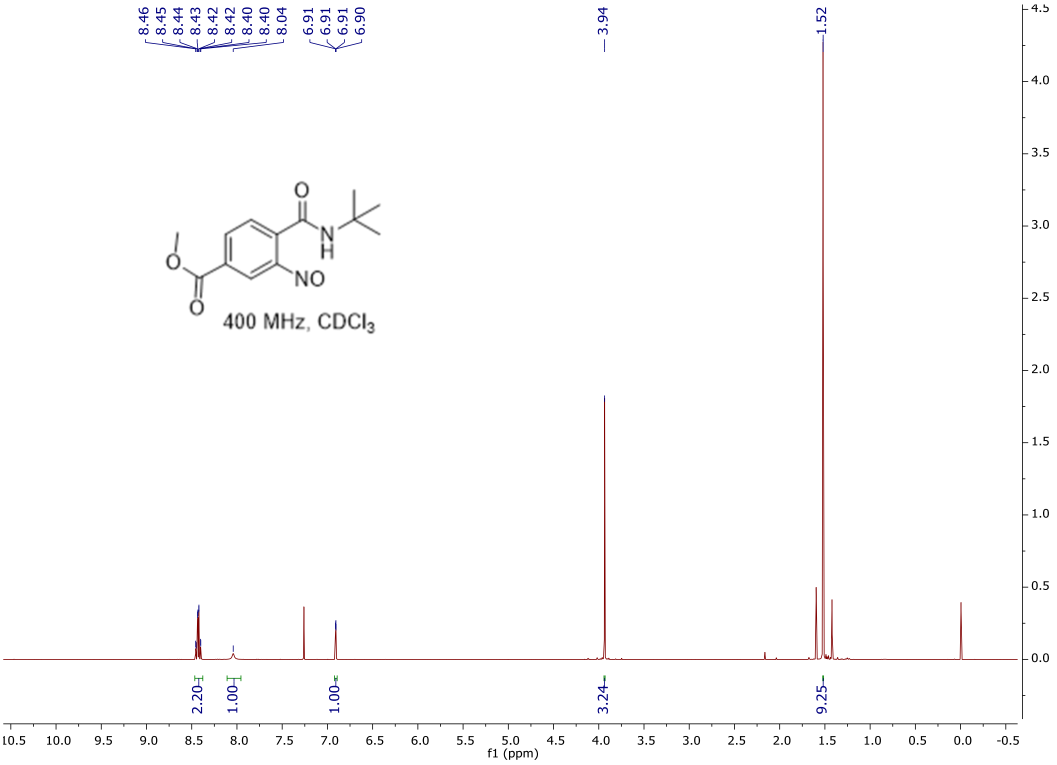

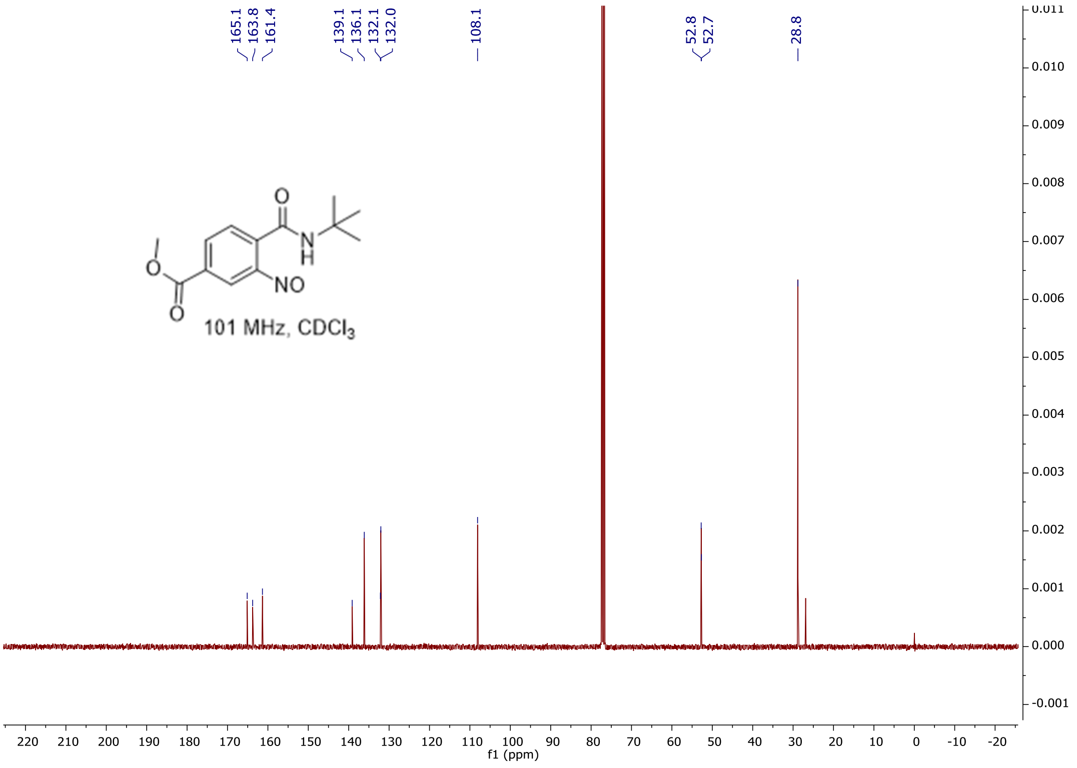

2o: *N*-(*tert*-butyl)-3-methyl-2-nitrosobenzamide

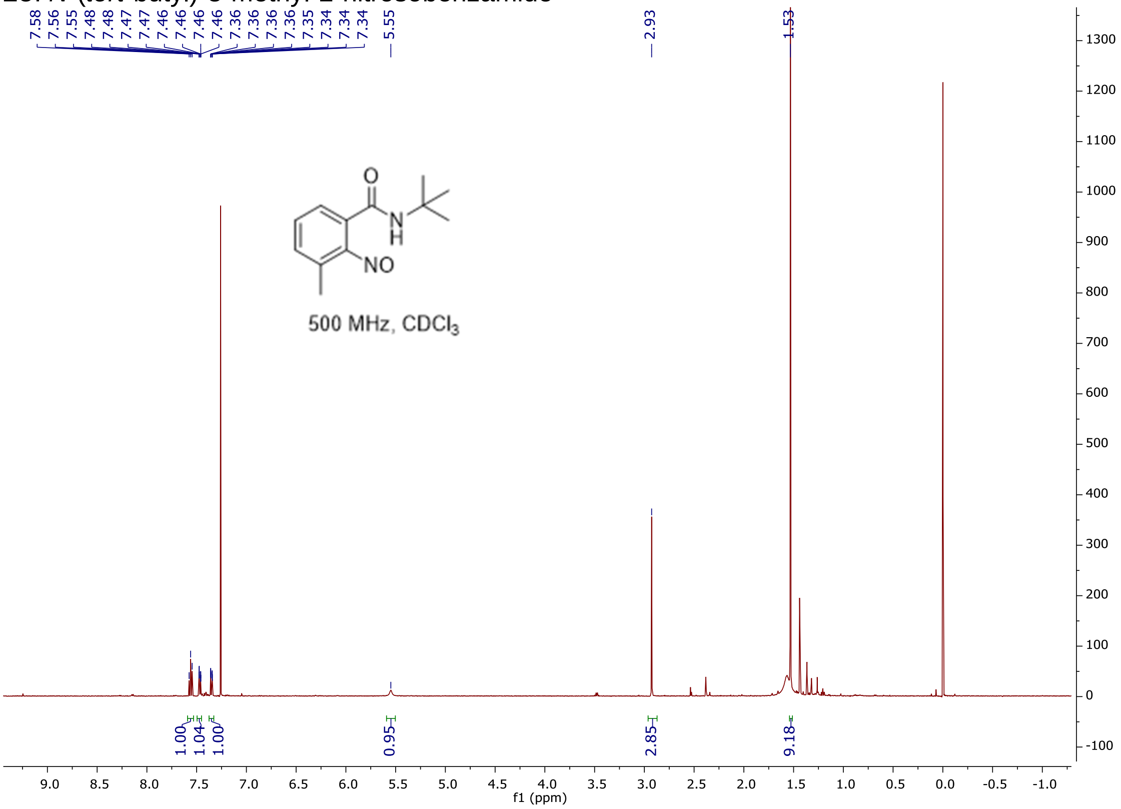

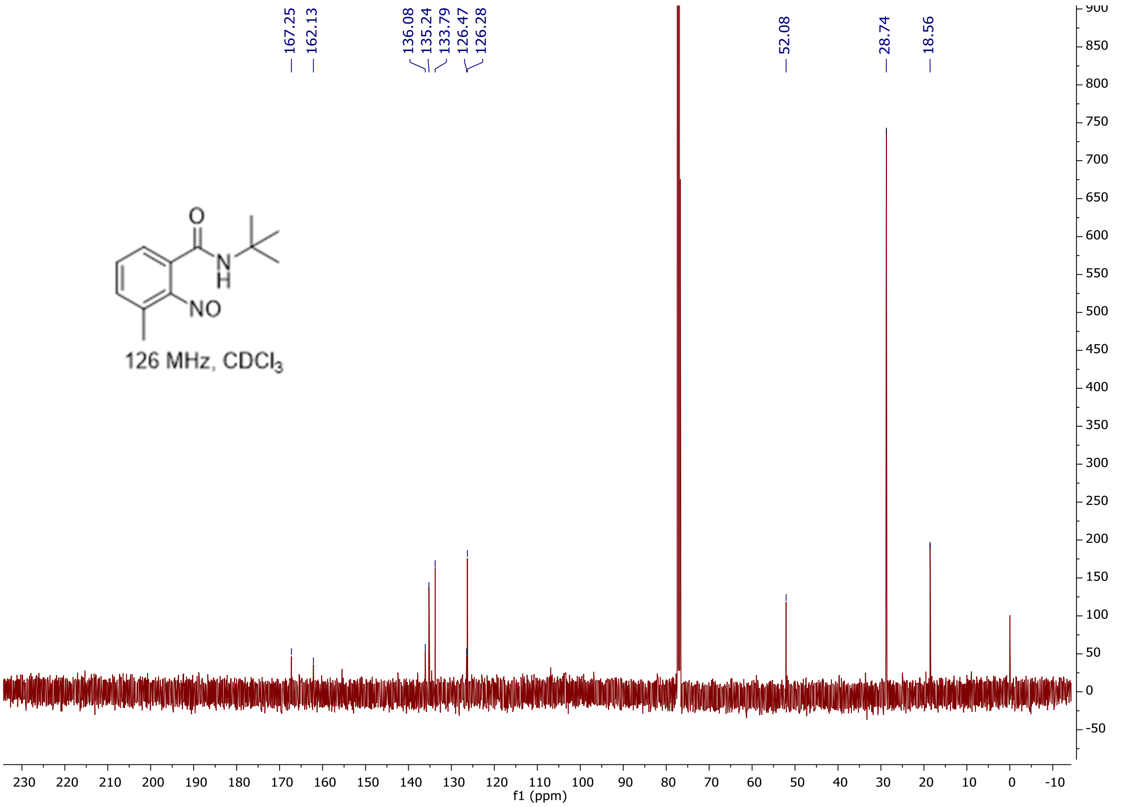

NMR copies of side products **3** and **4**

3: 2,2,2-trifluoroethyl *(Z)*-*N*-cyclopentyl-2-nitrosobenzimidate

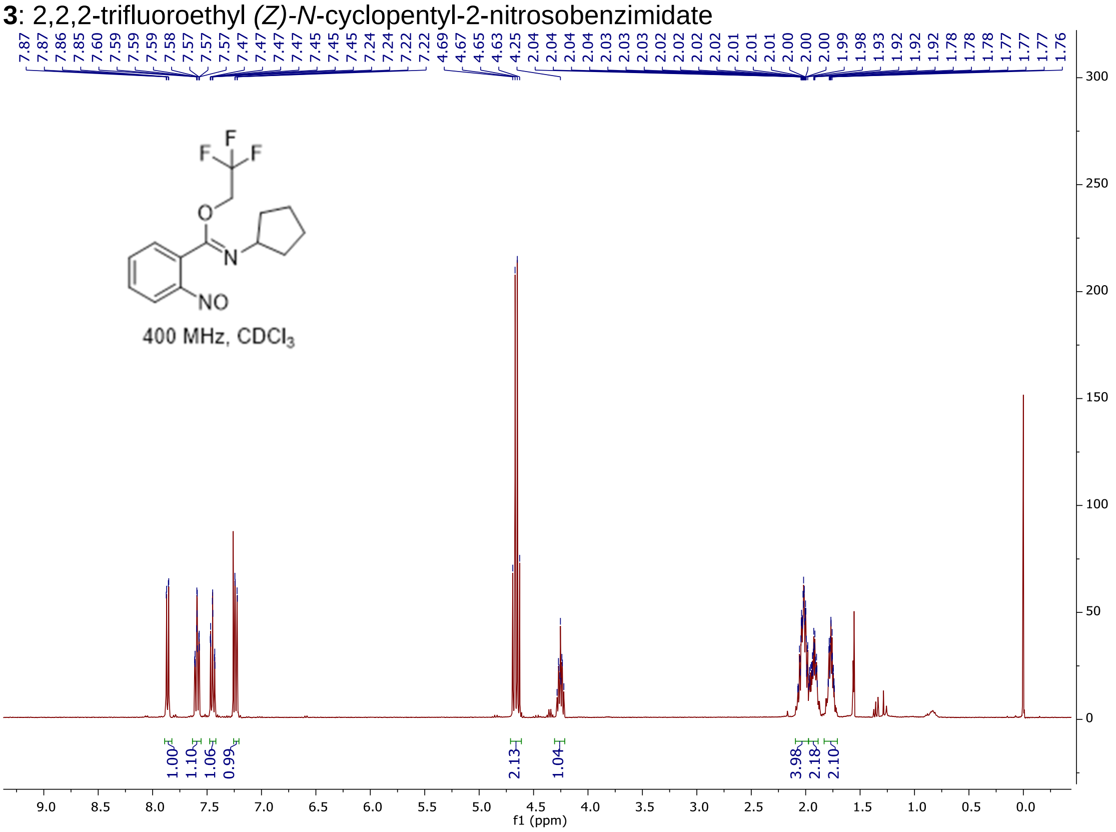

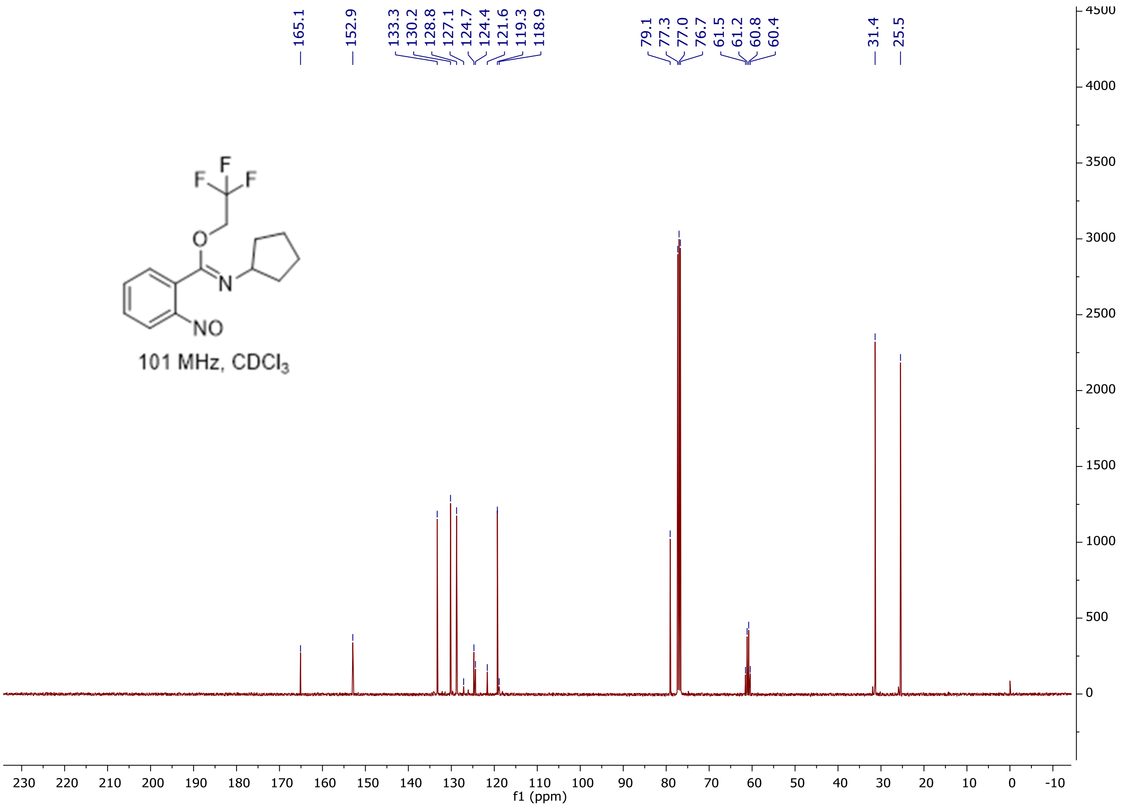

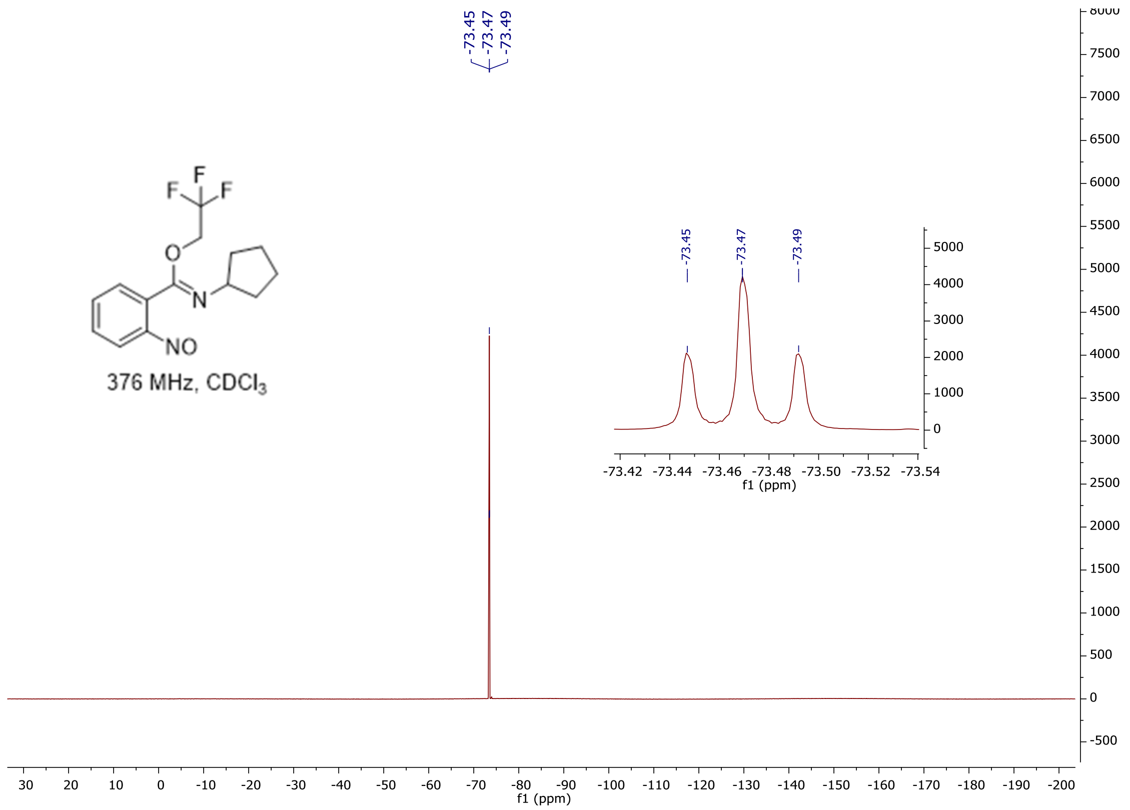

-73.45
-73.47
-73.49
F
F
F
O
N
NO
376 MHz, CDCl3
8000
7500
7000
6500
6000
5500
5000
4500
4000
3500
3000
2500
2000
1500
1000
500
0
-500
30 20 10 0 -10 -20 -30 -40 -50 -60 -70 -80 -90 -100 -110 -120 -130 -140 -150 -160 -170 -180 -190 -200
f1 (ppm)
-73.42 -73.44 -73.46 -73.48 -73.50 -73.52 -73.54

4: 2,2,2-trifluoroethyl 2-(2-cyclopentylidenehydrazineyl)benzoate

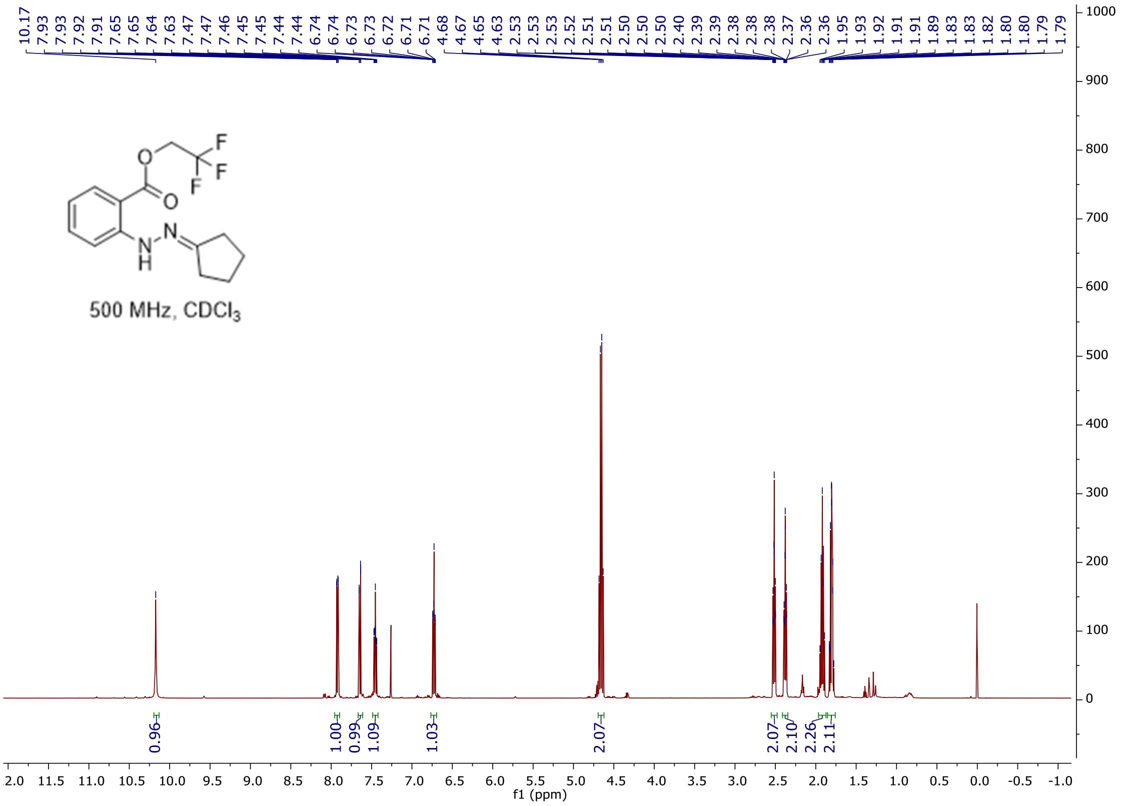

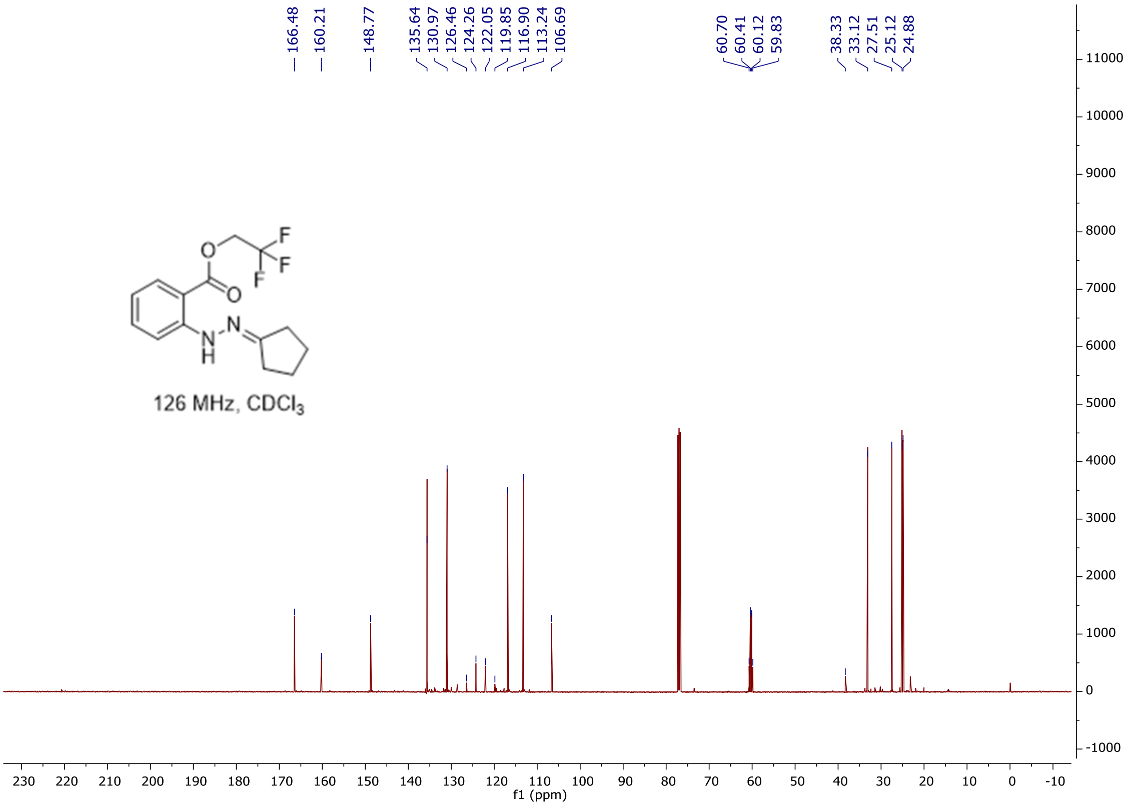

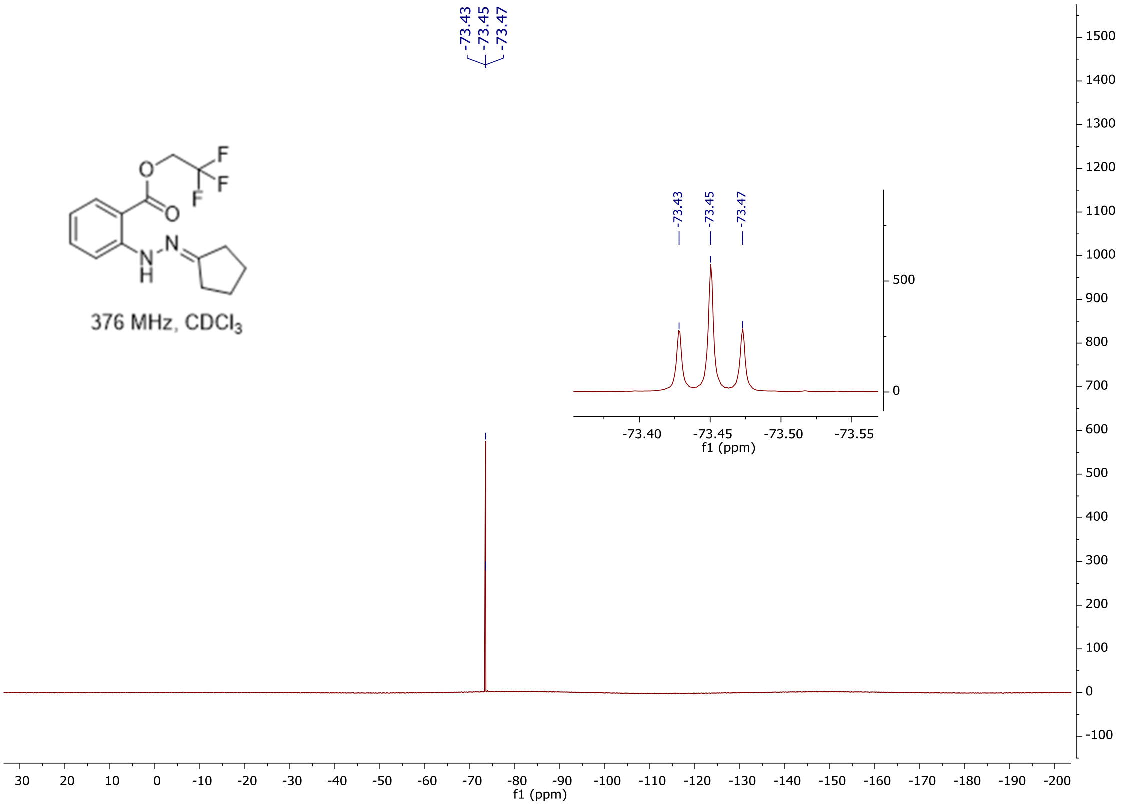
-73.43
-73.45
-73.47
376 MHz, CDCl3
f1 (ppm)

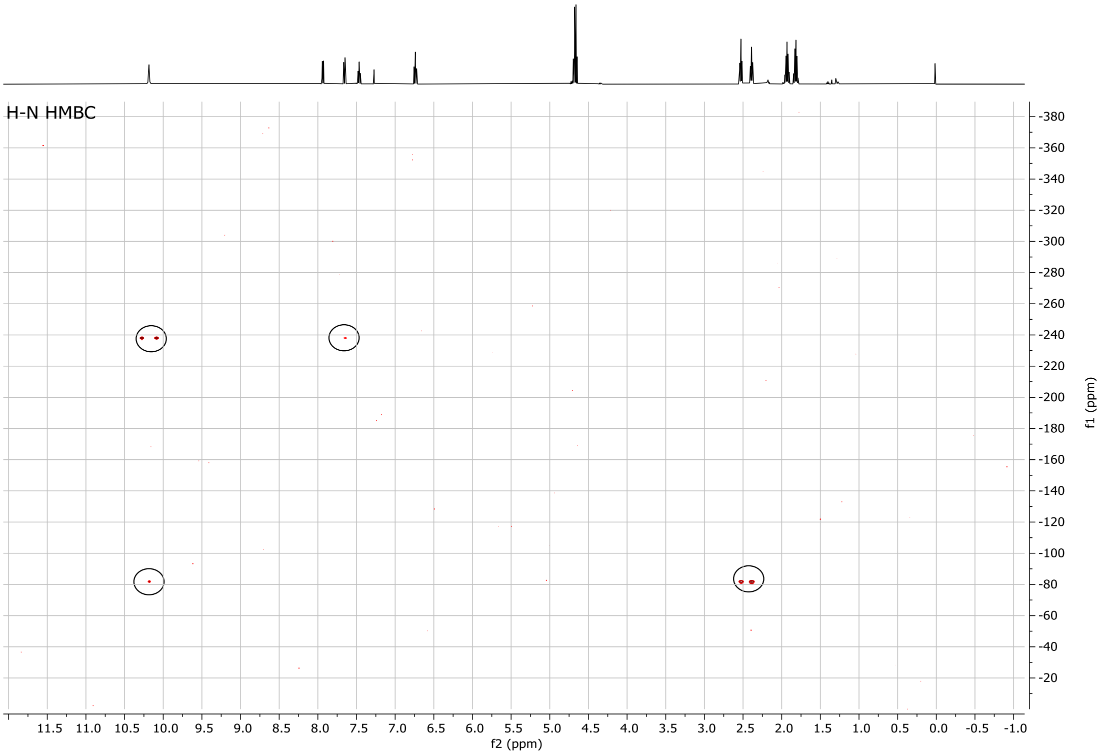
H-N HMBC
f2 (ppm)
f1 (ppm)

NMR copies of compounds: **5a-f**

5a: (*E*)-2-(2-(2-methylpropylidene)hydrazineyl)benzoic acid

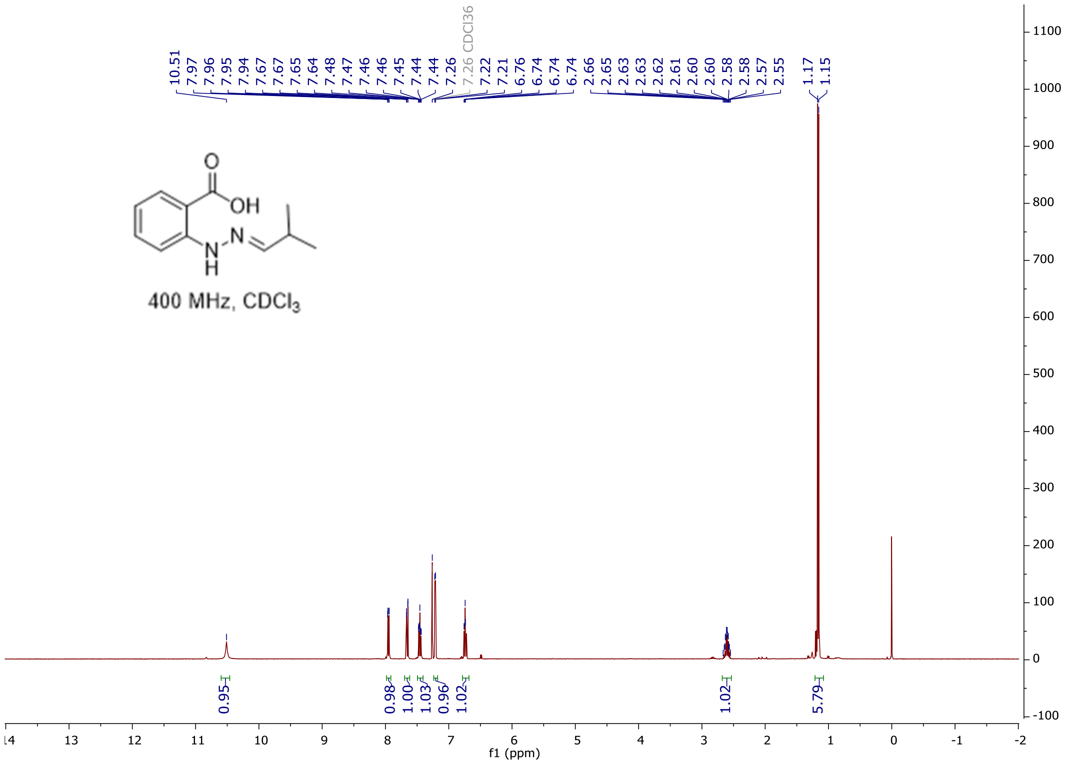

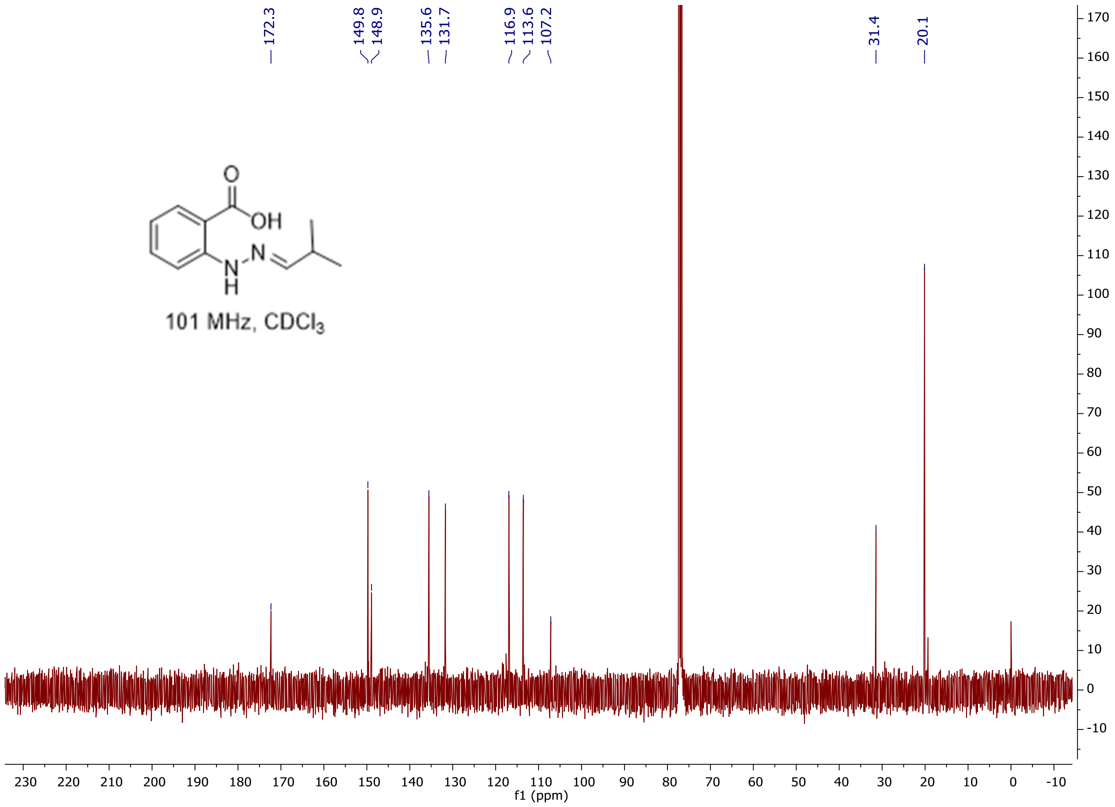

5b: 2-(2-cyclopentylidenehydrazineyl)benzoic acid

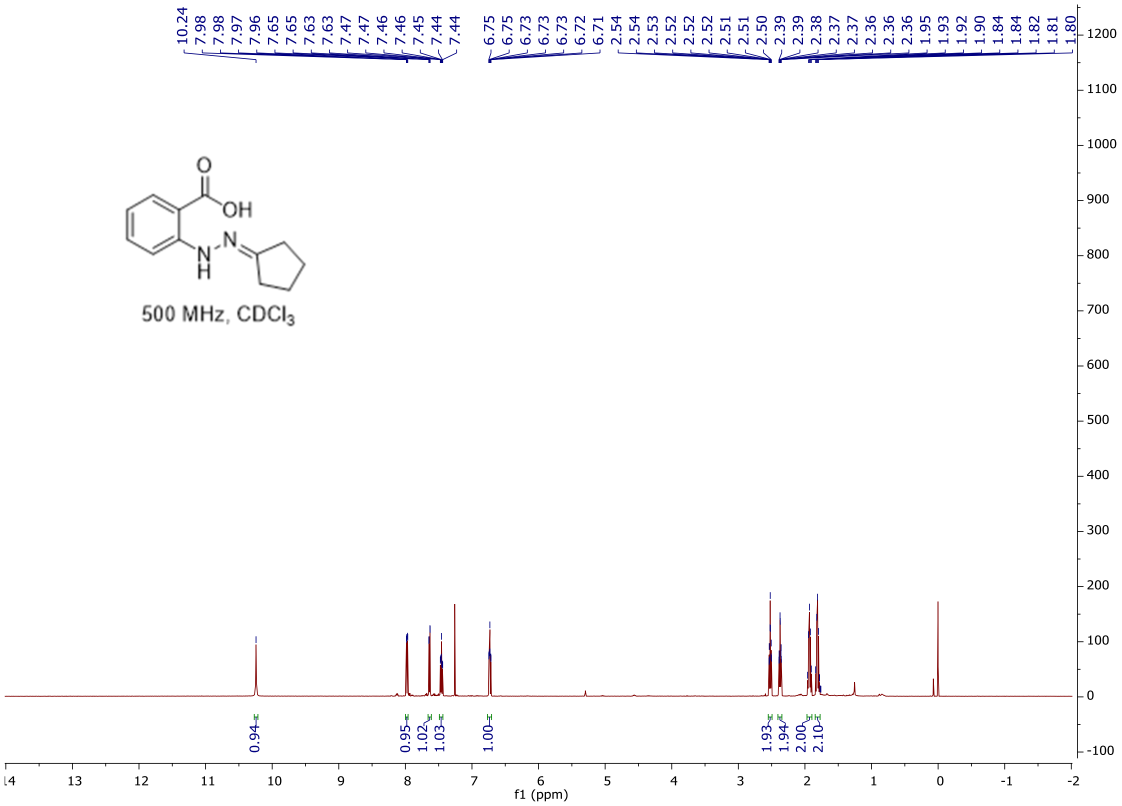

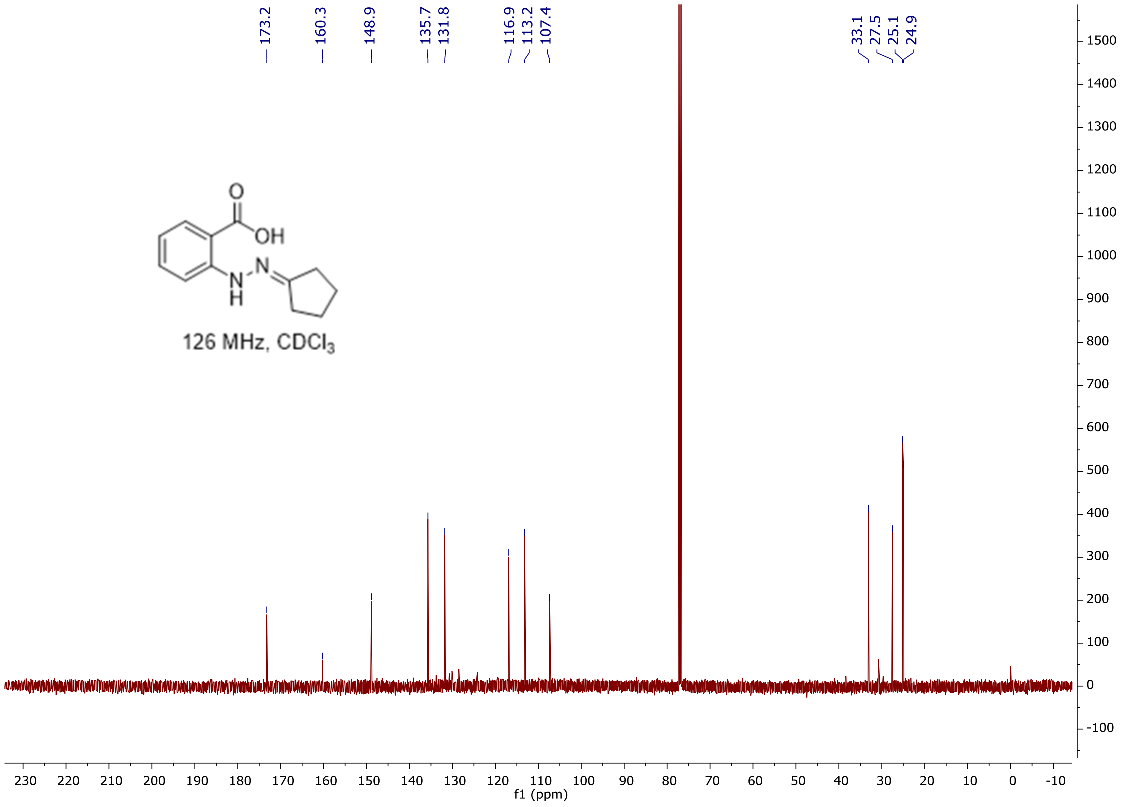

5c: (*E*)-2-(*tert*-butyldiazenyl)benzoic acid

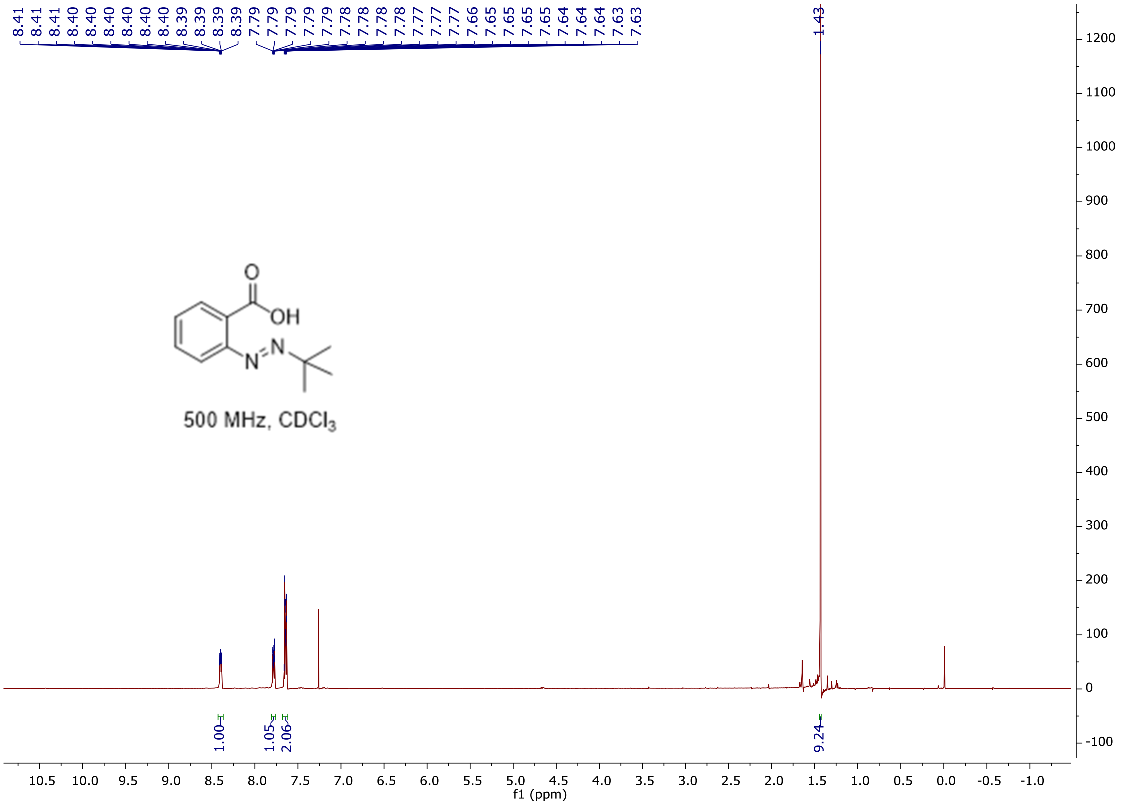

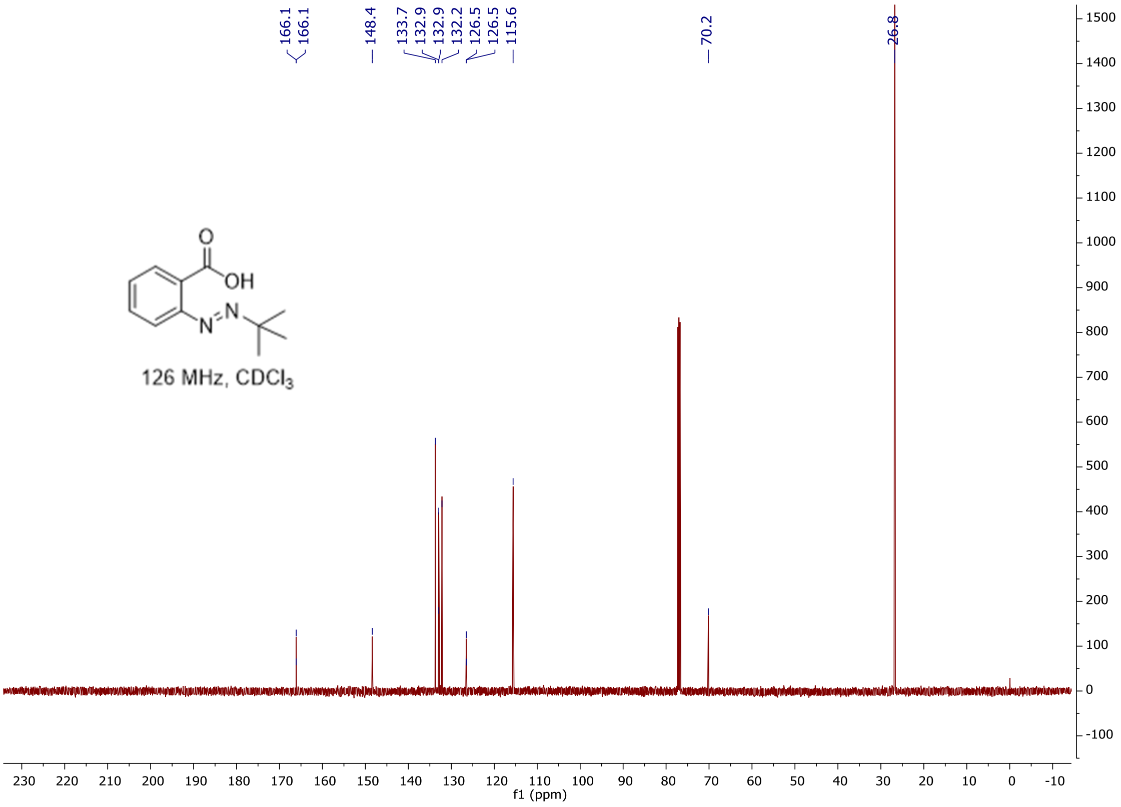

5d: (*E*)-2-(phenyldiazenyl)benzoic acid

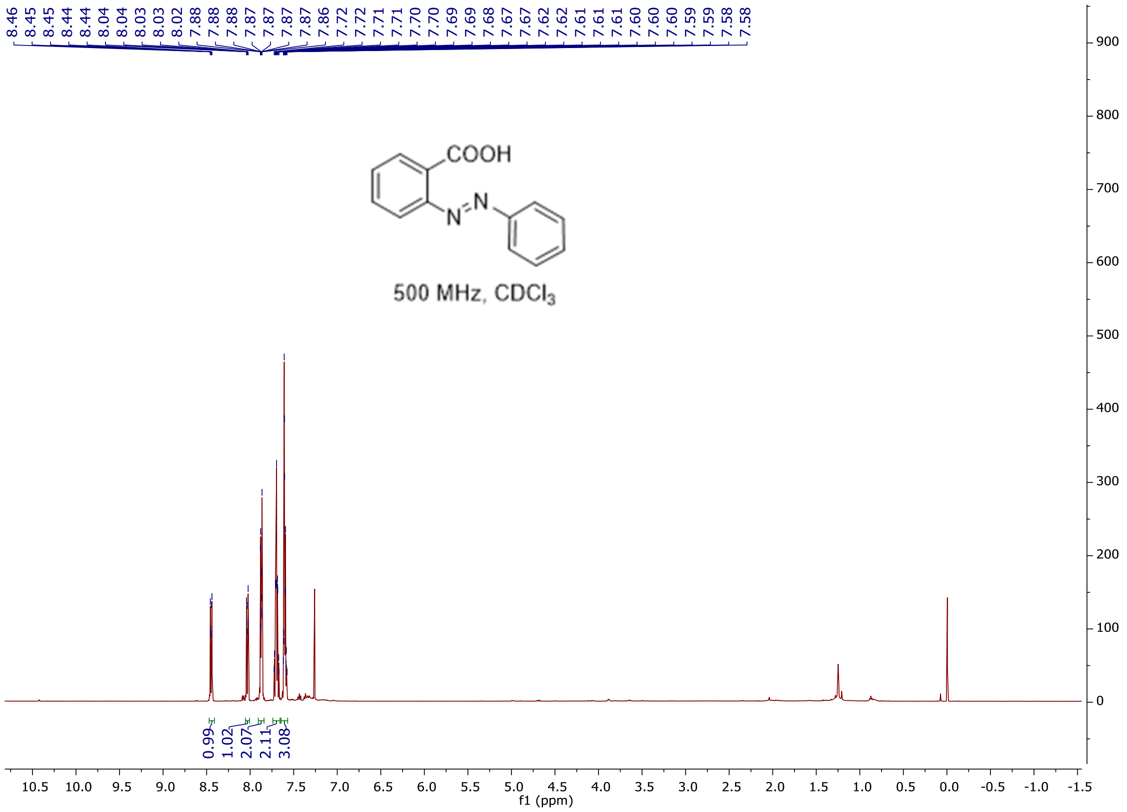

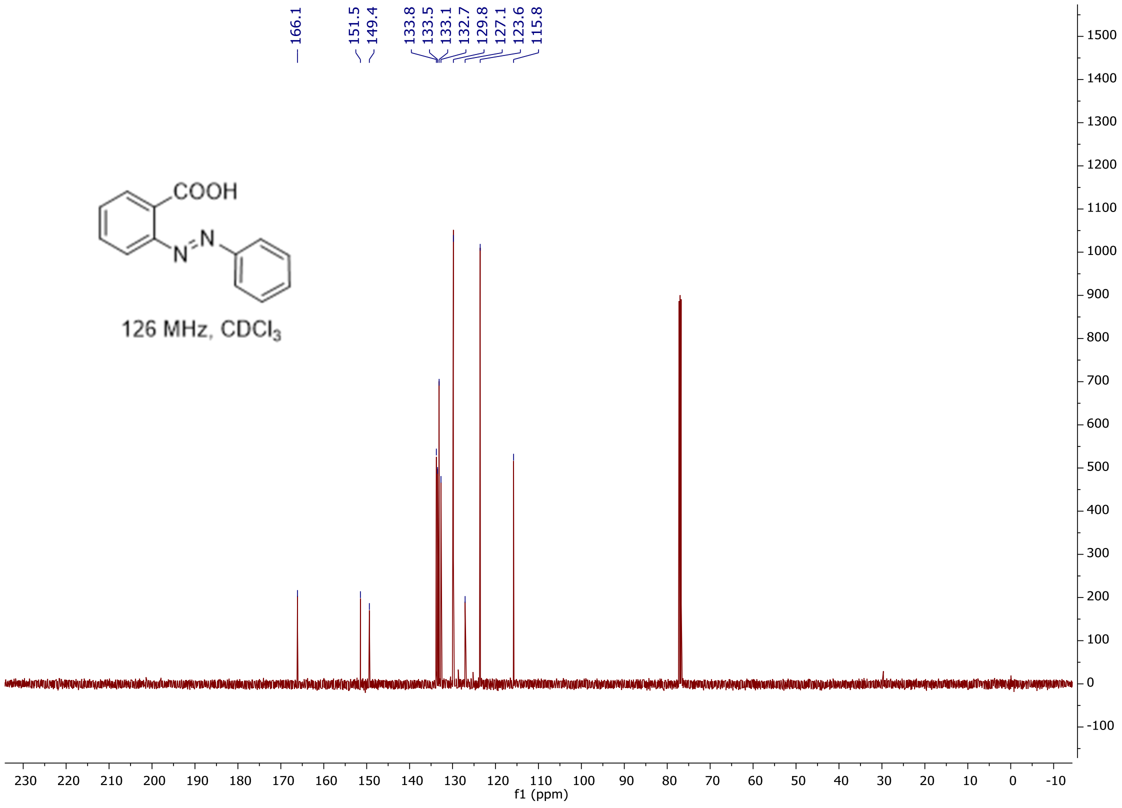

5e: 6-(2-cyclopentylidenehydrazineyl)benzo[*d*][1,3]dioxole-5-carboxylic acid

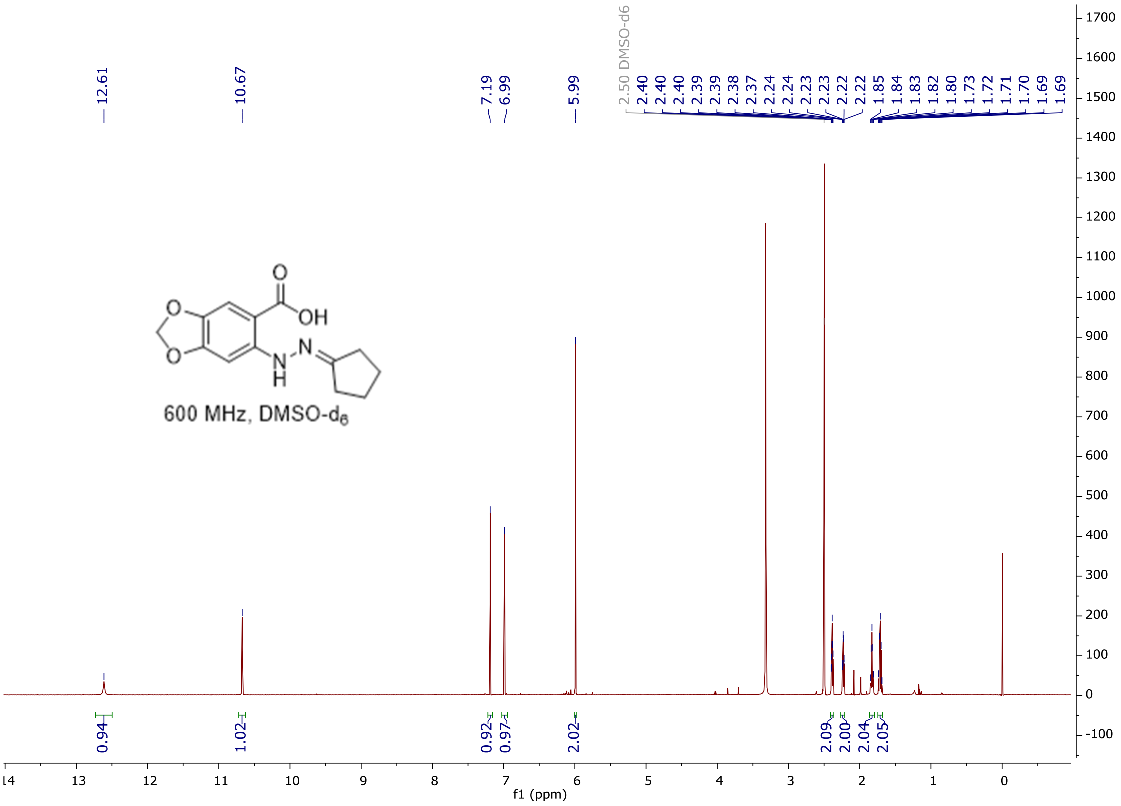

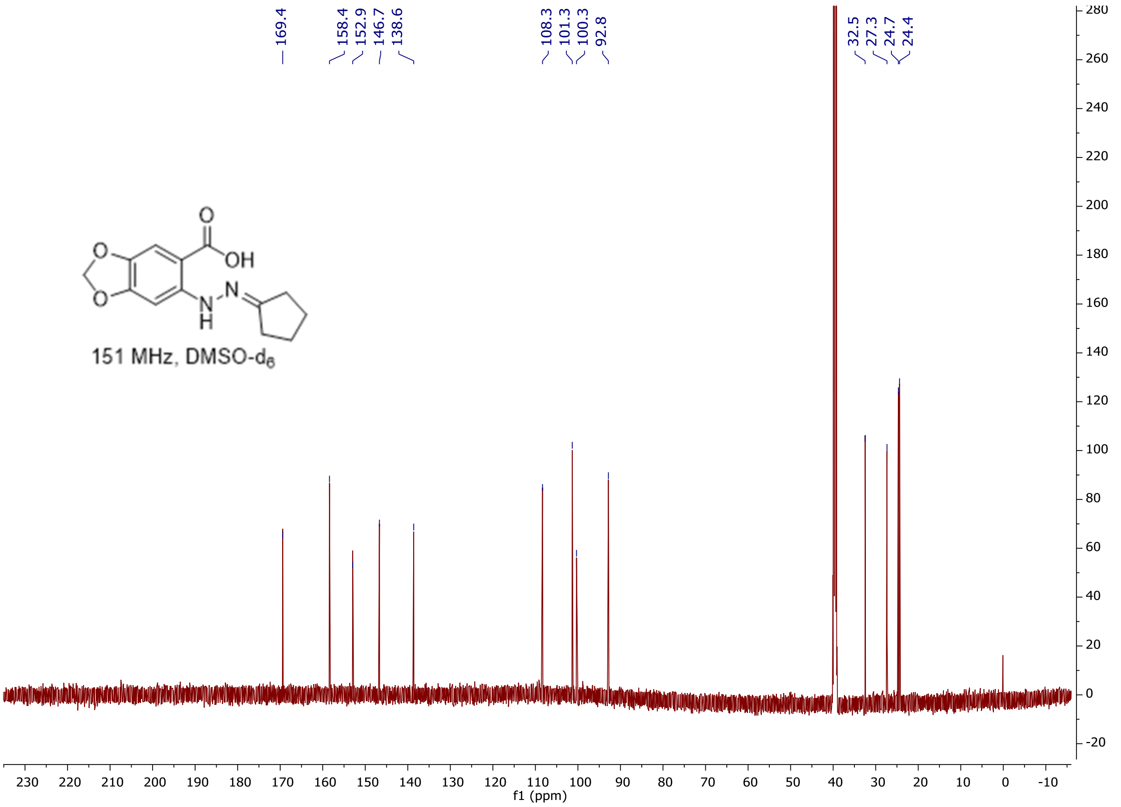

5f: (*E*)-2-(*tert*-butyldiazenyl)-3-methylbenzoic acid

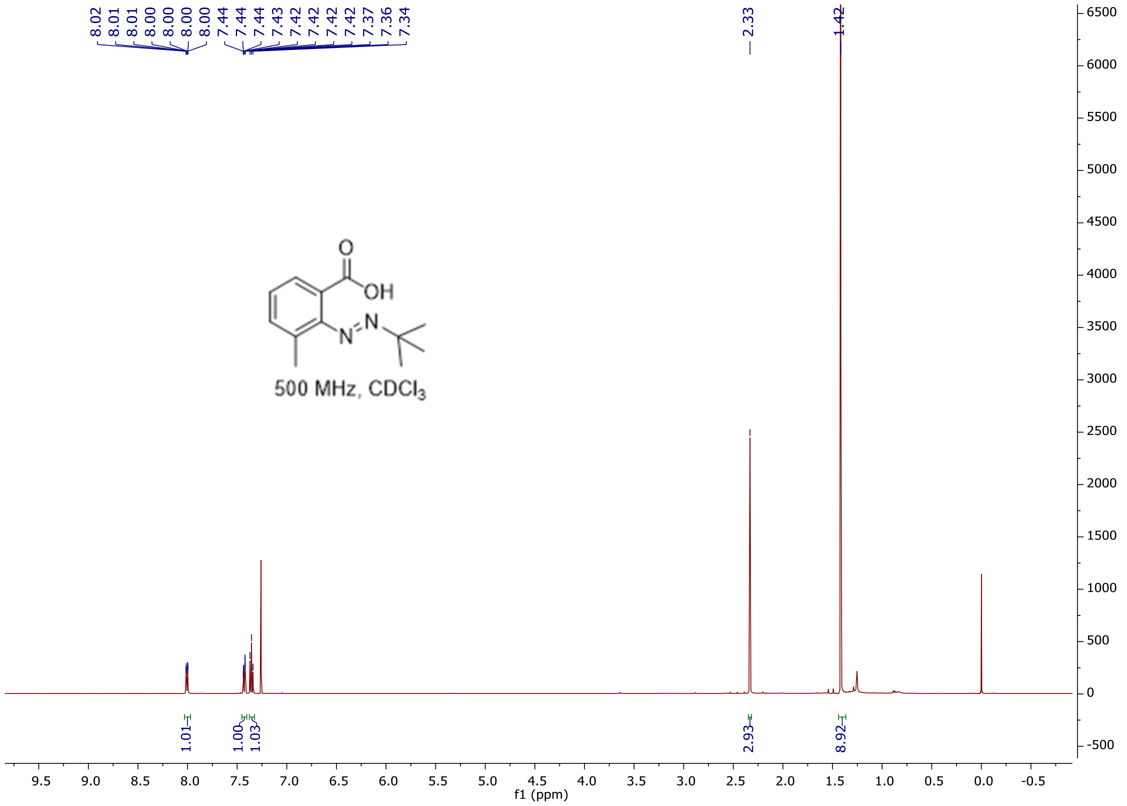

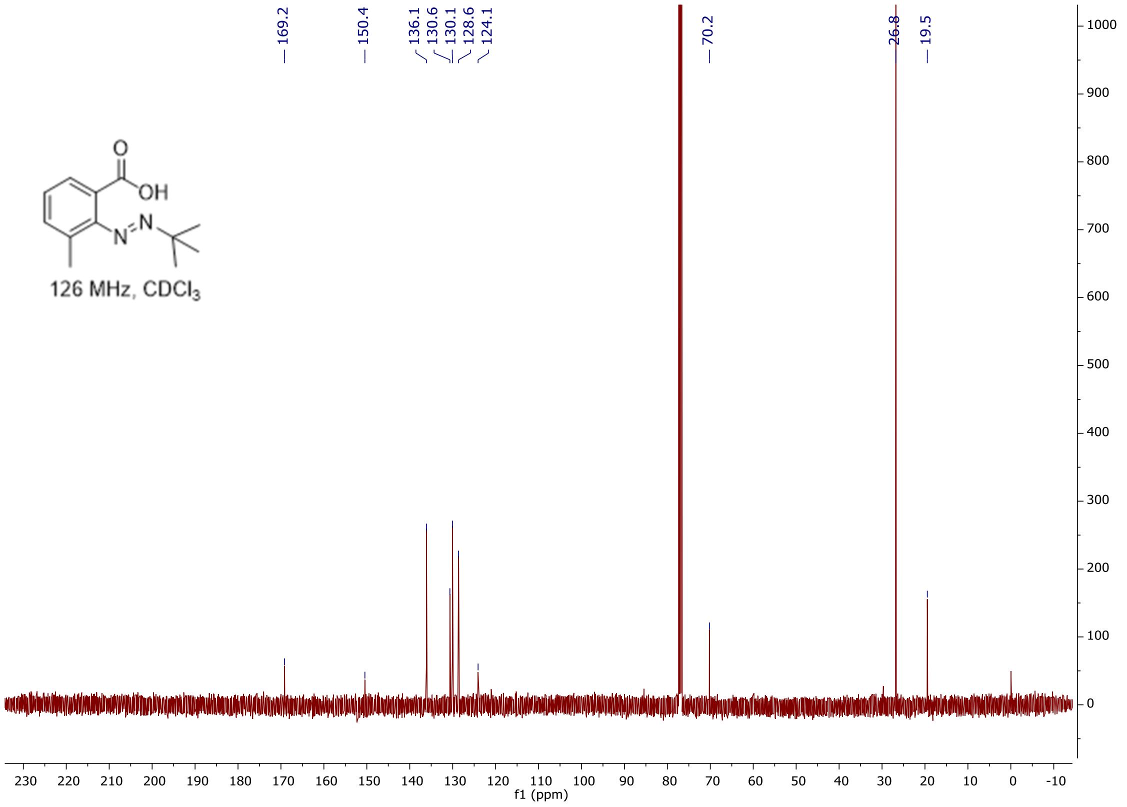

NMR copies of nitrosoarenes functionalization products: **6-9**

6: ***N*-cyclopentyl-2-nitrobenzamide**

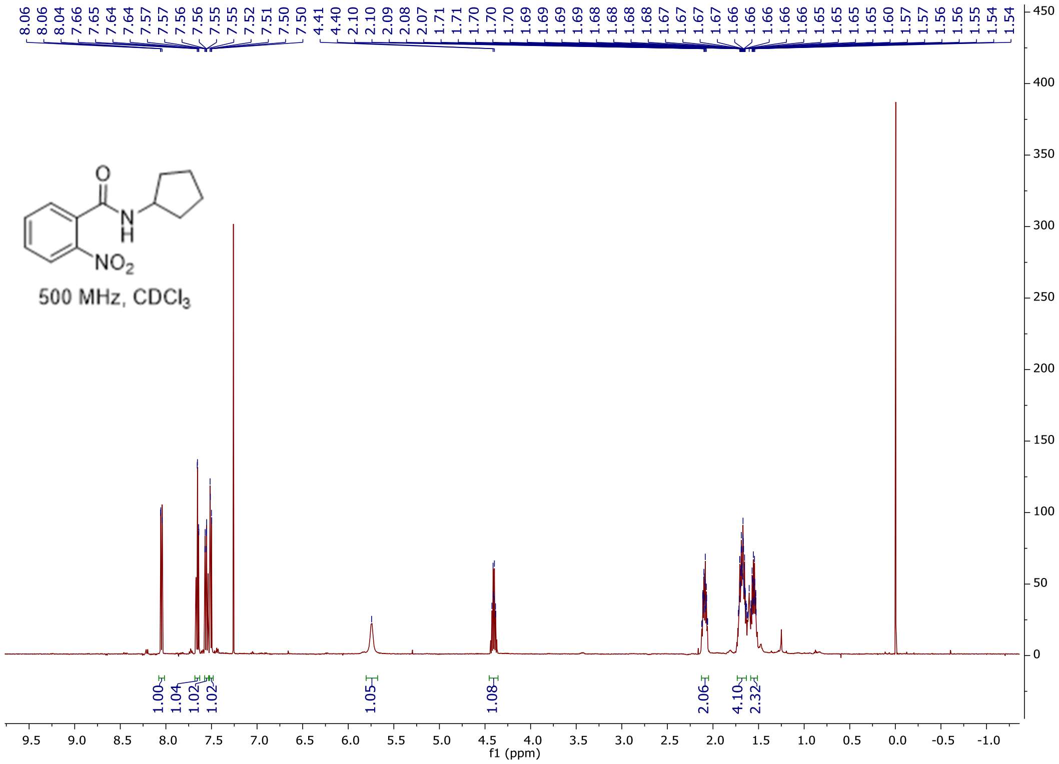

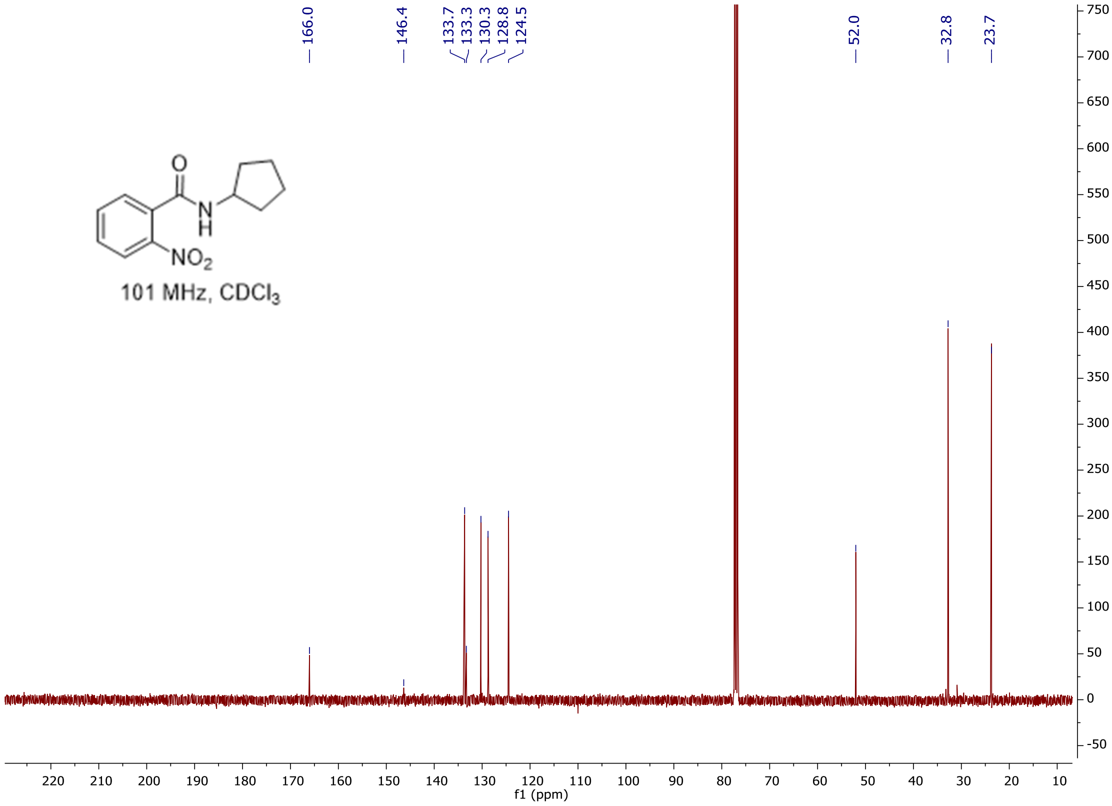

7: 2-amino-*N*-cyclopentylbenzamide

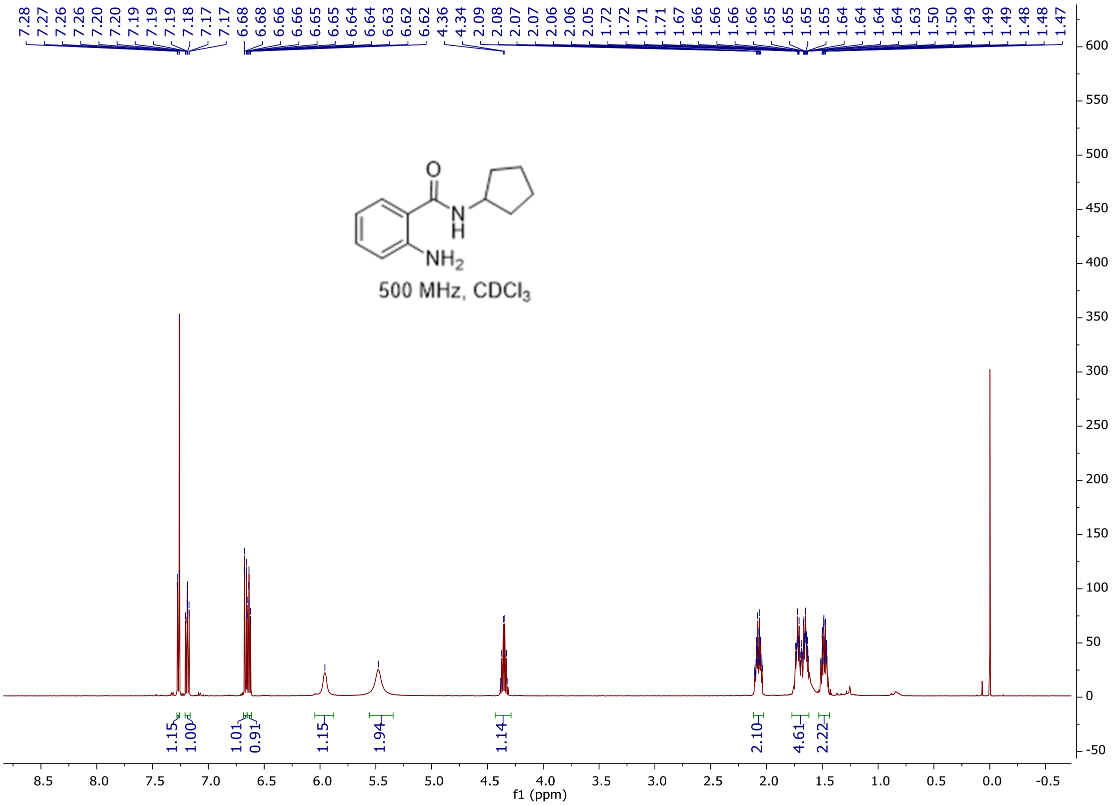

8: 2-(2-oxa-3-azabicyclo[2.2.1]hept-5-en-3-yl)-*N*-cyclopentylbenzamide

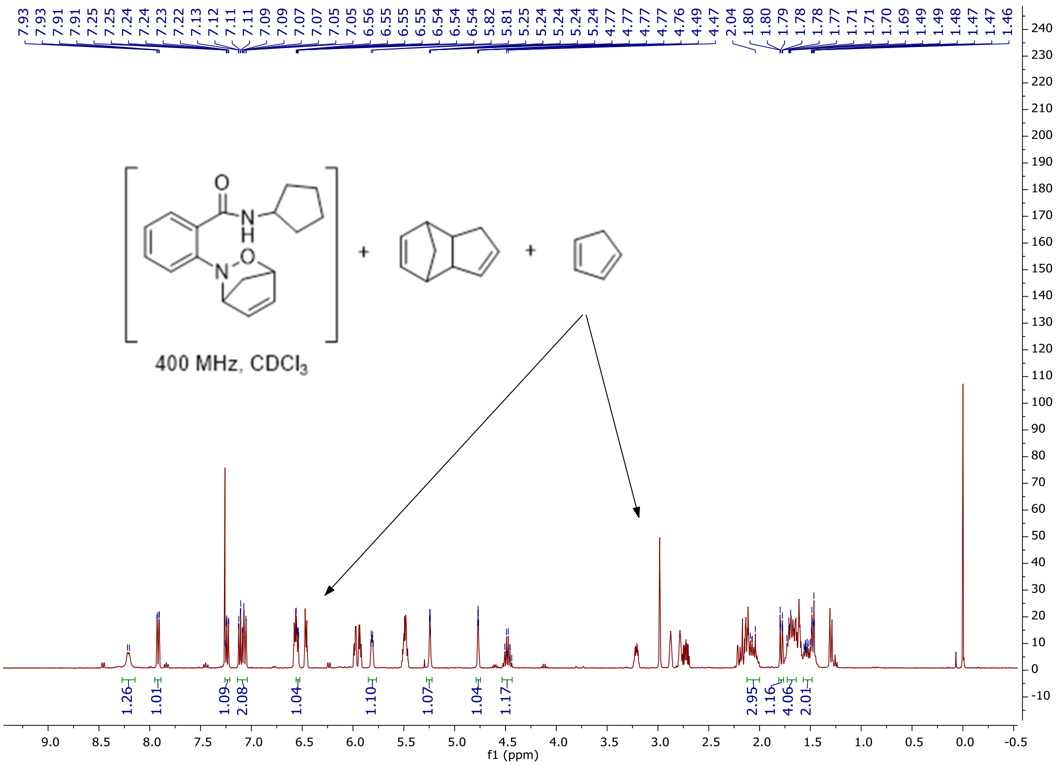

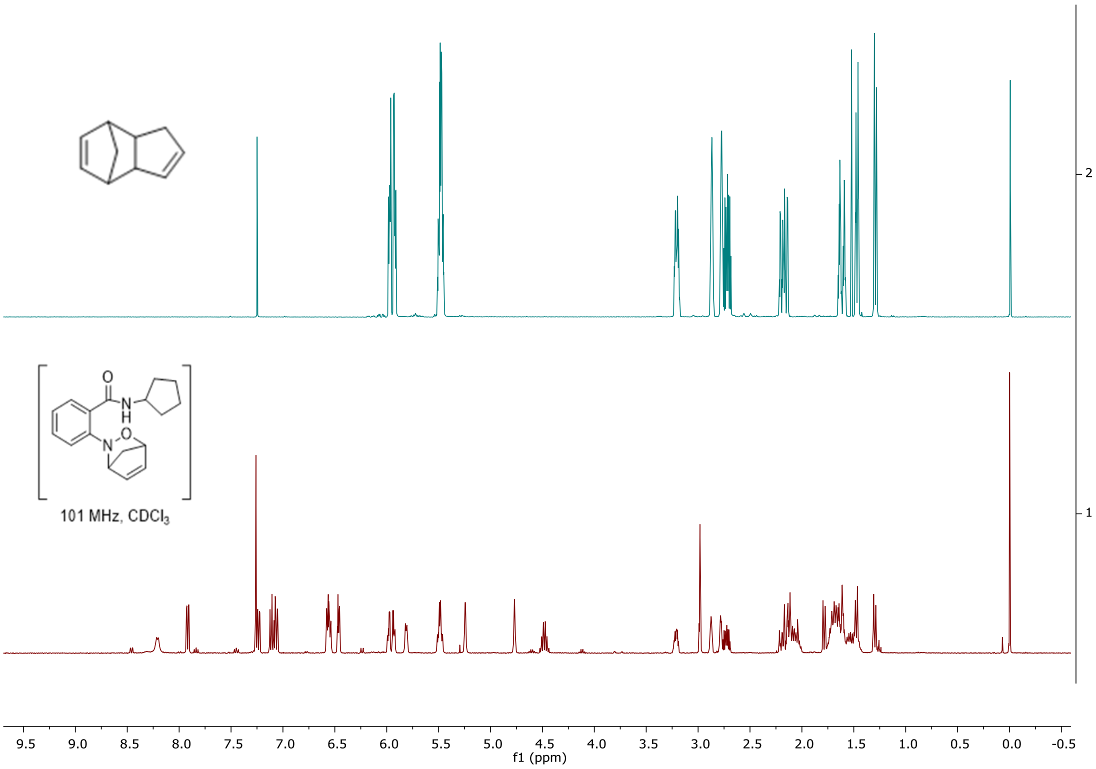

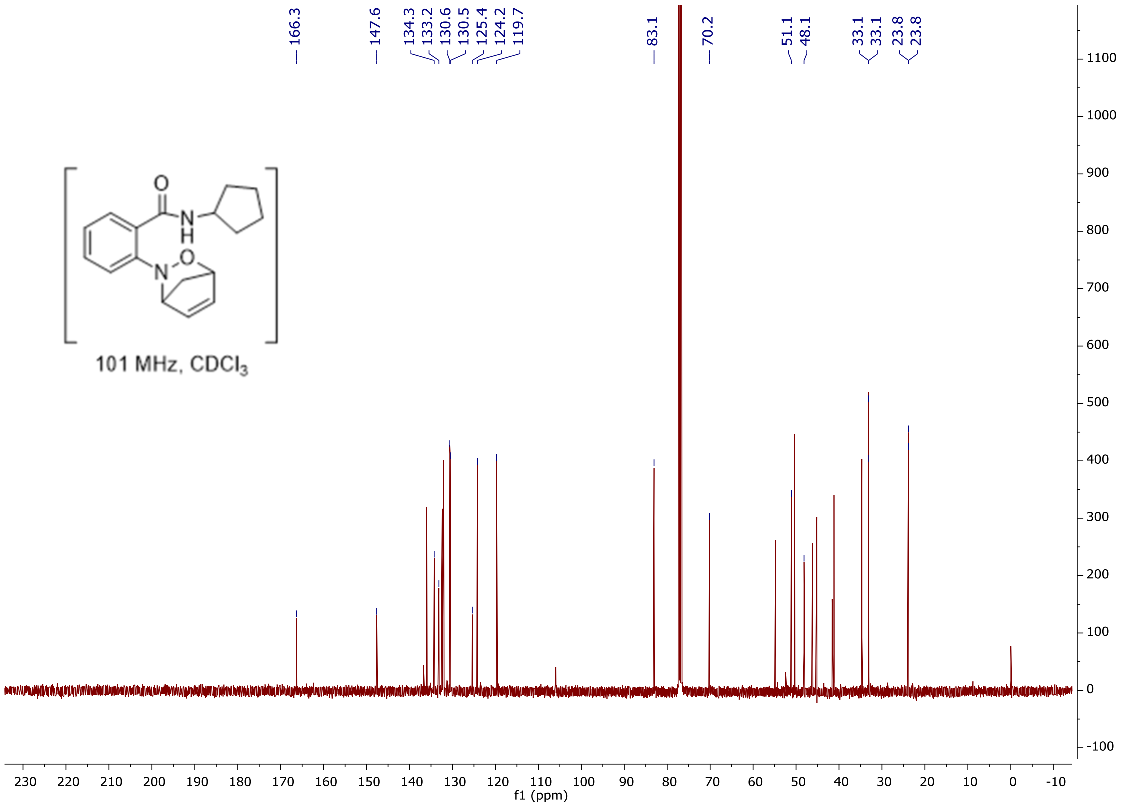
166.3
147.6
134.3
133.2
130.6
130.5
125.4
124.2
119.7
83.1
70.2
51.1
48.1
33.1
33.1
23.8
23.8
101 MHz, CDCl3
f1 (ppm)

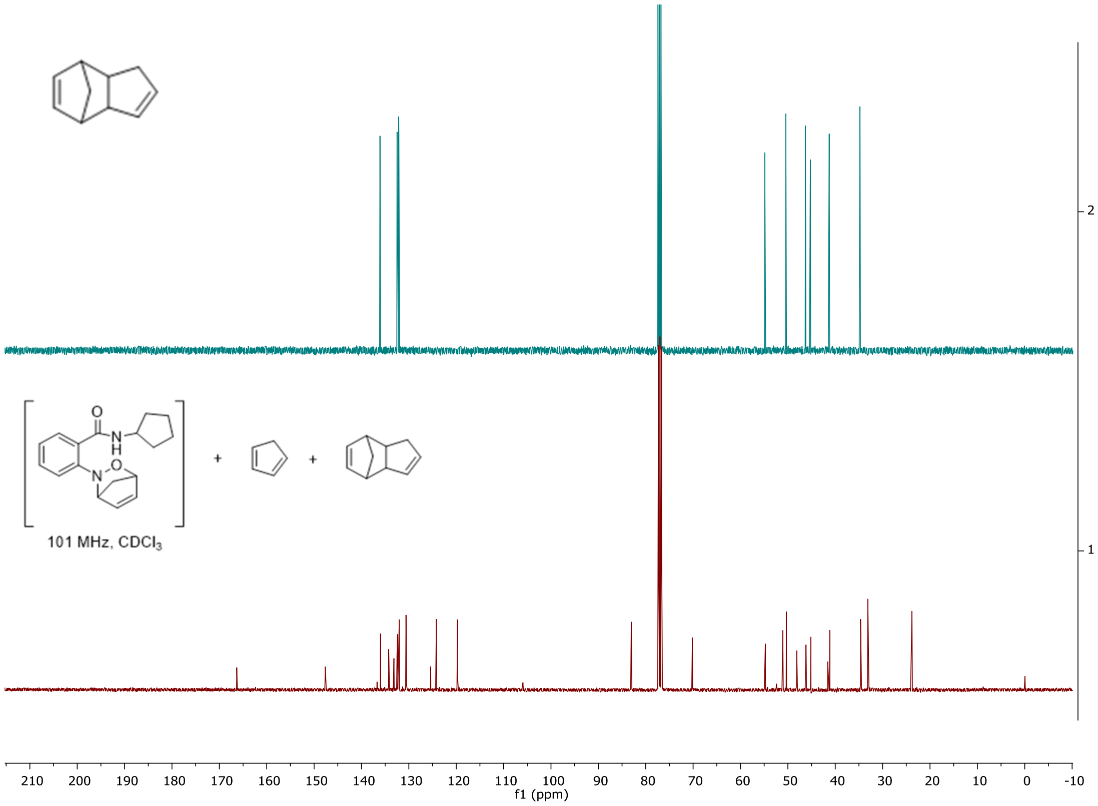
101 MHz, CDCl3
f1 (ppm)

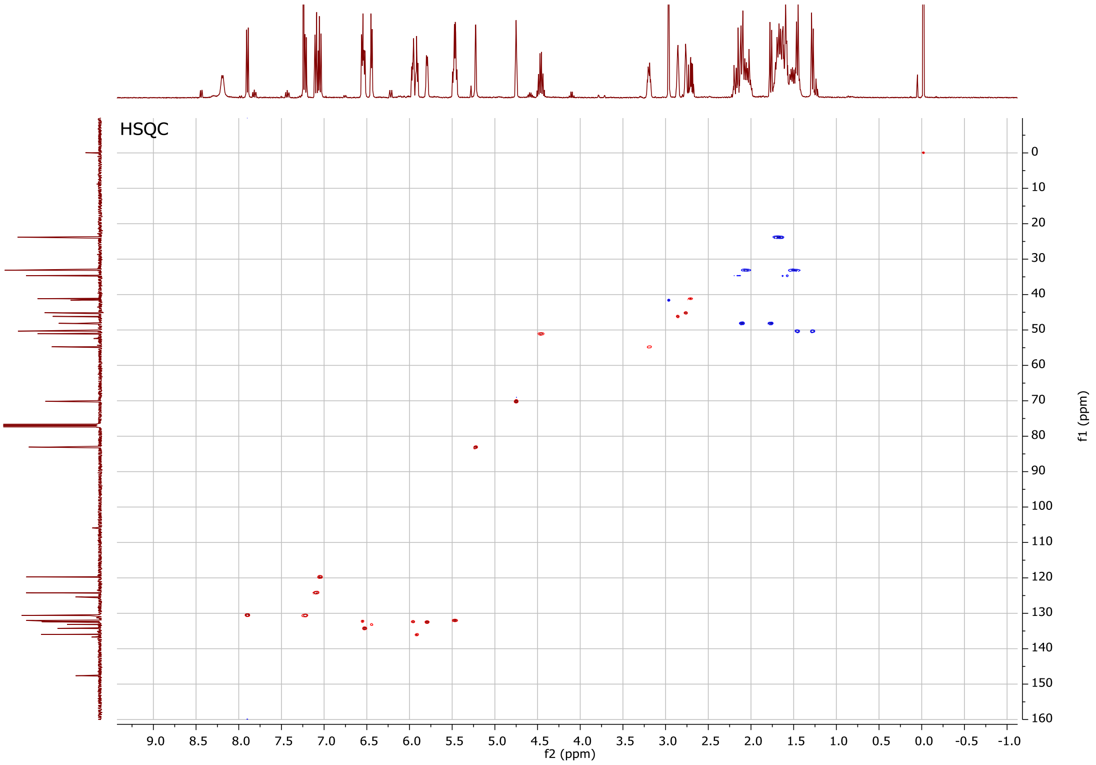
HSQC
f1 (ppm)
f2 (ppm)

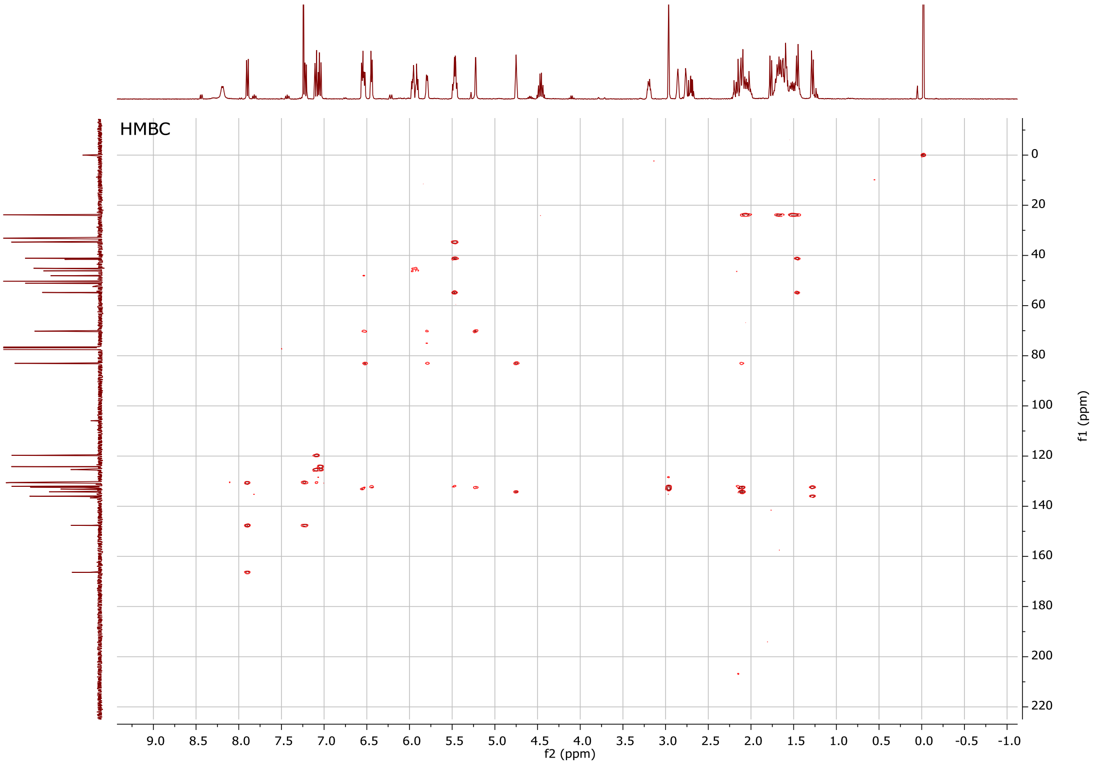
HMBC
f1 (ppm)
f2 (ppm)

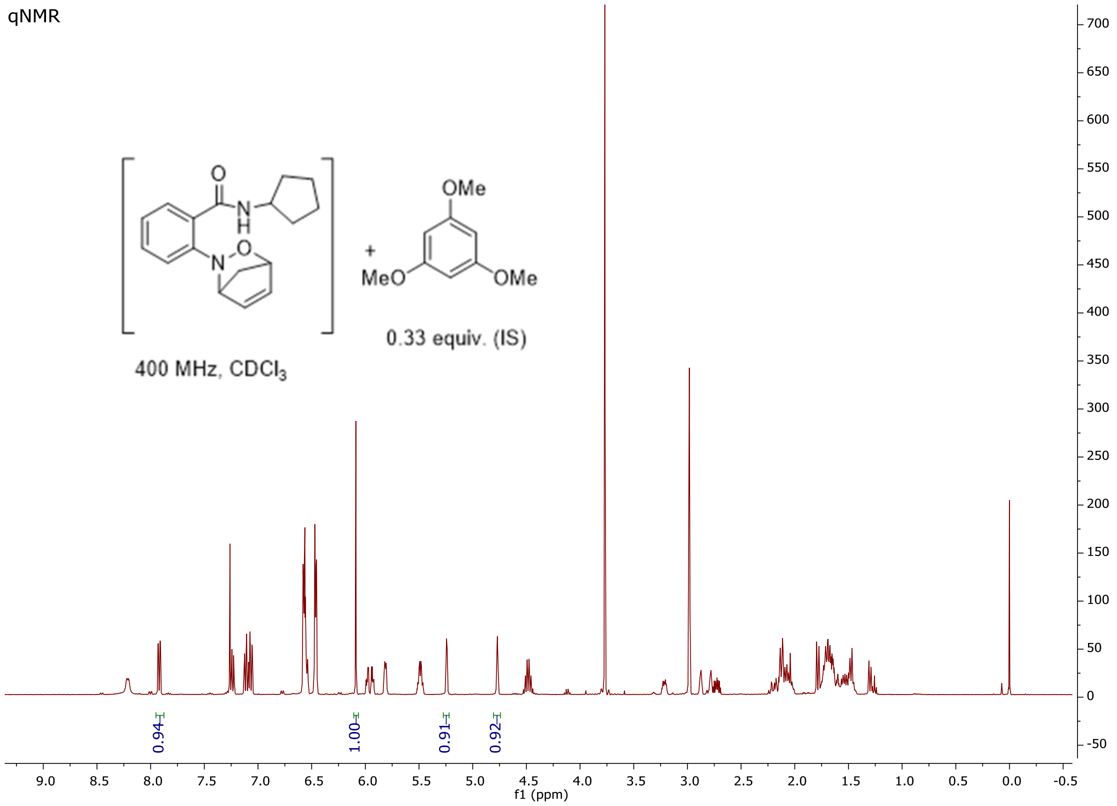

qNMR
OMe
MeO
OMe
0.33 equiv. (IS)
400 MHz, CDCl3
0.94
1.00
0.91
0.92
f1 (ppm)

9a: diethyl (2-(cyclopentylcarbamoyl)phenyl)phosphoramidate

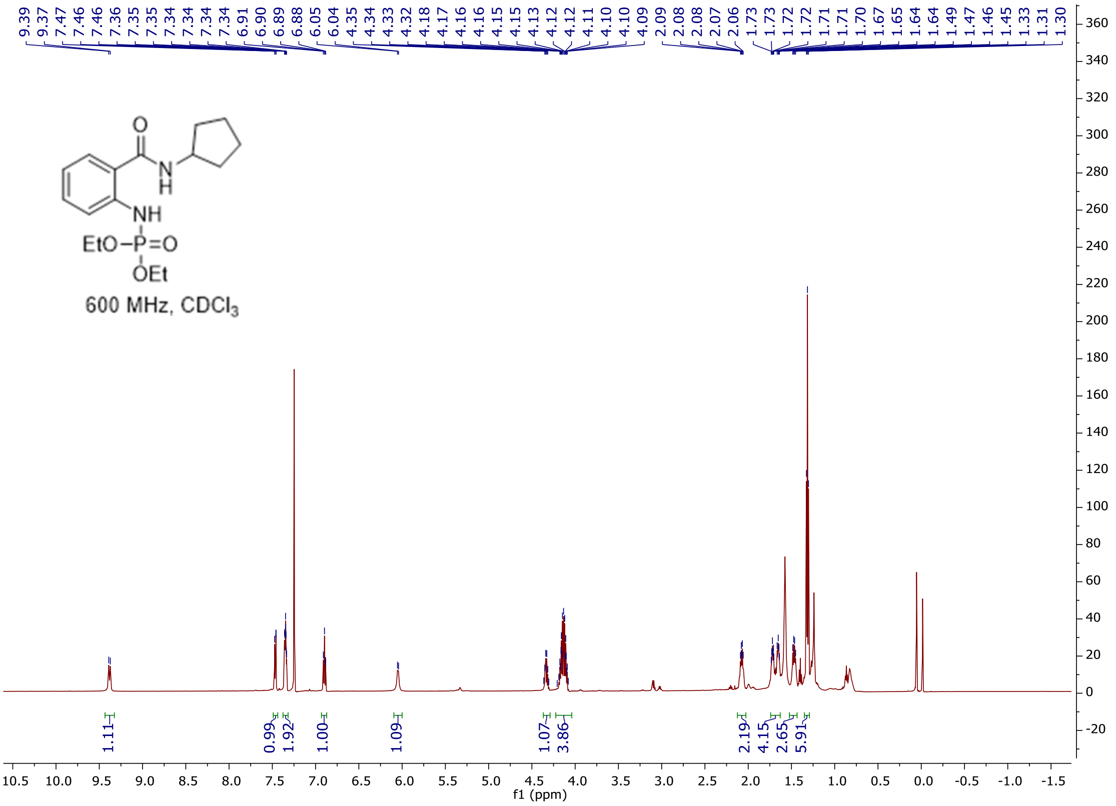

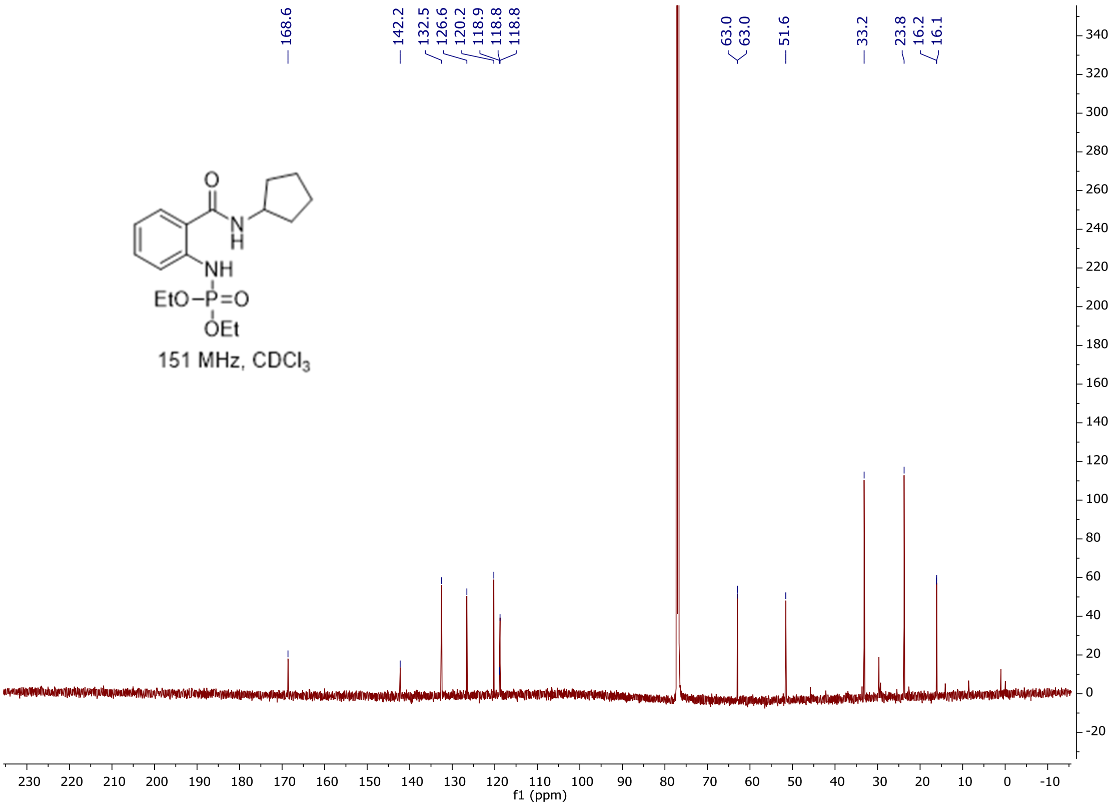

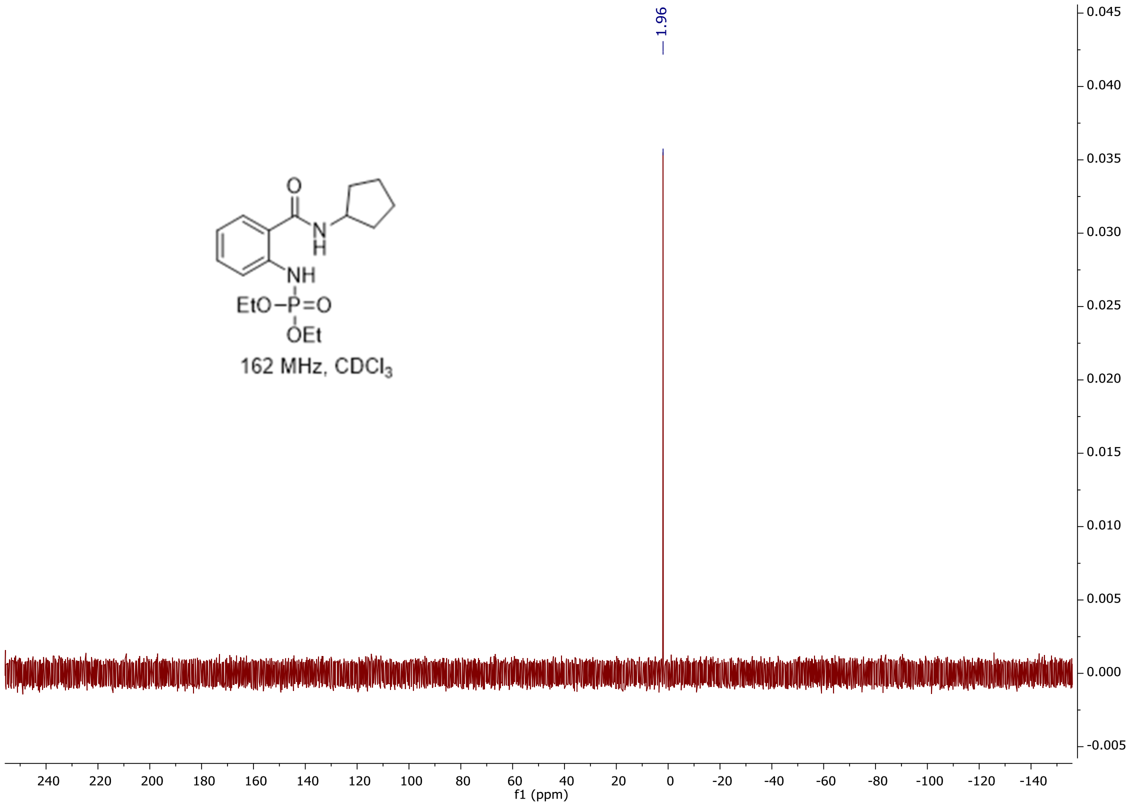
1.96
NH
EtO–P=O
OEt
162 MHz, CDCl3
f1 (ppm)

9b: diethyl (2-(isobutylcarbamoyl)phenyl)phosphoramidate

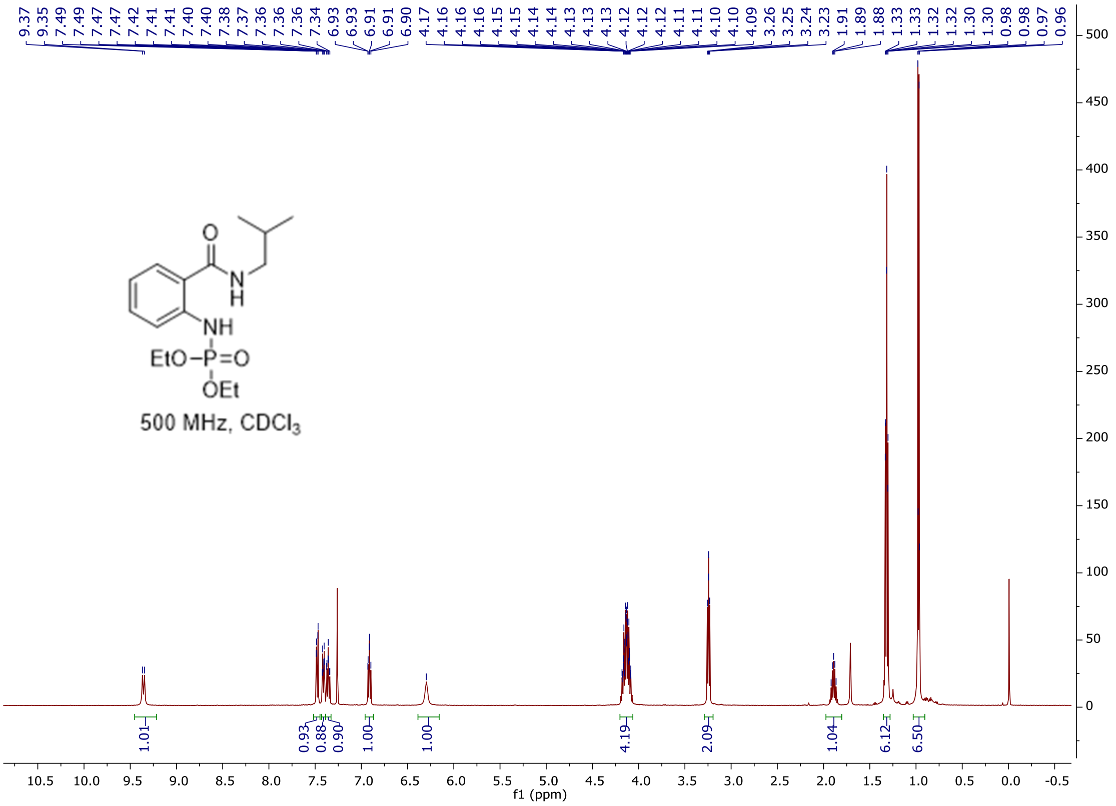

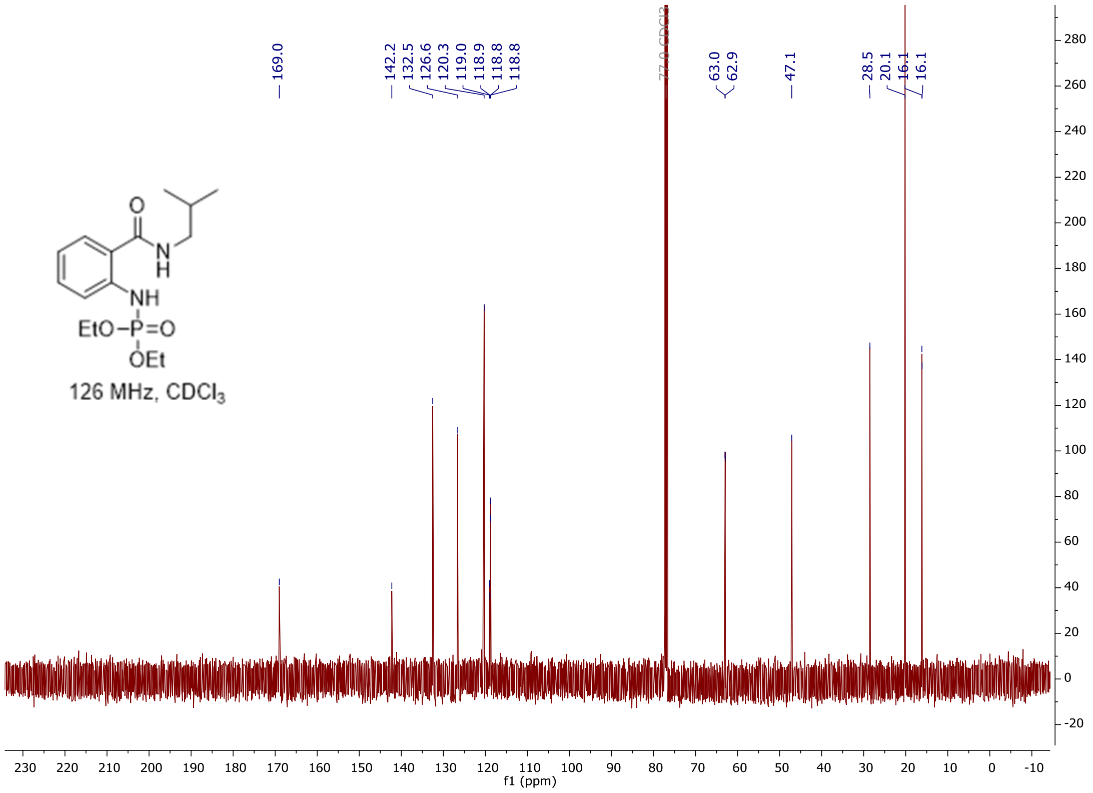

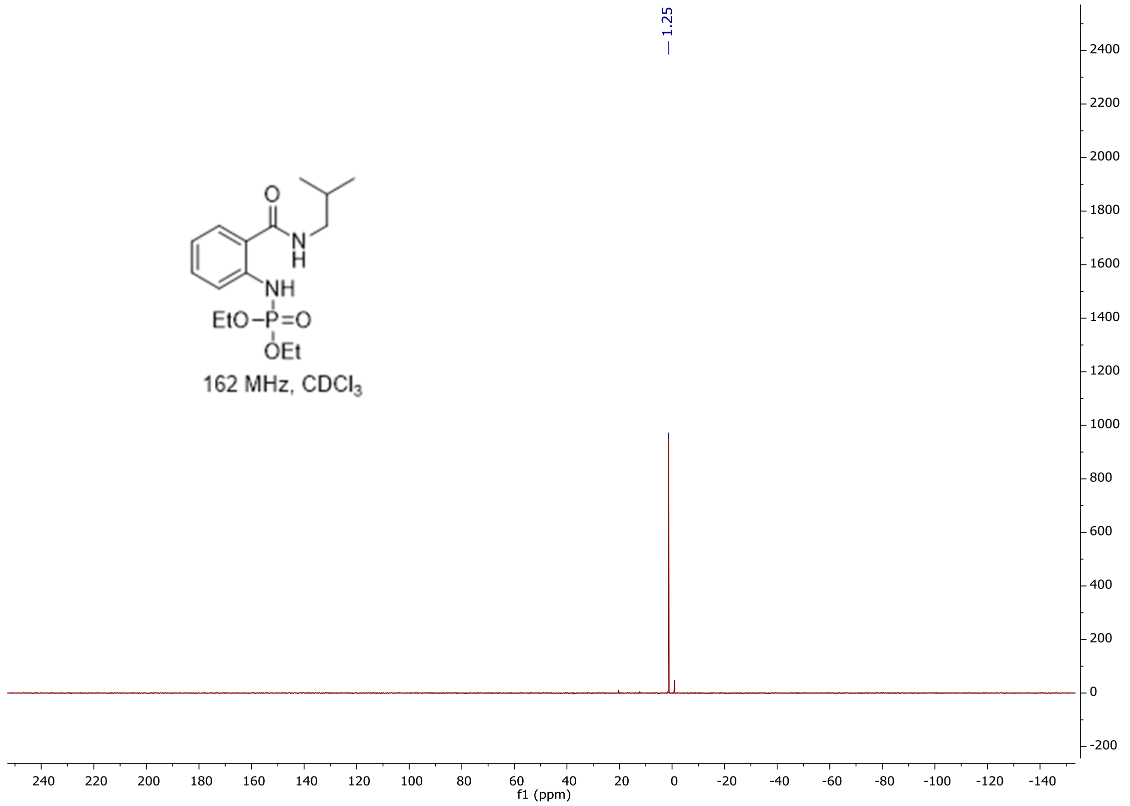

1.25
EtO
NH
OEt
162 MHz, CDCl3
f1 (ppm)

NMR copies of starting materials (*o*-nitrophenylimines): **1a-1o**

1a: (*E*)-*N*-isobutyl-1-(2-nitrophenyl)methanimine

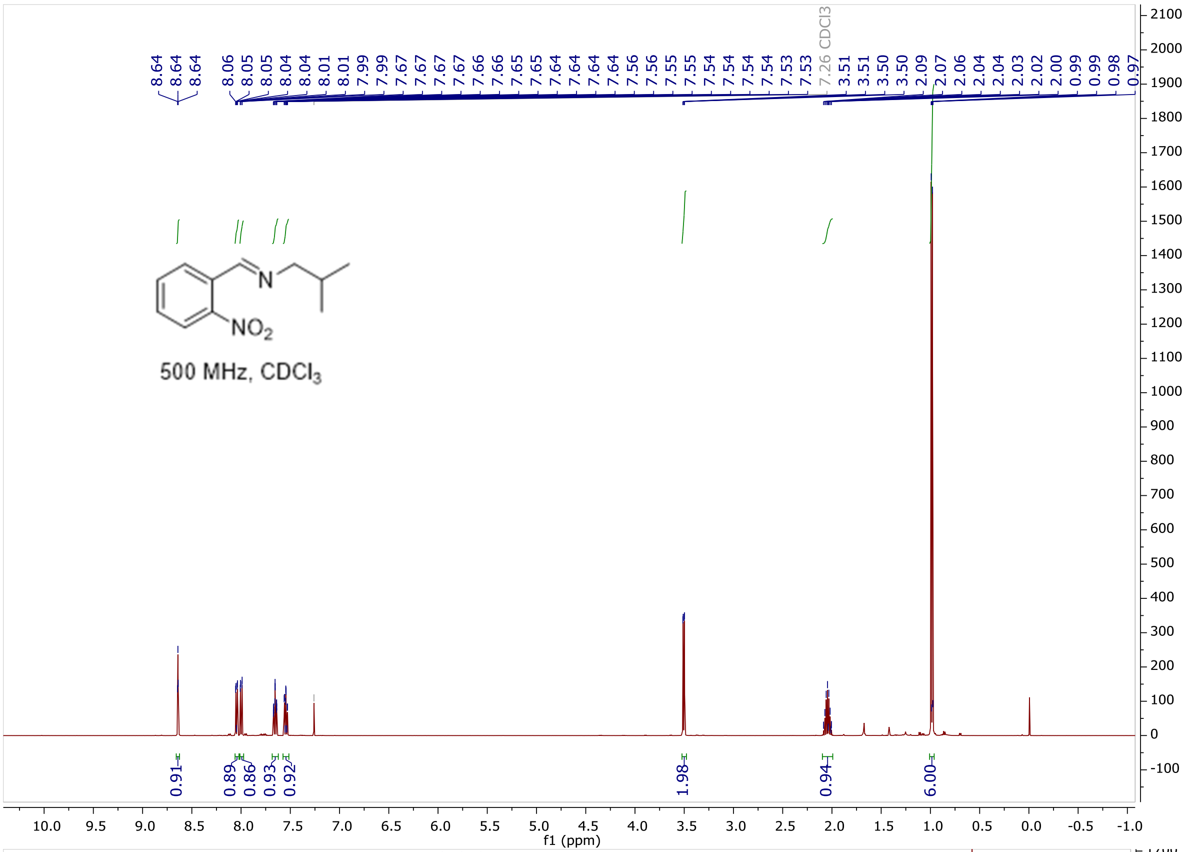

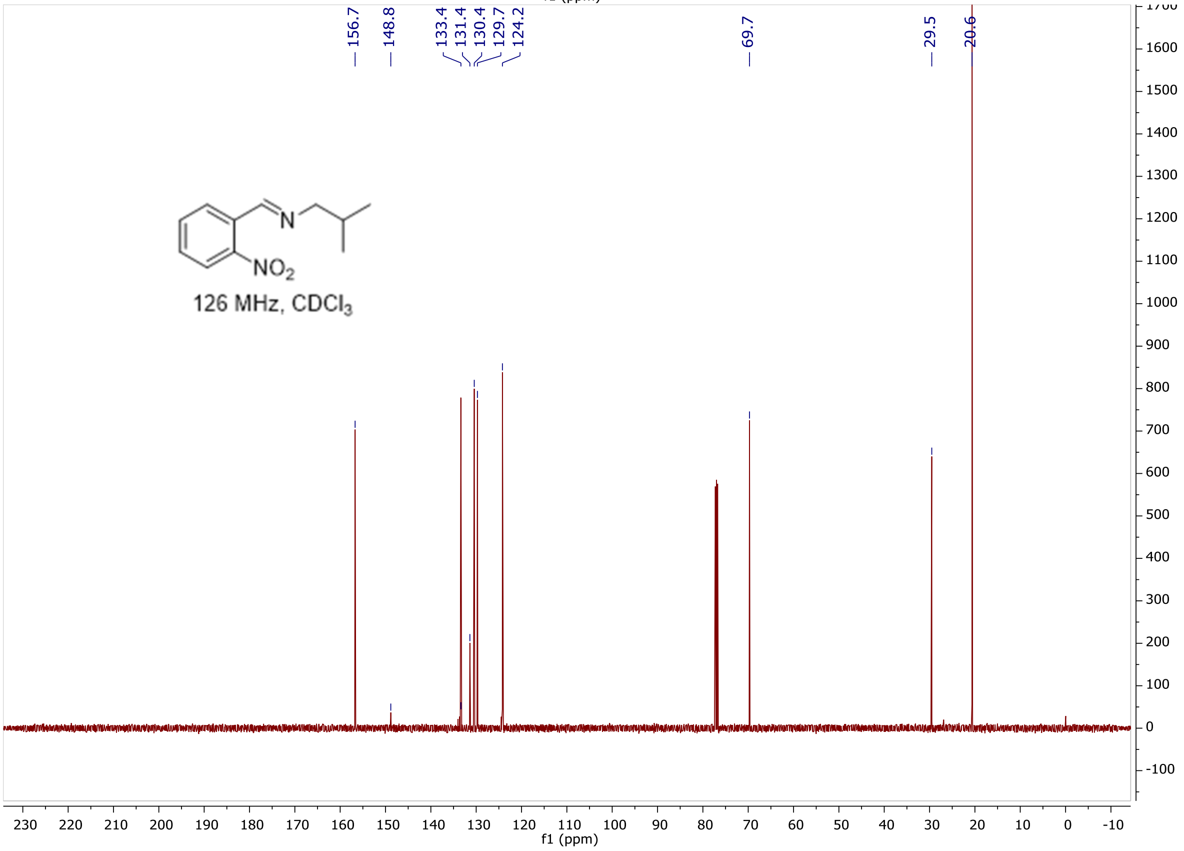

1b: (*E*)-*N*-cyclopentyl-1-(2-nitrophenyl)methanimine

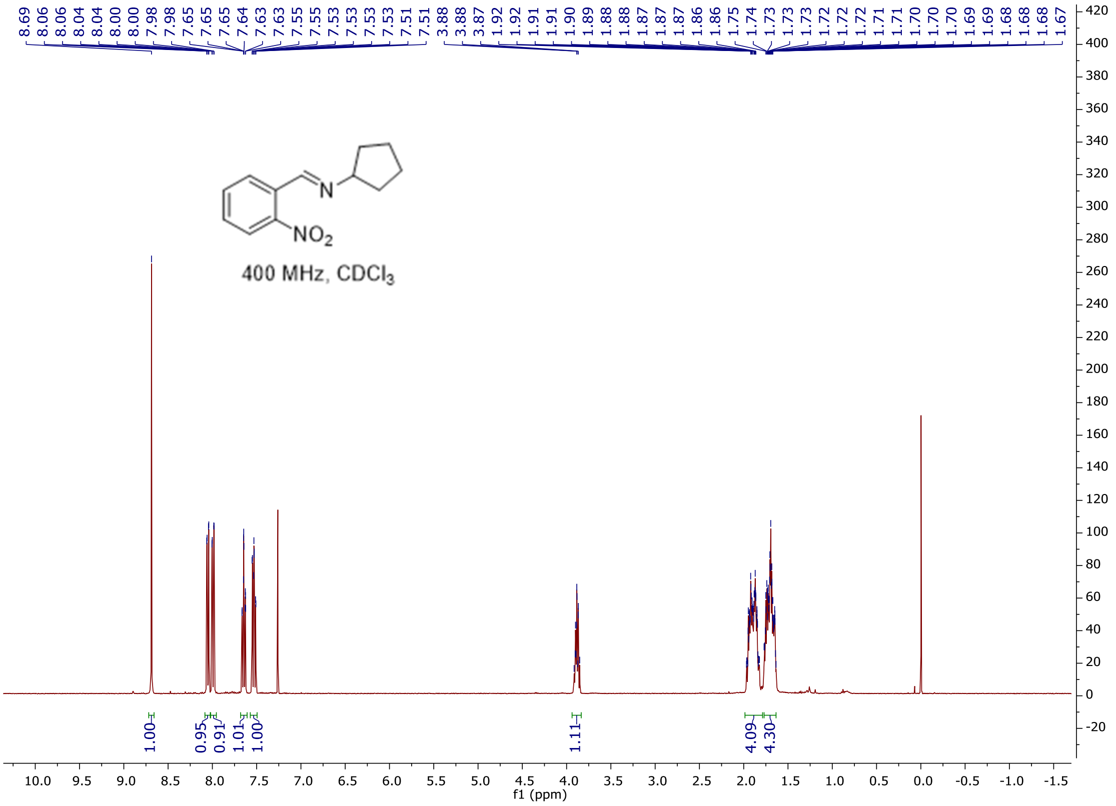

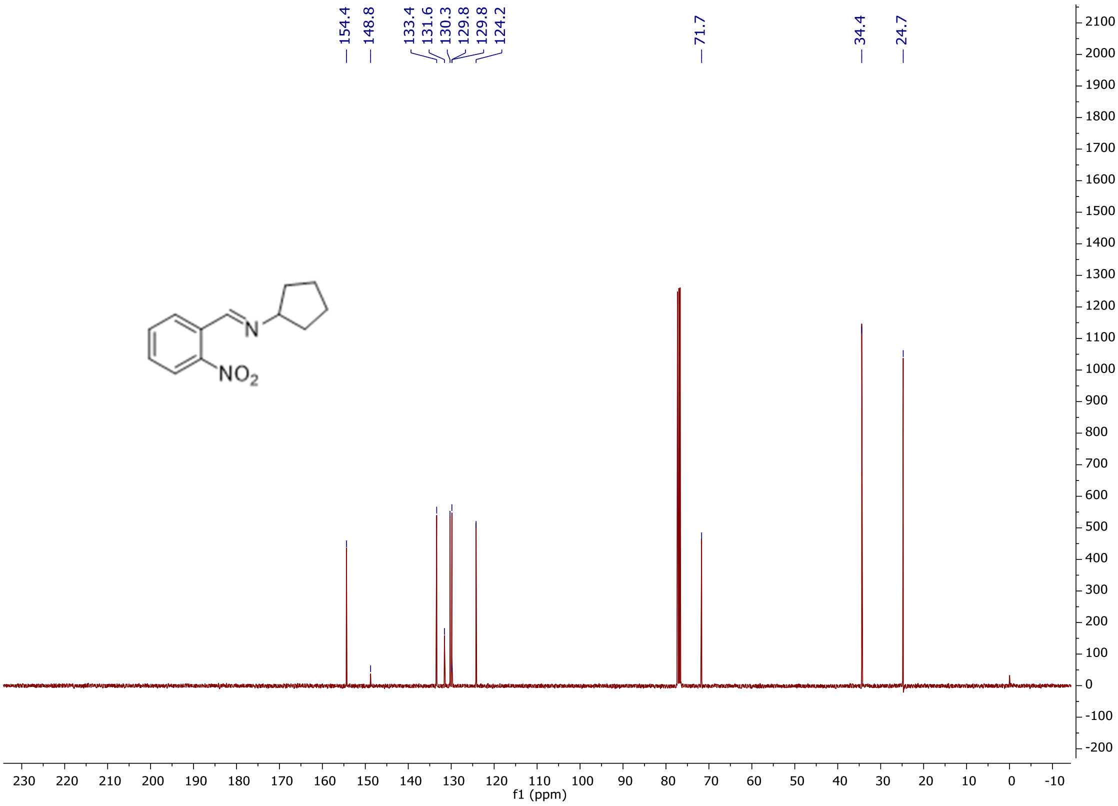

1c: (*E*)-*N*-cyclohexyl-1-(2-nitrophenyl)methanimine

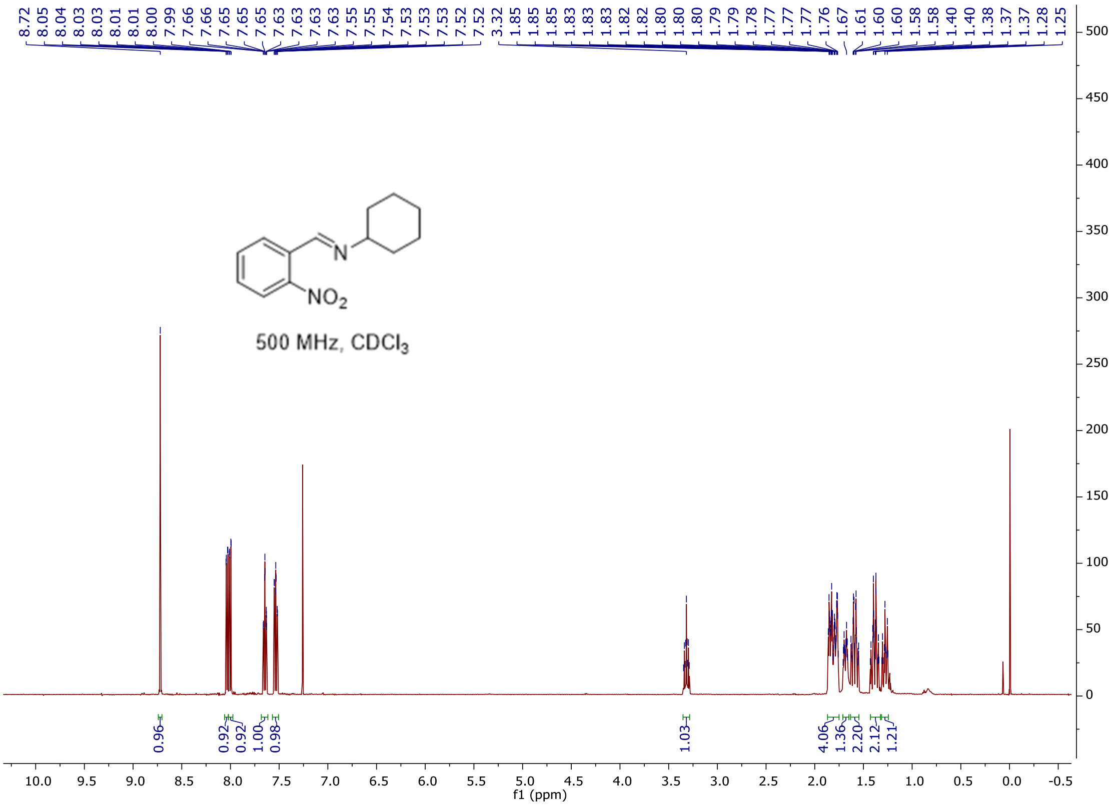

1d: (*E*)-*N*-*tert*-butyl-1-(2-nitrophenyl)methanimine

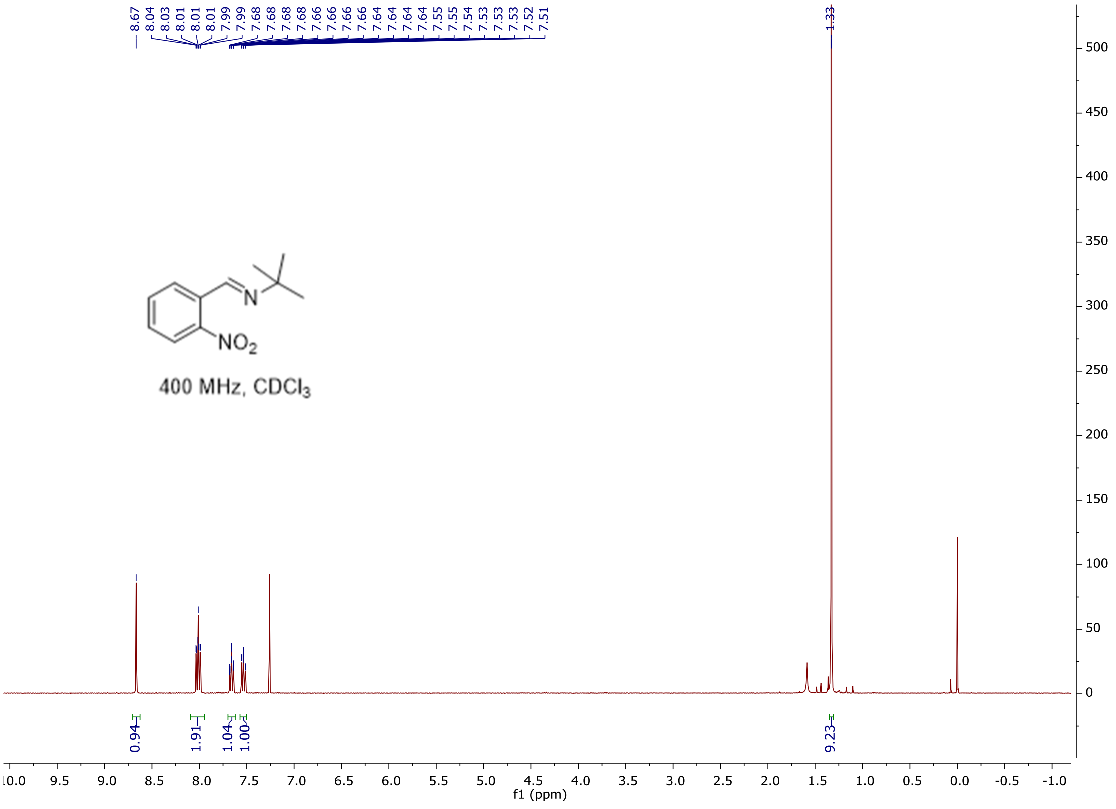

1e: (*E*)-*N*-decyl-1-(2-nitrophenyl)methanimine

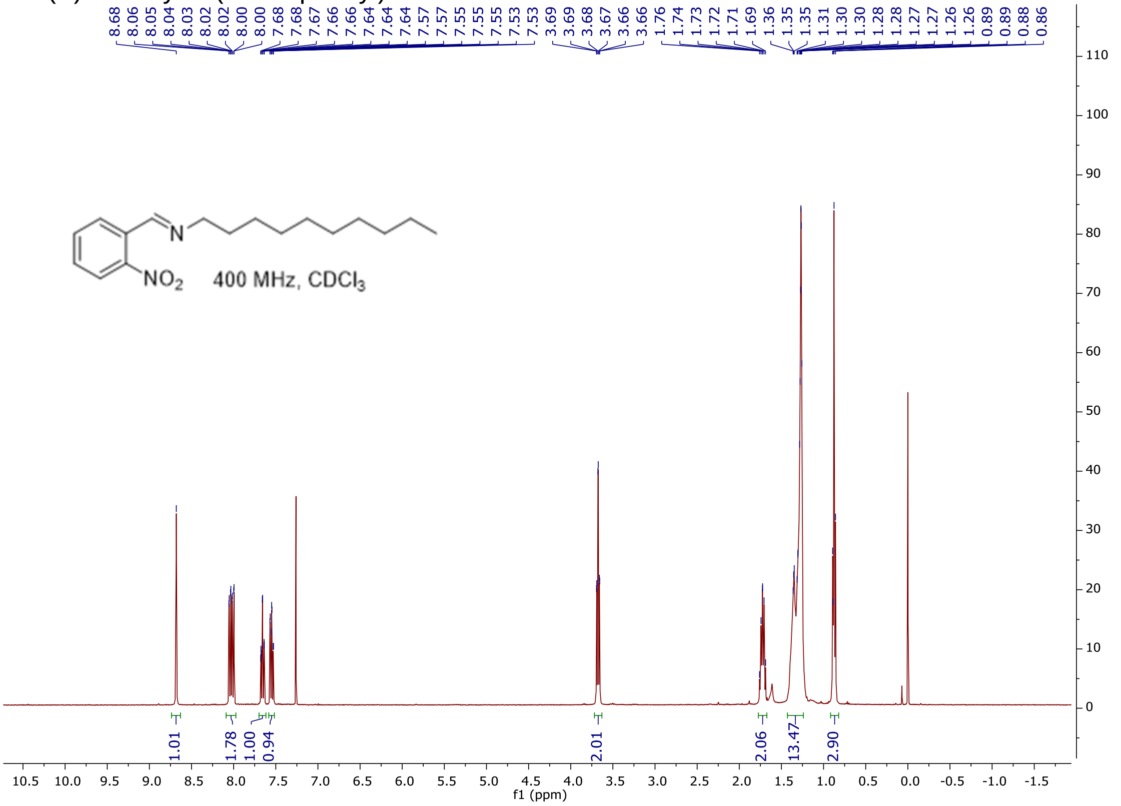

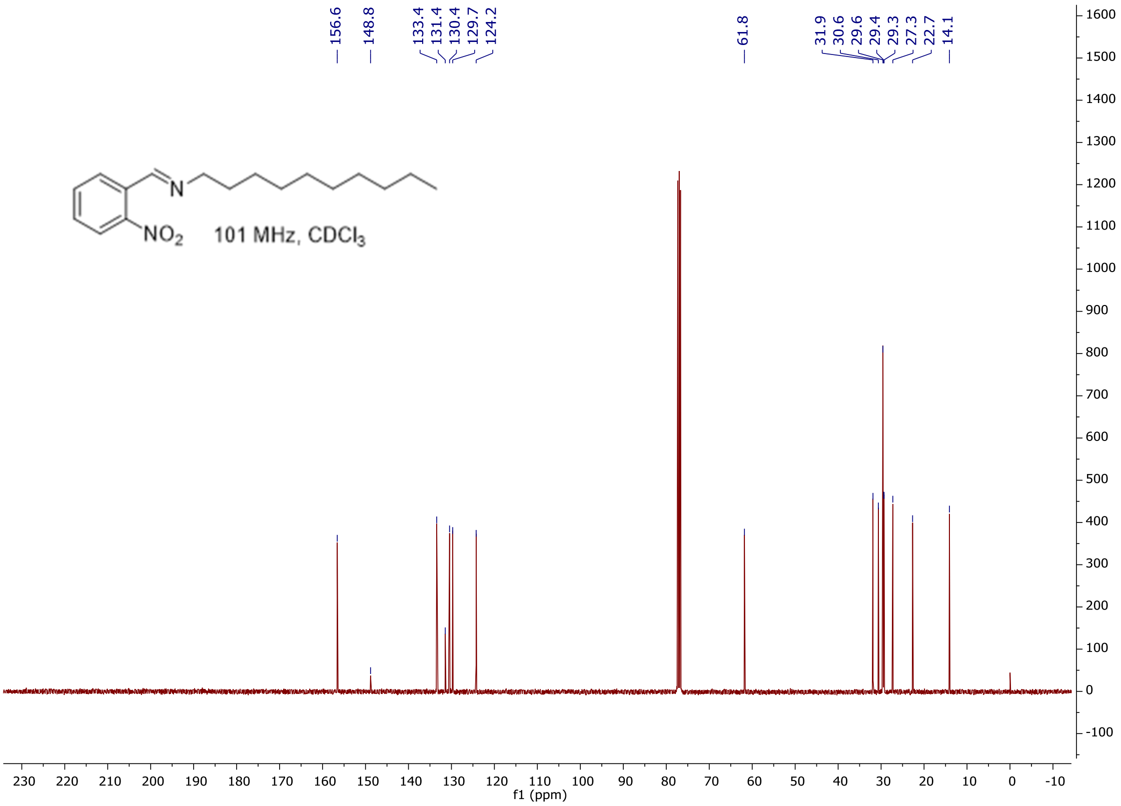

1f: (*E*)-*N*-(2-methoxybenzyl)-1-(2-nitrophenyl)methanimine

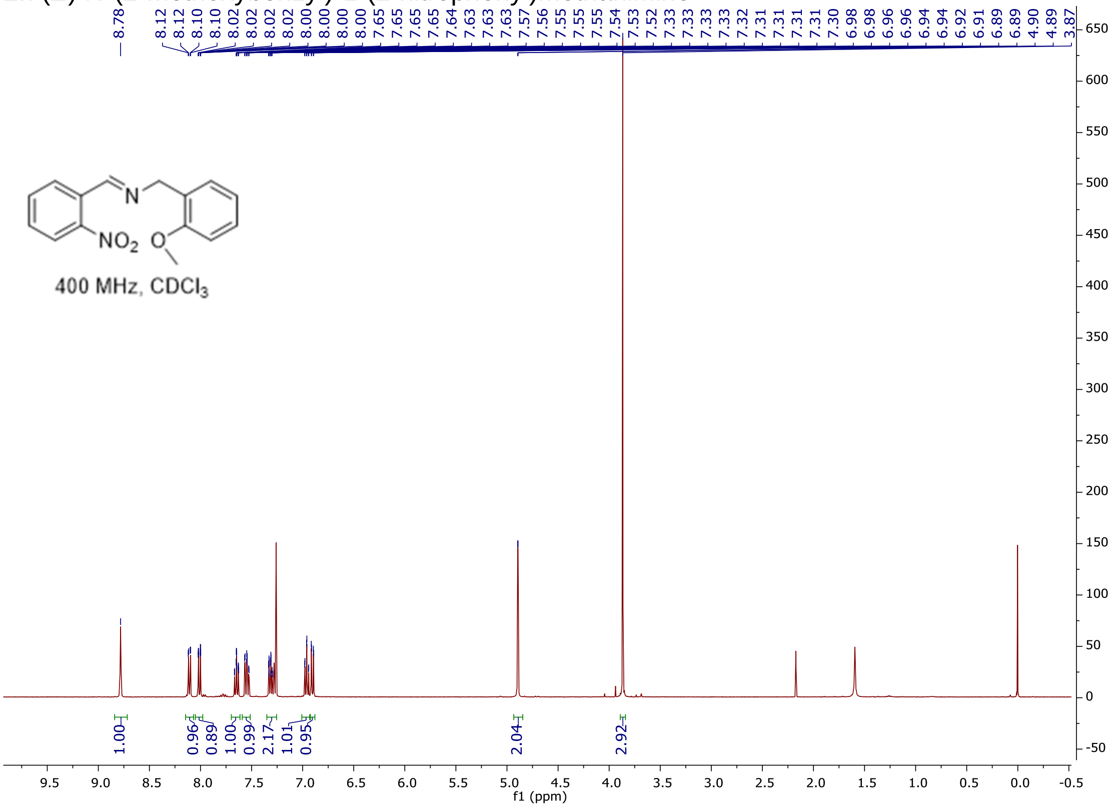

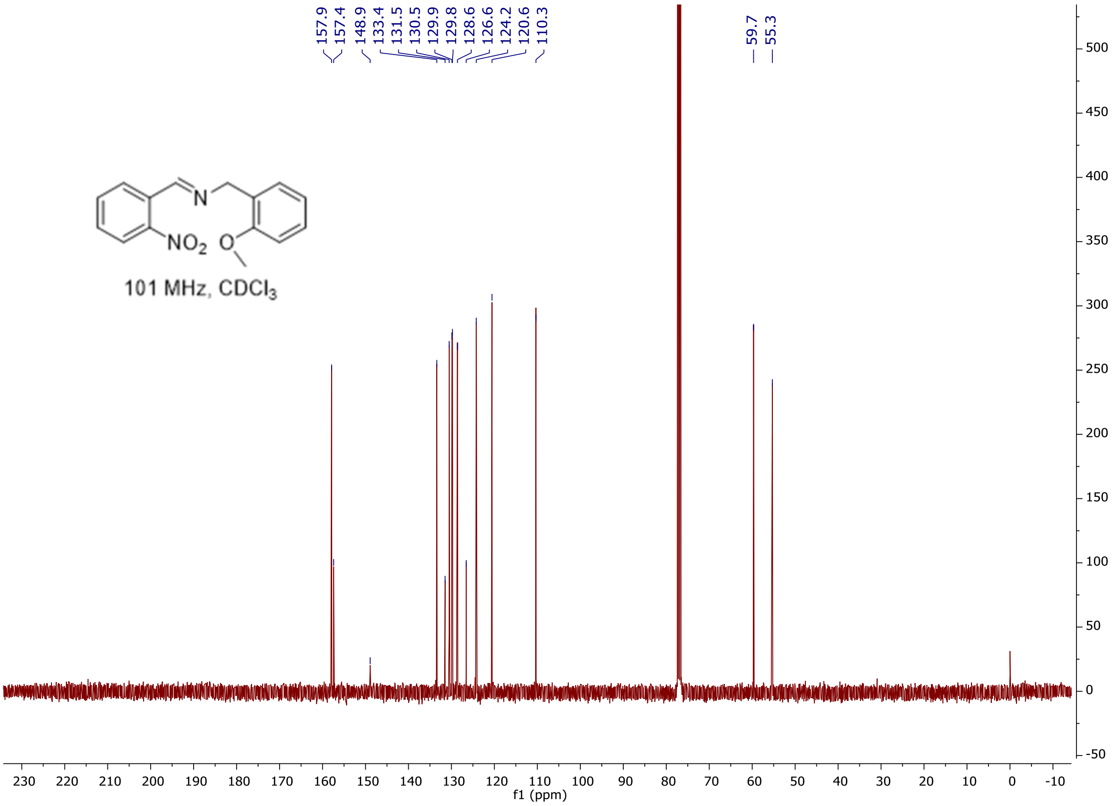

1g: (*E*)-*N*-(4-fluorobenzyl)-1-(2-nitrophenyl)methanimine

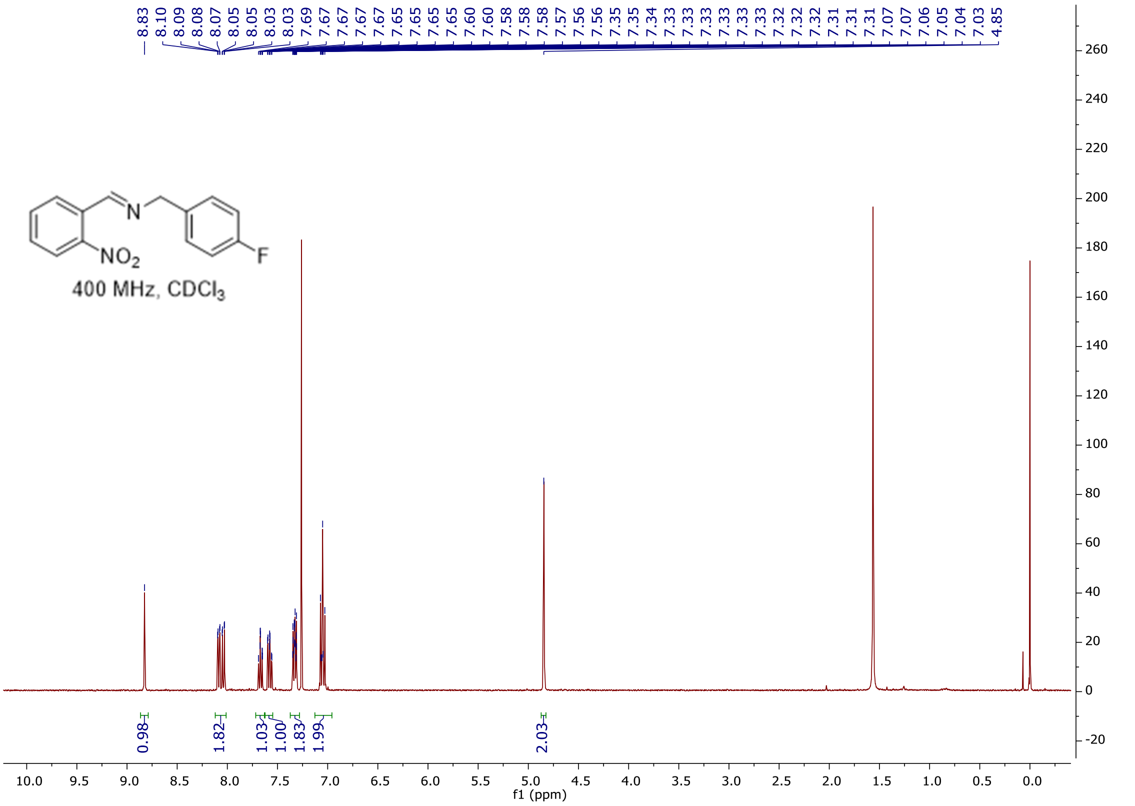

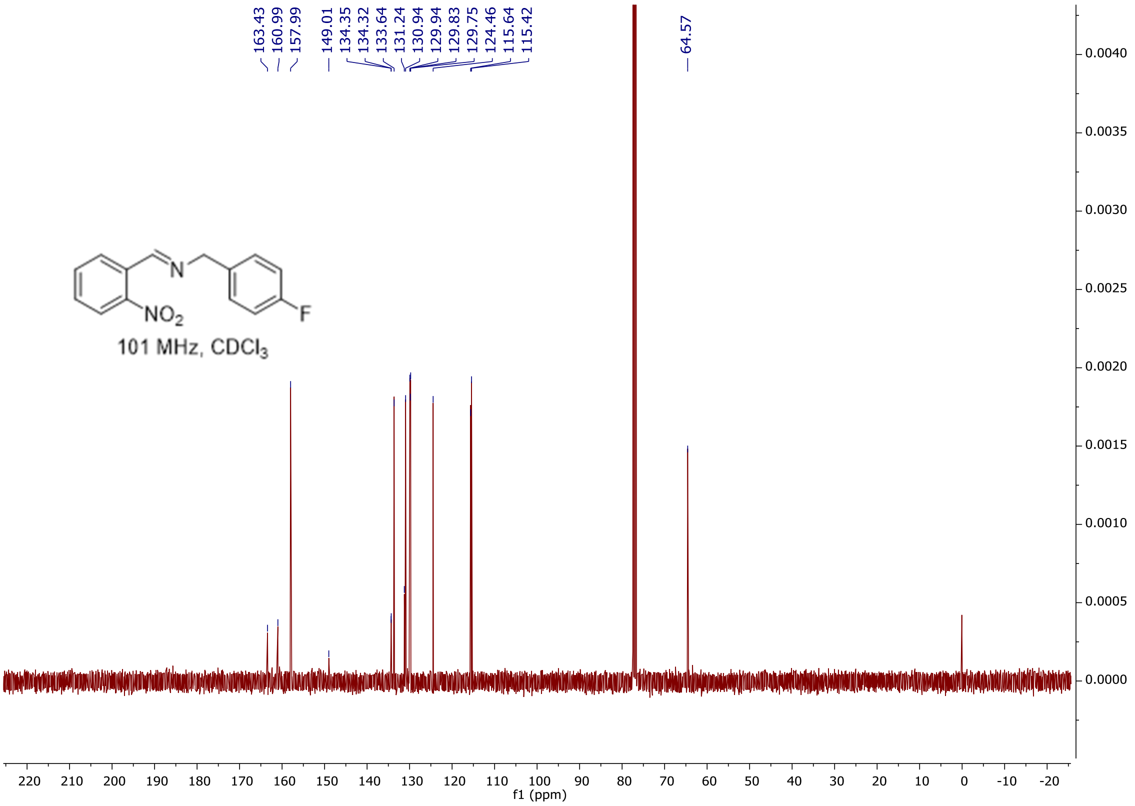

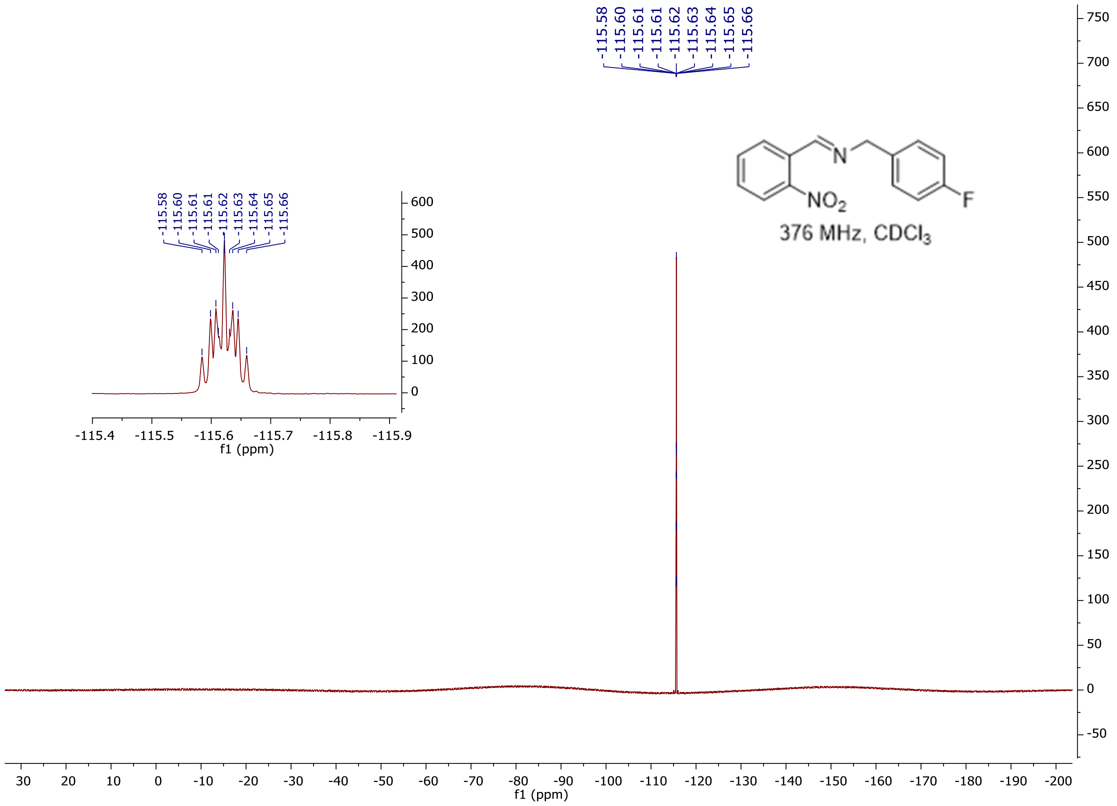

-115.58
-115.60
-115.61
-115.61
-115.62
-115.63
-115.64
-115.65
-115.66
NO2
F
N
376 MHz, CDCl3
f1 (ppm)

1h: methyl (*E*)-2-((2-nitrobenzylidene)amino)-3-phenylpropanoate

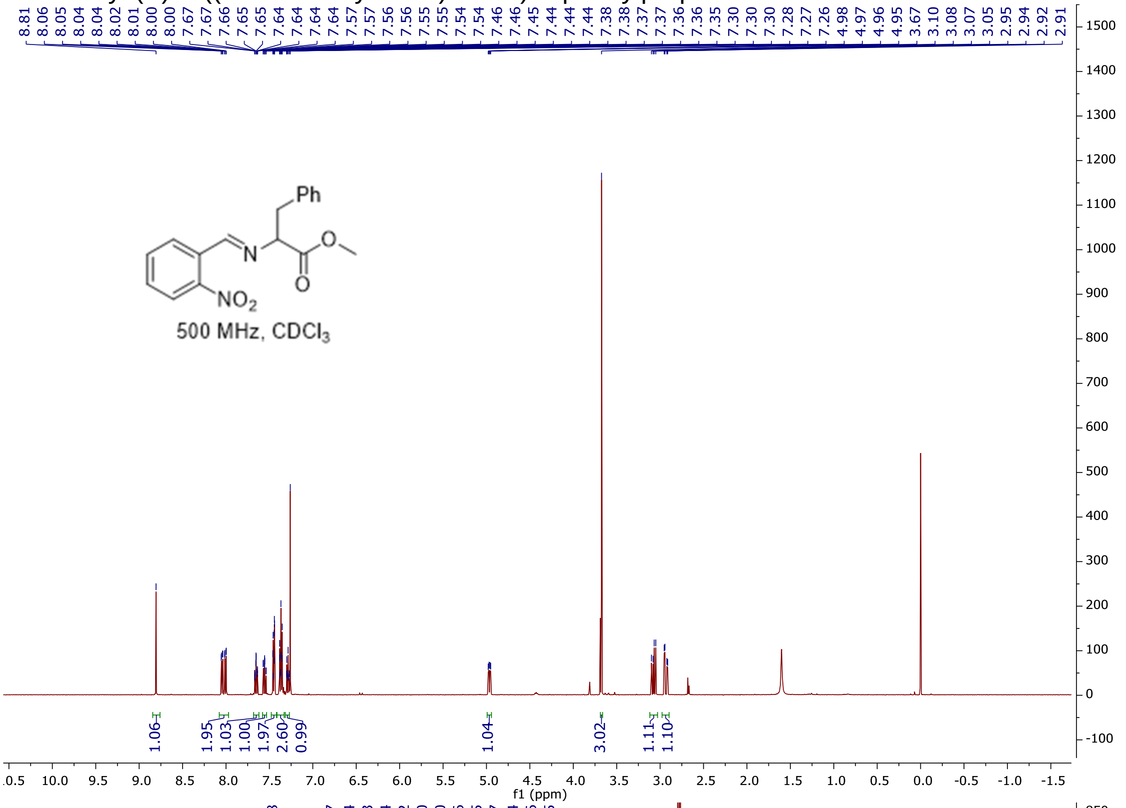

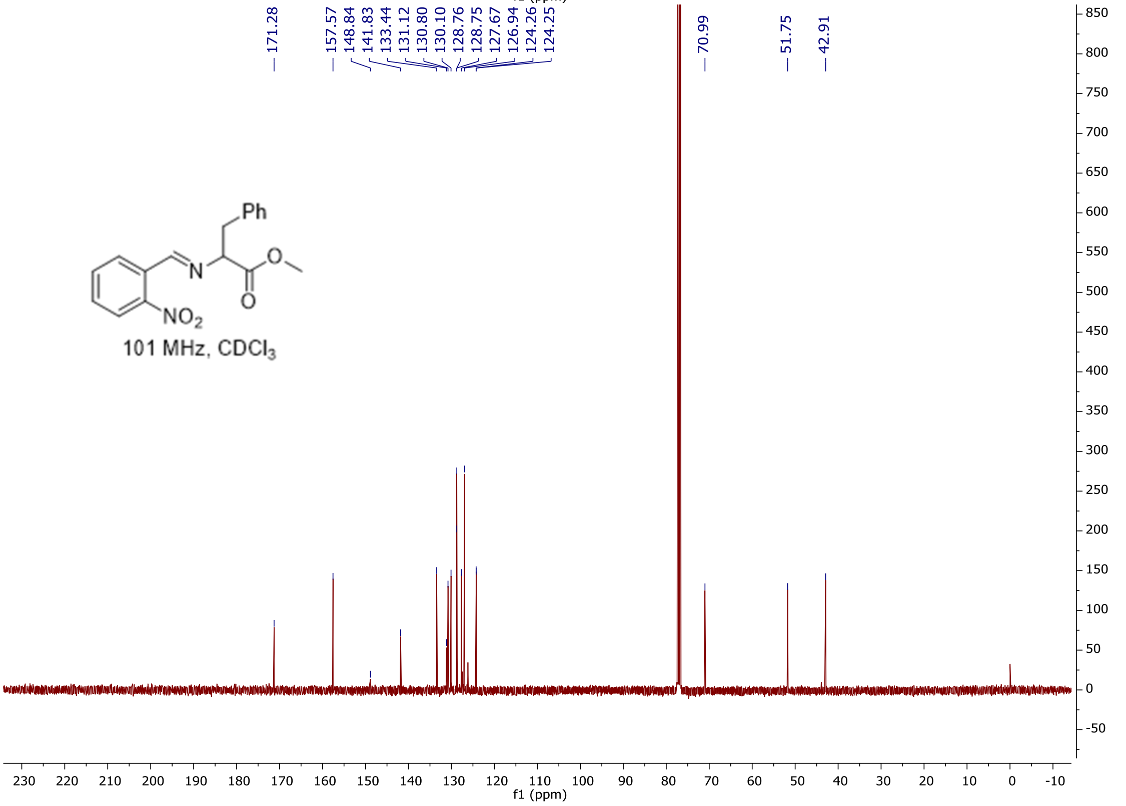

1i: (*E*)-1-(2-nitrophenyl)-N-phenylmethanimine

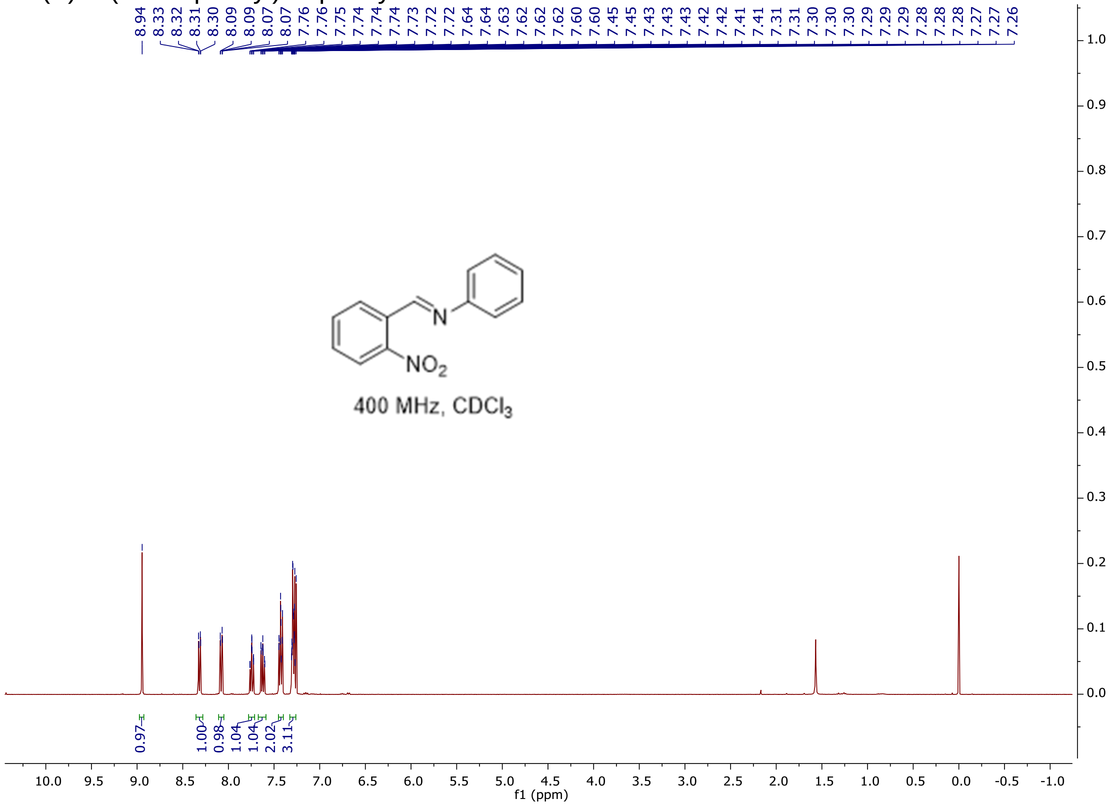

1j: (*E*)-*N*-(4-fluorophenyl)-1-(2-nitrophenyl)methanimine

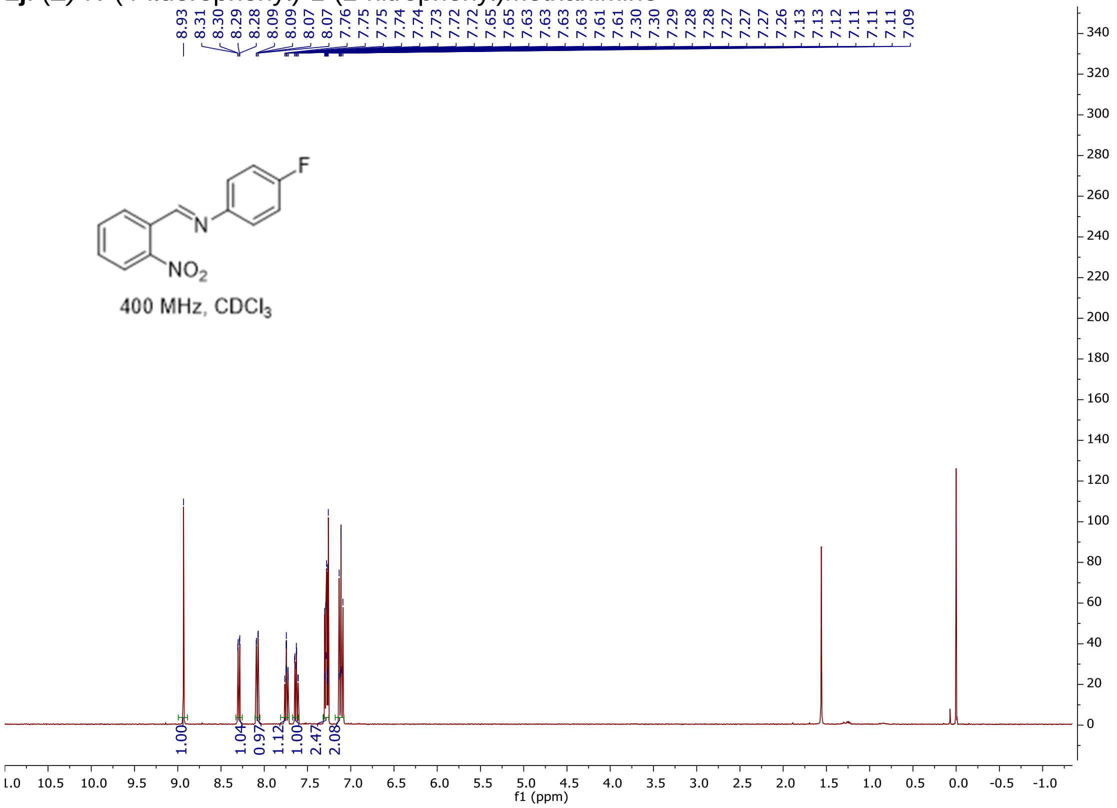

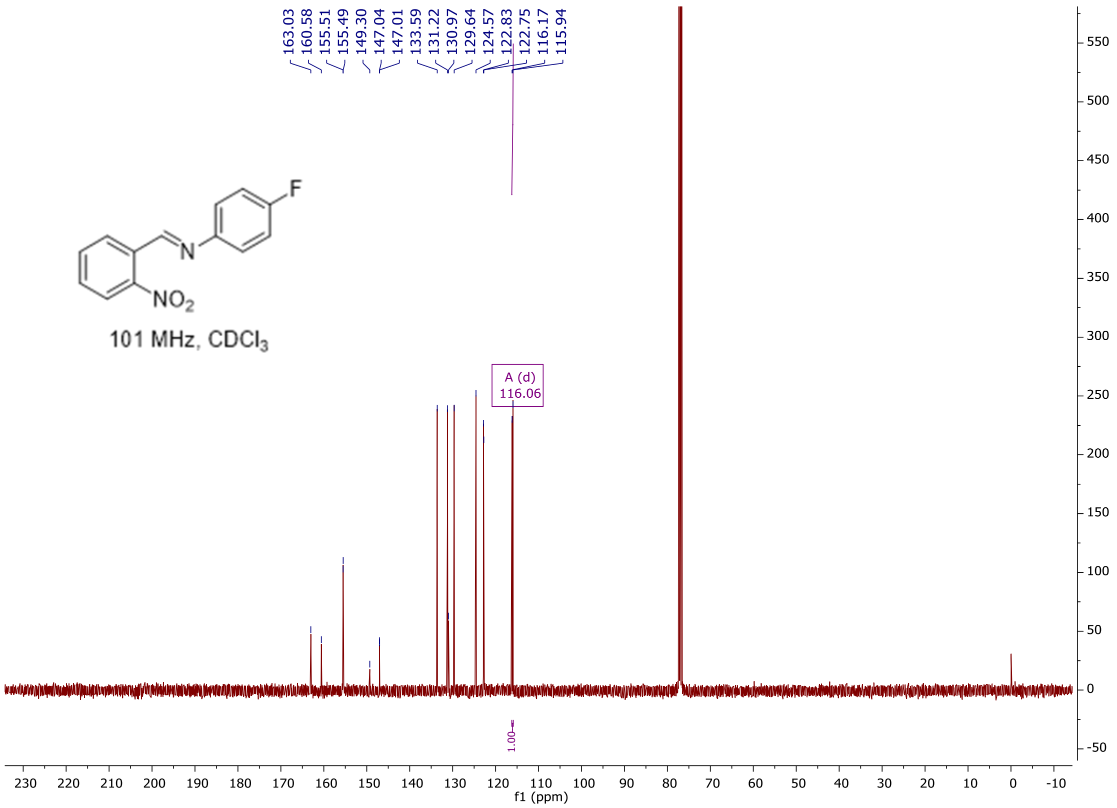

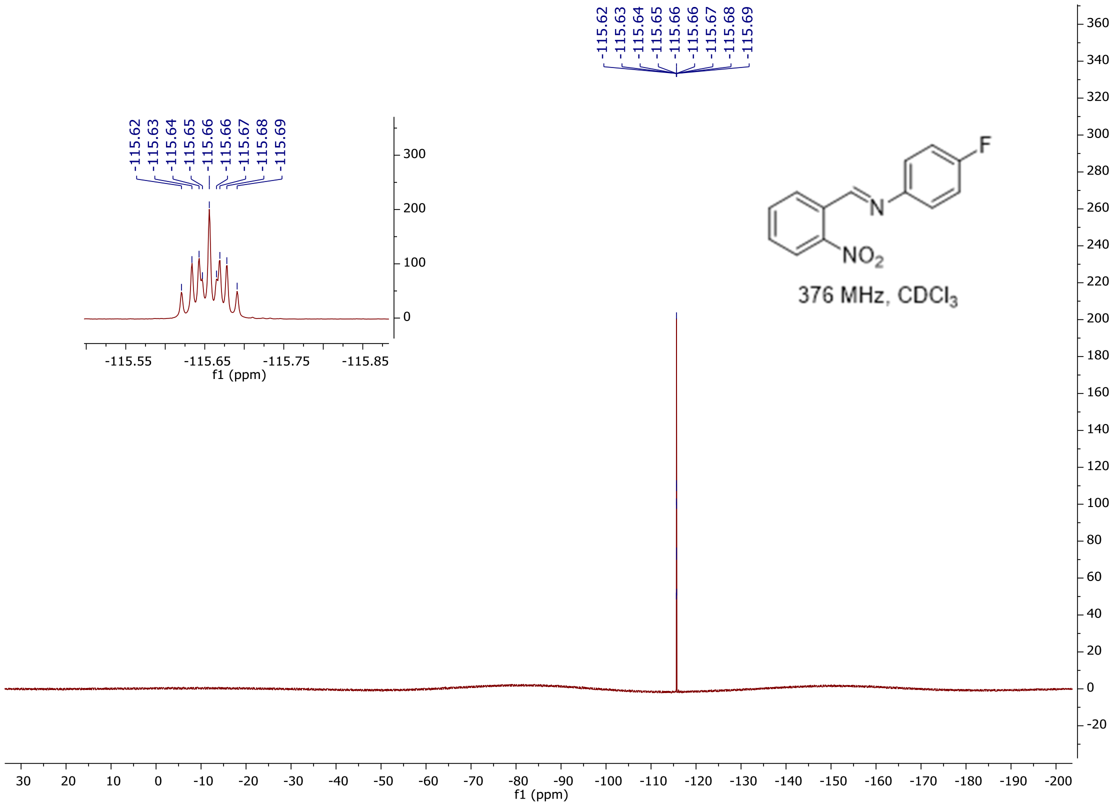

1k: (*E*)-*N*-(4-isopropylphenyl)-1-(2-nitrophenyl)methanimine

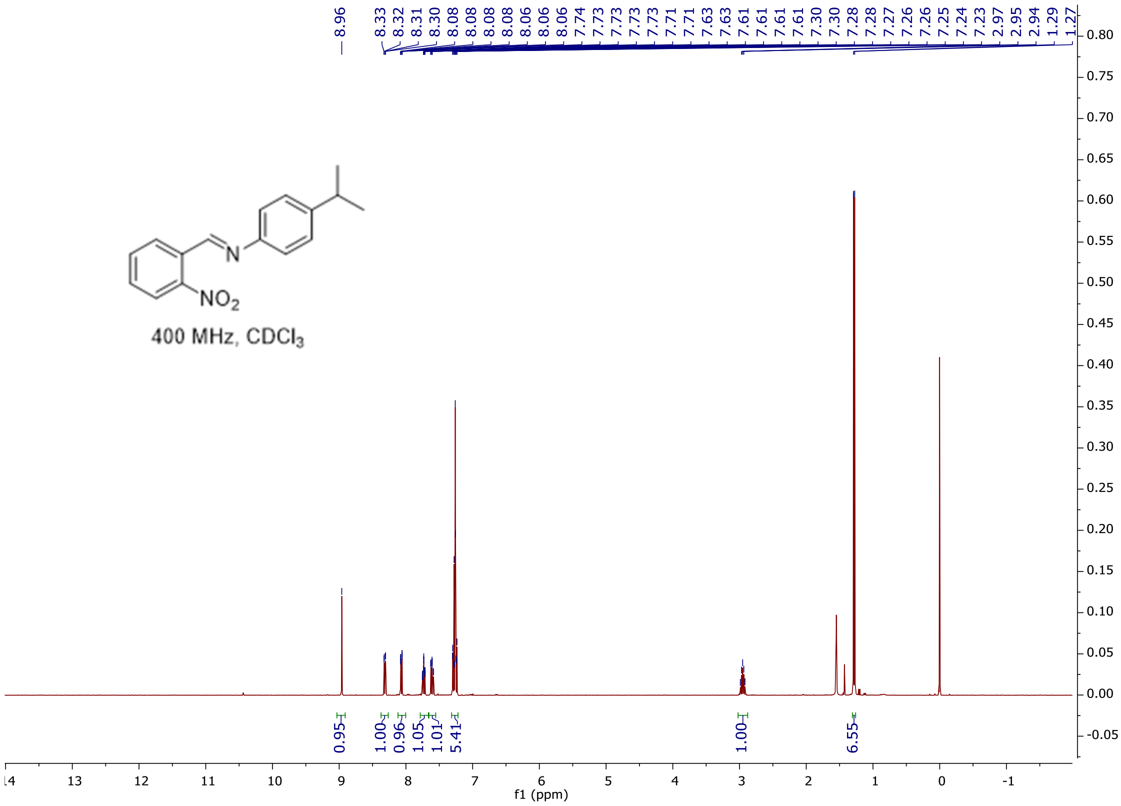

1 l: (*E*)-*N*-cyclopentyl-1-(5-fluoro-2-nitrophenyl)methanimine

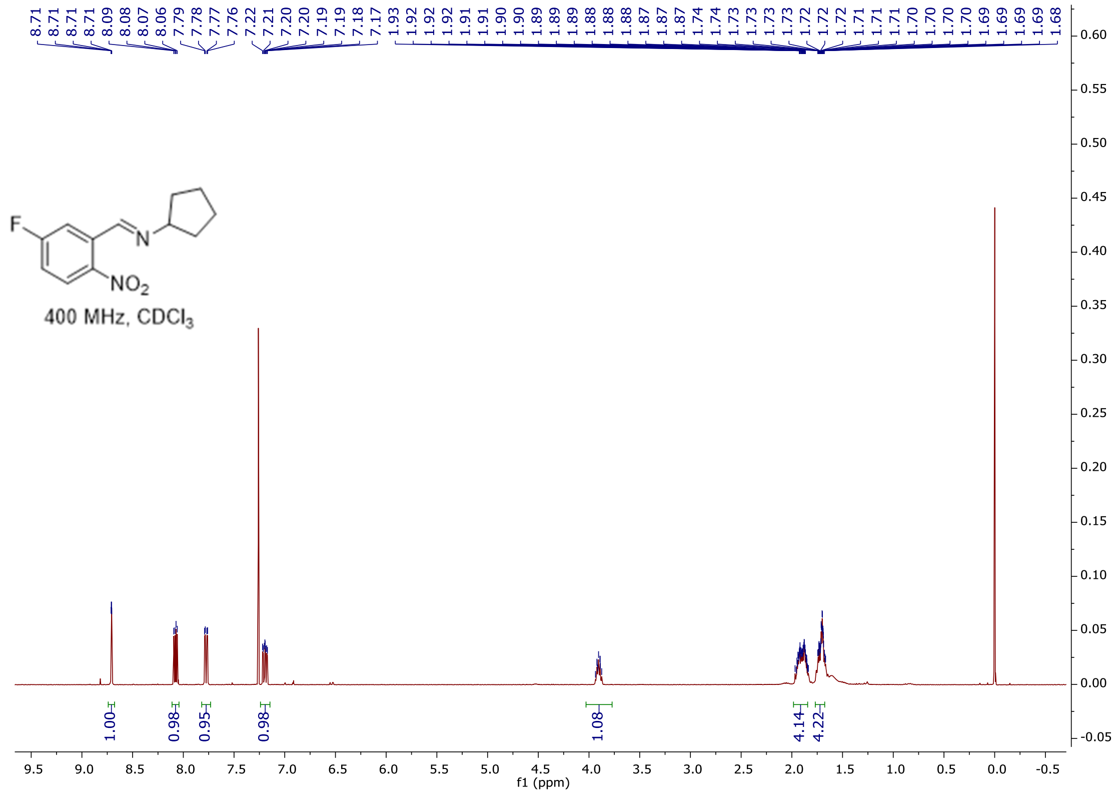

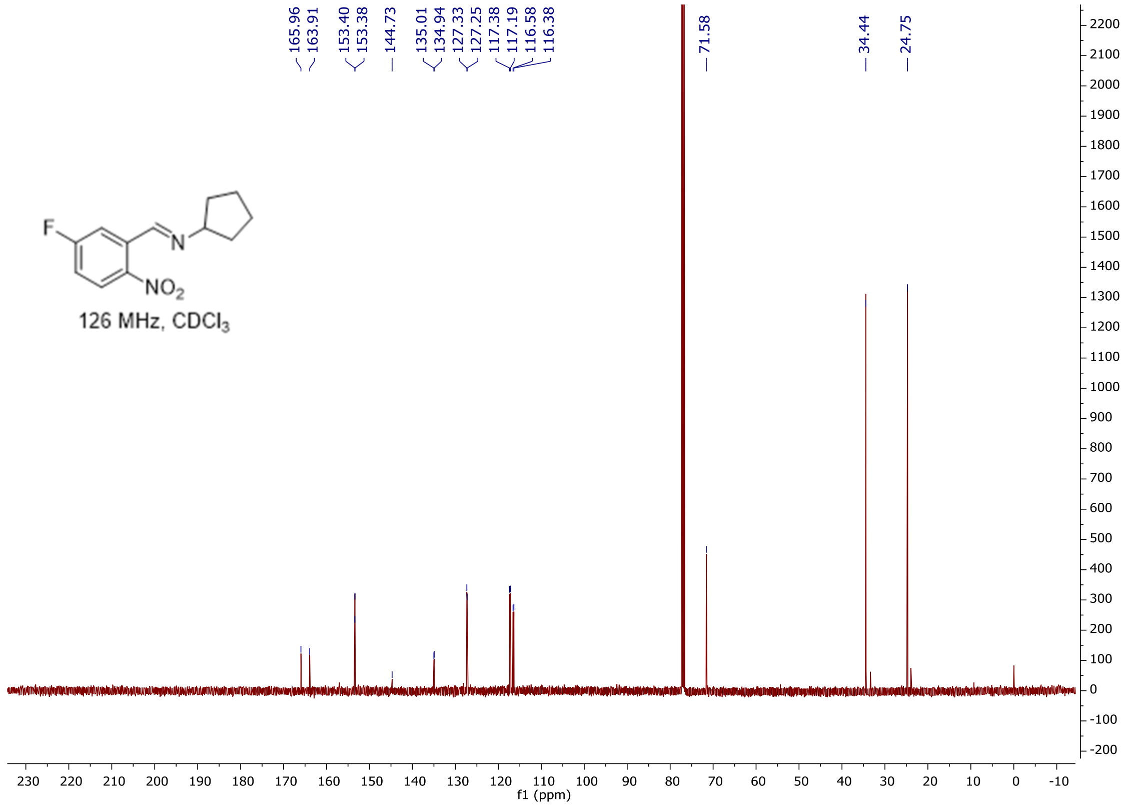

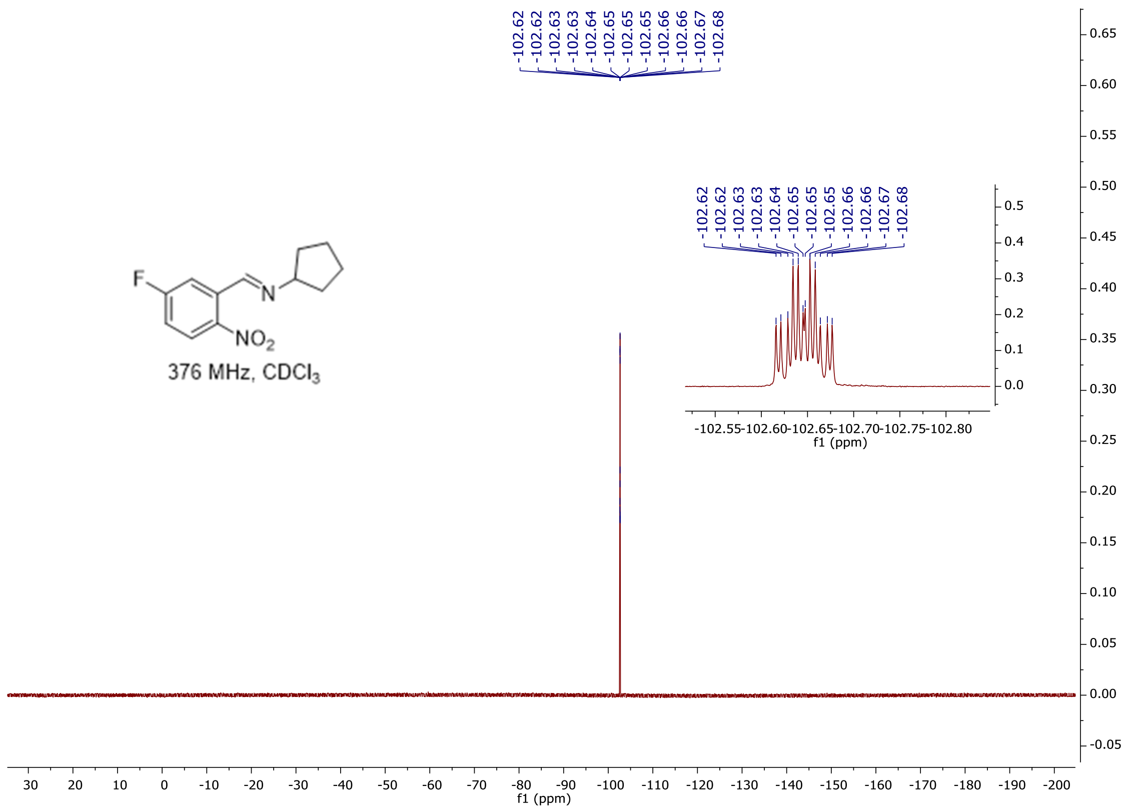

-102.62
-102.62
-102.63
-102.63
-102.64
-102.65
-102.65
-102.65
-102.66
-102.66
-102.67
-102.68
376 MHz, CDCl3
f1 (ppm)

1m: (*E*)-*N*-cyclopentyl-1-(6-nitrobenzo[d][1,3]dioxol-5-yl)methanimine

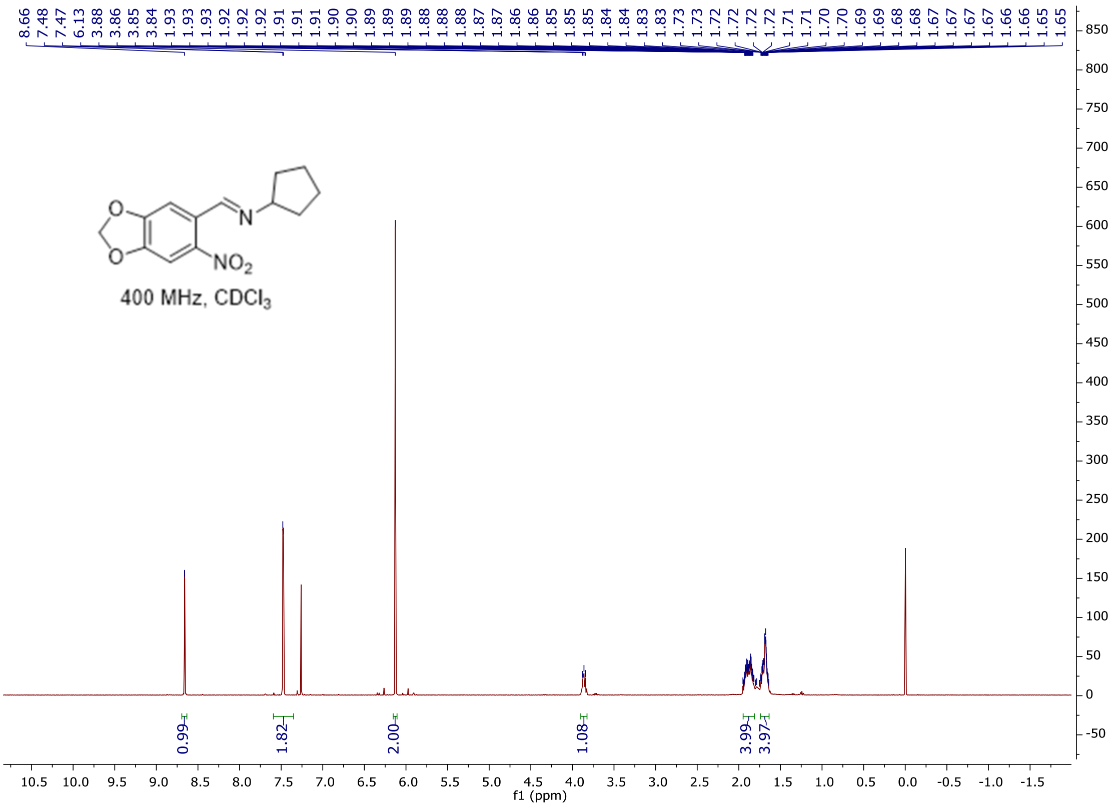

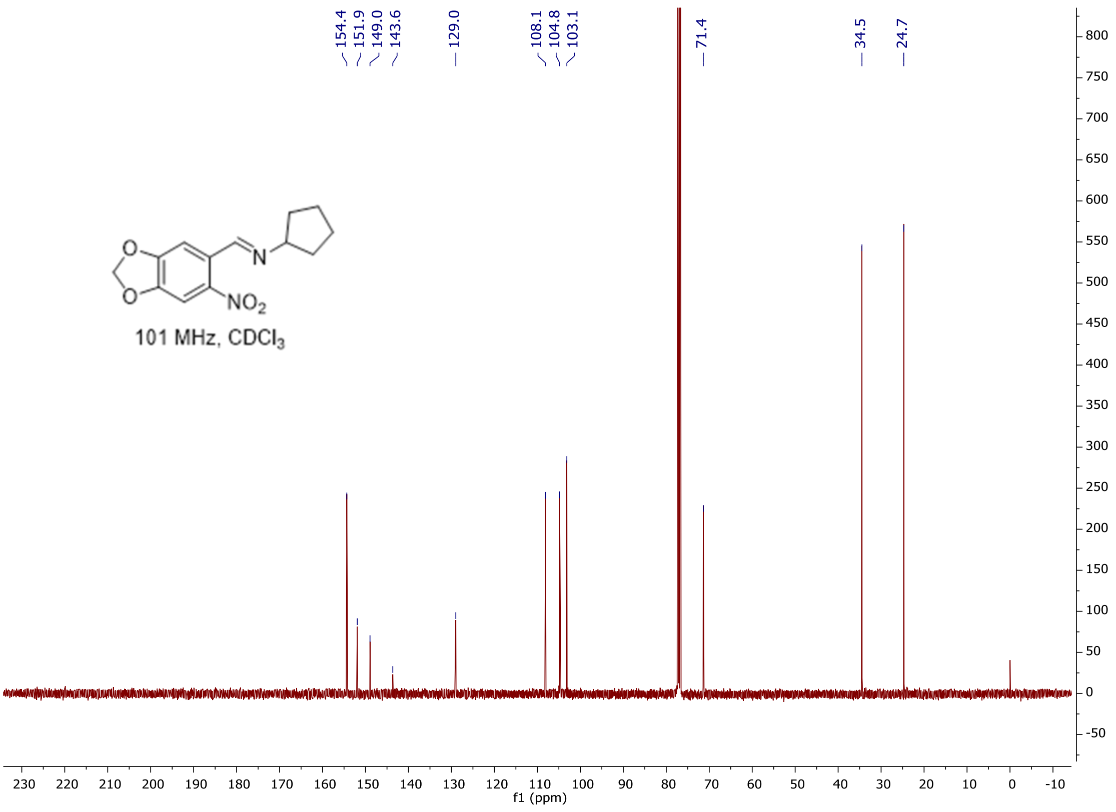

1n: methyl (*E*)-4-((*tert*-butylimino)methyl)-3-nitrobenzoate

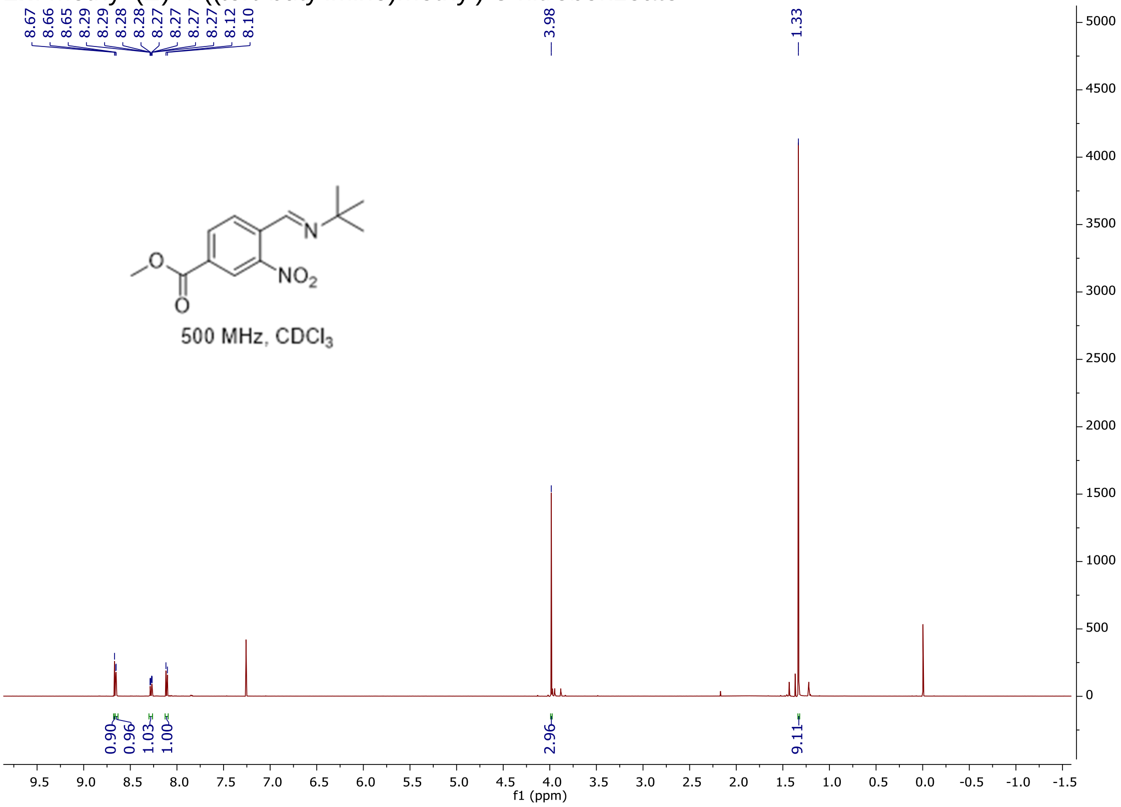

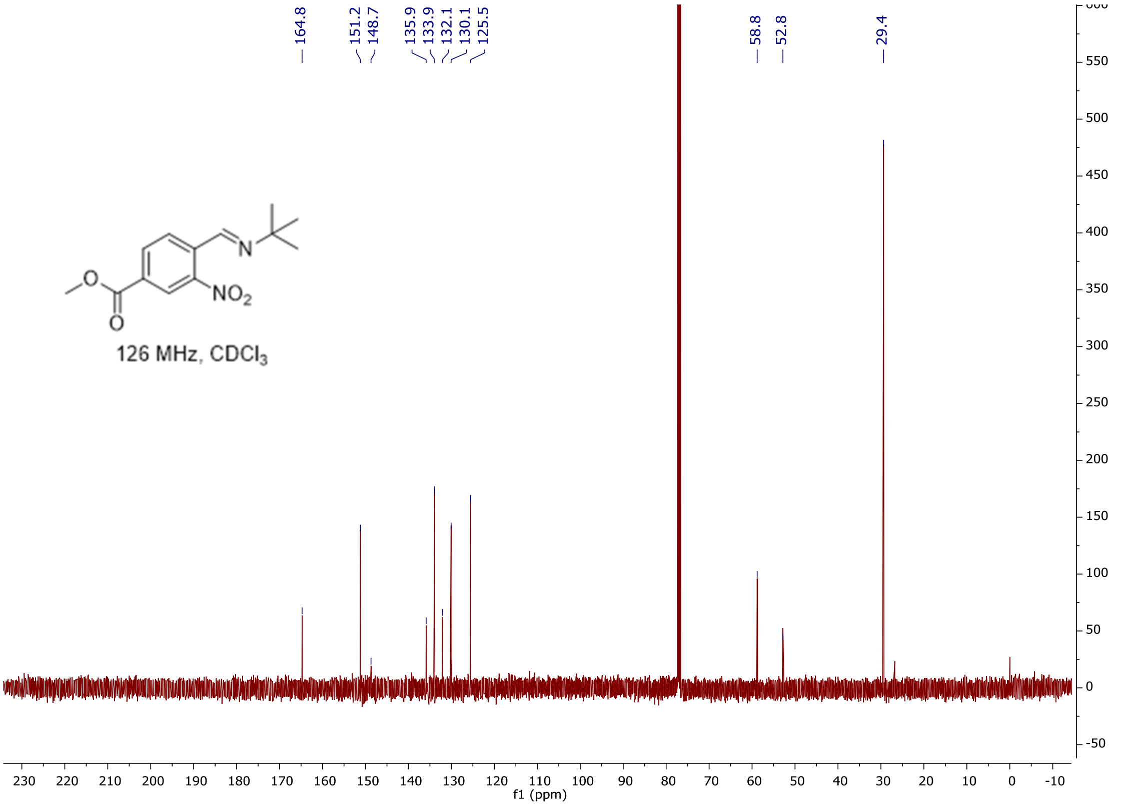

1o: (*E*)-*N*-*tert*-butyl-1-(3-methyl-2-nitrophenyl)methanimine

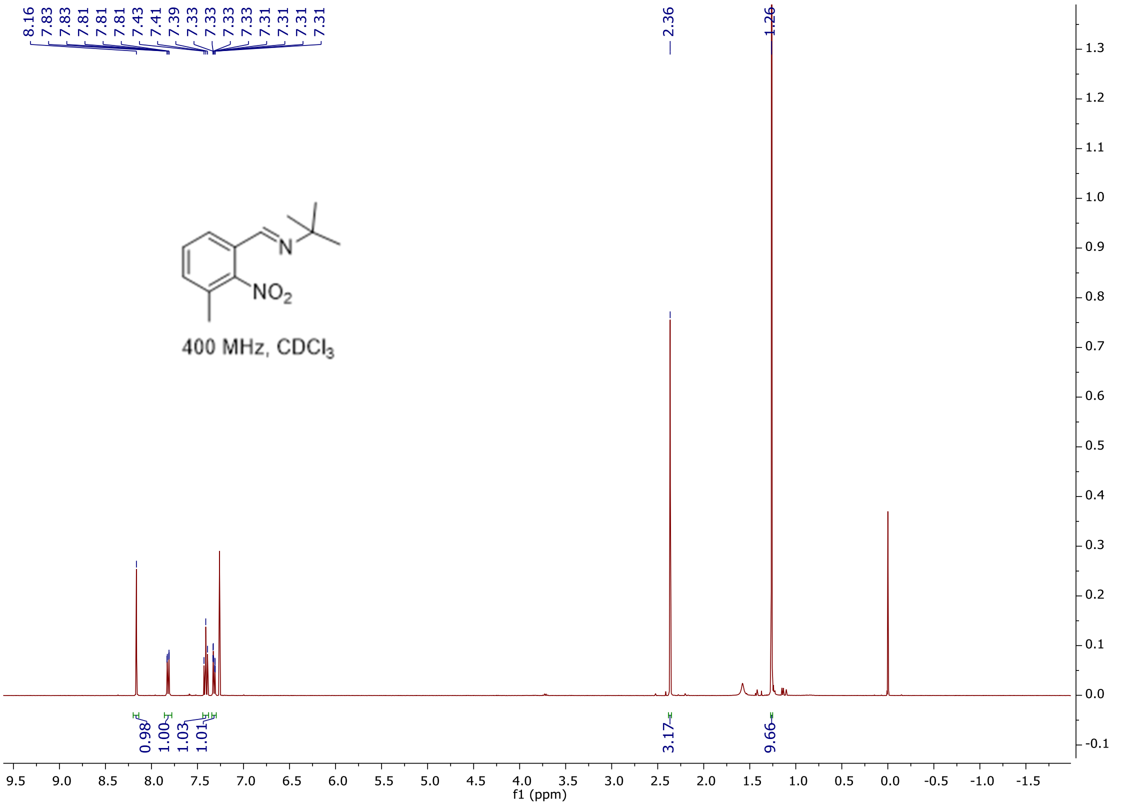

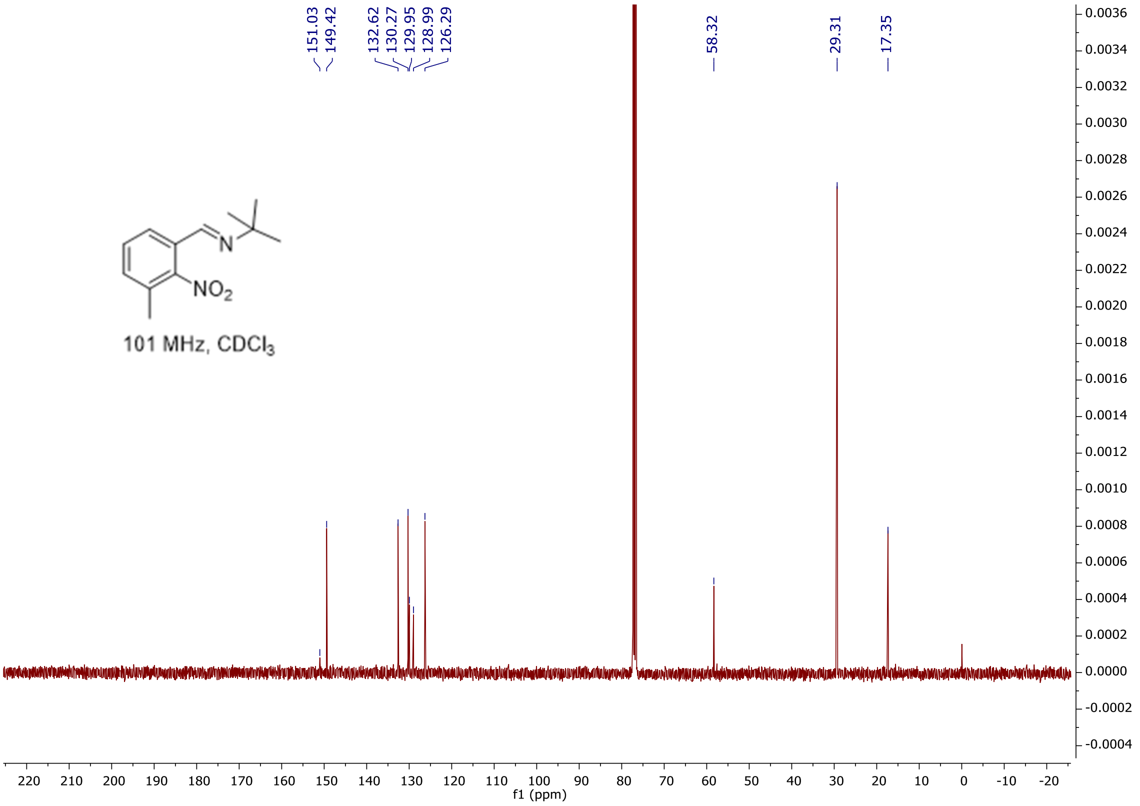

Supplement: Supplementary file 1 — jo3c02362_si_001.pdf [file jo3c02362_si_001.pdf]
